# Clinical, imaging and blood biomarker outcomes in a Phase 3 clinical trial of tau aggregation inhibitor hydromethylthionine mesylate in mild cognitive impairment and mild to moderate dementia due to Alzheimer’s disease

**DOI:** 10.1016/j.tjpad.2026.100480

**Published:** 2026-01-21

**Authors:** Claude M Wischik, Richard Stefanacci, Peter Bentham, Serge Gauthier, Henrik Zetterberg, Gordon K Wilcock, Lutz Froelich, Alistair Burns, Emer MacSweeney, Clive Ballard, Jin-Tai Yu, Tay Siew Choon, Vahe Asvatourian, Natalia Muehlemann, Jan Priel, Karin Kook, Tenecia Sullivan, Diane Downie, Sonya Miller, Carol Pringle, John M. D Storey, Tom Baddeley, Charles R Harrington, Lewis K Penny, Mohammad Arastoo, Roger Staff, Anca-Larisa Sandu, Helen Shiells, Serena Lo, Nafeesa Nazlee, Emily Evans, Claire Hull, Bjoern O Schelter

**Affiliations:** a Institute of Medical Sciences, University of Aberdeen, Aberdeen AB25 2ZD, UK; b TauRx Therapeutics Ltd., Aberdeen, AB24 5RP, UK; c Thomas Jefferson University, Philadelphia, PA, USA; d Birmingham and Solihull Mental Health NHS Foundation Trust, Birmingham, UK; e Department of Neurology and Neurosurgery, McGill University, Montreal, Quebec, Canada; f Department of Psychiatry and Neurochemistry, Institute of Neuroscience & Physiology, Sahlgrenska Academy at the University of Gothenburg, Mölndal, Sweden; g Clinical Neurochemistry Laboratory, Sahlgrenska University Hospital, Mölndal, Sweden; h Department of Neurodegenerative Disease, UCL Institute of Neurology, London, United Kingdom; i UK Dementia Research Institute at UCL, London, United Kingdom; j Hong Kong Centre for Neurodegenerative Diseases, Clear Water Bay, Hong Kong, China; k Wisconsin Alzheimer’s Disease Research Centre, University of Wisconsin School of Medicine and Public Health, University of Wisconsin-Madison, Madison, WI 53792, USA; l Emeritus Professor of Geratology, University of Oxford, Oxford, UK; m Department of Geriatric Psychiatry, Medical Faculty Mannheim, Central Institute of Mental Health, Heidelberg University, (SCU-B), Mannheim, Germany; n University of Manchester, Manchester, UK; o Re:Cognition Health London UK; p Clinical and Biomedical Sciences, Faculty of Health and Life Sciences, University of Exeter, Exeter, UK; q Department of Neurology, Huashan Hospital, Fudan University, Shanghai, China; r Cytel Inc. 675 Massachusetts Ave, Cambridge, MA 02139, UK; s Salamandra LLC, Bethesda, MD, USA; t Department of Chemistry, University of Aberdeen, Aberdeen AB24 3UE, UK; u Scottish Biologics Facility, University of Aberdeen, Aberdeen, UK; v Aberdeen Royal Infirmary NHS Grampian, Aberdeen Royal Infirmary, Foresterhill, Aberdeen AB25 2ZN, UK; w Aberdeen Biomedical Imaging Centre, School of Medicine, Medical Sciences and Nutrition, University of Aberdeen, Lilian Sutton Building, Foresterhill, Aberdeen, AB25 2ZD, UK; x Institute for Complex Systems and Mathematical Biology, University of Aberdeen, Aberdeen, UK

**Keywords:** Hydromethylthionine mesylate, Tau aggregation inhibitor, Alzheimer’s disease, Neurofilament light chain, Phosphorylated-tau 217

## Abstract

**Background:**

Hydromethylthionine mesylate (HMTM) targets tau pathology and has tau-independent symptomatic activity.

**Objectives:**

To evaluate the safety and efficacy of HMTM in participants with mild cognitive impairment (MCI) and mild to moderate dementia due to Alzheimer’s disease (AD).

**Setting:**

82 centres in Canada, European Union, United Kingdom and United States of America.

**Participants:**

A total of 598 amyloid β-PET positive participants were included; 44% (263) met clinical criteria for MCI due to Alzheimer’s disease and 56% (335) were diagnosed with mild to moderate dementia due to AD. **Intervention:** HMTM 16 mg/day and 8 mg/day were compared with methylthioninium chloride (MTC) 4 mg twice weekly, intended as an inactive urinary colourant to preserve blinding with respect to possible urinary discolouration caused by HMTM.

**Measurements:**

HMTM and MTC were compared on cognitive and functional endpoints for the first 52 weeks followed by all receiving HMTM 16 mg/day to 104 weeks in a modified delayed-start trial design. Biomarker outcomes included change in plasma levels of neurofilament light chain (NfL), pTau217 and MRI measures of grey matter atrophy.

**Results:**

It was not possible to demonstrate significant differences on the co-primary clinical endpoints (ADAS-cog_11_ and ADCS-ADL_23_) at 52 weeks due to symptomatic activity in the control arm. In participants with MCI, statistically significant differences in cognitive decline (ADAS-cog_13_) emerged at 78 weeks (*p* = 0·0291) and 104 weeks (*p* = 0·0308) between early- and delayed-start HMTM 16 mg/day in analyses specified prior to the 24-month database lock. Statistically significant cognitive improvement over baseline score was sustained for 78 weeks in the early start MCI group, with no significant cognitive or functional decline to 104 weeks. There was a significant reduction in progression of neurodegeneration measured by NfL change (*p* = 0·0291) at 52 weeks in the whole population, consistent with significant reductions in progression of grey matter atrophy at 52 and 104 weeks, and a reduction in progression of tau pathology (pTau217, *p* = 0·0165) in MCI. Headache (1·5%) and diarrhoea (1·2%) were the most frequent adverse effects.

**Conclusions:**

Although HMTM 16 mg/day arrested progression of neurodegeneration and reduced grey matter atrophy at 52 weeks, symptomatic activity in the control arm precluded separation of treatment arms at 52 weeks on primary clinical endpoints. In participants with MCI, significant clinical separation was seen only at 78 and 104 weeks. This effect has been confirmed in a further study. HMTM was well tolerated and has the potential to offer an accessible oral treatment option with a benign safety profile which could be delivered with minimal patient/physician burden.

## 1 Introduction

Given the expense, limited efficacy and safety concerns with currently approved intravenous treatments for Alzheimer’s disease (AD) targeting amyloid [1,2], there is growing interest in alternative approaches. The tau pathology of AD is an important potential target for a disease-modifying treatment [3]. We report the results of a Phase 3 trial (TRx-237–039) with hydromethylthionine mesylate (HMTM), an oral tau aggregation inhibitor which delivers hydromethylthionine (HMT). The design and rationale for the trial have been described in this journal previously [4]. We show here that HMT significantly reduced cognitive decline over 2 years in MCI due to AD, reduced progression of neurodegeneration and tau pathology measured by plasma biomarkers, and reduced progression of grey matter atrophy in hippocampus relative to the control population. HMTM has the potential to offer an accessible and convenient disease-modifying treatment which has a benign safety profile and is well tolerated by patients.

The tau pathology of AD has a strong correlation with clinical severity and brain atrophy [5], independent of amyloid β [6]. HMTM is a stabilised crystalline form of reduced methylthionine (MT) which delivers hydromethylthione (HMT) to the brain [7]. HMT is a potent tau aggregation inhibitor in vitro [8]. It works by binding the proteolytically stable truncated tau core of neurofibrillary tangle filaments, making it assembly-incompetent [9] and preventing propagation of tau aggregation [8,10]. In tau-transgenic mice, oral HMT inhibits progression of tau pathology and reverses associated behavioural deficits [8,10]. It also has a second independent symptomatic mode of action which increases hippocampal acetylcholine [11] via a mechanism thought to be linked to enhanced mitochondrial metabolism [12]. Both methylthioninium chloride (MTC) [13], an oxidised form of MT [8], and HMTM [14] have been shown to reverse scopolamine-induced learning deficits in a standard mouse model for symptomatic activity which does not have tau pathology.

In two earlier Phase 3 trials comparing HMTM 150–250 mg/day with 8 mg/day in mild-to-moderate AD [[15], [16], [17]], a threshold based on first-dose plasma levels being within the calibration range of the assay identified about 65 % of participants at the 8 mg/day dose whose exposure was above-threshold; these individuals experienced significantly less cognitive decline (ADAS-cog_11_) and whole brain volume loss over 15–18 months than those with subthreshold levels [15]. A response plateau was predicted above a theoretical dose of 16 mg/day [15]. Similar cognitive, functional and whole brain shrinkage reduction benefits were seen in a Phase 3 trial in behavioural-variant frontotemporal dementia [18].

All MT-derived compounds risk causing variable urinary discolouration. The 8 mg/day dose in these earlier studies had been intended as a therapeutically inactive urinary colourant to maintain blinding with respect to higher doses, based on a Phase 2 dose-finding trial using MTC which demonstrated dose-dependent clinical and functional neuroimaging benefits versus placebo at 138 mg/day [19]. In the present study (TRx-237–039; NCT03446001), MTC was given in the control arm at the even lower dose of 4 mg twice weekly. This was the minimum unit dose shown to permit blinding with respect to higher doses in Phase 1 studies. Regulatory authorities discouraged our proposed use of a true placebo combined with non-disclosure of urinary colouration information to efficacy raters because of risk of biasing clinical outcomes. Despite extensive evaluation of more than 30 potential compounds, none was found to be both safe and pharmacologically inert. Based on Phase 1 findings, a 4 mg twice-weekly MTC dose was adopted as the control because it was found to be the lowest able to blind volunteers receiving higher doses. It was assumed from the results of the earlier exposure-response studies [15] that this dose was too low to have pharmacological activity. However, the present study has shown that this assumption was incorrect.

As described in an earlier publication [4], TRx-237–039 was a two phase study in which 598 participants with a positive amyloid β-PET scan ranging in clinical disease severity from MCI to moderate AD were randomised to receive HMTM 16 mg/day or the MTC control over the first 52 weeks, after which all participants received HMTM 16 mg/day to 104 weeks in a modified delayed-start design. The trial was unable to meet its prespecified primary endpoints at 52 weeks because of unexpected symptomatic activity in the MTC arm. Statistically significant differences in cognitive function emerged at 78 and 104 weeks in MCI (44 %, 263/598) between early- and delayed-start HMTM 16 mg/day in analyses specified prior to the 24-month database lock. Statistically significant cognitive improvement over baseline score was sustained for 78 weeks in the early start MCI group, with no significant cognitive or functional decline to 104 weeks. There was a significant reduction in progression of neurodegeneration measured by plasma neurofilament light chain (NfL) change at 52 weeks in the whole population, consistent with significant reductions in progression of grey matter atrophy at 52 and 104 weeks in hippocampus and other brain regions vulnerable to tau pathology, and a reduction in progression of tau pathology measured directly by change in pTau217 in the MCI subgroup.

## 2 Methods

### 2.1 Study design and participants

A description of the rationale for the design of Study TRx-237–039 (NCT03446001; EudraCT: 2017–003,558–17) has been published previously [4]. In brief, TRx-237–039 compared HMTM as monotherapy at doses 16 mg/day and 8 mg/day with MTC 4 mg twice weekly on a varying schedule as control to ensure blinding. The initial 52-week blinded period was followed by a 52-week modified delayed start open label period in which all participants received 16 mg/day. The study was conducted at 82 study sites located in Canada, the European Union, United Kingdom, and United States of America. Participants had to be less than 90 years of age and have a clinical diagnosis of probable AD or MCI [20,21]. All were required to have a positive amyloid−β positron emission tomography (PET) scan at baseline and were therefore on the AD continuum [22]. They must have been community-dwelling with a mini mental state examination (MMSE) score of 16 to 27, and a CDR stage of 0·5 to 2 at screening (with at least one non-zero functional domain score for CDR 0.5). Concomitant use of acetylcholinesterase inhibitors (AChEIs) or memantine was not permitted.

### 2.2 Ethical conduct of the study

All patients provided written informed consent prior to enrolling in the study; legal representatives provided consent on behalf of patients with reduced decision-making capacity. Informants for the participants also provided consent for involvement. The study was conducted in accordance with the Declaration of Helsinki and the International Conference on Harmonisation Guidelines for Good Clinical Practice, and approval of the study protocol and all related documents was obtained from the appropriate Independent Ethics Committees and Institutional Review Boards for all study sites. An independent Data and Safety Monitoring Board was established for oversight of accruing safety information.

### 2.3 Randomisation and blinding

Eligible participants were randomised at baseline in a 4:1:4 ratio to receive HMTM 16 mg/day (administered as two 4 mg tablets twice daily), 8 mg/day (administered as one 4 mg tablet and one blank tablet containing only excipients twice daily), or excipient-only tablets given twice daily among which were included 4 mg MTC tablets given every 2–4 days (4 mg twice weekly). Details of sample size determination and statistical methods are provided in the Supplementary.

### 2.4 Procedures

Five post-baseline visits were scheduled during the double-blind treatment phase (Supplementary). Timed morning blood samples were collected pre-dose and at 1-, 2-, and 4-hours post dose at baseline and months 1 and 12 for pharmacokinetic and blood biomarker analyses. A single blood sample for apolipoprotein E (*APOE*) genotype was obtained from consenting participants prior to month 12. Eligibility and safety assessments were performed by an independent qualified medical assessor not involved in efficacy assessments. Three further visits were scheduled during the open-label treatment phase (Supplementary) with timed morning blood samples collected pre-dose and at 1-, 2-, and 4-hours post dose at month 24 for pharmacokinetic analyses.

Plasma NfL and tau phosphorylated at threonine 181 (pTau181) were prespecified biomarker outcomes in “Secondary Research Plan – Plasma Biomarker Analysis” finalised prior to the interim database lock at 52 weeks, with change in plasma NfL concentration at 52 weeks prespecified as the primary plasma biomarker outcome. As pTau217 has since become recognised as having greater diagnostic utility [[23], [24], [25]], this biomarker was also analysed at 52 and 104 weeks, as was grey matter atrophy using statistical parametric mapping (SPM) in MRI scans. Detailed methods and methods of statistical analyses are described in the Supplementary Methods.

### 2.5 Outcomes

The co-primary endpoints were comparison of change over 52 weeks in the 11-item Alzheimer's Disease Assessment Scale-Cognitive Subscale (ADAS-Cog_11_, determined from the administered 13-item ADAS-cog_13_ version) and the 23-item Alzheimer's Disease Cooperative Study Activities of Daily Living Scale (ADCS-ADL_23_). Secondary endpoints included change in whole brain volume (WBV) quantified using the Boundary Shift Integral (BSI) [26]. Subgroup analyses according to verified AD diagnosis at baseline (probable AD or MCI-AD) were prespecified. Similar comparisons were undertaken for change in plasma concentrations of NfL and pTau181 over 52-weeks, which were prespecified in the “Secondary Research Plan – Plasma Biomarker Analysis”, with change in NfL defined as the primary exploratory blood biomarker prior to unblinding of the double-blind phase. The protocol (Protocol included in the Supplementary) outlined several analyses of change over 104 weeks and these were specified in the final statistical analysis plan (SAP) prior to the 104-week database lock. These included comparisons of change over 104 weeks on the ADAS-cog_11_, ADAS-cog_13_ and ADCS-ADL_23_ scales and change in WBV. The prespecified comparisons were between participants receiving HMTM 16 mg/day and those initially randomised to the control MTC dose, with similar comparisons split according to baseline clinical diagnosis.

### 2.6 Comparisons with other patient groups

Comparisons of arms as originally randomised with external controls over 52 weeks were prespecified in the final protocol as secondary outcomes, with details specified in the final SAP prior to the 104-week database lock, using data available from placebo arms of clinical trials in comparable populations. A systematic literature review (SLR) has been conducted and eligible publications identified from the SLR provided for analysis. Further methodological details are provided for the meta-analytic comparison are provided in the Supplementary Methods.

### 2.7 Role of the funding source

The study was financed entirely by TauRx Therapeutics Ltd. TauRx took the lead in study design and conduct, data interpretation, and report preparation. This study included a scientific advisory board comprising external experts who contributed to data interpretation and report preparation. Data analysis was conducted by an external vendor (Cytel, Inc.), checked by and in some instances complemented by TauRx.

## 3 Results

**Fig. 1:**
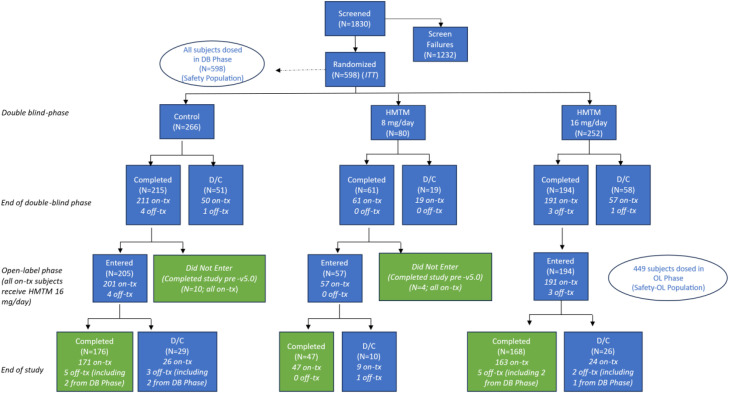
Trial profile. Abbreviations: DB=double-blind; D/*C*=did not complete; ITT=intention to treat; OL=open label; tx=treatment.

Between 2017 and 2023, we recruited 598 patients; the last patient visit was on 4th April 2023. Taking into consideration the subsequent 52-week open-label phase in which all patients received HMTM 16 mg/day, 537 patients received at least one dose of HMTM. The Intention-to-Treat (ITT) population included 598 participants (266 assigned to control (MTC 8 mg/week), 80 in the HMTM 8 mg/day group and 252 in the HMTM 16 mg/day group) of whom 470 (79 %) completed the study to 52 weeks (Fig. 1).

### 3.1 Patient populations

The baseline demographic and clinical characteristics of the population are presented in Table 1. The 598 patients in the primary efficacy analysis had a mean age of 72 years. Patients were predominantly Caucasian (88·1 %) and 60·7 % were female. With respect to diagnosis, 56 % of patients were diagnosed with mild-to-moderate dementia due to AD and 44 % with MCI. On average, participants had been diagnosed 2·6 years prior to randomisation. Overall, 372 participants (62·2 %) were treatment-naïve with respect to prior use of an AChEI and/or memantine.

**Table 1:** Demographic and baseline characteristics.

	**Control** **(MTC 4mg twice weekly)**	**HMTM 8mg/day**	**HMTM 16mg/day**	**Overall**
	*n* = 266	*n* = 80	*n* = 252	*n* = 598
Age (years)				
Mean (SD)	72.4 (8·3)	71·8 (8·1)	71·9 (8·6)	72·1 (8·4)
Median (range)	73·0 (37–89)	73·0 (48–87)	73·0 (41–88)	73·0 (37–89)
Sex, n ( %)				
Male	113 (42·5)	31 (38·8)	91 (36·1)	235 (39·3)
Female	153 (57·5)	49 (61·3)	161 (63·9)	363 (60·7)
Diagnosis, n ( %)				
MCI	116 (43·6)	42 (52·5)	105 (41·7)	263 (44·0)
AD	150 (56·4)	38 (47·5)	147 (58·3)	335 (56·0)
MMSE score				
Mean (SD)	21.6 (3·3)	21.9 (3·3)	21.5 (3·4)	21.6 (3·3)
26–27, n ( %)	43 (16.2)	14 (17.5)	46 (18·3)	103 (17·2)
16–25, n ( %)	223 (83·8)	66 (82.5)	206 (81·7)	495 (82.8)
Years since AD diagnosis, mean (SD)	2.7 (3·1)	2.8 (2·8)	2.5 (2·3)	2.6 (2·8)
History of using ChEI and/or memantine, n ( %)	104 (39·1)	28 (35)	94 (37·3)	226 (37·8)
Race, n ( %)				
Asian	0	2 (2·5)	0	2 (0·3)
Black or African American	10 (3.8)	2 (2·5)	8 (3·2)	20 (3·3)
Native Hawaiian or Other Pacific Islander	0	0	1 (0·4)	1 (0·2)
White	239 (89·8)	73 (91·3)	215 (85·3)	527 (88·1)
Unknown	15 (5·6)	3 (3·8)	24 (9·5)	42 (7·0)
Other	1 (0·4)	0	3 (1·2)	4 (0·7)
Multiple Races Checked	1 (0·4)	0	1 (0.4)	2 (0·3)
Ethnicity, n ( %)				
Hispanic or Latino	89 (60·1)	25 (52·1)	93 (67·9)	207 (62·2)
Geographic Region, n ( %)				
Europe	109 (41·0)	32 (40·0)	108 (42·9)	249 (41·6)
North America	157 (59·0)	48 (60·0)	144 (57·1)	349 (58·4)
Presence of *APOE* ε4 allele, n ( %)				
Positive	129 (55·1)	36 (49·3)	103 (45·8)	268 (50·4)
Negative	105 (44·9)	37 (50·7)	122 (54·2)	264 (49·6)

Within the MCI subgroup, baseline characteristics were consistent with those of the whole population (Supplementary Table 1). The mean age was similar between treatment arms (70.8 vs. 69.9 years), as were baseline MMSE scores (23.3 vs. 23.6). Additional baseline characteristics are presented in Supplementary Table 1.

### 3.2 Primary endpoints

**Fig. 2:**
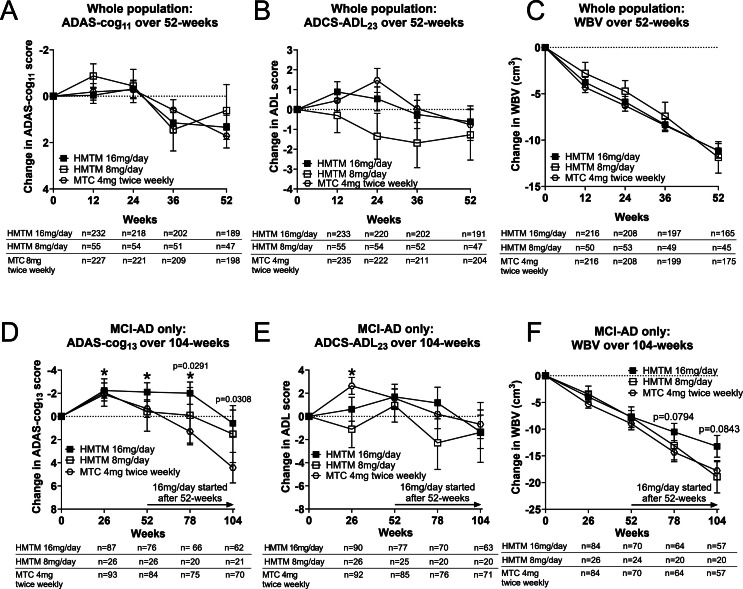
Cognitive, functional, and imaging endpoints. Prespecified efficacy results for the co-primary clinical, functional and secondary endpoints for all participants at 52-weeks: (A) ADAS-cog_11_ (B) ADCS-ADL_23_ and (C) whole brain volume (WBV). Corresponding outcomes in the MCI subgroup are shown at 104 weeks for: (D) ADAS-cog_13_, (E) ADCS-ADL_23_ and (F) WBV. p-values indicate statistical significance of differences between arms as randomised. Asterisks indicate statistically significant improvement over baseline; *P < 0.05. Error bars denote SEM.

The primary prespecified efficacy results for the co-primary clinical endpoints (ADAS-cog_11_ and ADCS-ADL_23_) and the principal secondary neuroimaging outcome (WBV) are summarized in Table 2 and Fig. 2. None of the comparisons of HMTM 16 mg/day was significantly different from MTC 4 mg twice weekly for the co-primary outcomes. Similarly, there was no difference in WBV between participants receiving HMTM 16mg/day and the MTC control (Fig. 2; Table 2).

**Table 2:** Prespecified treatment group comparisons at 52-weeks in the whole population in ADAS-cog_13_, ADCS-ADL_23_ and whole brain volume comparing HMTM 16mg/day versus MTC 4 mg twice weekly.

**Endpoint**	**Baseline**	**Statistic**	**52-weeks**	
**Whole population**			**Control (4mg twice weekly)**	**HMTM 16mg/day**
ADAS-cog_11_	19.16 (18.40, 19.92)	Mean Change (95 % CI)	1.66 (0.63, 2.69)	1.24 (0.21, 2.27)
		Mean Difference (95 % CI)	-	−0.421 (−1.84; 1.00)
		*p-value* versus *Control:*	-	0.5601
ADCS-ADL_23_	61.90 (60.91, 62.89)	Mean Change (95 % CI)	−0.89 (−2.45, 0.67)	−0.53 (−2.11, 1.05)
		Mean Difference (95 % CI)	-	0.36 (−1.81; 2.53)
		*p-value* versus *Control:*	-	0.7431
WBV (cm^3^)	972·87 (963.69, 982.06)	Mean Change (95 % CI)	−11.24 (−12.82, −9.66)	−11.01 (−12.60, −9.42)
		Mean Difference (95 % CI)		0.23 (−1.95; 2.41)
		*p-value* versus *Control:*	-	0.8359

TRx-237–039 was designed to allow comparison of participants continuing at the HMTM 16 mg/day dose (early starters) over 104-weeks with participants receiving MTC 4 mg twice weekly over the first 52-weeks, and who then switched to the HMTM 16 mg/day dose (delayed starters). Although the difference between HMTM 16 mg/day and MTC 4 mg twice weekly could not be demonstrated over 52-weeks, the predicted difference in cognitive decline between these arms as randomised could be demonstrated over a longer period of observation when split by diagnosis according to prespecified analyses in the open-label phase of the trial.

### 3.3 Outcomes in participants with MCI

In the MCI subgroup, there were statistically significant differences in cognitive decline (ADAS-cog_13_) over 78 (−3.3 units, *p* = 0·0291) and 104 weeks (−3·8 units, *p* = 0·0308; Fig. 2, Table 3). ADCS-ADL_23_ did not differ, and WBV change was near significant (*p* = 0.0794 and *p* = 0·0843; Fig. 2, Table 3). These differences could not be attributed to retention bias, as similar proportions completed 104-weeks in the HMTM 16 mg/day group (62/105, 59 %) and MTC 4 mg twice weekly group (70/116, 60 %). Similar differences were not seen in mild-to-moderate AD. Relative to baseline, MCI participants receiving 16 mg/day had statistically significant cognitive improvement over baseline on the ADAS-cog_13_ scale at 26, 52 and 78 weeks, with scores on ADAS-cog_13_ and ADCS-ADL_23_ not differing significantly from baseline at 104 weeks. MCI participants randomised to MTC 4 mg twice weekly showed a similarly significant initial improvement on ADAS-cog_13_ and ADCS-ADL_23_ over the initial 26 weeks, but by 104 weeks, their scores had declined significantly below baseline despite the switch to HMTM 16 mg/day at 52 weeks (ADAS-cog_13_ *p* = 0.0012; Fig. 2, Table 3).

**Table 3:** Prespecified treatment group comparisons at 78-weeks and 104-week in the MCI subpopulation in ADAS-cog_13_, ADCS-ADL_23_ and whole brain volume comparing participants originally randomised to HMTM 16 mg/day versus MTC 4 mg twice weekly.

**Endpoint**	**Statistic**	**78-weeks**		**104-weeks**	
		**Control (4mg twice weekly → HMTM 16mg/day)**	**HMTM** **16mg/day**	**Control (4mg twice weekly → HMTM 16mg/day)**	**HMTM** **16mg/day**
**MCI only**					
**ADAS-cog_13_**	n	75	66	70	62
	Baseline (95 % CI)	21.95 (19.86, 24.04)	22.65 (20.69, 24.62)	21.72 (19.47, 23.97)	23.03 (21.04, 25.02)
	Mean Change (95 % CI)	1.31 (−0.95; 3.57)	−2.01 (−3.92; −0.10)	4.42 (1.81; 7.03)	0.59 (−1.68; 2.86)
	Mean Difference (95 % CI)	−3.32 (−6.29; −0.34)		−3.83 (−7.30; −0.36)	
	*p-value for Mean Change*	0.2530	0.0391	0.0012	0.6073
	*p-value* versus *Control:*	-	0.0291	-	0.0308
**ADCS-ADL_23_**	n	76	70	71	63
	Baseline (95 % CI)	66.83 (65.63, 69.04)	66.43 (64.80, 68.05)	66.01 (64.25, 67.76)	67.66 (65.77, 69.55)
	Mean Change (95 % CI)	0.19 (−2.30; 2.67)	1.15 (−1.58; 3.88)	−0.68 (−3.15; 1.78)	−1.33 (−4.22; 1.56)
	Mean Difference (95 % CI)	0.97 (−2.68; 4.62)		−0.65 (−4.39; 3.09)	
	*p-value for Mean Change*	0.8820	0.4026	0.5829	0.3606
	*p-value* versus *Control:*	-	0.6015	-	0.7323
**WBV (cm^3^)**	n	68	64	64	57
	Baseline (95 % CI)	1006.50 (979.82, 1033.17)	988.29 (964.97, 1011.60)	1001.03 (974.97, 1027.08)	986.88 (961.47, 1012.29)
	Mean Change (95 % CI)	−14.33 (−17.18; −11.47)	−10.52 (−13.75; −7.30)	−17.77 (−21.12; −14.41)	−13.21 (−17.29; −9.14)
	Mean Difference (95 % CI)	3.80 (−0.45; 8.06)		4.55 (−0.63; 9.73)	
	*p-value for Mean Change*	<0.0001	<0.0001	<0.0001	<0.0001
	*p-value* versus *Control:*	-	0.0794	-	0.0843

Note: Baseline values and corresponding 95 % confidence intervals are calculated based on observed cases included in the analytic samples at 78 and 104 weeks.

### 3.4 Blood biomarker outcomes

**Fig. 3:**
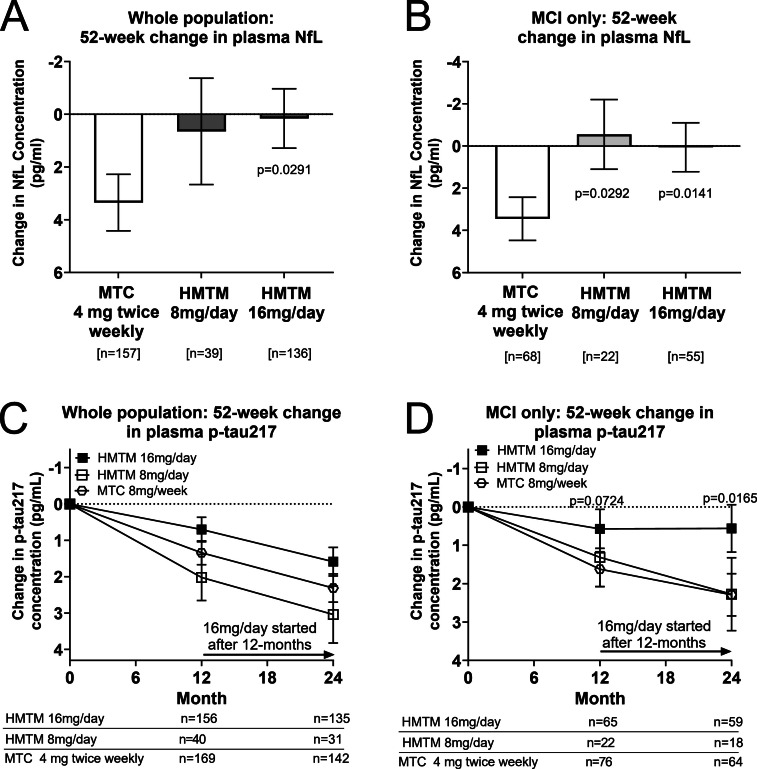
Plasma biomarker outcomes. Change in plasma neurofilament light chain (NfL) concentration over 52-weeks in (A) whole population and (B) MCI subgroup. Change in pTau217 concentration over 104-weeks in (C) whole population (D) MCI subgroup. Error bars denote SEM.

Baseline plasma concentrations of NfL and pTau217 were highly correlated with cognitive and neuroimaging measures of disease severity (Supplementary Table 3). HMTM 16 mg/day significantly reduced change in mean NfL plasma levels over 52 weeks by 95 % compared to the MTC control (*p* = 0·0291 overall; *p* = 0·0141 in MCI), producing a mean change statistically indistinguishable from zero; HMTM 8 mg/day produced a comparable result in MCI (*p* = 0·0292; Fig. 3).

Change in NfL was highly correlated with change in CDR-SB in MCI (*p* = 0.0002; Supplementary Figure 1). We also show a significant association between change in NfL and progression of dementia to CDR-global stages greater than 1 in the MCI subpopulation with both sources of data available for participants receiving HMTM 16 mg/day (NfL change, *p* = 0.0070; CDR progression, *p* = 0.04996, Supplementary Figure 2).

In the whole study population, there was no significant difference between treatment groups in change in plasma pTau217 at 52 or 104 weeks (Fig. 3C). In MCI, there was a statistically significant effect of HMTM 16 mg/day on change in pTau217 at 104-weeks (*p* = 0·0165; Fig. 3D). Change in pTau217 was significantly correlated with change at 104-weeks in ADAS-cog_13_ (*r* = 0.219, *p* = 0.0170), NfL (*r* = 0.269, *p* = 0.0016) and pTau181 (*r* = 0.355, *p* < 0.0001). Treatment with HMTM 16 mg/day uncoupled the predictive effect of baseline plasma concentration on change in pTau217. Although pTau217 and pTau181 were highly correlated at baseline (*r* = 0.774, *p* < 0.0001), the treatment effect of HMTM 16 mg/day on pTau181 was directionally consistent but did not reach statistical significance (*p* = 0·1699).

### 3.5 Significant treatment effects on grey matter atrophy

**Fig. 4:**
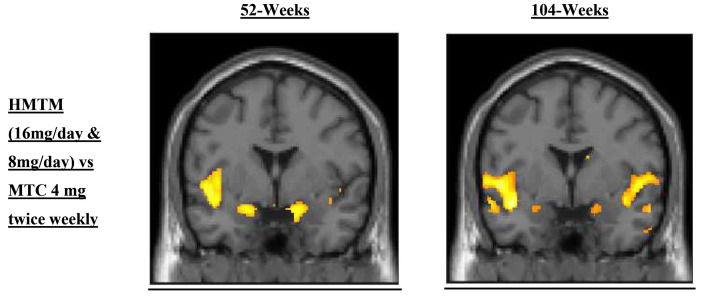
Statistical parametric mapping analysis comparing differences in change in GM volume over 52 and 104 weeks in subjects receiving HMTM (8mg/day or 16 mg/day) vs those receiving MTC 4 mg twice weekly. The locations of differences indicating statistically significant reduction in grey matter atrophy are shown in colour superimposed onto a normal control registered to a standardised template supplied with the SPM software platform (www.fil.ion.ucl.ac.uk/spm/).

Statistical parametric mapping revealed significantly less grey matter atrophy at both 52 and 104 weeks in participants randomised to HMTM (8 mg/day and 16 mg/day pooled) than in those randomised to MTC 4 mg twice weekly. Regions of reduced grey matter atrophy included hippocampus, parahippocampal gyrus, temporal lobes and parts of medial frontal lobe including cingulate gyrus (Fig. 4).

### 3.6 Comparison of MTC control with meta-analytic placebo controls

**Fig. 5:**
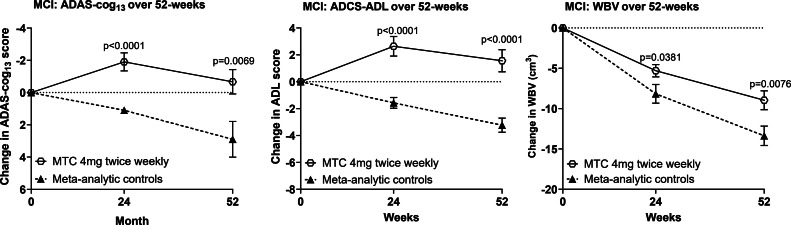
Change from baseline in cognitive, functional, and imaging outcomes in MCI comparing MTC 4 mg twice weekly versus meta-analytic controls. (A) ADAS-Cog_13_, (B) ADCS-ADL_23_, and (C) whole-brain volume over 52 weeks in participants with MCI treated with MTC 4 mg twice weekly compared with meta-analytic control data. Error bars represent the standard error of the mean (SEM).

To better understand the dissociation between clinical and biological outcomes at 52 weeks, we undertook a meta-analytic comparison of cognitive and functional decline between participants receiving MTC 4 mg twice weekly and the decline seen in placebo arms of clinical trials in comparable populations (ADAS-cog_13_: *n* = 2617; ADCS-ADL_23_: *n* = 2031; WBV: *n* = 2659). As can be seen in Fig. 5 and Supplementary Table 2, the clinical decline in the MTC control arm was significantly less than is typical of true placebo arms (Fig. 5, Supplementary Table 2; p-values 0.0381 to <0·0001), despite being significantly more impaired clinically at baseline by 1.2–2.2 MMSE units (p-values 0.0223 to <0·0001), depending on data availability for each outcome.

### 3.7 Safety profile

A total of 598 participants were randomised and treated in the TRx-237-039 double-blind period (Safety Population). The overall risk/benefit profile for HMTM remains unchanged relative to earlier studies [16], [17] (Table 4). Study completion was high (79 %), with no difference between the three treatment groups (76 % to 81 %). Overall, 328 of the 598 participants (55 %) had one or more treatment-emergent adverse events (TEAEs) reported during the double-blind phase. These events were considered at least possibly related to treatment by the Investigator in only 11 % of participants exposed to HMTM. The incidences were similar across the three treatment groups (apart from the HMTM 8 mg/day group which had greater variability due to small numbers). TEAEs reported in ≥1·0 % of participants randomized to HMTM (and at a greater incidence than in the control arm) are summarized in Table 4. Those considered treatment related were headache (1·5 %), diarrhoea (1·2 %), and anaemia, vomiting, gastroesophageal reflux disease, fatigue, anxiety, and somnolence (each <1 %).

**Table 4:** Treatment emergent adverse event (TEAE) during double-blind phase with an incidence of ≥1.0 % in subjects receiving MTC 4 mg twice weekly and HMTM (8mg/day, 16mg/day and pooled).

**MedDRA system preferred term**	**MTC** **4mg twice weekly (*N*=252)**	**HMTM**			
		**8mg/day** **(*N*=80)**	**16mg/day** **(*N*=252)**	**Pooled** **(*N*=332)**	
				**All**	**Related**
Participants with any TEAE, n ( %)	140 (55·6)	48 (60·0)	133 (52·8)	181 (54·5)	36 (10·8)
Anaemia	5 (2.0)	1 (1·3)	8 (3·2)	9 (2·7)	2 (0·6)
Diarrhoea	3 (1·2)	3 (3·8)	6 (2·4)	9 (2·7)	4 (1·2)
Gastrooesophageal reflux disease	2 (0·8)	0	4 (1·6)	4 (1·2)	2 (0·6)
Vomiting	1 (0·4)	3 (3·8)	5 (2·0)	8 (2·4)	1 (0·3)
Corona virus infection	11 (4·4)	6 (7·5)	9 (3·6)	15 (4·5)	0
Upper respiratory tract infection	2 (0·8)	3 (3·8)	1 (0·4)	4 (1·2)	0
Headache	7 (2.8)	5 (6·3)	10 (4·0)	15 (4·5)	5 (1·5)
Sciatica	0	0	4 (1·6)	4 (1·2)	0
Somnolence	1 (0·4)	2 (2·5)	3 (1·2)	5 (1·5)	3 (0·9)
Anxiety	6 (2·4)	2 (2·5)	6 (2·4)	8 (2·4)	1 (0·3)
Depressed mood	2 (0·8)	3 (3·8)	1 (0·4)	4 (1·2)	0
Insomnia	4 (1·6)	2 (2·5)	6 (2·4)	8 (2·4)	0
Fatigue	2 (0·8)	3 (3·8)	3 (1·2)	6 (1·8)	2 (0·6)
Hypertension	5 (2.0)	3 (3·8)	9 (3·6)	12 (3·6)	0

## 4 Discussion

*Key findings*. HMTM is the first oral treatment targeting the tau pathology of AD to reach late-stage development. The MCI subpopulation who represented 44 % of the randomised population was the most responsive to HMTM treatment. The latter group receiving HMTM 16 mg/day continuously for 104 weeks experienced sustained statistically significant cognitive improvement over baseline for 78 weeks and had no significant cognitive or functional decline over 104 weeks. This contrasted with participants initially randomised to MTC who experience statistically significant cognitive decline at 104 weeks despite starting on HMTM 16 mg/day after 52 weeks. These clinical benefits were accompanied by statistically significant biomarker benefits. Progression of neurodegeneration measured by change in plasma NfL at 52 weeks was stopped in the whole population with HMTM 16 mg/day and in MCI with both HMTM 8 mg/day and 16 mg/day. There was statistically significant reduction in tau aggregation pathology measured by change in plasma pTau217 at 104 weeks in MCI participants receiving HMTM 16 mg/day and significant reduction in grey matter atrophy in hippocampus and other brain regions vulnerable to tau pathology at 52 and 104 weeks measured by MRI statistical parametric mapping in participants receiving either 8 mg/day or 16 mg/day doses of HMTM.

*Control arm*. A major challenge in attempting to conduct placebo-controlled trials with HMTM is the risk of urinary discolouration. Although this is slight and varies between and within subjects over time, it hampers maintenance of the blind. We used HMTM 8 mg/day to control for this in three earlier Phase 3 trials, but found this to have statistically significant therapeutic activity in both AD [15] and bvFTD [18]. In the latest trial, we used an intermittent low dose of MTC in a further attempt to maintain blinding with respect to a target dose of HMTM 16 mg/day. As discussed in an earlier design methodology report [4], we initially proposed using a true placebo and keeping urinary discolouration information from efficacy raters, but regulatory authorities strongly encouraged the use of a urinary colourant rather than a true placebo because of risk of bias in reporting of clinical outcomes by participants. Although we investigated 30 compounds, we have been unable to identify any suitable alternative urinary colourant which could be given safely. The 4 mg unit dose was the lowest found to maintain blinding with respect to higher doses in Phase 1 studies, and MTC 4 mg given twice weekly on a varying schedule was the solution agreed with the regulatory authorities.

We now report that even this very low dose had clinical therapeutic activity over 52 weeks. This was demonstrated by highly significant differences on cognitive and functional outcomes in comparisons with meta-analytic controls based on true placebo data from trials in comparable populations in which participants received inactive infusions. MCI participants receiving MTC were significantly more impaired at baseline than in meta-analyses and yet performed significantly better on clinical outcomes. It seems unlikely that slight and variable urinary discolouration in a proportion of participants receiving MTC population could account for the differences relative to the more invasive placebo interventions where the likelihood of the 2-unit improvement over baseline seen with MTC at 26 weeks was less than 10^−6^. Plasma levels of drug were recently shown to be in the range required as a result of HMT's atypical pharmacokinetics [27], for symptomatic activity in preclinical studies [13,14]. Furthermore, the statistically significant clinical improvements over baseline scores that were observed at 26 weeks on ADAS-cog_13_ and ADCS-ADL_23_ were not seen in comparable amyloid-PET positive trial populations and are therefore unlikely to represent a placebo effect unique to the MTC population. The clinical improvement produced by MTC was not sustained and MCI participants originally randomised to this treatment declined significantly over 104 weeks despite being switched to the HMTM 16 mg/day dose after 52 weeks.

Participants with MCI starting treatment with HMTM 16 mg/day after a 52-week delay could not catch up cognitively with those starting treatment earlier. This was paralleled by a similar profile for change in pTau217. Participants starting the HMTM 16 mg/day dose after a delay of 52 weeks still had a significant increase in pTau217 concentration and differed significantly at 104 weeks from those starting the high dose earlier. Ongoing neurodegeneration demonstrated by increasing levels of plasma NfL and ongoing grey matter atrophy in participants receiving MTC for the first 52 weeks could not be compensated for by starting HMTM 16 mg/day after the 52-week delay. The inability of later-starters to catch up to those starting treatment earlier is the hallmark of a disease-modifying treatment.

*Biomarker outcomes*. Symptomatic activity at the lowest dose needed to maintain blinding did not confound the biomarker outcomes. HMTM 16 mg/day prevented the increase in NfL plasma concentration observed in the MTC 4 mg twice weekly population. This was seen in the whole population including both MCI and mild-to-moderate AD participants, although 8 mg/day was adequate for this effect in MCI participants. Change in plasma NfL concentration is generally recognised as a valid biomarker for neurodegeneration in AD [28], [29], although it is not specific for AD [30,31]. Plasma NfL concentration has a significant association with brain tau pathology as shown by tau-PET [32,33], is highly correlated with clinical severity, and levels at baseline are significant predictors of clinical decline 34. In the present study, there was a high correlation between baseline NfL and pTau217 concentrations at baseline. Unlike NfL, a significant effect on pTau217 required the HMTM 16 mg/day dose, even in MCI. pTau217 is highly correlated with clinical severity at baseline and with brain tau pathology [35], [36], [37]. Baseline level of pTau217 was a significant predictor of change in pTau217 in the present study. Since treatment with HMTM 16 mg/day had a significant effect on change in pTau217, the predictive relationship between baseline and change in pTau217 was uncoupled by treatment. The greater sensitivity of NfL as a plasma biomarker of treatment effect compared to pTau217 is most likely explained by NfL being a downstream indicator of convergent mechanisms of neurodegeneration, whereas the treatment effect on pTau217 is dependent on a single pathway.

*Limitations and blinding challenge*. The fundamental limitation of the present study is the lack of a true placebo control. This limitation had no effect on biomarker outcomes and therefore permitted the demonstration of statistically significant differences predicted from exposure-response analyses in earlier trials [38]. A key trial design choice must be made between maintaining blinding and allowing for the possibility of therapeutic activity in the control arm. In TRx-237–039, we elected to maintain blinding in accordance with strong regulatory recommendation. The net result of this choice was that TRx-237–039 did not have a valid placebo control over the first 52 weeks thereby preventing the study meeting its primary clinical endpoints, although prespecified and exploratory biomarker outcomes consistent with disease-modifying clinical efficacy were met after 52 weeks.

The present study has shown that it is not possible to control for urinary discolouration without therapeutic activity. Although a true placebo could be used with non-disclosure of discolouration information to clinical assessors, this would not permit control for possible bias in clinical assessment and biased withdrawal. A true placebo was used in the first dose-finding Phase 2 study with MTC, but there was biased withdrawal in the placebo arm, particularly of participants with more rapid clinical decline, resulting in reduced overall decline [19]. An alternative in circumstances where a traditional placebo is problematic is to use closely matched external control data. This approach has been recommended in regulatory guidances from the FDA [39], EMA [40] and MHRA [41]. We have therefore undertaken a further study comparing participants receiving HMTM 16 mg/day with true placebo subjects available from the Critical Path in Alzheimer’s Disease (CPAD) database who met the same inclusion/exclusion criteria and were matched on a range of key baseline covariates. As reported in an accompanying paper [38], this study was undertaken under a new protocol and SAP (TRx-237–080) and was successful in meeting its primary endpoints [38]. The results from both studies are consistent in demonstrating the efficacy of HMTM 16 mg/day on clinical and biomarker outcomes in AD.

*Safety*. The safety profile of HMTM as monotherapy (8 and 16 mg/day) is consistent with that seen in previous Phase 3 trials [16], [17], [18]. Overall, the frequency of adverse events was less than seen at the higher doses studied previously (150 – 250 mg/day), particularly diarrhoea and urinary frequency/urgency. The withdrawal rate from the double-blind phase was 21 %, and 97 % of participants who completed this phase elected to enter the delayed start phase. The present study confirms the absence of serotonin toxicity in participants taking antidepressants concomitantly and there were no cases of tyramine reaction. Importantly, the frequency of ARIA is comparable to previously reported placebo rates. Thus, the safety profile of HMTM remains benign and the drug is well tolerated.

*Conclusions*. In summary, HMTM is a novel and potentially first of a new drug class for the treatment of AD which combines symptomatic and disease-modifying activity on underlying neurodegeneration. The activity of a tau aggregation inhibitor in MCI is consistent with the increasing body of evidence that tau aggregation pathology is a critical driver of clinical decline and brain atrophy from the earliest detectable stages of AD [42], [43], and potentially prior to symptom onset [44]. HMTM is the first drug to combine sustained cognitive improvement over baseline in MCI and reduction in change in NfL and pTau217 concentration in plasma, and reduction in grey matter atrophy in regions particularly vulnerable to tau aggregation pathology. In participants with MCI, significant clinical separation was seen only at 78 and 104 weeks. This effect has been confirmed in a further study. Treatment with HMTM as monotherapy offers the possibility of meaningful intervention at an early clinical stage of AD reducing the rate of progression to more severe stages. HMTM is a simple oral potential treatment with a benign safety profile with no risk of safety concerns that require additional monitoring above routine clinical practice.

## Declaration of generative AI and AI-assisted technologies in the writing process

Generative AI and AI-assisted technologies were not used during the preparation of this manuscript.

## Funding

The study was financed entirely by TauRx Pharmaceuticals Ltd.

## Availability of data and materials

The datasets and analyses used during the current study are available from the corresponding author on reasonable request.

## CRediT authorship contribution statement

**Claude M Wischik:** Writing – review & editing, Writing – original draft, Methodology, Investigation, Conceptualization. **Richard Stefanacci:** Writing – review & editing, Writing – original draft. **Peter Bentham:** Writing – review & editing, Writing – original draft. **Serge Gauthier:** Writing – review & editing, Writing – original draft. **Henrik Zetterberg:** Writing – review & editing, Writing – original draft. **Gordon K Wilcock:** Writing – review & editing, Writing – original draft. **Lutz Froelich:** Writing – review & editing, Writing – original draft. **Alistair Burns:** Writing – review & editing, Writing – original draft. **Emer MacSweeney:** Writing – review & editing, Writing – original draft, Investigation. **Clive Ballard:** Writing – review & editing, Writing – original draft. **Jin-Tai Yu:** Writing – review & editing, Writing – original draft, Methodology. **Tay Siew Choon:** Writing – review & editing, Writing – original draft, Methodology, Conceptualization. **Vahe Asvatourian:** Writing – review & editing, Formal analysis. **Natalia Muehlemann:** Writing – review & editing, Formal analysis. **Jan Priel:** Writing – review & editing, Formal analysis. **Karin Kook:** Writing – review & editing. **Tenecia Sullivan:** Writing – review & editing. **Diane Downie:** Writing – review & editing, Writing – original draft, Methodology, Conceptualization. **Sonya Miller:** Writing – review & editing, Writing – original draft, Methodology, Conceptualization. **Carol Pringle:** Writing – review & editing, Writing – original draft. **John M. D Storey:** Writing – review & editing, Writing – original draft, Methodology, Conceptualization. **Tom Baddeley:** Writing – review & editing, Writing – original draft, Methodology, Conceptualization. **Charles R Harrington:** Writing – review & editing, Writing – original draft, Methodology. **Lewis K Penny:** Writing – review & editing, Methodology. **Mohammad Arastoo:** Writing – review & editing, Methodology. **Roger Staff:** Writing – review & editing, Methodology, Formal analysis. **Anca-Larisa Sandu:** Writing – review & editing, Formal analysis. **Helen Shiells:** Writing – review & editing, Writing – original draft. **Serena Lo:** Writing – review & editing, Writing – original draft. **Nafeesa Nazlee:** Writing – review & editing. **Emily Evans:** Writing – review & editing, Writing – original draft. **Claire Hull:** Writing – review & editing, Writing – original draft. **Bjoern O Schelter:** Writing – review & editing, Writing – original draft, Methodology, Conceptualization.

## Declaration of competing interest

Bjoern Schelter reports a relationship with GT Diagnostics that includes: employment. Serge Gauthier reports a relationship with MBI-C that includes: equity or stocks. Serge Gauthier, Henrik Zetterberg reports a relationship with AbbVie Inc that includes: consulting or advisory. Serge Gauthier reports a relationship with ADvantage that includes: consulting or advisory. Serge Gauthier reports a relationship with AmyriAD that includes: consulting or advisory. Serge Gauthier, Lutz Froelich, Alistair Burns reports a relationship with Eisai Inc that includes: consulting or advisory. Serge Gauthier reports a relationship with ENIGMA that includes: consulting or advisory. Serge Gauthier, Lutz Froelich, Clive Ballard, Alistair Burns reports a relationship with Eli Lilly that includes: consulting or advisory. Serge Gauthier reports a relationship with Otsuka Pharmaceutical Co Ltd that includes: consulting or advisory. Serge Gauthier, Lutz Froelich, Clive Ballard, Henrik Zetterberg reports a relationship with Novo Nordisk Inc that includes: consulting or advisory. Lutz Froelich, Clive Ballard reports a relationship with Biogen Inc that includes:. Lutz Froelich reports a relationship with Hoffmann-LaRoche that includes: funding grants. Lutz Froelich reports a relationship with Hector II Foundation that includes: funding grants. Lutz Froelich reports a relationship with Dietmar Hopp Foundation that includes: funding grants. Lutz Froelich reports a relationship with BioVie that includes: consulting or advisory. Lutz Froelich reports a relationship with GE Healthcare that includes: consulting or advisory. Lutz Froelich reports a relationship with Grifols Inc that includes: consulting or advisory. Lutz Froelich reports a relationship with Janssen Pharmaceuticals Inc that includes: consulting or advisory. Lutz Froelich reports a relationship with Neurimmune AG that includes: consulting or advisory. Lutz Froelich reports a relationship with Noselab that includes: consulting or advisory. Lutz Froelich, Clive Ballard, Henrik Zetterberg reports a relationship with Roche that includes: consulting or advisory. Lutz Froelich reports a relationship with Schwabe that includes: consulting or advisory. Clive Ballard reports a relationship with Novartis that includes: funding grants. Clive Ballard reports a relationship with Johnson and Johnson that includes: consulting or advisory and funding grants. Clive Ballard reports a relationship with ReMynd that includes: funding grants. Clive Ballard reports a relationship with Acadia Pharmaceuticals Inc that includes: funding grants. Clive Ballard reports a relationship with Acadia Pharmaceuticals Inc that includes: consulting or advisory. Clive Ballard reports a relationship with AARP that includes: consulting or advisory. Clive Ballard reports a relationship with BMS Pharmaceutical Ltd that includes: consulting or advisory. Clive Ballard reports a relationship with Janssen Pharmaceuticals that includes: consulting or advisory. Clive Ballard reports a relationship with Orion Corp that includes: consulting or advisory. Clive Ballard reports a relationship with Exciva that includes: consulting or advisory. Clive Ballard reports a relationship with Sumitomo Pharma America Inc that includes: consulting or advisory. Clive Ballard reports a relationship with Suven Pharmaceuticals Limited that includes: consulting or advisory. Henrik Zetterberg reports a relationship with Swedish Research Council that includes: funding grants. Henrik Zetterberg reports a relationship with Horizon Europe that includes: funding grants. Henrik Zetterberg reports a relationship with Swedish State Support for Clinical Research that includes: funding grants. Henrik Zetterberg reports a relationship with Alzheimer’s Drug Discovery Foundation that includes: funding grants. Henrik Zetterberg reports a relationship with AD Strategic Fund that includes: funding grants. Henrik Zetterberg reports a relationship with Alzheimer’s Association that includes: funding grants. Henrik Zetterberg reports a relationship with Bluefield Project that includes: funding grants. Henrik Zetterberg reports a relationship with Cure Alzheimer’s Fund that includes: funding grants. Henrik Zetterberg reports a relationship with Olav Thon Foundation that includes: funding grants. Henrik Zetterberg reports a relationship with Erling Persson Family Foundation that includes: funding grants. Henrik Zetterberg reports a relationship with Stiftelsen för Gamla Tjänarinnor that includes: funding grants. Henrik Zetterberg reports a relationship with Hjärnfonden that includes: funding grants. Henrik Zetterberg reports a relationship with European Union Joint Programme that includes: funding grants. Henrik Zetterberg reports a relationship with National Institute for Health and Care Research University College London Hospitals Biomedical Research Centre that includes: funding grants. Henrik Zetterberg reports a relationship with UK Dementia Research Institute that includes: funding grants. Henrik Zetterberg reports a relationship with Acumen Pharmaceuticals Inc that includes: consulting or advisory. Henrik Zetterberg reports a relationship with Alector Inc that includes: consulting or advisory. Henrik Zetterberg reports a relationship with Alzinova AB that includes: consulting or advisory. Henrik Zetterberg reports a relationship with ALZpath Inc that includes: consulting or advisory. Henrik Zetterberg reports a relationship with Amylyx Pharmaceuticals Inc that includes: consulting or advisory. Henrik Zetterberg reports a relationship with Annexon Biosciences that includes: consulting or advisory. Henrik Zetterberg reports a relationship with Apellis Pharmaceuticals, Inc that includes: consulting or advisory. Henrik Zetterberg reports a relationship with Artery Therapeutics Inc that includes: consulting or advisory. Henrik Zetterberg reports a relationship with AZTherapies that includes: consulting or advisory. Henrik Zetterberg reports a relationship with Cognito Therapeutics Inc that includes: consulting or advisory. Henrik Zetterberg reports a relationship with CogRx that includes: consulting or advisory. Henrik Zetterberg reports a relationship with Denali Therapeutics Inc that includes: consulting or advisory. Henrik Zetterberg reports a relationship with LABCORP that includes: consulting or advisory. Henrik Zetterberg reports a relationship with Merry Life that includes: consulting or advisory. Henrik Zetterberg reports a relationship with NervGen Pharma Corp that includes: consulting or advisory. Henrik Zetterberg reports a relationship with Optoceutics that includes: consulting or advisory. Henrik Zetterberg reports a relationship with Passage Bio Inc that includes: consulting or advisory. Henrik Zetterberg reports a relationship with Pinteon Therapeutics Inc that includes: consulting or advisory. Henrik Zetterberg reports a relationship with Prothena that includes: consulting or advisory. Henrik Zetterberg reports a relationship with Quanterix Corp that includes: consulting or advisory. Henrik Zetterberg reports a relationship with Red Abbey Labs that includes: consulting or advisory. Henrik Zetterberg reports a relationship with reMYND that includes: consulting or advisory. Henrik Zetterberg reports a relationship with Samumed that includes: consulting or advisory. Henrik Zetterberg reports a relationship with Siemens Healthineers that includes: consulting or advisory. Henrik Zetterberg reports a relationship with Triplet Therapeutics Inc that includes: consulting or advisory. Henrik Zetterberg reports a relationship with Wave that includes: consulting or advisory. Henrik Zetterberg reports a relationship with Brain Biomarker Solutions that includes: equity or stocks. Claude M Wischik has patent issued to WisTa Laboratories Ltd. John M.D Storey, Charles R Harrington has patent issued to WisTa Laboratories Ltd. If there are other authors, they declare that they have no known competing financial interests or personal relationships that could have appeared to influence the work reported in this paper.

## Appendix Supplementary materials

Image, application 1

Image, application 2
