# Supplementary material for: Clinical, imaging and blood biomarker outcomes in a Phase 3 clinical trial of tau aggregation inhibitor hydromethylthionine mesylate in mild cognitive impairment and mild to moderate dementia due to Alzheimer’s disease
Source: J Prev Alzheimers Dis. 2026 Jan 21;13(3):100480. doi: 10.1016/j.tjpad.2026.100480 (PMC12861207; doi:10.1016/j.tjpad.2026.100480)
Supplement: Supplementary file 1 [file mmc1.pdf]

CLINICAL STUDY PROTOCOL

Randomized, Double-Blind, Placebo-Controlled, Three-Arm, 12-Month, Safety and Efficacy Study of Hydromethylthionine Mesylate (LMTM) Monotherapy in Subjects with Alzheimer's Disease Followed by a 12-Month Open-Label Treatment

STUDY CODE: TRx-237-039

STUDY PHASE: 3

VERSION 7.1 DATED 16 JUNE 2023

Previous Versions:
VERSION 7.0 DATED 28 JULY2021
VERSION 6.0 DATED 09 OCT 2020
VERSION 5.1 DATED 05 FEB 2020
VERSION 5.0 DATED 09 JUL 2019
VERSION 4.1 DATED 09 NOV 2018
VERSION 4.0 DATED 24 AUG 2018
VERSION 3.0 DATED 31 MAY 2018
VERSION 2.1 DATED 25 OCT 2017
VERSION 2.0 DATED 31 AUG 2017
VERSION 1.0 DATED 23 AUG 2017

TauRx Therapeutics Ltd.
3 Shenton Way, #21-04
Shenton House
Singapore 068805
Republic of Singapore

Operational Location:
395 King Street
Aberdeen AB24 5RP
Scotland, UK
Tel: +44 (0) 1224 440905

THIS PROTOCOL IS A CONFIDENTIAL DOCUMENT

This protocol is the property of TauRx Therapeutics Limited. The information within it is confidential and is provided to you, for review by you, your staff and applicable Ethics Committees and Institutional Review Boards. The protocol must be kept in a confidential manner and must be returned to TauRx upon request. No part of this document may be reproduced in any form without permission from TauRx. By accepting this document, you agree that the information contained therein will not be disclosed to a third party without written authorization from TauRx.

1 TABLE OF CONTENTS

PAGE

1 TABLE OF CONTENTS 2
2 GCP COMPLIANCE STATEMENT 7
3 PROTOCOL APPROVAL 8
4 RESPONSIBLE PERSONNEL 9
5 INVESTIGATOR SIGNATURE SHEET 11
6 SYNOPSIS 12
7 ABBREVIATIONS 22
8 BACKGROUND AND RATIONALE FOR THE STUDY 25
8.1 Background 26
8.1.1 Investigational Product 26
8.1.2 Nonclinical Data 26
8.1.3 Clinical Data 28
8.2 Rationale 33
9 OBJECTIVES 34
9.1 Primary Objectives 34
9.2 Secondary Objectives 34
9.2.1 Double-Blind Treatment Period 34
9.2.2 Open-Label, Delayed-Start Phase 35
10 STUDY DESIGN 35
10.1 General Description 35
10.2 Changes Implemented Due to COVID-19 37
10.2.1 Changes to Study Conduct 38
10.2.2 Changes to Study Monitoring 41
10.2.3 Changes to Statistical Analyses 41
10.3 Study Population 42
10.4 Duration 43
10.5 Schedule of Assessments 43
10.5.1 Screening Assessments 43
10.5.2 Baseline and Post-randomization Assessments in Double-Blind Treatment Period 47
10.5.3 Assessments in the Open-Label, Delayed-Start Phase 51
10.6 Data and Safety Monitoring Board 54
10.7 Definition of End of Study 54
11 SUBJECT ENROLLMENT AND WITHDRAWAL 54
11.1 Inclusion Criteria 54
11.2 Exclusion Criteria 56
11.3 Re-screening 59

TABLE OF CONTENTS - continued

PAGE

11.4 Discontinuations / Withdrawals 60
11.4.1 Handling of Subjects Who Discontinue Study Drug 60
11.4.2 Handling of Study Discontinuation / Withdrawal 60
11.4.3 Replacements 61
12 STUDY DRUG 61
12.1 Treatments Administered: Form, Dosage and Administration 61
12.1.1 Active Ingredient 61
12.1.2 Inactive Ingredients 61
12.2 Study Regimens 62
12.2.1 Maximum Anticipated Dosage 62
12.2.2 Dose Interruption 63
12.3 Randomization 63
12.4 Packaging, Labeling, and Storage 63
12.5 Dispensing 64
12.6 Compliance 64
12.7 Study Drug Accountability 65
12.8 Breaking the Blind for Double-Blind Treatment Period 65
13 CONCOMITANT MEDICATIONS AND SUBJECT RESTRICTIONS 66
13.1 AChEI and/or Memantine 66
13.2 Drugs with Serotonergic Potential 67
13.3 CYP and P-gp Substrates 67
13.4 Drugs Used to Manage Behavioral Disturbance 67
13.5 Other Medications 68
13.6 Dietary Tyramine 68
13.7 Contraceptive Measures 68
13.8 Folate and Vitamin B_{12} 69
14 TERMINATION OF THE STUDY 70
15 STUDY ASSESSMENTS 70
15.1 Demographic Data/Medical History 70
15.2 Assessment of Efficacy 70
15.2.1 Raters 70
15.2.2 Instruments 72
15.3 Imaging Assessments and Procedures 75
15.3.1 General Considerations 75
15.3.2 Site Selection and Qualification 76
15.3.3 Imaging Methods for Efficacy 76
15.4 Safety Assessments and Procedures 78
15.5 Other Assessments 79
15.5.1 MT Concentration 79
15.5.2 Genotyping 80

TABLE OF CONTENTS - continued

PAGE

16 ADVERSE EVENTS AND SAFETY 80

16.1 Definition of AEs, Period of Observation, and Recording of AEs 80
16.2 Eliciting Adverse Event Information 81
16.3 Categorizing Intensity 81
16.4 Investigator's Brochure 82
16.5 Assessing Causality 82
16.6 Categorizing the Outcome 82
16.7 Serious Adverse Event Reporting 82
16.8 Suspected, Unexpected Serious Adverse Reactions and Emerging Safety Information 84
16.9 Malignancies 85
16.10 Reporting of Pregnancy 85
16.11 Guidance for the Handling of Adverse Events of Special Interest and Selected Test Abnormalities 86
16.11.1 Hemolytic Anemia 86
16.11.2 Lens Discoloration 86
16.11.3 Other Safety Reasons Requiring Discontinuation of Study Drug 86
16.12 Urgent Safety Measures 87

17 STATISTICAL ANALYSIS 87

17.1 Efficacy Endpoints 88
17.1.1 Primary Efficacy Endpoints for Double-Blind Treatment Period 88
17.1.2 Secondary Efficacy Endpoints for Double-Blind Treatment Period 89
17.1.3 Secondary Endpoint for Open-Label, Delayed-Start Phase 89
17.2 Number of Subjects and Sample Size Calculation 89
17.3 Analysis Populations 90
17.4 Clinical Efficacy and Imaging Analysis 91
17.4.1 Hypothesis 91
17.4.2 MITT (FDA) Analysis 92
17.4.3 ITT (EMA) Analysis 93
17.4.4 Estimands 93
17.4.5 Handling of Missing and Incomplete Data 95
17.4.6 Open-Label, Delayed-Start Analysis 96
17.4.7 Dose Response Analyses 96
17.4.8 Responder Analyses 96
17.4.9 Time-to-Event Analyses 97
17.4.10 Subgroup Analyses 98
17.4.11 Sensitivity Analyses 98
17.5 Exploratory Analyses 100
17.6 Demographic and Baseline Characteristics 102
17.7 Safety Analysis 102

TABLE OF CONTENTS - continued

PAGE

17.8 Other Data 103
17.9 Interim Analysis 103
18 REGULATORY AND ETHICS 104
18.1 Approval of the Protocol and Amendments 104
18.2 Serious Breaches 105
18.3 Informed Consent 105
18.4 Investigator Responsibilities 106
19 CONFIDENTIALITY AND DATA PROTECTION 107
20 STORED SAMPLES AND IMAGING DATA 108
20.1 Biosamples 108
20.2 Imaging Data 108
21 QUALITY ASSURANCE AND CLINICAL MONITORING 108
21.1 Standard Procedures 108
21.2 COVID-19 Risk Assessment 109
22 DOCUMENTATION 110
23 PUBLICATION 111
24 INDEMNITY, INSURANCE, AND COMPENSATION 111
25 ADMINISTRATIVE AND FINANCIAL AGREEMENT 111
26 STUDY ADMINISTRATION 111
27 REFERENCES 113
28 APPENDICES 117
28.1 Disallowed Treatments and Windows 117
28.1.1 Disallowed Medical Food / Medications Beginning 90 Days Before Baseline and During the Study 117
28.1.2 Disallowed Medications Beginning 60 Days Before Baseline and During the Study 117
28.1.3 Disallowed Treatments Beginning 28 Days Before Baseline and During the Study 117
28.2 Study Blood Assessments 118
28.3 Education / Occupation 119
28.3.1 Education 119
28.3.2 Occupation 119
28.4 Summary of Changes to the Protocol 120
28.4.1 Protocol Version 2.0 120
28.4.2 Protocol Version 2.1 121
28.4.3 Protocol Version 3.0 122
28.4.4 Protocol Version 4.0 127
28.4.5 Protocol Version 4.1 129
28.4.6 Protocol Version 5.0 130
28.4.7 Protocol Version 5.1 137

TABLE OF CONTENTS - continued

PAGE

28.4.8 Protocol Version 6.0 138
28.4.9 Protocol Version 7.0 142

2 GCP COMPLIANCE STATEMENT

This study will be conducted in compliance with the protocol, the principles contained in the Declaration of Helsinki, International Council for Harmonisation of Technical Requirements for Pharmaceuticals for Human Use (ICH) Integrated Addendum to ICH E6(R1): Guideline for Good Clinical Practice (GCP) E6(R2) (or ICH E6(R1) in those jurisdictions where ICH E6(R2) is not yet implemented by the regulatory authorities), and the applicable regulatory requirement(s).

3 PROTOCOL APPROVAL

**Sponsor Signatory**<br>[redacted]	
	Signature Date
**TauRx Medical Oversight**<br>[redacted]	
	Signature Date
**Statistician**<br>[redacted]	
	Signature Date

4 RESPONSIBLE PERSONNEL

**TauRx Global Project Lead(s)**<br>[redacted]<br>TauRx Therapeutics Ltd.<br>395 King Street<br>Aberdeen AB24 5RP<br>Scotland, United Kingdom	**Global Project Manager**<br>Marta Medina<br>Syneos Health<br>Direct [redacted]<br>Email: [redacted]<br>**Coordinating Investigator**<br>[redacted]
**North America Medical Monitor**<br>Medical Management and Scientific Services<br>Clinical Solutions<br>Syneos Health<br>Contact details for specific personnel are provided in the Site Contact List in the Investigator Site File	**Europe Medical Monitor**<br>Medical Management and Scientific Services<br>Clinical Solutions<br>Syneos Health<br>Contact details for specific personnel are provided in the Site Contact List in the Investigator Site File
**24-hour Medical Contact:**<br>**Emergency Scientific and Medical Services Global (ESMS Global)**<br>A current list of contact details is provided in the Site Contact List in the Investigator Site File	**Pharmacovigilance**<br>Syneos Health<br>Safety/Pharmacovigilance Department<br>Farnborough Business Park – 1 Pinehurst Road, Farnborough,<br>Hampshire, GU14 7BF<br>United Kingdom<br>Fax: [redacted]<br>Email [redacted]
**Investigational Medicinal Product Manufacturer (including packaging, labelling and distribution)**<br>Piramal Healthcare UK Ltd.<br>Piramal Pharma Solutions<br>Whalton Road<br>Morpeth, Northumberland NE61 3YA<br>United Kingdom<br>Tel: [redacted]	**Rater Training and Approval, Scale Management, Electronic Capture of Clinical Efficacy Data, Central Review of Assessments**<br>MedAvante-ProPhase Inc.<br>100 American Metro Blvd, Suite 106<br>Hamilton, New Jersey 08619<br>United States<br>Tel.: [redacted]
**Data Management**<br>Synteract, Inc.<br>5909 Sea Otter Place<br>Carlsbad, California 92010<br>United States<br>Tel: [redacted]	**Randomization and Trial Supply Management System**<br>Bioclinica, Inc.<br>800 Adams Avenue<br>Audubon, Pennsylvania 19403<br>United States<br>Tel: [redacted]

**Central Laboratory**<br>Labcorp Central Laboratory Services<br><br>**Americas**<br>Labcorp Central Laboratory Services LP<br>8211 SciCor Drive<br>Indianapolis, Indiana 46214<br>United States<br>Tel: [redacted]<br>Fax: [redacted]<br><br>**Europe**<br>Labcorp Central Laboratory Services S.à r.l.<br>7 Rue Moïse-Marcinhès<br>Meyrin, Geneva 1217<br>Switzerland<br>Tel: [redacted]<br>Fax [redacted]	**Magnetic Resonance Imaging (MRI) Imaging**<br><br>Suitably Qualified Vendor<br><br>**Positron Emission Tomography (PET)**<br>Invicro<br>60 Temple Street, Suite 8A<br>New Haven, Connecticut 06510<br>United States<br>Tel: [redacted]<br>Fax: [redacted]
**Methylthioninium (MT) Concentrations**<br>University of Aberdeen GLP Test Facility<br>Meston Building<br>Old Aberdeen<br>Aberdeen AB24 3UE<br>Scotland, United Kingdom<br>Tel: [redacted]<br>Fax [redacted]<br><br>Charles River Laboratories Edinburgh Ltd.<br>(cross-validation purposes only)<br>Elphinstone Research Centre<br>Tranent, East Lothian, EH33 2NE<br>United Kingdom<br>Tel: [redacted]	**Statistical Analysis of Pharmacokinetic Data**<br>Certara USA, Inc.<br>100 Overlook Center, Suite 101<br>Princeton, NJ 08540<br><br>Amsterdam office, Tauro office building,<br>Teleporboulevard 110, 104EJ Amsterdam,<br>The Netherlands<br>Tel: [redacted]
**Statistics**<br>Cytel Inc<br>Geneva Branch<br>Route de Prebois 20<br>1215 Geneva<br>Switzerland<br>Tel: [redacted]	

5 INVESTIGATOR SIGNATURE SHEET

By signing below, I agree to the conditions relating to this study as set out in this protocol (TRx-237-039 Version 7.1 dated XX June 2023).

I agree to conduct this study according to the principles contained in the Declaration of Helsinki, International Council for Harmonisation of Technical Requirements for Pharmaceuticals for Human Use (ICH) Integrated Addendum to ICH E6(R1): Guideline for Good Clinical Practice (GCP) E6(R2) (or ICH E6(R1) in those jurisdictions where ICH E6(R2) is not yet implemented by the regulatory authorities), the National Institute on Aging (NIA), the Alzheimer's Association (AA), and the applicable regulatory requirement(s).

I fully understand that any changes instituted by me without previous discussion with TauRx Therapeutics Limited or their designated representative constitute a deviation from the protocol.

I agree to adhere to the protocol in all circumstances other than where necessary to protect the well-being of the subject.

I will ensure that the drugs supplied by TauRx will be used only for administration to subjects enrolled in this study and for no other purpose.

Study Site Principal Investigator's Name, Title, Address and Contact Information:

____
____
____
____
____
____
____
____
____

Signature: Date:

6 SYNOPSIS

Name of Sponsor / Company: TauRx Therapeutics Ltd (TauRx)	
Name of Finished Product: LMTM (TRx0237) Film-coated Tablets, 4 mg	
Name of Active Ingredient (Drug Substance): Hydromethylthionine Mesylate	
**Number and Title of Study:** TRx-237-039: Randomized, Double-Blind, Placebo-Controlled, Three-Arm, 12-Month, Safety and Efficacy Study of Hydromethylthionine Mesylate (LMTM) Monotherapy in Subjects with Alzheimer's Disease Followed by a 12-Month Open-Label Treatment	
**Study Site(s):** Sites in North America (United States and Canada) and Europe	
**Study Duration:** The total duration of participation for an individual subject will be up to 120 weeks, including a Screening period of up to 16 weeks (112 days), a double-blind treatment period of 52 weeks, and a further open-label treatment period of 52 weeks. It is anticipated that the study will have an overall duration of at least 40 months, depending on recruitment rate. In addition, subjects who complete the study and receive treatment with LMTM up to and including the last open-label visit may be subsequently offered an opportunity to receive treatment with LMTM in a separate Expanded Access Program (EAP).	Phase of Development: Phase 3

Objectives

The primary objectives of the study pertain to the randomized, double-blind treatment period, listed below. The secondary objectives are presented separately for the double-blind treatment period and the open-label, delayed-start phase.

Primary (double-blind treatment period):

1. To compare the LMTM dose of 16 mg/day with the placebo group on the following co-primary endpoints:
   a. Alzheimer's Disease Assessment Scale, 11-item version (ADAS-cog_{11})
   b. Alzheimer's Disease Cooperative Study - Activities of Daily Living, 23-item version (ADCS-ADL_{23})
2. To assess the safety and tolerability of LMTM 16 mg/day given for up to 52 weeks

Secondary (double-blind treatment period):

3. To compare the LMTM dose of 16 mg/day with the placebo group in annualized rate of whole brain atrophy over 52 weeks as measured by brain magnetic resonance imaging (MRI) and quantified using the Boundary Shift Integral (BSI)
4. To compare the LMTM dose of 16 mg/day with the placebo group in temporal lobe ^{18}F-fluorodeoxyglucose positron emission tomography (^{18}F-FDG-PET) change in Standardized Uptake Value Ratio (SUVR) (normalized to pons) over 52 weeks, restricted to subjects with Clinical Dementia Rating (CDR) 0.5 at Screening, if a predefined threshold is reached for a sufficient number of subjects providing data
5. To compare the LMTM dose of 8 mg/day with the placebo group in temporal lobe ^{18}F-FDG-PET change in SUVR (normalized to pons) over 52 weeks, restricted to subjects with CDR 0.5 at Screening, if a predefined threshold is reached for a sufficient number of subjects providing data
6. To compare the LMTM dose of 8 mg/day with the placebo group on the co-primary endpoints (ADAS-cog_{11} and ADCS-ADL_{23})
7. To compare the LMTM doses of 8 and 16 mg/day with the placebo group in annualized rate of temporal and parietal lobe atrophy over 52 weeks as measured by MRI and quantified using the BSI
8. To assess the safety and tolerability of LMTM 8 mg/day given for up to 52 weeks

Secondary (open-label, delayed-start phase):

9. To determine if there is a difference in disease progression on the co-primary clinical endpoints and the MRI imaging endpoint for subjects who started treatment in the double-blind treatment phase and those who started treatment in the open-label, delayed-start phase (referred to as "early" and "late" LMTM starters, respectively)
   a. Only ADAS-cog_{11} will serve as a secondary endpoint; ADCS-ADL_{23} and other imaging endpoints are exploratory with the aim to be directionally supportive

Name of Sponsor / Company: TauRx Therapeutics Ltd (TauRx)
Name of Finished Product: LMTM (TRx0237) Film-coated Tablets, 4 mg
Name of Active Ingredient (Drug Substance): Hydromethylthionine Mesylate

10. To evaluate safety and tolerability of LMTM given for up to 104 weeks

Sensitivity analyses, including the evaluation of the secondary imaging endpoints to confirm the absence of treatment unblinding, are described in the protocol and Statistical Analysis Plan (SAP).

Population Pharmacokinetic (PK) analyses will be performed to estimate PK exposure in each subject for use in the evaluation of exposure-response relationships; these will be defined in a separate Population PK SAP or a dedicated section of the main SAP.

Additional exploratory analyses are described in the protocol and SAP. These include analyses of:

1. ADAS-cog_{13}
2. A new composite endpoint/composite endpoints based on selected item of the Alzheimer’s Disease Assessment Scale, 13-item version (ADAS-cog_{13}) and the ADCS-ADL_{23}, analyzed at 9 months and 12 months to evaluate its usefulness for future studies (for all subjects and also separately for subjects with CDR 0.5 and CDR 1-2 at Screening).
3. MMSE
4. CDR sum of boxes
5. Influence of Apolipoprotein E (*ApoE*) genotype

Study Design

This is a two-phase outpatient study of LMTM administered as monotherapy in 500 subjects with early to mild-moderate Alzheimer’s disease (AD): a randomized, double-blind, placebo-controlled, 52-week treatment period followed by a 52-week open-label phase that represents a modified delayed start of treatment. Subjects who are not receiving concomitant AChEI and/or memantine, for whom legally acceptable informed consent has been obtained and who are found eligible on the basis of screening evaluations, will be randomly assigned at Baseline to receive either LMTM 16 mg/day, LMTM 8 mg/day, or placebo; the drug supplies for the placebo group will include tablets containing a urinary discolorant, methylthioninium chloride (MTC), 4 mg. The primary treatment group comparisons during the double-blind treatment phase are of LMTM 16 mg/day and placebo. Following completion of the 52-week treatment period, all subjects (regardless of randomized treatment assignment or response) will continue open-label treatment with LMTM 16 mg/day for a further 52 weeks; prior treatment assignment will not be unblinded.

The Screening period is to be up to 9 weeks for subjects who are not receiving an AChEI and/or memantine at the time of signing the consent (Initial Screening Visit, Visit 1). For subjects who are receiving an AChEI and/or memantine, the Screening period may be extended for up to a further 6 weeks, 15 weeks in total (+7 days at Sponsor discretion), to allow for the performance of the necessary screening tests prior to the discontinuation of an AChEI and/or memantine and to permit a washout of at least 60 days from the last dose prior to the baseline assessments (inclusive of the baseline ^{18}F-FDG-PET scan in subjects who have a screening CDR of 0.5).

A sufficient number of subjects will be recruited from sites in North America and Europe such that approximately 500 subjects are enrolled. Beginning with Protocol Version 5.0, approximately 450 subjects are to be enrolled and randomized at the Baseline/Randomization Visit (Visit 2) to the LMTM 16mg/day (200 subjects), LMTM 8mg/day (50 subjects), or placebo (200 subjects) groups. Randomization will be stratified by severity (Mini-Mental State Examination [MMSE] 16-19, 20-25, or 26-27 at study level for those randomized to Protocol Version 5.0 and above), by prior use of AChEIs and/or memantine, and by region (grouped into North America or Europe). Subjects will be assigned to the MMSE severity groups based on screening MMSE score with a target ratio of approximately 2:3:1 (MMSE 16-19, 20-25, 26-27, respectively) for those randomized to Protocol Version 5.0 and above; to achieve this target, enrollment will be monitored and controlled at the site level for high recruiting sites (i.e., sites projected to recruit more than 10% of the subjects) and capped as needed at the study level.

Eight post-Baseline visits are scheduled: five during the double-blind treatment period (Visit 3: safety, and Visits 4, 5, 6, and 7: imaging, efficacy, and safety) and three during the open-label, delayed-start phase (Visits 8 [telephone contact only], 9, and 10). A summary of modifications to the post-Baseline visits that may be implemented in response to the Coronavirus Disease 2019 Public Health Emergency (hereafter referred to as COVID-19) is described in the body of the protocol. Unscheduled visits may occur as needed for assessment, or

Name of Sponsor / Company: TauRx Therapeutics Ltd (TauRx)
Name of Finished Product: LMTM (TRx0237) Film-coated Tablets, 4 mg
Name of Active Ingredient (Drug Substance): Hydromethylthionine Mesylate

upon early termination. In addition, subjects are to be followed as needed for the resolution or stabilization of an adverse event (AE), including following the last dose, consistent with the investigator's medical judgement.

Subjects who drop out after randomization will not be replaced (however, the study partner may be replaced if the current one withdraws/drops out); subjects will be encouraged to continue with study visits until the scheduled completion of the double-blind treatment period (Visit 7). Only subjects who continue in the study and receive LMTM treatment up to and including the last visit (Visit 10) without the addition of concomitant AChEIs and/or memantine may be eligible for a separate EAP.

Sample Size

Sample size estimations to achieve 90% power (two-sided alpha = 0.05) to detect a difference between LMTM 16 mg/day and placebo, the primary treatment group comparison in the double-blind treatment period, have been performed for the two co-primary clinical endpoints. These assume a withdrawal rate of 20% to 25% over 52 weeks. The study sample size of approximately 450 subjects enrolled under Protocol Version 5.0 and above (200 subjects in each treatment group, with a further 50 subjects for secondary analyses of an LMTM 8-mg/day group) is based on the ADCS-$ADL_{23,}$ since a larger sample size is required for this rating scale to achieve the target power.

Based on an estimated decline in ADCS-ADL_{23} over 52 weeks in the control arm of 7.7 units with an estimated standard deviation (SD) of 8.5 units, the study will have >90% power to detect a reduction in decline of 3.4 units or more. The 3.4 units are motivated by an estimated treatment effect of 5.0 ± 1.6 (mean ± standard error) units in the pooled studies TRx-237-005/TRx-237-015.

Based on an estimated decline in ADAS-cog_{11} over 52 weeks based on pooled information from studies TRx-237-005/TRx-237-015 in the control arm of 6.5 units with an estimated SD of 5.9 units, 200 subjects per treatment arm provide >90% power to detect a reduction in decline of 2.6 units or more. The 2.6 units represent a conservative value as the estimated treatment effect based on pooled studies TRx-237-005/TRx-237-015 is 5.2 ± 1.3 (mean ± standard error) units.

With 200 subjects randomized to the primary comparison in the double-blind treatment period under Protocol Version 5.0 and above, 160 to 170 subjects per arm will enter the open-label, delayed-start treatment phase assuming the 20-25% drop-out rates mentioned above. Assuming a further 10% drop out in the delayed-start phase, the key secondary analysis to demonstrate disease modification by comparing early to late starters using a non-inferiority margin of -2 ADAS-cog_{11} units has approximately 80% power.

Subject Population

Inclusion Criteria:

1. AD, encompassing probable AD and mild cognitive impairment due to AD (MCI-AD) based on 2011 National Institute on Aging (NIA) / Alzheimer's Association (AA) criteria:
   - All cause dementia and probable AD (probable AD)
     In brief, subjects with probable AD dementia must have insidious onset, worsening impairment in at least two cognitive areas (learning and recall, language, executive function, visuospatial skills), sufficient to significantly interfere with work or usual activities, that is not explained by delirium, drugs, major psychiatric disorder, medical illness, cerebrovascular disease, other forms of dementia, or neurological disorder. The accuracy of the diagnosis will be confirmed independently by the diagnosing physician at site
     OR
   - MCI-AD
     In subjects with MCI-AD, there should be evidence of concern about a change in cognition, in comparison with the person's previous level verified by a knowledgeable informant or clinician. Other mild cognitive deficits may also be present, but there must be preservation of independence in functional abilities. Subjects should not meet the criteria for dementia. The cognitive changes must be mild and there must be no evidence of a significant impairment in social or occupational functioning. Impairments must not be explained by delirium, drugs, major psychiatric disorder, medical illness, cerebrovascular disease, other forms of dementia, or neurological disorder.
2. Documented PET scan that is positive for amyloid; if most recent PET scan was performed >3 years prior to Screening and was negative, it may be repeated (a negative amyloid PET scan within the 3 years prior to Screening is exclusionary)

Name of Sponsor / Company: TauRx Therapeutics Ltd (TauRx)
Name of Finished Product: LMTM (TRx0237) Film-coated Tablets, 4 mg
Name of Active Ingredient (Drug Substance): Hydromethylthionine Mesylate

3. MMSE score of 16-27 (inclusive) at Screening, subject to stratification requirements
4. Global CDR score of 0.5 to 2 at Screening (if 0.5, including a score of > 0 in one of the functional domains: Community Affairs, Home and Hobbies, or Personal Care)
5. Age < 90 years at Screening
6. Females must meet one of the following:
    - Surgically sterile (hysterectomy, bilateral salpingectomy / oophorectomy) for at least 6 months minimum
    - Have undergone bilateral tubal occlusion / ligation at least 6 months prior
    - Post-menopausal for at least 1 year
    - Using adequate contraception (a barrier method [such as condom, diaphragm or cervical/vault cap] with spermicidal foam, gel, film, cream, or suppository; intrauterine device [IUD] or system, or oral or long-acting injected or implanted hormonal contraceptives for at least 90 days prior to Baseline; or vasectomized partner [with the appropriate post-vasectomy documentation of the absence of spermatozoa in the ejaculate]) or true abstinence (when this is in line with the preferred and usual lifestyle of the subject); subjects must be competent to use adequate contraception and to agree to continue to maintain adequate contraception throughout participation in the study (including up to 4 weeks after the last dose of study drug)
7. Subject and/or, in the case of reduced decision-making capacity, legally acceptable representative(s) (LAR(s)), consistent with local and national law, is able to read, understand and provide written informed consent in the designated language of the study site
8. Has one (or more) identified adult study partner (*i.e.*, a caregiver or informant) who meets the following criteria:
    - Either lives with the subject, or in the investigator's opinion, the extent of contact is sufficient to provide meaningful assessment of changes in subject behavior and function over time and provide information on safety and tolerability (*e.g.*, sees the subject on average for ≥1 hour/day ≥3 days/week)
    - Is willing to provide written informed consent for his/her own participation
    - Is able to read, understand, and speak the designated language at the study site
    - Agrees to accompany the subject to each study visit
    - Is able to verify compliance with study drug
9. The subject must not have been taking either an AChEI, *i.e.*, donepezil, galantamine, or rivastigmine, and/or memantine, for at least 60 days at the time of the baseline assessments
    - Subjects never previously treated with an AChEI and/or memantine may be enrolled if initiation of treatment with these medications is not planned for the time period during which the subject will be participating in this study
10. Able to comply with the study procedures in the view of the investigator

Exclusion Criteria:

1. Significant central nervous system (CNS) disorder other than probable AD or MCI-AD, *e.g.*, Lewy body dementia, Parkinson's disease, multiple sclerosis, progressive supranuclear palsy, hydrocephalus, Huntington's disease, any condition directly or indirectly caused by Transmissible Spongiform Encephalopathy (TSE), Creutzfeldt-Jakob Disease (CJD), variant Creutzfeldt-Jakob Disease (vCJD), or new variant Creutzfeldt-Jakob Disease (nvCJD)
2. Significant intracranial focal or vascular pathology seen on brain MRI scan that would, based on the independent reviewer imaging evaluation, lead to a diagnosis other than probable AD or MCI-AD, including but not limited to:
    - Large confluent white matter hyperintense lesions (*i.e.*, Fazekas score of 3)
    - Other focal brain lesions judged clinically relevant by the investigator
    - Evidence of a prior or current macrohemorrhage
3. Clinical evidence or history of any of the following (within specified period prior to Baseline):
    - Cerebrovascular accident (2 years)
    - Transient ischemic attack (6 months)
    - Significant head injury, for example, associated loss of consciousness, skull fracture or persisting cognitive impairment (2 years)

Name of Sponsor / Company: TauRx Therapeutics Ltd (TauRx)
Name of Finished Product: LMTM (TRx0237) Film-coated Tablets, 4 mg
Name of Active Ingredient (Drug Substance): Hydromethylthionine Mesylate

- Other unexplained or recurrent loss of consciousness (2 years)

4. Diagnosed with epilepsy (a single prior seizure >6 months prior to Screening, is considered acceptable)
5. Diagnostic and Statistical Manual of Mental Disorders, Fifth Edition criteria met (for any of the following within specified period):
   - Major depressive disorder (current)
   - Schizophrenia (lifetime)
   - Other psychotic disorders, bipolar disorder (within the past 5 years)
   - Substance (including alcohol) related disorders (within the past 2 years)
6. Metal implants in the head (except dental), pacemaker, cochlear implants, or any other non-removable items that are contraindications to MRI. MRI compatible prosthetics, clips, stents, or any other device proven to be compatible are allowed
7. Resides in hospital or moderate to high dependency continuous care facility (residence in low grade assisted living facility where there is sufficient autonomy to permit valid evaluation of activities of daily living is allowed so long as it is not mandated by an order issued either by the judicial or the administrative authorities)
8. Any physical disability that would prevent completion of study procedures or assessments (*e.g.*, blindness or significant uncorrected visual impairment, deafness or significant hearing loss not corrected by hearing aids, non-AD-related speech impairment)
9. History of swallowing difficulties (note: study drug should be swallowed whole and MUST NOT be broken, crushed, chewed, or dissolved in fluids prior to ingestion)
10. Pregnant or breastfeeding
11. Glucose-6-phosphate dehydrogenase (G6PD) deficiency based on World Health Organization classification (<60% of normal, *i.e.*, <6.1 U/g hemoglobin [Hgb])
12. History of significant hematological abnormality or current acute or chronic clinically significant abnormality, including:
   - History of hemoglobinopathy, myelodysplastic syndrome, hemolytic anemia, or splenectomy
   - Screening Hgb value (confirmed upon repeat) below age/sex appropriate lower limit of the central laboratory normal range

   Subjects in whom folate is <4.0 ng/mL may be entered into the study provided folate supplementation (approximately 1 mg/day) is initiated and maintained for the duration of the study.
   Subjects in whom vitamin B_{12} is <150 pg/mL can be allowed if the investigator confirms that it does not affect the cognitive state of the subject and that the subject is supplemented as appropriate prior to the initiation of study drug
13. Abnormal serum chemistry laboratory value at Screening deemed to be clinically significant by the investigator. In addition, subjects with either of the following abnormalities must be excluded:
   - Creatinine clearance <30 mL/min, estimated by the central laboratory according to the Cockcroft and Gault equation
   - Thyroid stimulating hormone (TSH) above laboratory normal range (subject may be treated [if clinically indicated based on further laboratory testing] and re-screened after 90 days)
14. Clinically significant cardiovascular disease or abnormal assessments (based on the investigator's interpretation of the locally obtained electrocardiogram [ECG]) such as:
   - Hospitalization for acute coronary syndrome (acute myocardial infarction or unstable angina) or symptoms consistent with angina pectoris, within the 12 months preceding Baseline
   - Signs or symptoms of clinical heart failure within the 12 months preceding Baseline
   - Atrial fibrillation on screening ECG or history of atrial fibrillation that is not currently controlled (heart rate ≥ 85 bpm and/or inappropriate anticoagulation)
   - QTcF (QT corrected for heart rate using Fridericia's formula) at Screening >460 msec in males or > 470 msec in females, or low or flat T waves making measurement of QT interval unreliable
   - Recent history of poorly controlled hypertension, systolic blood pressure >180 mmHg, or diastolic blood pressure >100 mmHg, after 5 minutes in a seated position at Screening
   - Hypotension: systolic blood pressure <100 mmHg after 5 minutes in a seated position at Screening

Name of Sponsor / Company: TauRx Therapeutics Ltd (TauRx)
Name of Finished Product: LMTM (TRx0237) Film-coated Tablets, 4 mg
Name of Active Ingredient (Drug Substance): Hydromethylthionine Mesylate

- Heart rate <48 bpm or >96 bpm by measurement of vital signs (after 5 minutes in a seated position) or by local ECG at Screening

15. Pre-existing or current signs or symptoms of respiratory failure, *e.g.*, caused by chronic obstructive pulmonary disease, bronchial asthma, lung fibrosis, or other disease
    - Subjects with currently diagnosed moderate to severe sleep apnea should be excluded; the definition of moderate to severe includes oxygen supplementation, *e.g.*, nasal prongs or Continuous Positive Airway Pressure (CPAP)
16. Concurrent acute or chronic clinically significant (in the opinion of the investigator) immunologic, hepatobiliary (such as presence of encephalopathy or ascites), or endocrine disease (not adequately treated), and/or other unstable or major disease other than probable AD or MCI-AD; the following are specifically excluded:
    - Active hepatitis or primary biliary cirrhosis
    - Active Human T-Cell Lymphocytic Virus Type III (HTLV-III), Lymphadenopathy Associated Virus (LAV), any mutants or derivatives of HLTV-III or LAV, any condition associated with active Acquired Immunodeficiency Syndrome or similar condition however named
17. Diagnosis of cancer (excluding basal cell carcinoma, squamous cell carcinoma, or prostate carcinoma in situ [Stage 1]) meeting either of the following criteria:
    - Newly diagnosed within past 2 years
    - Previous (>2 years) diagnosis of cancer that has required any form of intervention or treatment within the past 2 years, *e.g.*, chemotherapy, radiotherapy, hormonal therapy, or surgery
18. Prior intolerance or hypersensitivity to MT-containing drug or methemoglobinemia induced by MT-containing drug, similar organic dyes, or any of the excipients
19. Treatment currently or within 90 days before Baseline with any of the following:
    - Souvenaid®
    - Antipsychotics
        - Clozapine (and there is no intent to initiate therapy during the course of the study)
        - Other antipsychotics are allowable provided they have not been initiated within 90 days before Baseline and preferably at a stable dose and regimen
    - Carbamazepine, primidone, valproate
    - Drugs for which there is a warning or precaution in the labeling about methemoglobinemia at approved doses (*e.g.*, dapsone, local anesthetics such as benzocaine used chronically, primaquine and related antimalarials)
20. Current or prior participation in a clinical trial as follows:
    - Any clinical trial of LMTM
    - Clinical trial of a product for cognition prior to Baseline in which the last dose was received within 90 days prior to Baseline unless confirmed to have been randomized to placebo
    - A clinical trial of any other investigational drug, biologic, device, or medical food in which the last dose was received within 28 days prior to Baseline

Dose/Route/Regimen

Throughout the study, all subjects will receive four tablets orally per day (two in the morning and two in the evening), to be taken at the same time (and same meal condition) to the extent possible. Study drug should be swallowed whole and MUST NOT be broken, crushed, chewed, or dissolved in fluids prior to ingestion.

For the 52-week, double-blind treatment period, subjects will be randomized 4:1:4 (beginning with Protocol Version 5.0) to one of the following treatment groups:

- LMTM 16-mg/day group: Two 4-mg tablets in the morning and two 4-mg tablets in the evening
- LMTM 8-mg/day group: One 4-mg tablet and one placebo tablet in the morning, and one 4-mg tablet and one placebo tablet in the evening
- Placebo group: Two “dummy” tablets in the morning and two “dummy” tablets in the evening, one of which may be replaced by a 4-mg MTC tablet to maintain the treatment blind (the remainder being placebo tablets)

Name of Sponsor / Company: TauRx Therapeutics Ltd (TauRx)
Name of Finished Product: LMTM (TRx0237) Film-coated Tablets, 4 mg
Name of Active Ingredient (Drug Substance): Hydromethylthionine Mesylate

The first dose of study drug (Visit 2) and the morning dose at Visit 3 (after 4 weeks of study drug), Visit 7 (after 52 weeks of study drug), and Visit 10 (after 104 weeks of study drug) will be administered in the clinic. These visits should be scheduled to occur in the morning and subjects will be required to remain at the site for at least 4 hours post-dose on each occasion. Alternative arrangements for dosing and dispensing study drug supplies due to COVID-19 may be permitted and are further described in the body of the protocol.

Interruption of dosing for up to a maximum of 14 consecutive days may be allowed if the investigator determines this is indicated (*e.g.*, due to an AE or any other reported change in the subject's physical condition in the judgment of the investigator) on a maximum of two occasions. Dose reduction is not permitted. The reason for dose interruption should be recorded in the source documentation.

After the completion of the 52-week double-blind treatment period, beginning with supplies dispensed at Visit 7 (Baseline/Day 1 for the open-label phase), all subjects will receive LMTM 16 mg/day (two 4-mg tablets in the morning and two 4-mg tablets in the evening) from a newly dispensed open-label study kit for an additional 52 weeks during the open-label, delayed-start phase. The in-clinic dose of study drug at Visit 7 is to be taken after pre-dose assessments have been completed (refer to Table 10-3 for the pre-dose and post-dose assessments to be performed at Visit 7).

Methodology

All subjects should have a likely diagnosis of probable AD or MCI-AD prior to being offered the consent forms, on the basis of investigator interview and examination. Subjects currently using an AChEI and/or memantine must be willing to discontinue such medication before continuing with screening (for timing of withdrawal reference Table 10-1).

Following provision of written informed consent by the subject (and/or legal representative[s]) and study partner(s), consistent with national and/or local law, eligibility for enrollment will be assessed initially during the Screening period which may require multiple visits (collectively designated Visit 1). These will occur no earlier than 63 days before Visit 2 for subjects not using an AChEI and/or memantine. For subjects using an AChEI and/or memantine at the time of signing the consent document or who have recently discontinued use, Screening visits can occur no earlier than 105 days before Visit 2.

The diagnosis of probable AD or MCI-AD should be confirmed per the 2011 NIA/AA criteria, documented in the subject's medical records and independently confirmed by site diagnosing physician. MMSE and CDR will then be completed at Screening for eligibility assessment. Note that MMSE should be conducted first, followed by the CDR assessment only if the subject meets the MMSE inclusion criteria. If the subject does not qualify with either of these scales, no further assessments should be performed and the subject should be considered a screen failure. If, however, the subject meets the MMSE and CDR inclusion criteria, a blood sample for clinical laboratory testing should be obtained and medical screening assessments performed.

In subjects for whom it is appropriate to proceed with further screening, a PET scan that is positive for amyloid and an MRI scan that excludes other CNS pathology are required at Screening to confirm subject eligibility. The amyloid PET scan can have been documented previously as positive or, alternatively, be based on the local read (or central for those sites not able to read locally) of a PET scan using an approved amyloid ligand and according to criteria recommended as standard by the manufacturer of the ligand. All images (including prior images with subject consent) will be sent to an imaging core laboratory, either as a repository (amyloid PET) or for evaluation by a trained technologist for acceptable quality (MRI). MRIs will be reviewed by an independent neuroradiologist (reader) who is trained in the evaluations and is not involved in the clinical conduct of the study to confirm eligibility of the subject. The reader's MRI assessment will be communicated to the site within 5 business days of image transfer to the imaging core laboratory (or of resolution of any quality issues).

Subjects willing to discontinue AChEI and/or memantine in order to take part in the study must be documented to be eligible according to clinical inclusion / exclusion criteria, screening MRI and amyloid positive PET scan, before these drugs are discontinued.

Only subjects with a CDR 0.5 will undergo ^{18}F-FDG-PET. ^{18}F-DG-PET images are to be obtained prior to Visit 2 (and at least 60 days after the last dose of AChEI and/or memantine) for purposes of establishing the baseline assessment. Completion of this scan must be confirmed prior to randomization; other than confirmation of acceptable quality, the results per se are not considered for eligibility at the Screening visit. The baseline

Name of Sponsor / Company: TauRx Therapeutics Ltd (TauRx)
Name of Finished Product: LMTM (TRx0237) Film-coated Tablets, 4 mg
Name of Active Ingredient (Drug Substance): Hydromethylthionine Mesylate

^{18}F-FDG-PET image will be sent to a core imaging laboratory and will be reviewed by an independent, appropriately trained nuclear medicine reader. Imaging receipts will be sent in all cases; only in those instances where the scan is deemed unacceptable will the site and imaging laboratory be further notified.

At the Baseline/Randomization visit (Visit 2), eligibility will be confirmed, subjects will be randomized, and baseline safety and efficacy assessments will be completed. Subjects will be treated with study drug for 52 weeks on an outpatient basis. During the double-blind, treatment period, on-treatment, post-Baseline study visits will occur at time points approximately 4, 13, 26, 39, and 52 weeks after Baseline. All subjects will receive a supply of study drug according to their randomization, to take home for use until Visit 4 (13 weeks after Baseline). Study drug will be resupplied at Visits 4, 5, and 6 (13, 26, and 39 weeks after Baseline). Visit 7 (the final visit in the double-blind treatment period) will serve as the open-label Baseline. During the open-label, delayed-start phase, study visits will occur at approximately 4 weeks (by telephone), 26 weeks, and 52 weeks after the open-label Baseline (Visits 8, 9, and 10), with a supply of open-label study drug from a newly dispensed study kit provided at Visits 7 and 9. Alternative arrangements for study procedures and assessments in response to COVID-19 are described in the body of the protocol.

The study will be monitored for safety by a Data and Safety Monitoring Board (DSMB) throughout its duration.

Assessments

Efficacy:

Imaging assessments will be made by central readers as follows:

- Brain MRI will be evaluated for temporal, parietal, whole brain, lateral ventricular, hippocampal, putamen, nucleus accumbens, and nucleus basalis volumes at Screening and after 13, 26, 39, and 52 weeks, or upon early termination. Change in MRI volumetric parameters will be quantified at the imaging core laboratory. The image hyperintensities will also be quantified. MRIs will also be obtained and evaluated after the additional 26 and 52 weeks of open-label treatment.
- Brain ^{18}F-FDG-PET will be evaluated in subjects with CDR 0.5 for normalized temporal, parietal, and frontal lobe SUVR at Baseline and after 52 weeks, or upon early termination. Other regions of interest will also be examined including, but not restricted to, inferior temporal gyrus, angular gyrus, anterior and posterior cingulate gyrus, and cerebellum. Change in SUVR parameters will be quantified by the imaging core laboratory.

Two primary clinical efficacy scales will be used, ADAS-cog$_{13}$ and ADCS-ADL$_{23}$. Clinical assessments will be made by suitably trained Efficacy Raters who are not involved in safety assessments, using the instruments on an electronic tablet (and captured via an audio recording of the assessment for independent evaluation). The following will be assessed at Baseline; after 13, 26, 39, and 52 weeks during the double-blind treatment period; and after the additional 26 and 52 weeks of open-label treatment, or upon early termination:

- ADAS-cog$_{13}$
  - As the primary endpoint is ADAS-cog$_{11,}$ in order to be able to link the results of this study to the recently completed Phase 3 studies TRx-237-005 and TRx-237-015, an ADAS-cog$_{11}$ will be derived from the ADAS-cog$_{13}$.
- ADCS-ADL$_{23}$

The MMSE and CDR will be repeated at the final double-blind treatment period visit (Visit 7) and the final open-label visit (Visit 10) or upon early termination.

The clinical efficacy scale assessments should be performed at approximately the same time of day throughout the study for a given subject, to the greatest extent possible.

Safety and Tolerability:

Safety assessments will be performed during Screening to assess subject eligibility for enrollment. All safety assessments will be performed by an independent qualified assessor not involved in efficacy assessments; where specified below, the assessments must be made by a medical assessor (physician, doctor of medicine [MD], or doctor of osteopathic medicine [DO]). For enrolled subjects, safety assessments will be made at Baseline and at each clinic visit (*i.e.*, after 4, 13, 26, 39, and 52 weeks during the double-blind treatment period as well as after 26 and 52 weeks in the open-label, delayed-start phase); during a telephone contact after 4 weeks in the open-label, delayed-start phase; when needed to follow up on an AE; and upon early termination. All AEs, vital signs,

Name of Sponsor / Company: TauRx Therapeutics Ltd (TauRx)
Name of Finished Product: LMTM (TRx0237) Film-coated Tablets, 4 mg
Name of Active Ingredient (Drug Substance): Hydromethylthionine Mesylate

ECG, clinical laboratory findings, physical and neurological examinations, and ophthalmological examinations (as applicable) will be assessed according to the following:

- AEs will be recorded from the time informed consent is signed and recording will continue throughout the study and, if pertinent, until resolution of the event; AEs with an onset after the first dose of study drug or that worsen in intensity or treatment relationship after the first dose will be considered treatment-emergent (and must be reviewed by a medical assessor).
- Blood pressure and pulse will be obtained at Screening, on Day 1 (pre-dose and approximately 2 hours post-dose), and at each clinic visit thereafter or upon early termination. Blood pressure and pulse will be measured after the subject has been in a seated position for at least 5 minutes.
- Height will be measured at Screening only. Body weight will be measured at Screening and at each clinic visit thereafter or upon early termination.
- A 12-lead ECG will be obtained at Screening, with the site assessing eligibility based on the local clinical interpretation by a medical assessor.
- TSH, vitamin B_{12}, folate, haptoglobin, and G6PD will be measured at Screening; a thyroid panel may be obtained in response to an elevated TSH. Standard clinical laboratory testing, including hematology and blood chemistry, will be performed at Screening and each subsequent clinic visit (or upon early termination). Testing may also be performed as needed in response to an AE. All laboratory testing results must be reviewed by a medical assessor.
- A blood sample for a serum pregnancy test will be collected from all women of childbearing potential at Screening and at each subsequent clinic visit (or upon early termination).
- Complete physical and neurological assessments will be performed at Screening. Targeted examinations will be performed pre-dose and approximately 3 hours after administration of the first dose of study drug (Visit 2). Thereafter, targeted examinations are to be performed at each subsequent clinic visit (or upon early termination). At a minimum, targeted examinations should include heart and lung auscultation and brief neurological assessment guided by any reported signs/symptoms/AEs (*e.g.*, evaluating subjects for potential serotonin toxicity). These examinations are to be performed by a medical assessor.
- Ophthalmological examination (slit lamp) of subjects with history of lens implants will be performed by a licensed optometrist, ophthalmologist, or other suitably qualified medical assessor prior to the first dose of study drug (during the Screening procedures or as part of the baseline assessments), at Visit 7, and at Visit 10 / early termination (after completion of efficacy assessments), to assess whether the lens has been discolored during the trial. A slit lamp examination should also be performed if a subject has cataract surgery/lens implantation at any point during his or her study participation (as soon as possible after the surgery), as well as in response to visual complaints if suggestive of lens discoloration.
- At Screening, medications administered within the last 90 days will be recorded; the exception is for anti-dementia medications, where lifetime use (as far as possible) is to be recorded. Changes in concomitant medications and any new medications will be recorded at all subsequent visits, including the telephone contact, and reviewed by a medical assessor.

Other Assessments:

- At Visit 2, Visit 3, Visit 7, and Visit 10, blood will be collected on the same day on three occasions for determination of plasma MT and whole blood concentrations (parent MT/LMT, *N*-desmethyl MT, and total MT, to the extent possible); these visits should be scheduled to occur in the morning. Samples will be collected prior to dosing (in the clinic) and again approximately 1 to 2 hours post-dose; a third sample will be collected approximately 4 hours after the dose. Times of dose and blood sampling (and at Visits 3, 7, and 10, times of the prior dose taken on the preceding day) will be recorded. At any early termination visit, if the subject has not yet discontinued study drug and is willing to take a final in-clinic dose, three blood samples will be collected as described above. If, however, the subject has already discontinued study drug or is unwilling to take a final in-clinic dose, a single blood sample only for determination of MT concentrations will be collected, irrespective of the date or time of the last dose of study drug. The date and time of the last dose will be collected and recorded. For subjects who continue in the study off-treatment (the off-treatment-on-study [TOTOS] group), blood samples will not continue to be collected for the determination of MT concentrations.
- A single blood sample for *ApoE* will be obtained from subjects who provide legally acceptable informed consent; the blood sample may be collected any time after eligibility for randomization and continued participation in the study has been confirmed but prior to Visit 7 (end of the double-blind treatment period).

Name of Sponsor / Company: TauRx Therapeutics Ltd (TauRx)
Name of Finished Product: LMTM (TRx0237) Film-coated Tablets, 4 mg
Name of Active Ingredient (Drug Substance): Hydromethylthionine Mesylate

Statistical Analyses

Double-blind Treatment Period:

The primary target of estimation is the difference in ADAS-cog_{11} and ADCS-ADL_{23} over 52 weeks in subjects receiving LMTM in a dose of 16 mg/day as compared to placebo.

The primary efficacy analyses for FDA will be performed on the Efficacy Modified Intent-to-Treat (E-MITT) which will include all randomized subjects who took at least one dose of study drug and have a baseline and a valid post-baseline efficacy assessment, and for EMA on the Intent-to-Treat (ITT) population with conservative assumptions about disease progression made for subjects who have missing post-baseline assessments. Subjects will be analyzed in the treatment group and according to the stratification variables to which they were randomized.

Changes from baseline to Week 52 on the co-primary efficacy endpoints and other modalities with repeated measures such as MRI will be analyzed using a linear mixed model for repeated measures with unstructured covariance matrix. The linear mixed model will contain fixed effects for time, treatment, and a treatment-by-time interaction; additionally, the baseline value of the corresponding endpoint will be included as a covariate and the randomization stratification variables of prior usage of AChEIs and/or memantine, severity based on MMSE (16-19, 20-25, or 26-27) as determined at Screening, and region will be included as fixed effects in the model. ^{18}F-FDG-PET and other modalities with only one planned post-baseline assessment will be analyzed using an ANCOVA; the covariates for this model will be adjusted accordingly by removing terms with time.

More details about the exact contrast statements used for these tests as well as further sensitivity analyses, additional analyses of the primary variables (such as responder analyses and subgroup analyses), analyses of the secondary and exploratory endpoints, and modifications to be implemented regarding sensitivity analyses due to COVID-19 are described in the body of the protocol and SAP.

Open-Label, Delayed-Start Phase:

"Late" starters, *i.e.*, subjects originally randomized to placebo, will be compared to "early" starters, *i.e.*, those originally randomized to LMTM 16 mg/day or 8 mg/day (pooled, and individually for both groups as a sensitivity analysis). The last assessment prior to start of open-label treatment (Visit 7) will serve as the baseline assessment for these comparisons. The analyses will be based on a similar linear mixed model for repeated measures with the aim to investigate whether there is a difference in change in primary and MRI imaging endpoints dependent on LMTM treatment history. The comparison will be implemented through a non-inferiority test. The non-inferiority margin for the primary endpoint of ADAS-cog_{11} will be 2 units, motivated by the fact that these 2 units are small compared to the estimated treatment effect of > 5 units (~40% of this effect size). Margins for other endpoints which are exploratory to support a disease modifying argument will be defined in the SAP. The results will also be presented with the Visit 2 Baseline as baseline and treatment visits Week 26 and Week 52 in the open-label, delayed-start phase as Week 78 and Week 104.

7 ABBREVIATIONS

Abbreviations	Definitions
AA	Alzheimer’s Association
AChEI	acetylcholinesterase inhibitor
AD	Alzheimer’s Disease
ADAS-cog_{11}	Alzheimer's Disease Assessment Scale – Cognitive Subscale (11-item)
ADAS-cog_{13}	Alzheimer's Disease Assessment Scale – Cognitive Subscale (13-item)
ADCS-ADL_{23}	Alzheimer's Disease Cooperative Study – Activities of Daily Living (23-item)
ADNI	Alzheimer's Disease Neuroimaging Initiative
ADR	Adverse Drug Reaction
AE	adverse event
AESI	adverse event of special interest
ApoE	Apolipoprotein E gene
ATC	Anatomical Therapeutic Classification
AUC	area under the plasma concentration *versus* time curve
BCRP	breast cancer resistance protein
bid	twice daily
BSI	Boundary Shift Integral
BSA	body surface area
bvFTD	behavioral variant frontotemporal dementia
CDR	Clinical Dementia Rating
CJD	Creutzfeldt-Jakob Disease
CL/F	apparent plasma clearance
C_{max}	peak plasma concentration
$C_{max, ss}$	peak steady-state plasma concentration
CMP	Clinical Monitoring Plan
CNS	central nervous system
COVID-19	Coronavirus Disease 2019 Public Health Emergency
CT	computerized tomography
CYP	Cytochrome P450 isoenzyme
DO	Doctor of Osteopathic Medicine
DSMB	Data and Safety Monitoring Board
EAP	Expanded Access Program
ECG	electrocardiogram
eCRF	electronic Case Report Form
EMA	European Medicines Agency
E-MITT	Efficacy Modified Intent-to-Treat
eTMF	electronic Trial Master File
EU	European Union
FDA	Food and Drug Administration (United States)
^{18}F-FDG-PET	^{18}F-fluorodeoxyglucose positron emission tomography
g, kg, mg, pg	gram, kilogram, milligram, picogram
G6PD	glucose-6-phosphate dehydrogenase
GCP	Good Clinical Practice
GI	gastrointestinal

Abbreviations	Definitions
GMP	Good Manufacturing Practice
Hgb	hemoglobin
HTLV-III	human T-cell lymphocyte virus Type III
ICE	intercurrent events
ICF	informed consent form
ICH	International Council for Harmonisation of Technical Requirements for Pharmaceuticals for Human Use
IEC	Independent Ethics Committee
INN	International Nonproprietary Name
IRB	Institutional Review Board
ITT	Intent-To-Treat
IUD	intrauterine device
L, mL, dL	liter, milliliter, deciliter
LAR	Legally Acceptable Representative
LAV	Lymphadenopathy Associated Virus
LDH	lactate dehydrogenase
LMT	hydromethylthionine, leuco-methylthioninium
LMTB	leuco-methylthioninium bis(hydrobromide)
LMTM	hydromethylthionine mesylate, leuco-methylthioninium bis(hydromethanesulfonate)
LSM	Least Squares Mean
LVV	lateral ventricular volume
MAO	monoamine oxidase
MATE	Multidrug And Toxin Extrusion (protein)
MCI-AD	mild cognitive impairment due to AD
MD	Doctor of Medicine
MedDRA	Medical Dictionary for Regulatory Activities
MHRA	Medicines and Healthcare products Regulatory Agency
min, msec	minutes, millisecond
MI-MITT	MRI Imaging Intent-to-Treat
MMSE	Mini-Mental State Examination
MRI	magnetic resonance imaging
MT	Methylthioninium
MTC	methylthioninium chloride
NIA	National Institute on Aging
NMDA	N-methyl-D-aspartate
NOAEL	no-observed-adverse effect-level
nvCJD	new variant Creutzfeldt-Jakob Disease
OAT	Organic Anion Transporter
OCT	Organic Cation Transporter
PET	Positron Emission Tomography
P-gp	P-glycoprotein
PHF	paired helical filament
PI	principal investigator
PI-MITT	Imaging Modified Intent-to-Treat
PK	Pharmacokinetic
PP	Per Protocol

**Abbreviations**	**Definitions**
QA	Quality Assurance
RBC	red blood cell
ROI	region of interest
RTSM	Randomization and Trial Supply Management
SAE	serious adverse event
SAP	Statistical Analysis Plan
SI	Sub-Investigator
SOC	System Organ Class
SOP	Standard Operating Procedure
SPM	statistical parametric mapping
SUSAR	suspected unexpected serious adverse event
SUVR	Standardized Uptake Value Ratio
$T_{½}$	terminal elimination half-life
TEAE	treatment-emergent adverse event
tid	three times daily
TOTOS	The-Off-Treatment-On-Study (subjects)
TSE	Transmissible Spongiform Encephalopathy
TSH	thyroid stimulating hormone
UGT	UDP-glucuronosyltransferase
USAN	United States Abbreviated Name
USP	United States Pharmacopeia
vCJD	variant Creutzfeldt-Jakob Disease
WBC	white blood cell
WHO	World Health Organization

8 BACKGROUND AND RATIONALE FOR THE STUDY

Hydromethylthionine (International Nonproprietary Name [INN] and United States Abbreviated Name [USAN]), the reduced form of methylthioninium (MT) that is also known as leuco-methylthioninium (LMT), is proposed for treatment of tauopathies and other protein misfolding disorders. Tauopathies are a class of neurodegenerative diseases associated with pathological aggregation of tau protein in the human brain. The tauopathies include diseases that primarily affect cognition such as Alzheimer's disease (AD). AD is a severe irreversible neurodegenerative disease resulting in complete loss of mental faculties. In AD, the microtubule associated protein tau is redistributed exponentially into paired helical filaments (PHF) forming neurofibrillary tangles that correlate with pyramidal cell destruction (Wischik *et al.*, 1997). There is a robust clinico-pathological correlation between tau pathology, tau aggregation, and clinical measures of dementia (Bierer *et al.*, 1995; Mukaetova-Ladinska *et al.*, 2000). These relationships are maintained from the earliest detectable stages of dementia and progress in parallel with clinical deterioration and are also seen by imaging using recently developed tau ligands (Lockhart *et al.*, 2016; Xia *et al.*, 2017; Pontecorvo *et al.*, 2017.

The repeat-domain tau fragment originally identified biochemically as a structural constituent of the tangle PHF core (Wischik *et al.*, 1988a,b) has recently been confirmed using cryo-electron microscopy (Fitzpatrick *et al.*, 2017). This fragment assembles spontaneously *in vitro* to form PHF-like filaments (Al-Hilaly *et al.*, 2017). The active moiety required both to block filament assembly and to disassemble formed filaments is the reduced LMT form of MT (Al-Hilaly *et al.*, 2018).

As of 2020, drugs currently available to treat AD, such as acetylcholinesterase inhibitors (AChEIs) or memantine, are symptomatic treatments that address certain central neuronal dysfunctions associated with AD, but are not known to directly affect the neurofibrillary tangles in the brain that represent a core pathological component of AD. Controlled studies with the AChEIs donepezil, rivastigmine, and galantamine have demonstrated small improvements in cognitive tests and global measures of change in selected subjects with mild to moderate AD over 3 to 12 months (Birks and Harvey, 2003; Olin and Schneider, 2002). However, improvements in function and behavior have been demonstrated less reliably with AChEIs. Furthermore, although these medications provide benefits for some subjects, their effectiveness often is limited in duration and they do not affect the rate of progression of the disease (Courtney *et al.*, 2004).

Memantine is a noncompetitive, low-affinity, N-methyl-D-aspartate (NMDA) receptor antagonist that might prevent calcium-mediated glutamate excitatory toxicity in AD. Studies with memantine over 6 months in subjects with moderate to severe AD have shown small benefits on cognition, global measures, daily living activities, and behavior (Reisberg *et al.*, 2003), but evidence for a benefit is lacking in mild AD (Schneider *et al.*, 2011).

Aduhelm™ (aducanumab), an amyloid beta-directed antibody, was recently granted accelerated approval by FDA in June 2021 for early AD based on a biomarker (reduction of amyloid plaque). One particularly significant limitation in the widespread use of such drugs is the requirement for monthly intravenous infusions of the monoclonal antibody preparations that target aggregated forms of amyloid, which has a substantial impact on the costs of production, distribution, and administration of such treatments. Based on the clinical trials to date, there are uncertainties regarding the effectiveness of aducanumab on clinical outcomes.

In addition, there is a significant risk of triggering amyloid-related imaging abnormalities, requiring regular monitoring by magnetic resonance imaging (MRI) scans (summarized in the Aduhelm FDA Drug Approval Package, June 2021).

Therefore, an unmet need exists to develop new medications for AD that more directly modify the underlying disease pathology and offer longer-term and greater efficacy. LMTM, the investigational product, is believed to have the potential to confer benefits over existing treatments for AD due to its ability to affect the process of tau aggregation responsible for the underlying neurofibrillary pathology of AD. Available nonclinical and clinical evidence supports the clinical evaluation of LMTM in AD.

8.1 Background

8.1.1 Investigational Product

The investigational product is LMTM (USAN hydromethylthionine mesylate, also referred to as leuco-methylthioninium bis(hydromethanesulfonate), hydromethylthionine bis(hydromesylate), leucomethylene blue dimesylate, and its code name, TRx0237). As a dihydromethanesulfonate salt (also known as mesylate), LMTM stabilizes the reduced crystalline form of the MT moiety in the solid state.

LMTM is provided as 4-mg, immediate-release tablets, to be taken twice daily; the pharmacokinetics (PK) of LMTM 4 mg as a single dose and given twice daily at steady state (8 mg/day) are described in Section 8.1.3.1. Following dissociation of the counter ions, the uncharged (reduced) LMT form, *i.e.*, the active moiety, is absorbed passively. The charged (MT^+) form does not have the conformation necessary to block filament assembly and to disassemble formed filaments (Al-Hilaly *et al*., 2018). When dosed as the charged (MT^+) form (as in methylthioninium chloride [MTC], also known as methylene blue), it is postulated that MT^+ requires an additional reduction step (to LMT) to be distributed to the brain. Within cells, MT exists in equilibrium between the reduced (LMT) and oxidized (MT^+) forms, the predominant form present depending on the cellular milieu, *i.e*., pH and reductive capacity within that cell, with the LMT form predominating intracellularly (May *et al*. 2004).

Matching placebo tablets are provided. The drug supplies for the placebo group will include tablets containing a urinary discolorant, MTC, 4 mg, in order to provide urinary discoloration and maintain the treatment blind.

8.1.2 Nonclinical Data

In cell models, LMT (including as LMTM) has been shown to prevent aggregation and facilitate disaggregation of pre-existing oligomers and fibers. In transgenic mouse models, facilitation of clearance of tau aggregates and associated improvements in cognitive and motor learning abilities have been demonstrated. Tau pathology in these transgenic mouse models has been shown to be ameliorated in certain brain regions (*e.g.,* entorhinal cortex, hippocampus, and neocortex) following treatment with LMTM.

The primary toxicity of MT is hematological, manifesting as methemoglobinemia and a regenerative hemolytic anemia. Depending on the species, Heinz body formation also occurs. A no-observed-adverse effect-level (NOAEL) of 3 to 5 mg MT/kg/day was established in rats, regardless of salt or duration, depending on the low dose in a given study; the parent

MT/LMT plasma levels (exposure) at these doses are comparable to human exposure at an LMTM dose of 8 mg/day. In monkeys, the NOAEL for hematological toxicity was 0.9 mg MT/kg/day in the 6-month MTC phase (toxicokinetic data at this dose are not available); the NOAEL in the subsequent 9-month LMTB phase in which higher doses were given was <20 mg MT/kg/day (the lowest dose studied). Hematological toxicity was seen in minipigs even at the lowest dose studied; therefore, the NOAEL is considered to be <3 mg MT/kg/day. The hematological effects in rats occur at exposures that are 2.1-fold higher than in humans at an LMTM dose of 16 mg/day and in minipigs at exposures that are 1.2-fold higher. In rats and minipigs (but not monkeys) at the hematologically toxic doses, myeloid generation is also affected, and extramedullary hematopoiesis is seen. Pigment (hemosiderin) deposition becomes evident in liver and renal cortical tubules (some lipofuscin is also present in minipigs) with no adverse effect on either liver or kidney function evident. At relatively high doses, effects on white blood cells (WBCs) are seen, but not consistently across species (seen only in rats, not monkeys or minipigs). Decreases in circulating neutrophils occurred with an exposure-based safety margin of 8.9.

The other potential significant toxicities observed include effects on the heart, described as myocardial necrosis, generally occurring at rapidly lethal doses that exceed the maximum tolerated doses for chronic administration. In the minipig, no cardiac toxicity was observed at the highest dose administered chronically, providing safety margins of >7.5-fold relative to an LMTM dose of 16 mg/day in humans. Gastric irritation is seen in rodents and urinary bladder irritation is seen in mice and minipigs (with single cell necrosis in the latter species).

MT is genotoxic *in vitro* and damages DNA *in vitro,* but is not genotoxic *in vivo.* There is no evidence of an interaction with or damage to DNA *in vivo*, a difference which may be due to differences in the predominant redox state of MT *in vitro* and *in vivo*. In addition, MT^{+}, as a photosensitizer, is activated *in vitro* by visible light to form reactive hydroxyl and superoxide radicals and has been found to be mutagenic *in vitro* only in the presence of light; there is no evidence that MT administered orally undergoes photo-oxidation. According to the International Council for Harmonisation of Technical Requirements for Pharmaceuticals for Human Use (ICH)[1], "When a positive result occurs in an *in vitro* mammalian cell assay, clearly negative results in two well-controlled *in vivo* assays, in appropriate tissues and with demonstrated adequate exposure, are considered sufficient evidence for lack of genotoxic potential *in vivo*." Thus, MT (and by extension its active metabolite *N*-desmethyl MT) is considered to have no clinically relevant genotoxic potential.

As reviewed by the U.S. National Toxicology Program, there is "some evidence of carcinogenic activity"[2] of MTC in male rats based on increased incidences of pancreatic islet cell adenoma and adenoma or carcinoma (combined) and in male mice based on increased incidences of carcinoma and of adenoma or carcinoma (combined) in the small intestine. When data are evaluated using the U.S. Food and Drug Administration (FDA) criteria for a positive response[3], there were no statistically significant neoplastic findings in either species. However, FDA concluded that the combined incidence of pancreatic islet cell adenomas and carcinomas in male rats was higher than in historical controls, particularly at the mid-dose of

[1] ICH S2(R1): Genotoxicity Testing and Data Interpretation for Pharmaceuticals Intended for Human Use (2012)

[2] Text in quotation marks refers to the interpretation of results provided by the U.S. National Toxicology Program, the sponsoring organization. These analyses were not consistent with FDA statistical guidelines.

[3] FDA Guidance for Industry: Statistical aspects of the design, analysis, and interpretation of chronic rodent carcinogenicity studies of pharmaceuticals (draft, May 2001).

19 mg MT/kg/day, and thus caused by MT. At this dose, the exposure-based safety margin relative to a human LMTM dose of 16 mg/day is 3.0-fold.

MT has no effect on mating performance or pregnancy rate in rats when administered orally. The exposure-based safety margin is >8.3-fold and >10.1-fold in males and females, respectively, for an LMTM dose of 16 mg/day.

Two embryo-fetal development studies have been performed by NTP wherein MT was dosed orally during organogenesis. There was no evidence of teratogenicity in rats (NOAEL safety margin of 92-fold relative to a human LMTM dose of 16 mg/day, adjusted for differences in body surface area [BSA]); evaluation of fetotoxicity was confounded by maternal toxicity at all doses. In rabbits, the NOAEL for teratogenicity was the lowest dose studied, a 46-fold safety margin; fetotoxicity (abortions) occurred at the lowest dose (a dose that was maternally toxic). The observed malformations in rabbits, umbilical hernia, are of unclear relevance to humans. Thus, the nonclinical reproductive toxicity studies do not raise a strong suspicion of human teratogenicity/fetotoxicity at clinical doses of LMTM to be studied. Results of studies in other species and by other routes of administration are presented in the Investigator's Brochure. There have been no reports of human teratogenicity in early pregnancy. Thus, MT is considered to have "unlikely human terotogenicity/fetotoxicity in early pregnancy"[4].

In a standard study performed in male Long-Evans pigmented rats, MT does not cause phototoxicity.

Additional details regarding findings from nonclinical studies with various salt forms of MT (LMTM, leuco-methylthioninium bis(hydrobromide) [LMTB], and MTC) are described in the Investigator's Brochure.

8.1.3 Clinical Data

8.1.3.1 Pharmacokinetics

A single- and multiple-dose pharmacokinetic study has been completed in older (≥50 years), healthy, male and female volunteers (TRx-237-036). Following a single 4-mg dose, the mean peak (C_{max}) plasma parent MT/LMT concentration is 0.1 ng/mL, occurring approximately 1 to 1.5 hours post dose. From review of individual concentration *versus* time profiles, split peaks and one or more secondary peaks are evident within the first 12 hours following dosing in the majority of subjects, consistent with a possible biliary recirculation.

Following a single 125-mg dose of LMTM, the presence of food delays absorption by about 1 hour. C_{max} of parent MT/LMT increases, but not to a clinically meaningful extent; there is no difference in area under the plasma concentration *versus* time curve (AUC).

There is evidence of a possible saturation of absorption with increasing doses. With single-dose administration beginning with a dose of 75 mg, plasma concentrations increase proportionally until a dose of 500 mg. At higher doses up to 1000 mg, there is a delay in C_{max} and plateau in AUC.

MT is not highly protein bound: 69.1% to 74.9% in human blood.

[4] Clinical Trials Facilitation Group: Recommendations related to contraception and pregnancy testing in clinical trials (Version 1.1, September 2020)

Hepatic metabolism to *N*-desmethyl MT, an active metabolite, occurs, mediated primarily by cytochrome P450 isoenzyme (CYP) 1A2 and, to a minor extent, by CYP3A4. Plasma levels of this metabolite are approximately 10% to 30% of parent MT/LMT based on C_{max} and AUC following single or multiple doses (however, plasma concentrations could not be reliably measured).

Conjugation to the inactive *N*-glucuronide is the primary route of metabolism of LMTM. The primary UDP-glucuronosyltransferase (UGT) isoform responsible is UGT1A4; UGT1A7 and UGT1A9 may contribute, as well as UGT1A8 and UGT1A10 to a lesser extent. "Total" MT, *i.e.*, predominantly LMT-glucuronide(s), is present in concentrations that are >1000-fold higher than those of parent MT/LMT in plasma, whether based on C_{max} or AUC following a single 4-mg dose or 8 mg/day. This is consistent with extensive first pass metabolism (particularly glucuronidation) and rapid distribution of MT from the central compartment, as MT is absorbed and passed through the intestine and liver to the systemic circulation and deep compartments.

Steady-state plasma concentrations of parent MT/LMT are achieved within 10 days of dosing (the first timepoint at which trough plasma concentrations were measured in TRx-237-036). At an LMTM dose of 8 mg/day (given daily as two divided doses), plasma parent MT/LMT C_{max} is 0.3 ng/mL. Based on C_{max} and AUC, there is no unexpected accumulation with multiple dosing. At steady state, the mean terminal elimination half-life ($T_{½}$) of parent MT/LMT is 35.6 hours; mean $T_{½}$ of total MT is shorter (19.1 hours).

At the higher doses (150 and 250 mg/day dosed to steady state), there is a linear but greater than proportional increase in parent MT/LMT exposure and a less than proportional increase in total MT. As a result, at steady state, total MT-to-parent MT/LMT ratios are 243- to 388-fold for C_{max} and 249- to 291-fold for $AUC_{0-\tau}$ over the dosing interval, suggesting either a saturation in formation of the glucuronide conjugate or proportionately more extensive distribution into deep compartments at high doses.

LMT is not a substrate of either P-glycoprotein (P-gp) or breast cancer resistance protein (BCRP) transporters. MT^+ (but not LMT) is a substrate of the hepatic uptake transporter, organic cation transporter 1 (OCT1); the renal uptake transporter, organic cation transporter 2 (OCT2); and the renal/hepatic efflux transporters, multidrug and toxin extrusion proteins 1 and 2K (MATE1 and MATE2K). Neither LMT nor MT^+ is a substrate of the other transporters tested (organic anion transporters 1 and 3 [OAT1 and OAT3], or organic anion transporting polypeptides 1B1and 1B3 [OATP1B1 and OATP1B3]).

There are no clinically meaningful differences in apparent clearance (CL/F) between men and women (adjusted for differences in body weight) or between Caucasians and non-Caucasians.

In cross study comparisons, the CL/F of parent MT/LMT is lower in older subjects, possibly attributable to renal and/or hepatic impairment of varying degrees. The mean $T_{½}$ of parent MT/LMT after a single oral dose in healthy older subjects is 20.7 hours as compared to approximately 15.8 hours in younger healthy volunteers[5].

[5] Average based on Studies TRx-237-019, TRx-237-021, and TRx-237-024.

Renal clearance of parent MT/LMT in healthy volunteers represents 13% of CL/F. Most of a radiolabeled dose (72%) is recovered in the urine, with 24% as unchanged drug and the remainder as metabolites, primarily LMT-glucuronide (28% of the dose). Apparent clearance of parent MT/LMT and total MT is decreased in subjects with renal impairment; correspondingly, AUC increases and $T_{½}$ is prolonged. AUC for parent MT/LMT increases by 1.5-fold in subjects with mild impairment to 4.8-fold in subjects with severe impairment; in subjects with severe impairment, C_{max} is also affected (1.7-fold increase). $T_{½}$ of parent MT/LMT after a single dose increases with worsening degree of impairment (mean 24.4 hours, 29.3 hours, and 46.2 hours in mildly, moderately, and severely impaired subjects, respectively), relative to a mean $T_{½}$ of 15.7 hours in generally younger healthy controls [and in a cross-study comparison, 21.3 hours in older healthy volunteers]); the longest $T_{½}$ was 81.9 hours in a subject with severe impairment.

Apparent clearance of parent MT/LMT (but not total MT) is decreased in subjects with mild to moderate hepatic impairment; some of the subjects with mild to moderate hepatic impairment also had markedly elevated bilirubin (including due to Gilbert's syndrome) and/or history of cholecystectomy. When compared to control subjects with normal hepatic function, C_{max} was 2.1- to 3.2-fold higher with mild and moderate impairment, respectively; AUC was 1.4- to 2.1-fold higher, respectively. Mean $T_{½}$ was prolonged 54% to 87% relative to a mean $T_{½}$ of 15.2 hours in generally younger healthy controls (and in a cross-study comparison, 21.3 hours in older healthy volunteers as noted above); the longest $T_{½}$ was 50.1 hours in a subject with mild impairment.

Approximately 23% of an orally administered dose is recovered from the feces (primarily as parent MT/LMT). The observation of secondary peaks in plasma concentrations (and the demonstrated excretion into bile of bile duct-cannulated rats) is consistent with biliary recirculation, indicating that the fecal excretion cannot be attributed solely to unabsorbed drug.

Results of a drug-drug interaction study in healthy volunteers given LMTM (225 mg twice daily [bid]) for 3 days or 10 days indicate that MT is a weak inhibitor of CYP3A4, CYP2C8, and CYP2C19. LMTM is also a weak inducer of CYP2B6 and P-gp. These data indicate that MT may also increase exposure to other co-administered drugs that are substrates of CYP3A4, CYP2C8, or CYP2C19, and decrease exposure to substrates of CYP2B6 or P-gp. However, the extent to which this occurs at the lower LMTM doses of 8 or 16 mg/day is not known. LMTM is not an inducer or inhibitor of CYP1A2, CYP2C9, CYP2D6, or CYP2E1.

When evaluated *in vitro*, neither the reduced form (LMT) nor the oxidized form (MT^+) inhibits UGT enzyme activity or the OCT1, OCT2, OAT1, OAT3, OATP1B1, OATP1B3, MATE1, MATE2k, or the bile salt export pump (BSEP) transporters at clinically relevant test concentrations. LMT (but not MT^+) inhibits BCRP at clinically relevant test concentrations.

8.1.3.2 Cardiac Pharmacodynamics

LMTM given in single, supra-therapeutic doses of 250 and 875 mg (62.5- and 218.8-fold higher than an LMTM 4-mg dose administered at one time) does not prolong the QT interval and is not associated with any other abnormalities of cardiac repolarization.

8.1.3.3 Efficacy

In a double-blind, placebo-controlled Phase 2 study (TRx-014-001) of male and female subjects with mild or moderate AD (AChEIs and memantine excluded), MTC was administered orally at doses of 30 mg three times daily (tid), 60 mg tid, and 100 mg tid (total doses of 69 mg/day, 138 mg/day, and 228 mg/day MT base equivalents, respectively). MT 138 mg MT/day appeared to slow the clinical rate of decline on the Alzheimer's Disease Assessment Scale – cognitive subscale (ADAS-cog) and the Mini-Mental State Examination (MMSE) measured over 1 year. These effects and the benefit on the ADAS-cog, Alzheimer's Disease Cooperative Study-Clinical Global Impression of Change, and MMSE scales were already evident in subjects with moderate disease at Baseline within the first 6 months in an analysis of the entire population with severity as an interaction term. The 6-month analysis and methodology were pre-specified as part of the primary analysis. The result in subjects with moderate disease severity at 6 months remained significant after correction for multiple comparisons. Longer term efficacy was confirmed in mixed mild/moderate subjects by *post hoc* analyses at 50 weeks and 102 weeks, and efficacy was also confirmed in analyses of a variety of secondary endpoints (Wischik *et al*., 2015). The lower dose (69 mg MT/day) was below the minimally effective dose. The capsule formulation used limited the absorption of MT at the 228 mg MT/day dose due to a combination of dose-dependent delay in dissolution of the 100-mg MTC capsules used in the study and a formulation-independent limitation in the ability to absorb MT at the highest dose in the presence of food when administered as MTC. When both of these factors were taken into account, the clinically effective dose available for release within 60 minutes in subjects receiving a nominal dose of 228 mg MT/day was equivalent to approximately 69 mg MT/day (Baddeley *et al*., 2015), and the total available dose was equivalent to 109 mg MT/day (Wischik *et al*., 2015).

Evidence of clinical efficacy was supported by neuroimaging in 138 mild and moderate AD subjects imaged at Baseline and at 24 weeks. Despite lack of decline over this time detected by neuropsychological measures such as ADAS-cog in subjects with mild disease at Baseline, statistically significant decline in neuronal function as measured by regional cerebral blood flow was detected using hexamethylpropylamine oxime-single photon emission computed tomography in all predefined lobes in the region-of-interest analysis and was particularly marked in the inferior medial temporal and temporo-parietal regions of the neocortex on the statistical parametric mapping (SPM) analysis, areas characteristically affected by the tau aggregation pathology of AD and the regions primarily affected in the Braak staging system (Braak and Braak, 1991). MTC at a dose of 138 mg MT/day was found to eliminate this decline entirely (Wischik *et al*., 2015). A similar effect was seen in a smaller group of 20 subjects imaged by ^{18}F-fluorodeoxyglucose positron emission tomography (^{18}F-FDG-PET) to measure neuronal function by glucose uptake. The effect on functional neuroimaging markers at 24 weeks in subjects with mild AD was found to be predictive of a clinical response at 50 weeks (Wischik *et al*., 2015). The reduced efficacy of the 100-mg capsule dose (228 mg MT/day) relative to the 60-mg capsule dose (138 mg MT/day) was also confirmed by neuroimaging.

Two Phase 3 studies of LMTM in AD subjects have recently been completed (Gauthier *et al*., 2016; Wilcock *et al*. 2018); final study reports are in preparation. These were a 15-month study in subjects with mild to moderate AD comparing doses of 150 mg/day and 250 mg/day with a control arm of 8 mg/day (TRx-237-015), and an 18-month study in subjects with mild AD comparing a dose of 200 mg/day and the 8-mg/day control (TRx-237-005). AChEIs and/or memantine were used concomitantly by approximately 80% to 85% of the subjects

depending on study. Briefly, there was no statistically significant difference between LMTM in the higher doses of 150 to 250 mg/day in either of the two co-primary clinical efficacy endpoints or lateral ventricular volume (LVV) when compared with the dose of LMTM 8 mg/day, intended as a control. Based on the results of the Phase 2 study, it was assumed that a dose of 8 mg/day of LMTM would be without activity and could thus be used to control for urinary discoloration because of concerns about blinding. Further analyses of results from both studies suggest that LMTM 8 mg/day given as monotherapy may be effective in delaying progression of mild to moderate AD on co-primary clinical efficacy endpoints, the 11-item version of the ADAS-cog (ADAS-cog_{11}) or the Alzheimer's Disease Cooperative Study - Activities of Daily Living scale, 23-item version (ADCS-ADL_{23}), and also on imaging measures of progression of brain atrophy (MRI and ^{18}F-FDG-PET), and that there is no added benefit to using approximately ≥20-fold higher doses than 8 mg/day.

Recent population PK studies suggest that there are concentration-response relationships for cognitive and neuroimaging outcomes at the 8-mg/day dose. This is seen whether LMTM is taken alone or as add-on to symptomatic treatments (Schelter *et al*., 2019). In a within-cohort meta-analysis of both studies, LMTM at a dose of 8 mg/day as monotherapy was found to produce significant deceleration in the annualized rate of whole brain atrophy after 9 months of treatment. The magnitude of the concentration-dependent treatment effects was reduced by approximately half when LMTM was given to subjects who were concurrently using AChEIs and/or memantine. The reduction in treatment effects of LMTM by treatment with an AChEI or memantine has been reproduced in a tau transgenic mouse model, and appears to reflect a generalized homeostatic downregulation that is induced in multiple brain systems to compensate for the activating effects of symptomatic treatments (Riedel *et al*., 2020).

8.1.3.4 Safety

As of 17 December 2020, 2678 subjects have received at least a single dose of MT (as LMTM, MTC, or an earlier reduced salt studied previously). With respect to LMTM, a total of 2283 subjects (337 healthy volunteers, 40 hepatically or renally impaired subjects, 1688 subjects with AD and 218 subjects with bvFTD) have received at least one dose (ranging from 8 to 1000 mg/day) in completed TauRx-sponsored studies. A total of 321 subjects with AD participated in the 2-year Phase 2 study, TRx-014-001, of whom 307 were exposed to at least one dose of MTC.

The most common adverse events (AEs) associated with MT are anemia and gastrointestinal (GI), genitourinary, and central nervous system (CNS) effects; these are also the most common reasons for discontinuing treatment. These were seen in both the Phase 2 study performed with MTC (given in MT doses up to 228 mg/day) as well as in the Phase 3 studies with LMTM given in MT doses up to 250 mg/day. When all treatment-emergent adverse events (TEAEs) reflective of anemia are grouped, the overall incidence is 13% in subjects randomized to LMTM 8 mg/day. The GI and genitourinary TEAEs are less common at LMTM 8 mg/day than at higher doses. Overall, GI events occurred in 21% of subjects randomized to LMTM 8 mg/day; diarrhea and nausea were amongst the most common individual treatment-related TEAEs, occurring in 7% and 2% of subjects, respectively. The most common urinary TEAEs were frequency, urgency, and incontinence, each treatment related in 1 to 2%. Other less likely TEAEs were agitation, dizziness, fatigue, headache (each with an incidence of 2%), and confusional state (with an incidence of 1%).

Additional details regarding clinical findings from studies are described in the Investigator's Brochure.

8.2 Rationale

The two recently completed Phase 3 studies conducted in subjects with mild to moderate AD (TRx-237-015) and mild AD (TRx-237-005) were designed on the basis of results from the earlier Phase 2 study of MTC as monotherapy in mild to moderate AD. In that study, the minimum dose required for benefit on clinical and imaging endpoints was found to be 138 mg/day. Both of the Phase 3 studies were therefore designed on the assumption that a dose of 8 mg/day of LMTM could be used to control for urinary discoloration because of concerns about blinding. As noted earlier, results from both Phase 3 studies in subjects with mild to moderate AD suggest that LMTM 8 mg/day given as monotherapy may be effective in delaying progression of AD and that there is no added benefit to using doses of 150 to 250 mg/day.

The results of the first of the studies to become available (TRx-237-015) showed that there was no statistically significant difference between LMTM in doses of 150 mg/day or 250 mg/day in either of the two co-primary clinical efficacy endpoints or LVV when compared with the dose of LMTM 8 mg/day intended as a control. However, of the pre-specified terms in the analysis model used in the primary analysis, use of approved AD co-medications (AChEIs and/or memantine) at Baseline was a highly significant term. Further pre-specified analyses in which monotherapy and add-on subjects in the higher dose treatment arms were compared separately with the control group as randomized suggested that LMTM may be effective when given as monotherapy, but not when taken as add-on to approved treatments for AD. LMTM 8 mg/day as monotherapy was also found to differ significantly from 8 mg/day as add-on to existing AD treatments. In light of these results from TRx-237-015, the Statistical Analysis Plan (SAP) of TRx-237-005 was modified prior to database lock and unblinding to specify as primary two similar parallel analyses, each at a statistical threshold of 0.025, to examine 200 mg/day as monotherapy compared with the 8 mg/day control group as randomly assigned, and also 8 mg/day as monotherapy compared with 8 mg/day as add-on to approved AD treatments. Both of these sets of comparisons were statistically significant at the 0.025 threshold for both of the co-primary clinical efficacy endpoints, as well as LVV. No difference in baseline characteristics has been identified in either of the two Phase 3 studies that accounts for the discrepant responses between subjects taking and not taking approved AD treatments, nor is a pharmacokinetic basis for an interaction evident. As indicated above, a within-cohort meta-analysis of both studies showed that LMTM at a dose of 8 mg/day as monotherapy was associated with significant deceleration in the annualized rate of whole brain atrophy after 9 months of treatment.

In the continued development of LMTM, the efficacy of LMTM is to be confirmed in a placebo-controlled study at a dose of 16 mg/day. A population PK model was developed based on Study TRx-237-036 and applied to plasma concentrations from the Phase 3 studies. Using per-patient peak steady-state plasma concentration ($C_{max,ss}$) estimates, concentration-response analyses for ADAS-cog_{11} decline over 65 weeks were performed and a $C_{max,ss}$ threshold of 0.373 ng/mL was identified. The clearance of parent MT/LMT is most significantly associated with renal function, suggesting that the most important predictor of whether or not a subject achieves a parent MT/LMT $C_{max,ss}$ above the threshold is creatinine clearance. At a dose of 8 mg/day, approximately 60% of subjects with normal renal function

would be expected to have plasma concentrations above the threshold, whereas at a dose of 16 mg/day, all subjects would.

The co-primary endpoints are ADAS-cog_{11} and ADCS-ADL_{23}, consistent with trials in subjects with mild to moderate AD. Based on the prior studies, a 12-month duration is deemed sufficient to demonstrate superiority to placebo. To address concerns regarding inadvertent unblinding due to urinary discoloration, subjects randomized to placebo may receive a 4-mg MTC tablet as one of the four tablets to be taken daily. As subjects with early AD (also referred to as prodromal AD/mild cognitive impairment due to AD [MCI-AD]) are also included in the study, ^{18}F-FDG-PET and clinical composite scales are included as sensitive endpoints for this subset of subjects.

An open-label, “delayed-start” phase is also included to demonstrate a disease-modifying drug effect. During the delayed-start phase, placebo subjects are switched to receive the active treatment and thus become delayed-start subjects or “late” starters. Active-treatment subjects continue to receive active treatment (all at a dose of 16 mg/day) during the delayed-start phase and are labeled as “early” starters. The rationale of a delayed-start design is that under the null hypothesis, when the active drug has a purely symptomatic effect and has no effect on neuropathologic process, a delay in administration should have no lasting effect on subjects. An effect that slows the progression of disease by modifying the underlying biological pathology, rather than only attenuating symptoms, would be evident if late starters fail to “catch up” to early starters. Demonstrating a disease-modifying effect would imply a sustained benefit of starting such drugs early. Throughout (that is, both the placebo-controlled and delayed-start phases), all subjects and study personnel are blinded to each subject’s randomization to the early-start or late-start treatment group.

9 OBJECTIVES

The primary objectives of the study pertain to the randomized, double-blind treatment period. The secondary objectives are presented separately for the double-blind treatment period and the open-label, delayed-start phase. Further exploratory analyses and exploratory analyses compared to external data are described in Section 17.5.

9.1 Primary Objectives

1. To compare the LMTM dose of 16 mg/day with the placebo group on the following co-primary endpoints:
   a. ADAS-cog_{11}
   b. ADCS-ADL_{23}
2. To assess the safety and tolerability of LMTM 16 mg/day given for up to 52 weeks

9.2 Secondary Objectives

9.2.1 Double-Blind Treatment Period

3. To compare the LMTM dose of 16 mg/day with the placebo group in annualized rate of whole brain atrophy over 52 weeks as measured by brain magnetic resonance imaging (MRI) and quantified using the Boundary Shift Integral (BSI)

4. To compare the LMTM dose of 16 mg/day with the placebo group in temporal lobe ^{18}F-FDG-PET change in Standardized Uptake Value Ratio (SUVR) (normalized to pons) over 52 weeks, restricted to subjects with Clinical Dementia Rating (CDR) 0.5 at Screening, if a predefined threshold is reached for a sufficient number of subjects providing data
5. To compare the LMTM dose of 8 mg/day with the placebo group in temporal lobe ^{18}F-FDG-PET change in SUVR (normalized to pons) over 52 weeks, restricted to subjects with CDR 0.5 at Screening, if a predefined threshold is reached for a sufficient number of subjects providing data
6. To compare the LMTM dose of 8 mg/day with the placebo group on the co-primary endpoints (ADAS-cog_{11} and ADCS-ADL_{23})
7. To compare the LMTM doses of 8 and 16 mg/day with the placebo group in annualized rate of temporal and parietal lobe atrophy over 52 weeks as measured by MRI and quantified using the BSI
8. To assess the safety and tolerability of LMTM 8 mg/day given for up to 52 weeks

9.2.2 Open-Label, Delayed-Start Phase

9. To determine if there is a difference in disease progression on the co-primary clinical endpoints and the MRI imaging endpoint for subjects who started treatment in the double-blind treatment phase and those who started treatment in the open-label, delayed-start phase (referred to as "early" and "late" LMTM starters, respectively)
    a. Only ADAS-cog_{11} will serve as a secondary endpoint; ADCS-ADL_{23} and other imaging endpoints are exploratory with the aim to be directionally supportive
10. To assess the safety and tolerability of LMTM given for up to 104 weeks

Population PK analyses will be performed to estimate PK exposure in each subject for use in the evaluation of exposure-response relationships. These will be defined in a separate Population PK SAP or a dedicated section of the SAP.

Additional analyses including exploratory and sensitivity analyses are described in Section 17 and further detailed in the SAP.

10 STUDY DESIGN

10.1 General Description

This is a two-phase outpatient study of LMTM administered as monotherapy in subjects with early to mild-moderate AD: a randomized, double-blind, placebo-controlled, 52-week treatment period followed by a 52-week open-label, delayed-start phase. In the latter, all subjects are to continue with open-label treatment, such that subjects originally randomized to placebo are "late" starters of LMTM and subjects originally randomized to LMTM 8 mg/day or 16 mg/day are "early" starters. The total duration of participation for an individual subject will be up to 120 weeks, including a Screening period of up to 16 weeks. Subjects are to have pathophysiological changes as confirmed by positive amyloid PET and severity determined by a Mini-Mental State Examination (MMSE) score of 16-27 / Global Clinical Dementia Rating (CDR) score of 0.5 to 2 at Screening. Subjects must not have used an AChEI and/or memantine within the 60 days prior to the baseline assessments (inclusive

of the baseline ^{18}F-FDG-PET scan in subjects who have a screening CDR of 0.5). The primary treatment group comparisons are of LMTM 16 mg/day and placebo; secondary comparisons will be done for subjects receiving LMTM 8 mg/day *versus* placebo. Following completion of the 52-week treatment period, subjects will continue open-label treatment with LMTM 16 mg/day for a further 52 weeks; prior treatment assignment will not be unblinded. (However, upon completion of the initial 52-week, double-blind, placebo-controlled treatment period, the database will be locked and unblinded for analysis; individual subject prior treatment assignment will not be divulged to subjects or individuals involved in the operational conduct of the ongoing open-label, treatment phase). A description of the study is provided below; see Section 10.2 for a summary of the alternate arrangements that may be implemented due to the Coronavirus Disease 2019 Public Health Emergency (hereafter referred to as COVID-19).

Following provision of written informed consent by the subject (and/or LAR, consistent with national and/or local law) and study partner(s), eligibility will be assessed initially during the Screening period which may require multiple visits (collectively designated as Visit 1).

A sufficient number of subjects will be recruited from sites in North America (United States and Canada) and Europe such that approximately 500 subjects are enrolled. Beginning with Protocol Version 5.0, approximately 450 are to be enrolled and randomized in a 4:1:4 ratio (at the study level) to the LMTM 16-mg/day (200 subjects), LMTM 8-mg/day (50 subjects), and placebo (200 subjects) groups. The randomization will be stratified by severity (three levels: MMSE 16-19, 20-25, or 26-27, with a target ratio of approximately 2:3:1 for those randomized to Protocol Version 5.0 and above), prior use of AChEIs and/or memantine (two levels: prior use of either any AChEIs and/or memantine or none), and region (two levels: North America or Europe).

Eligibility will be confirmed during the Screening period, with the duration of screening depending on whether or not the subject enters the study using an approved AD medication, described as follows (and illustrated in Table 10-1):

- The Screening period will be up to 9 weeks for subjects who are not receiving an AChEI and/or memantine at the time of signing the Informed Consent (Initial Screening Visit, Visit 1).
- The Screening period will be extended for up to a further 6 weeks, 15 weeks in total (+7 days at Sponsor discretion), for subjects who are receiving an AChEI and/or memantine at the time of signing the Informed Consent to allow for the performance of necessary screening tests to confirm eligibility prior to the discontinuation of AChEI and/or memantine and to permit a washout of at least 60 days from the last dose prior to the baseline assessments (inclusive of the baseline ^{18}F-FDG-PET scan in subjects who have a screening CDR of 0.5).
- The Screening visit window may be further extended at the discretion of the Sponsor when justification is provided.

For eligible subjects, one Baseline/Randomization (Visit 2: imaging, efficacy, and safety), and five post-Baseline double-blind visits (Visit 3: safety, and Visits 4, 5, 6, and 7: efficacy, imaging, and safety), and three open-label treatment visits (Visits 8 [telephone contact only], 9, and 10) are scheduled. Unscheduled visits may occur as needed for assessment, or upon early termination. In addition, subjects are to be followed as needed to monitor for the resolution or acceptable stabilization of AEs (including after the last dose), consistent with the investigator’s medical judgement. For women of childbearing potential this includes a

return visit to the clinic for pregnancy testing within 3 months of last exposure to study drug in the event of delayed menstruation.

Visit 2 (Baseline/Randomization) must be scheduled to occur in the morning when the first dose of study drug will be administered in the clinic. Visit 3 (after 4 weeks of study drug), Visit 7 (after 52 weeks of study drug), and Visit 10 (after 104 weeks of study drug) should also be scheduled to occur in the morning; subjects are to be instructed not to take the morning dose of study drug as it will be administered in the clinic. Subjects will be required to remain at the clinic for at least 4 hours post-dose on each occasion to allow for safety assessments and PK blood sample collection.

Cognitive and functional assessments using two standard scales, ADAS-cog_{13} and ACDS-ADL_{23}, will be performed at Baseline, approximately every 3 months during the double-blind treatment period (Visits 4, 5, 6, and 7), and after the additional 26 and 52 weeks of open-label treatment (Visits 9 and 10), or upon early termination. These will be captured electronically on tablets and subject to independent review. The MMSE and CDR will be repeated after 52 weeks of double-blind treatment (Visit 7) and at the final open-label visit (Visit 10) or upon early termination. To the greatest extent practicable, a consistent rater(s) for a given subject is to be maintained throughout the study.

The MRI obtained during Screening will also be the basis for the baseline volumetric MRI; volumetric MRI will be repeated approximately every 13 weeks (Visits 4, 5, 6, and 7). The imaging endpoints will be quantified by independent core laboratories. MRI will also be done after the additional 26 and 52 weeks of open-label treatment (Visits 9 and 10).^{18}F-FDG-PET imaging will be performed at Baseline and Visit 7 (after 52 weeks), or upon early termination, only in subjects with CDR 0.5.

Safety assessments (AE and concomitant medication review, vital sign review, targeted physical and neurological examinations, and review of clinical laboratory results) will be performed by an independent qualified assessor not involved in efficacy assessments; where specified, the assessments must be made by a medical assessor (physician, doctor of medicine [MD], or doctor of osteopathic medicine [DO]). These will be performed at each clinic visit during the double-blind treatment period (Visits 2 to 7) and after 26 and 52 weeks of open-label treatment (Visits 9 and 10), and when needed to follow up on an AE. Selected safety assessments will also be performed during a telephone contact after 4 weeks in the open-label, delayed-start phase (Visit 8). An ophthalmological examination (only to be conducted in subjects with lens implants) will be performed prior to the first dose of study drug (during the screening procedures or as part of the baseline assessments) and at the final visits in each of the study phases (Visits 7 and 10), or upon early termination, to assess for potential discoloration of the lens. See Table 10-2, Table 10-3 and Section 15.4 for more details on these assessments and the qualifications required to perform them.

Other assessments include collection of blood for plasma and whole blood MT concentration determination and (optionally) collection of a blood sample for Apolipoprotein E (*ApoE*) genotyping.

10.2 Changes Implemented Due to COVID-19

Based on ongoing risk assessment regarding the impact of COVID-19 on this study as described in Section 21.2, selected changes to the performance of study conduct and

monitoring are to be implemented as deemed necessary to protect subjects due to COVID-19 as described in Sections 10.2.1 and 10.2.2, respectively. For subjects already enrolled under earlier versions of the protocol (prior to Protocol Version 6.0), subjects will be re-consented at their next in-clinic visit; in the interim, verbal consent will be obtained as needed for changes to study procedures. Changes to the statistical analyses, including sensitivity analyses to address any impact of COVID-19 on study outcomes, are described in Section 10.2.3.

10.2.1 Changes to Study Conduct

If a subject is undergoing screening assessments, but cannot have protocol-required assessments performed due to COVID-19, the subject should be put on screening pause until such time as COVID-19 impacts have ceased and abstention from treatment with an AChEI and/or memantine should be retained. Screening MMSE/CDR/DV, brain scans, and G6PD blood sample collection do not require repetition if already confirmed. The approach for individual assessments is described further below. Re-screening is addressed in Section 11.3.

Both the Screening visit (including informed consent and all screening assessments) and the Baseline/Randomization visit must continue to occur in the clinic at the study site. The first dose of study drug (Visit 2) will be administered in the clinic. This visit should be scheduled to occur in the morning and subjects will be required to remain at the site for at least 4 hours post-dose.

The intervening post-Baseline visits, *i.e.*, Visits 3, 4, 5, 6 and 7 (after 4, 13, 26, 29, and 52 weeks of double-blind study drug), for clinical safety assessments (adverse event and concomitant medication recording/review), safety and PK blood sample collection (inclusive of the optional genotyping blood sample collected any time after Baseline but prior to Visit 7), as well as efficacy assessments, may be completed in-clinic at the site, in the subject's home by visiting study personnel, or at other safe, suitable alternative location, if deemed necessary to protect a subject due to COVID-19. Similarly, Visits 9 and 10 (after 26 and 52 weeks of open-label treatment) may also be completed in the clinic at the site, in the subject's home by visiting study personnel, or at other safe, suitable alternative location. Efficacy scales may also be performed remotely, if approved by the Sponsor in advance on a case-by-case basis, as further described below. The principal investigators (PIs) retain unilateral decision-making and discretion regarding ongoing subject participation dependent on local circumstance and authority recommendations. Travel to the investigational site is the subject's decision. The following procedures are also to be implemented as necessary:

- Consideration may be given on an individual site basis to expand the allowable time windows for study visits if a site closure is anticipated to be short-term.
- Alternative arrangements for dispensing study drug supplies (*e.g.*, home delivery that would not raise new safety risks) may be permitted if deemed necessary to protect subjects due to COVID-19. If a trial site is not able to deliver study drug to subjects, the study drug may, as an exception, be shipped directly to the subjects by a distributor independent from and acting on behalf of the Sponsor, in line with national law or temporary emergency measures. Requirements for obtaining subject consent consistent with local and national requirements, maintaining the specified study drug storage conditions and handling, accountability, and compliance monitoring will be addressed and documented. In the event that a given subject cannot attend a visit at the study site, the study personnel will make reasonable attempts to assess study drug

compliance with the subject or study partner(s) and document the assessment in the source until it can be verified.

- The ADAS-cog$_{13}$ may be performed in the clinic at the site or, if deemed necessary to protect subjects due to COVID-19, in the subject's home or at other safe, suitable alternatives, in person or remotely via videoconference. For remote administrations to avoid loss of data, MedAvante ProPhase will provide clinical sites with electronic tablets provisioned with Zoom (or equivalent) videoconferencing software only[6]. The electronic tablets will be shipped by the clinical sites directly to subjects, and guidance will be provided to subjects and study partners regarding the use of the electronic tablets for remote scale administration as well as instructions for returning the electronic tablets to the sites. When assessments are performed remotely, the rater will confirm the identity of the subject and study partner before proceeding. The approved site raters will administer the scales through the videoconferencing software and will document the assessment as they would if performing the assessment in person; source data and audio recordings will still be captured via the Virgil system.
    - In the remote administration of the ADAS-cog$_{13}$ (if applicable at that visit), the standard (*i.e.*, validated) mode of administration has been adapted to enable data to be captured by videoconference, with the exception of Commands (only partially supported, and thus a total score cannot be calculated for this subset), Ideational Praxis, and Number Cancellation. Due to these missing data (not all subtests can be administered remotely), total scores will not be calculated directly for the remote administration of the ADAS-cog$_{13}$ but a derived upscaled score will be used for analysis as defined in the SAP.
- The MMSE, CDR, and ADCS-ADL$_{23}$ may be performed either in the clinic at the site or, if deemed necessary to protect subjects due to COVID-19, in the subject's home or at other safe, suitable alternatives in person or remotely by speakerphone. If assessments are performed remotely, the rater will confirm the identity of the subject and study partner before proceeding. The approved site raters will document the assessment as they would if performing the assessment in person; source data and audio recordings will still be captured via the Virgil system.
    - In the remote administration of the MMSE (if applicable at that visit), the approved site rater will administer as much of the MMSE as possible. For those elements that cannot be administered remotely, the rater will confirm that these were omitted before submitting the data to MedAvante-ProPhase per usual practice. Due to missing data, total MMSE scores for remote assessments will not be directly calculated but upscaled as per the SAP.
    - In the remote administration of an ADCS-ADL$_{23}$ or CDR (if applicable at that visit), all items will be scored.
- All MRI scans are to be performed at approved MRI centers using approved scanners; no alternative scanners will be accepted and there is to be no change in scanner for a given subject over the course of the study.
    - Guidance will be provided for scheduled MRI visits at approved imaging centers that may be closed due to COVID-19, including acceptable windows outside the scheduled visit window during which MRI scans can still be performed at approved imaging centers and would be accepted for analysis.

---

[6] While remote administration of the ADAS-cog$_{13}$, MMSE, CDR, and ADCS-ADL$_{23}$ has not been validated, MedAvante ProPhase will prepare written instructions for the rater that detail how to alter administration of each of the scales in order to minimize any impact on the data collected, and to minimize loss of data.

- In subjects with CDR 0.5 at Screening, ^{18}F-FDG-PET imaging will be performed at Baseline (unless the Sponsor approves subject randomization without a brain ^{18}F-FDG-PET scan, if the scan cannot be performed due to COVID-19). All ^{18}F-FDG-PET scans are to be performed at approved PET centers using approved scanners; no alternative scanners will be accepted and there is to be no change in scanner for a given subject over the course of the study.
    - If a Baseline ^{18}F-FDG-PET scan cannot be done due to lack of imaging facility, a follow-up ^{18}F-FDG-PET scan will not be required
- Blood samples for safety laboratory assessments and PK as well as optional genotyping may be collected from subjects in the clinic at the site, in the subject's home, or at other safe, suitable alternative location, using the central laboratory (Labcorp) kits for sample collection; samples are to be shipped to Labcorp for analysis using a designated courier service, and the shipment parameters as per the laboratory manual must be adhered to during transfer (*e.g*., between the subject's home, primary care provider, or clinical site).
    - If blood samples for safety laboratory assessments cannot be shipped to Labcorp, sample analysis for hematology testing will be performed at a local laboratory. As the first post-Baseline (Visit 3) hematology sample analysis is key in monitoring the adverse event of special interest (AESI) of hemolytic anemia, clinical sites will not be permitted to randomize a subject if the Visit 3 blood collection plan is not in place. Blood chemistry and genotyping samples can also be analyzed at a local laboratory or may be stored at -20 °C at the clinical site until shipment to the central laboratory.
    - If blood samples for PK laboratory assessments cannot be shipped to Labcorp, plasma PK samples should be stored at -20 °C at the clinical site; whole blood PK data may be compromised as the samples are stable for a limited period. The University of Aberdeen GLP Test Facility must analyze the PK whole blood sample within 22 days after the sample is taken to confirm it is acceptable for analysis. Results are to be entered into the eCRF by the site.
    - For serum pregnancy testing performed in women of childbearing potential, such women should be encouraged to return to the clinic or request serum pregnancy testing (to be performed at the subject's home or at other suitable, safe alternatives) in the event of a delayed menstrual period to rule out possible pregnancy, during study conduct and for up to 3 months after the last dose of study drug. Site follow up on such subjects should be recorded in the source documentation.
    - For PK blood sample collection at Visit 2, Visit 3, Visit 7, and Visit 10, the third sample to be collected approximately 4 hours after the dose is not required if the visit occurs in the subject's home (although timing of previous dose should be ascertained as accurately as possible).
- Multiple study partners participating only as caregivers will be permitted for a given subject, either simultaneously or as replacements for previous study partners, with no specified maximum. For study partners participating as informants providing data from assessments of the subject, a maximum total of two study partners (*i.e.*, one replacement) would normally be permitted for a given subject; however, up to a total of four study partners (*i.e*., three replacements) are permitted if circumstances change due to COVID-19 and provided they have sufficient contact with the subject to act as a reliable informant. Information obtained from study partners participating as informants will be appropriately identified to distinguish each informant in the event that a given subject has more than one study partner providing data for analysis.

10.2.2 Changes to Study Monitoring

As part of ongoing risk assessment regarding the impact of COVID-19, the Sponsor has assessed the risk associated with the collection of post-baseline data for this study. All study subjects should continue to attend clinic visits as defined in the protocol as long as it is safe and feasible to do so. However, in the event that subjects and/or study partners are unable to attend post-Baseline visits at the clinic in person, guidance for continued data collection to be completed in the subject's home or at other safe, suitable alternatives is described in Section 10.2.1. Any impact of COVID-19 on subject participation in the study will be recorded in the eCRFs.

Due to the unpredictable nature of COVID-19, remote monitoring may be conducted *in lieu* of onsite monitoring, including the targeting of critical data and source (for example to include consent, eligibility, subject safety and drug accountability) for periods where a source data verification backlog exists due to inaccessibility of the site to the clinical monitor. Any change to monitoring of the study as a direct result of COVID-19, such as remote monitoring policies and procedures, will be captured through an addendum/amendment to the Clinical Monitoring Plan (CMP). Every effort will be made to complete the same monitoring tasks remotely as would be conducted onsite while accommodating site-specific policies and limitations. Every effort will also be made to ensure the continued protection of subject safety, subject rights, and data integrity. Any deviations from the CMP will be clearly documented.

There is the possibility of increased amounts of missing data and protocol deviations due to limitations on protocol implementation imposed by COVID-19. Clinical sites are expected to maintain documentation of missing data and protocol deviations in source documents with data entered accordingly in the eCRFs.

As the study continues, there will be a need to address other new and challenging scenarios in real time as they arise at different clinical sites during COVID-19. The Sponsor will continue to assess any limitations imposed on protocol implementation that may pose safety risks to study subjects, and whether it is feasible to mitigate these risks by amending study processes and/or procedures such that subject safety, trial integrity, and compliance with regulations are assured.

10.2.3 Changes to Statistical Analyses

Changes to the Statistical Analyses will be outlined in the SAP and will follow the most recent regulatory guidelines. This section describes briefly the general approach followed and addresses particular challenges related to this study. A detailed list of analyses will be provided in the SAP including the conditions that need to be met in order to trigger these analyses. At a minimum, the number of missing or likely impacted assessments and data points will be provided.

Sites projected to recruit more than 10% of the subjects will be closely monitored to ensure that the MMSE balance is met as closely as possible. It is acknowledged that COVID-19 will have an impact on certain MMSE groups as they are more likely to be asked to take social distancing precautions; corresponding sensitivity analyses will be implemented to assess the impact as briefly described below and summarized further in the SAP.

As COVID-19 can impact specific assessments and lead to missing data due to missed visits, various sensitivity analyses will be provided assessing the impact of COVID-19 on the results. Dependent on the actual number of assessments impacted by COVID-19, a more or less exhaustive set of analyses will be provided taking account of regulatory guidelines and as specified in the SAP.

Among these additional analyses, some will focus on the impact on primary endpoints. If more than 5% of all the assessments (over all subjects and visits) of a given primary endpoint are impacted by COVID-19 (based on the eCRF Covid-19 Impact Assessment), the assessments impacted by COVID-19 will be excluded and upscaled when the respective primary endpoint is analyzed.

Other sensitivity analyses include:

- Subgroup analysis by the way of endpoint ascertainment (in-clinic, remote). A subject is assigned to the subgroup with the most visits done with respect to in-clinic or remotely (excluding Baseline and Week 52, which must be performed in-clinic).
- ADAS-cog$_{11}$: Include only the subset of items (for all visits), which can be administered remotely by telephone or video call.
- ADCS-ADL$_{23}$: Exclude items which are affected by COVID-19 restrictions.
- A composite scale designed as a COVID-19 impact free joint score ("COVID-19 Composite Scale") will be analyzed. It will include items, selected from ADAS-Cog$_{11}$ and ADCS-ADL$_{23}$, that are not expected to be impacted by COVID-19.

Details and further analysis will be specified in the SAP.

If a significant number of assessments is impacted by COVID-19, a sample size increase may be applied. The conditions for this to happen will be outlined and specified before any analyses/assessments are conducted.

As indicated above, a more exhaustive list will be provided accounting for regulatory guidelines in the SAP.

10.3 Study Population

To be eligible for randomization, subjects are to have AD based on a diagnosis of probable AD or MCI-AD according to the criteria of the 2011 National Institute on Aging [NIA] / Alzheimer's Association [AA] with documented pathological changes as confirmed by positive amyloid PET (McKhann *et al.*, 2011; Albert *et al.*, 2011). The allowable severity will be a MMSE score of 16 to 27 (inclusive) and Global CDR score of 0.5 to 2 (if 0.5, including a score of >0 in one of the functional domains). Inclusion and exclusion criteria are given in Section 11.

The study will be conducted in approximately 150 study sites in North America and Europe.

Approximately 2000 subjects may be screened to have approximately 500 subjects who will be enrolled, of whom approximately 450 are to be enrolled and randomized under Protocol Version 5.0 and above. Randomization will be such that subjects will be assigned to the MMSE severity groups at study level based on screening MMSE score with a target ratio of approximately 2:3:1 (MMSE 16-19, MMSE 20-25, MMSE of 26-27, respectively) for those

randomized to Protocol Version 5.0 and above. To achieve this target, enrollment will be monitored and controlled at the site level for high recruiting sites (*i.e*., sites projected to recruit more than 10% of the subjects) as per Section 10.2.3 and capped as needed at the study level *via* the Randomization and Trial Supply Management (RTSM) system. Subjects who drop out after randomization will not be replaced; subjects will be encouraged to continue with study visits until the scheduled completion of the double-blind treatment period (Visit 7). Only subjects who continue in the study and receive LMTM until and including the last open-label visit (Visit 10) may be eligible for a separate EAP.

10.4 Duration

The total duration of participation for an individual subject will be up to 120 weeks, including a Screening period of up to 16 weeks (112 days, with further extension permitted at the discretion of the Sponsor when justification is provided), a double-blind treatment period of 52 weeks, and a further open-label, delayed-start phase of 52 weeks. It is anticipated that the study will have an overall duration of at least 40 months, depending on recruitment rate. The current study will be concluded after the last visit for the last subject under this protocol.

10.5 Schedule of Assessments

10.5.1 Screening Assessments

All subjects should have a likely diagnosis of probable AD or MCI-AD prior to being offered the consent forms on the basis of investigator interview and examination. Subjects for whom legally acceptable informed consent has been obtained will be screened at trial sites for determination of eligibility to enter the study on the basis of further diagnostic evaluations, cognitive assessments, and safety assessments (clinical laboratory testing, vital signs, complete physical and neurological examinations, and a local 12-lead electrocardiogram [ECG]). An overview of the screening assessments is provided in Table 10-1 below; assessments must be performed in the sequence presented. Where required and indicated, eligibility should be confirmed prior to progressing to the next screening assessment. ^{18}F-FDG-PET, to be evaluated only in subjects with CDR 0.5 at Screening, is not an eligibility assessment; however, a valid baseline scan is required prior to randomization, hence it is included on this table. See Section 10.2 for a summary of alternate arrangements that may be implemented due to COVID-19.

Table 10-1: Schedule of Screening Assessments (Over Multiple Visits, Collectively Designated Visit 1)

<table>
<tr><th rowspan="2">Required Sequence of Assessments</th><th colspan="2">Time Frame</th></tr>
<tr><th>On AChMem at Start of Screening</th><th>Not on AChMem at Start of Screening</th></tr>
<tr><td>1. Likely diagnosis of probable AD or MCI-AD (M)</td><td rowspan="14">9 to 16 weeks before Baseline,
Days -112 to -63
(+7 days at Sponsor discretion)[d]</td><td rowspan="14">≤9 weeks before Baseline,
Days -63 to -1[d]</td></tr>
<tr><td>2. Informed consent by Subject (and/or LAR) and Study Partner(s) (M[a])</td></tr>
<tr><td>3. Demographics</td></tr>
<tr><td>4. Medical history and concomitant medication review (M)</td></tr>
<tr><td>5. NIA/AA probable AD or MCI-AD clinical diagnosis confirmed (M)</td></tr>
<tr><td>6. MMSE</td></tr>
<tr><td>7. CDR</td></tr>
<tr><td>8. Diagnostic Verification Form (M)</td></tr>
<tr><td>9. Medical assessments (M):
Vital sign measurement[b]
Physical/neurological examinations[c]
12-lead electrocardiogram
Laboratory testing:
Serum chemistry
Hematology
Haptoglobin
Folate / vitamin B_{12}
Thyroid stimulating hormone
Glucose-6-phosphate dehydrogenase
Serum pregnancy test (women of childbearing potential only)</td></tr>
<tr><td>10. Wait for Confirmation of Eligibility email received from MedAvante-ProPhase and laboratory testing confirmation of compliance by Labcorp Central Laboratory Services before moving to step 11</td></tr>
<tr><td>11. Amyloid PET scan
• Documentation of prior positive scan
OR
• If no negative scan within prior 3 years, perform using approved amyloid ligand</td></tr>
<tr><td>12. Wait for Amyloid PET scan confirmation of compliance by local / central imaging center (if scan required)</td></tr>
<tr><td>13. MRI scan</td></tr>
<tr><td>14. Wait for MRI confirmation of compliance by Bioclinica</td></tr>
<tr><td>15. Withdraw AChEI/memantine</td><td>≥ 60 days before baseline assessments</td><td>Not applicable</td></tr>
<tr><td>16. Medical history and concomitant medication review, serum chemistry, hematology, serum pregnancy test (M)</td><td>Repeat within 42 days of Baseline</td><td>Repeat if not performed within 42 days of Baseline</td></tr>
<tr><td>17. ^{18}F-FDG-PET only in subjects with CDR 0.5
(Not an eligibility assessment, but independent confirmation of a valid baseline ^{18}F-FDG-PET)</td><td>Within 42 days before randomization, but at least 60 days after the last dose of AChEI and/or memantine</td><td>Within 42 days before randomization</td></tr>
<tr><td>18. Wait for ^{18}F-FDG-PET confirmation of scan quality by Invicro for subjects with CDR 0.5</td><td colspan="2">Before randomization</td></tr>
</table>

Abbreviations: AChEI = anticholinesterase inhibitor; ACh/Mem = AChEI and/or memantine; AD = Alzheimer's disease; CDR = Clinical Dementia Rating; ^{18}F-FDG = ^{18}F-fluorodeoxyglucose positron emission tomography; LAR = Legally Acceptable Representative; MCI-AD = mild cognitive impairment due to Alzheimer's disease; MMSE = Mini-Mental State Examination; MRI = magnetic resonance imaging; PET = positron emission tomography

Note: (M) requires medical assessor (physician / MD / DO); study partner = caregiver or informant

[a] At all study sites, the PI or SI who obtains informed consent must be a physician (such as a neurologist, psychiatrist) or other medically qualified assessor (physician/MD/DO); in the case of subjects with reduced decision-making capacity, LARs, consistent with national and/or local law, may provide written informed consent
[b] Vital signs include seated blood pressure and pulse, height, and body weight
[c] Based on medical history, subjects with a history of lens implantation should be scheduled for an ophthalmological examination by a licensed ophthalmologist, optometrist, or other suitably qualified medical assessor (physician/MD/DO) to occur prior to the first dose of study drug (once eligibility has been confirmed), during the screening procedures or as part of the baseline assessments
[d] The Screening visit window may be further extended at the discretion of the Sponsor when justification is provided.

10.5.1.1 Diagnostic and Cognitive Eligibility Assessments

After consent is obtained, the principal investigator (PI) (or sub-investigator [SI]) is required to review medical records and confirm the accuracy of the diagnosis during the examination of each subject. The NIA/AA diagnostic interview must be conducted with the subject, and the designated study partner (*i.e.*, a caregiver or informant) providing data from assessments of the subject, by the PI (or SI). In brief:

- Subjects with probable AD dementia must have insidious onset, worsening impairment in at least two cognitive areas (learning and recall, language, executive function, visuospatial skills), sufficient to significantly interfere with work or usual activities, that is not explained by delirium, drugs, major psychiatric disorder, medical illness, cerebrovascular disease, other forms of dementia, or neurological disorder.
- In subjects with MCI-AD, there should be evidence of concern about a change in cognition in comparison with the person’s previous level verified by a knowledgeable informant or clinician. There should also be evidence of lower performance in episodic memory than would be expected for the subject’s age and educational background (typically 1 to 1.5 standard deviations below the mean). Other mild cognitive deficits may also be present, but there must be preservation of independence in functional abilities. Subjects should not meet the criteria for dementia. The cognitive changes must be mild and there must be no evidence of a significant impairment in social or occupational functioning. Impairments must not be explained by delirium, drugs, major psychiatric disorder, medical illness, cerebrovascular disease, other forms of dementia, or neurological disorder.

The diagnostic interview should be completed and documented in the subject’s medical records prior to administering the MMSE and CDR. The MMSE will be the first evaluation at Screening (Visit 1), followed by the CDR, to obtain Severity Ratings. These will be assessed on a Virgil tablet. Note that the CDR assessment should only be performed if the subject meets the MMSE inclusion criterion. If the subject does not qualify with either of these scales, no further assessments should be made and the subject should be considered a screen failure. If eligible, a Diagnostic Verification Form will be completed by the PI/SI and submitted to independent reviewers at MedAvante-ProPhase Inc., who will confirm the accuracy of the diagnosis and Severity Ratings and will provide confirmation (by e-mail) that the subject is eligible to proceed to the remaining screening assessments. The PI must acknowledge receipt of this independent confirmation prior to subject randomization. In the event that MedAvante-ProPhase central reviewers identify issues that cannot be resolved, the subject will be deemed not eligible. The subject may be re-screened (after a wait of 90 days from last MMSE), inclusive of repeat of MMSE and CDR which will again be subject to the independent review to confirm eligibility.

Eligibility assessments include documentation of a previous PET scan that was positive for amyloid. If such documentation is absent, or if the subject has a negative amyloid PET scan that is dated >3 years prior to Screening, an amyloid PET scan must be conducted as part of screening. A negative amyloid PET scan within 3 years prior to Screening is exclusionary and no amyloid PET scan should be performed. If a new amyloid PET scan is performed as part of screening, local determination (or central for those sites not able to read locally) of positivity using criteria routinely applied at the local imaging facility and using an amyloid PET ligand that is approved in the country of participation will be accepted. With subject (and/or LAR) consent, these and previous amyloid PET scans will be sent to a core imaging laboratory for storage and later exploratory analysis.

The screening assessments should be performed so that MRI is the last of the key eligibility assessments to be scheduled. The screening MRI scan(s) results (of sufficient quality) must be available for the purpose of inclusion/exclusion review and confirmation of compliance with inclusion and exclusion criteria by an independent neuroradiologist. If the initial screening MRI is not of sufficient quality, then a repeat screening scan may be performed. If the repeat scan cannot be accomplished within the pertinent window (see Table 10-1), then the subject must be re-consented and re-screened. For subjects who are re-screened for other reasons and an acceptable MRI scan was already completed during the original Screening window, the scan does not require repetition. See Section 10.2 for a summary of alternate arrangements that may be implemented due to COVID-19.

In subjects receiving an AChEI and/or memantine at Screening, documentation of amyloid positive PET imaging (either by prior history or in the sequence of screening tests) and confirmation of compliance with inclusion and exclusion criteria by the independent neuroradiologist (inclusive of screening MRI), must be available prior to the discontinuation of such medication.

At any step, should any of the assessments indicate the subject’s ineligibility, the screening process should be stopped immediately (no further assessments performed) and the subject considered a screen failure. The reason(s) for screen failure is (are) to be documented.

10.5.1.2 Other Medical Screening Assessments

Medical screening assessments will include a review of medical history and concomitant medications. Physical and neurological examinations will be conducted by a qualified medical assessor (physician/MD/DO) at Screening. The complete physical examination is to consist of an evaluation of the skin, head, eyes, ears, nose, throat, neck, thyroid, lungs, heart, lymph nodes, abdomen, and extremities. The complete neurological examination is to consist of an evaluation of appearance and behavior (including observation for tremor and abnormal movements) and an evaluation of the following: speech, cranial nerves (2-12), motor (muscle strength), muscle tone, sensory abnormalities, coordination, gait, and tendon reflexes. At Screening, the primary aim of this assessment is to exclude neurological disorders other than the condition of interest. Any abnormalities noted on either the physical or neurological examination should be described.

An ophthalmological examination will be performed by a licensed optometrist, ophthalmologist, or other suitably qualified medical assessor (physician/MD/DO) prior to the first dose of study drug (during the screening procedures or as part of the baseline assessments) in subjects with a history of lens implantation.

At Screening, vital signs will be measured (seated blood pressure and pulse, body weight, and height), blood obtained for clinical laboratory testing, and a 12-lead ECG performed. Clinical laboratory testing at Screening will include hematology, serum chemistry panels, glucose-6-phosphate-dehydrogenase (G6PD) activity, haptoglobin, vitamin B_{12}, and folate. A serum pregnancy test will be performed in women of childbearing potential only. Thyroid stimulating hormone (TSH) will be measured during screening, with a thyroid hormone panel obtained only in the event of an elevated TSH, if clinically warranted, to determine further treatment before re-screening. Clinical laboratory testing (serum chemistry, hematology, and serum pregnancy test) and medical history and concomitant medication review should be repeated within 42 days of Baseline if the initial screening assessments were performed earlier. Review of the clinical laboratory and ECG results should be performed by a medical assessor (physician/MD/DO).

Should a subject be found to have met one or more of the exclusion criteria, he/she will be considered a screen failure; the reason(s) will be documented. For eligible subjects, findings will be documented in the subject's medical record and in the electronic Case Report Form (eCRF).

10.5.2 Baseline and Post-randomization Assessments in Double-Blind Treatment Period

The baseline and post-randomization assessments during the double-blind treatment period are presented below in Table 10-2. The procedures are further described, briefly, in the subsections that follow (with reference to more detailed discussions). See Section 10.2 for a summary of alternate arrangements that may be implemented related to COVID-19.

Table 10-2: Schedule of Post-Screening Assessments (Double-Blind Treatment Period)

Visit Name	Baseline[a]		Double-Blind Treatment Period				
Visit Number	2		3	4	5	6	7 (or ET) Also Baseline/ Day 1 for Open-Label Phase
Weeks Relative to Baseline	-		4 weeks	13 weeks	26 weeks	39 weeks	52 weeks
Allowable Time Window in Days	Pre-Dose	Post-Dose	(±3)	(±14)	(±14)	(±14)	(±14)
^{18}F-FDG-PET (see Table 10-1) [b]							X
Randomization	X						
MRI [c]				X	X	X	X
ADAS-cog$_{13}$ and ADCS-ADL$_{23}$ [d, o]	X			X	X	X	X
AE/Concomitant Medication Recording/Review [e] (M)	X	X	X	X	X	X	X
Targeted Physical/Neurological Examinations [f] (M)	X	X	X	X	X	X	X
Ophthalmological Examination [g] (M/O)	X						X
Clinical Laboratory Testing [h] (M)	X		X	X	X	X	X
Pregnancy Testing [i]	X		X	X	X	X	X
Blood Pressure, Pulse, Body Weight [j]	X	X	X	X	X	X	X
Study Drug Dispensing	X			X	X	X	X[p]
Study Drug Compliance Assessment [k]			X	X	X	X	X
Blood Sample for MT Concentration [l]	X	X	X				X
Blood Sample for Genotyping (optional) [m]	X						
MMSE [n, o]							X
CDR [n, o]							X

Abbreviations: ADAS-cog$_{13}$ = Alzheimer's Disease Assessment Scale – cognitive subscale (13-item); ADCS-ADL$_{23}$ = Alzheimer's Disease Cooperative Study – Activities of Daily Living (23-item); AE = adverse event; CDR = Clinical Dementia Rating; EOT/ET = end of treatment/early termination; ^{18}F-FDG-PET = ^{18}F-fluorodeoxyglucose positron emission tomography; MMSE = Mini-Mental State Examination; MRI = magnetic resonance imaging; MT = methylthioninium.

Note: Screening assessments, which include a PET scan for amyloid and a 12-lead ECG, are described in Table 10-1

Note: (M) = assessment of the result requires medical assessor (physician / doctor of medicine [MD] / doctor of osteopathic medicine [DO]); M/O = licensed optometrist/ophthalmologist or other suitably qualified medical assessor (physician/MD/DO)

a The Baseline visit (Visit 2) may span over 2-3 days if necessary; Day 1 will commence with the administration of the first in-clinic dose of study drug.

b ^{18}F-FDG-PET should be performed only in subjects confirmed to meet all inclusion and exclusion criteria and a screening CDR 0.5. The Baseline ^{18}F-FDG-PET should be performed within a 42-day window prior to Baseline (Visit 2), at least 60 days after the last dose of AChEI and/or memantine, and as described in Table 10-1. A valid scan must be confirmed by the independent neuroradiologist prior to randomization at Visit 2.
If the subject's last ^{18}F-FDG-PET scan was performed ≥30 days prior to the early termination date, the ^{18}F-FDG-PET scan must be repeated as part of the ET visit assessments within the time window of the EOT/ET visit (*i.e.*, ± 14 days of the EOT/ET visit).
Note: Use of sedatives is not permitted within 24-hours prior to ^{18}F-FDG-PET scans and the subject's fasting (for at least 4 to 6 hours) blood glucose should be <180 mg/dL (<9.9 mmol) for the injection to take place.

c Volumetric MRI scans will be obtained at Screening (which also will be the baseline scan) and approximately every 13 weeks after randomization; the screening MRI will also be used for purposes of confirmation of compliance with inclusion and exclusion criteria by an independent neuroradiologist. Subjects may or may not be receiving concomitant treatment with AChEI and/or memantine at the time of the screening MRI; the screening MRI will be used as the baseline. For subjects who terminate early, if the subject's last MRI scan was performed <90 days prior to the ET date, no additional scan is required.

d At each applicable time point, clinical efficacy assessments should be administered before vital signs measurement and blood sample collection. All ADAS-cog$_{13}$ and ADCS-ADL$_{23}$ assessments will be subject to central review by MedAvante-ProPhase clinicians in the native language.

e AEs and concomitant medication use will be evaluated by a medical assessor (M) and recorded on an ongoing basis starting after signing of informed consent, including at each scheduled visit; unscheduled visits should occur as needed to follow up an AE (including after the last dose of study drug), consistent with the physician/MD/DO judgment.

[f] Targeted examinations will be performed by a medical assessor (M) pre-dose and approximately 3 hours after administration of the first dose of study drug (Visit 2). Thereafter, targeted examinations are to be performed at each subsequent clinic visit (or upon ET). At a minimum, targeted examinations should include heart and lung auscultation and brief neurological assessment guided by any reported signs/symptoms/AEs (*e.g.*, evaluating subjects for potential serotonin toxicity).

[g] Ophthalmological examination (slit lamp) is to be performed only in those subjects with history of lens implant to evaluate discoloration of lens. The initial examination will be performed prior to the first dose of study drug (during the screening procedures or as part of the baseline assessments); performance of this examination is not restricted to the 2 to 3 days before the first dose of study drug. The final evaluation (EOT/ET) should be done after completion of the efficacy assessments.

[h] At each identified time point, blood samples will be obtained for chemistry and hematology panels; results are to be reviewed by a medical assessor (M). Testing is also to be performed as needed to follow-up on an AE.

[i] Serum pregnancy testing is to be performed at each identified time point in women of childbearing potential only.

[j] Within 1 hour prior to dosing on Day 1, blood pressure and pulse will be measured with subjects in a seated position (for at least 5 minutes). The post-dose measurement on Day 1 is to be made approximately 2 hours post-dose (after the 1-2 hour PK sample is taken). Body weight will be measured at each visit (pre-dose at Visit 2).

[k] Compliance with study drug will be assessed by counting returned tablets (see Section 12.6).

[l] At Visits 2, 3, and 7, blood will be collected on *the same day on three occasions*, pre-dose and again approximately 1 to 2 hours post-dose (these should be morning appointments); and a third sample with time of dose and sample at approximately 4 hours after the dose. Note: The time of the dose from the previous day must be collected and recorded at Visits 3 and 7, in addition to the time of the dose given in the clinic. For any ET visit, if the subject has not yet discontinued study drug and is willing to take a final in-clinic dose, three blood samples will be collected as described above. If, however, the subject has already discontinued study drug or is unwilling to take a final in-clinic dose, a single blood sample only for determination of MT concentrations will be collected, irrespective of the date or time of the last dose of study drug. The date and time of last dose must be collected and recorded. If a subject has discontinued study drug but wants to continue to attend study visits off-treatment (the off treatment on study [TOTOS] group), then MT concentration blood samples will not continue to be collected.

[m] A single blood sample for *ApoE* can be collected any time after eligibility for randomization and continued participation in the study has been confirmed (but prior to Visit 7, the end of the double-blind treatment period) for subjects who provide legally acceptable consent.

[n] These are to be rated by the same rater, where possible, as at Screening, after all other efficacy assessments have been completed. MMSE and CDR assessments will be subject to central review by MedAvante-ProPhase clinicians in the native language.

[o] The clinical efficacy scale assessments should be performed at approximately the same time of day throughout the study for a given subject, to the greatest extent possible.

[p] In-clinic dose at Visit 7 to be taken after pre-dose assessments, from newly dispensed Visit 7 open-label study drug kit (see Table 10-3 for the pre-dose and post-dose assessments to be performed at Visit 7).

10.5.2.1 Imaging Efficacy Assessments in Double-Blind Treatment Period

Only in subjects with CDR 0.5 at Screening, ^{18}F-FDG-PET will be performed within the 42 days before Baseline (but not earlier) and after 52 weeks of double-blind treatment or upon early termination. The purpose is to assess reduction in decline of glucose uptake in regions of interest. If the subject's last ^{18}F-FDG-PET scan was performed ≥30 days prior to the early termination date, the ^{18}F-FDG-PET scan must be repeated as part of the early termination visit assessments within the time window of the early termination visit (*i.e.*, ± 14 days of the last dose of study drug). ^{18}F-FDG-PET data will be evaluated by an independent, nuclear medicine staff physician experienced in neuro ^{18}F-FDG-PET, not involved in the clinical conduct of the study, and trained on the study endpoints. Note: The use of sedatives is not permitted within 24 hours prior to ^{18}F-FDG-PET scans and the subject should have a fasting (at least 4 to 6 hours) blood glucose level <180 mg/dL (<9.9 mmol/L) in order for the injection to take place.

Brain MRI (obtained at Screening and approximately every 13 weeks during the double-blind treatment period after randomization or upon early termination) will be evaluated for change in various parameters to determine whether there is reduction of temporal, parietal, whole brain, lateral ventricular, hippocampal, putamen, nucleus accumbens, and nucleus basalis

atrophy rates over the period of the study. Changes in the brain volumes above will be quantified at the imaging core laboratory.

For subjects who terminate early, if the subject's last MRI scan was performed <90 days prior to the early termination date, no additional MRI scan is required.

The obtainment and assessment of imaging data are further described in Section 15.3.

10.5.2.2 Clinical Efficacy Assessments in Double-Blind Treatment Period

The ADAS-cog_{13} and ADCS-ADL_{23} will be performed at Baseline and every 13 weeks after randomization (or upon early termination) during the double-blind treatment period by an approved Efficacy Rater using electronic tablets. Assessments will be recorded and reviewed independently as further described in Section 15.2.

As exploratory clinical efficacy assessments, the MMSE and CDR sum of boxes will be performed after 52 weeks of double-blind treatment (or upon early termination). For a given subject, these are to be performed by the same rater, where possible, who made the screening assessments and recorded on electronic tablets, as further described in Section 15.2. These are to be the last assessments performed, *i.e.*, after all other efficacy assessments have been completed.

All clinical efficacy assessments will be subject to independent review (all subjects, all applicable time points).

10.5.2.3 Safety Assessments in Double-Blind Treatment Period

Safety assessments (described in Section 15.4) will be performed throughout study participation, including at Baseline (prior to dosing and during the 4-hour post-dose evaluation at Visit 2) and during the balance of the double-blind treatment period (Visits 3, 4, 5, 6, and 7, or upon early termination). Safety assessments will be performed by an independent qualified assessor not involved in efficacy assessments; where specified, the assessments must be made by a medical assessor (physician/MD/DO). Study visits during the double-blind treatment period will occur at time points approximately 4, 13, 26, 39, and 52 weeks after Baseline.

At each in-clinic visit, AEs and changes in concomitant medications will be recorded. These must be reviewed by a medical assessor (physician/MD/DO).

Other post-screening assessments include measuring vital signs, performing targeted physical and neurological examinations, clinical laboratory testing (*e.g.*, hematology, serum chemistry panels), and serum pregnancy testing (women of childbearing potential only). The targeted physical and neurological examinations and review of the clinical laboratory results should be performed by a medical assessor (physician/MD/DO).

Subjects with a history of lens implant will undergo an ophthalmologic examination by a licensed optometrist, ophthalmologist, or other suitably qualified medical assessor (physician/MD/DO) prior to the first dose of study drug (during the screening procedures or as part of the baseline assessments) and at Visit 7 of the double-blind treatment period or

upon early termination (after completion of the efficacy assessments), to evaluate whether the lens has become discolored. Sites that do not have access to a licensed optometrist, ophthalmologist, or other suitably qualified medical assessor (physician/MD/DO) are not permitted to screen subjects with a history of lens implant.

10.5.2.4 Other Assessments in Double-Blind Treatment Period

At Visits 2, 3, and 7, blood will be collected for determination of MT concentrations. Samples will be collected pre-dose, approximately 1 to 2 hours post-dose, and approximately 4 hours post-dose. To achieve this sample collection schedule, these should be morning appointments (and for Visits 3 and 7, subjects instructed not to take their morning dose at home). See Section 15.5.1 for further details.

For any early termination visit during the double-blind treatment period, also refer to Section 15.5.1 for blood sampling requirements for determination of MT concentrations.

ApoE genotype will be determined for subjects who provide legally acceptable consent. A single blood sample may be collected any time after eligibility for randomization and continued participation in the study has been confirmed at Baseline (Visit 2), but prior to Visit 7 (end of the double-blind treatment period). Sample collection is further described in Section 15.5.2.

10.5.3 Assessments in the Open-Label, Delayed-Start Phase

The assessments during the open-label, delayed-start phase are presented below in Table 10-3. The procedures are further described, briefly, in the subsections that follow (with reference to more detailed discussions in Section 15). See Section 10.2 for a summary of alternate arrangements that may be implemented due to COVID-19.

Table 10-3: Schedule of Assessments (Open-Label, Delayed-Start Phase)

Visit Name	Baseline/Day 1 for Open-Label Phase		Continued Open-Label Treatment		
**Visit Number**	**7**		**8 (TC)**	**9**	**10 (OL-EOT or OL-ET)**
**Weeks Relative to Visit 2 Baseline**	**52 weeks**		**56 weeks**	**78 weeks**	**104 weeks**
**Allowable Time Window in Days**	**(±14 days)**		**(±3 days)**	**(±14 days)**	**(±14 days)**
	**Pre-Dose**	**Post-Dose**			
^{18}F-FDG-PET (see Table 10-1 and Table 10-2)	X				
MRI [a]	X			X	X
ADAS-cog$_{13}$ and ADCS-ADL$_{23}$ [b, l]	X			X	X
AE/Concomitant Medication Recording/Review [c] (M)	X	X	X	X	X
Targeted Physical/Neurological Examinations [d] (M)	X			X	X
Ophthalmological Examination[e] (M/O)	X				X
Clinical Laboratory testing [f] (M)	X			X	X
Pregnancy Testing [g]	X			X	X
Blood Pressure, Pulse, Body Weight [h]	X			X	X
Study Drug Dispensing	X[m]			X	
Study Drug Compliance Assessment [i]	X		X	X	X
Blood Sample for MT Concentration [j]	X	X			X
MMSE [k, l]	X				X
CDR [k, l]	X				X

Abbreviations: ADAS-cog$_{13}$ = Alzheimer's Disease Assessment Scale – cognitive subscale (13-item); ADCS-ADL$_{23}$ = Alzheimer's Disease Cooperative Study – Activities of Daily Living (23-item); AE = adverse event; CDR = Clinical Dementia Rating; MMSE = Mini-Mental State Examination; MRI = magnetic resonance imaging; OL-EOT = open-label end of treatment; OL-ET = open-label early termination; TC = telephone contact.

Note: Visit 7 results from Table 10-2 provide baseline values for the open-label, delayed-start phase.

Note: (M) = requires medical assessor (physician/doctor of medicine [MD]/doctor of osteopathy [DO]); M/O = licensed optometrist/ophthalmologist or other suitably qualified medical assessor (physician/MD/DO) is also acceptable

a For subjects who terminate early, if the subject's last MRI scan was performed <90 days prior to the ET date, no additional scan is required.

b At each applicable time point, clinical efficacy assessments should be administered before vital signs measurement and blood sample collection. All ADAS-cog$_{13}$ and ADCS-ADL$_{23}$ assessments will be subject to central review by MedAvante-ProPhase clinicians in the native language.

c AEs and concomitant medication use will be evaluated by a medical assessor and recorded on an ongoing basis starting after signing of informed consent, including at each scheduled visit; unscheduled visits should occur as needed to follow up an AE (including after the last dose of study drug), consistent with the physician/MD/DO judgment.

d Targeted examinations are to be performed by a medical assessor at each clinic visit (or upon ET). At a minimum, targeted examinations should include heart and lung auscultation and brief neurological assessment guided by any reported signs/symptoms/AEs (*e.g.*, evaluating subjects for potential serotonin toxicity).

e Ophthalmological examination (slit lamp) is to be performed only in those subjects with history of lens implant to evaluate discoloration of lens. The final evaluation (OL-EOT or OL-ET) should be done after completion of the efficacy assessments.

f At each identified time point, blood samples will be obtained for chemistry and hematology panels; results are to be medically reviewed by a medical assessor. Testing is also to be performed as needed to follow-up on an AE.

g Serum pregnancy testing is to be performed at each identified time point in women of childbearing potential only.

h Blood pressure and pulse will be measured with subjects in a seated position (for at least 5 minutes).

i Compliance with study drug will be assessed by counting returned tablets (see Section 12.6); compliance will also be queried in the TC.

j At Visits 7 and 10, blood will be collected on *the same day on three occasions*, pre-dose and again approximately 1 to 2 hours post-dose (these should be morning appointments); and a third sample with time of dose and sample at approximately 4 hours after the dose. Note: The time of the dose from the previous day must be collected and recorded at Visits 7 and 10, in addition to the time of the dose given in the clinic. For any ET visit, if the subject has not yet discontinued study drug and is willing to take a final in-clinic dose, three blood samples will be collected as described above. If, however, the subject has already discontinued study drug or is unwilling to take a final in-clinic dose, a single blood sample only for determination of MT concentrations will be collected, irrespective of the date or time of the last dose of study drug. The date and time of last dose must be collected and recorded. If a subject has discontinued study drug but wants to continue to attend study visits off-treatment (TOTOS group), then MT concentration blood samples will not continue to be collected.

k These are to be rated by the same rater, where possible, as at Screening, after all other efficacy assessments have been completed. MMSE and CDR assessments will be subject to central review by MedAvante-ProPhase clinicians in the native language.

l The clinical efficacy scale assessments should be performed at approximately the same time of day throughout the study for a given subject, to the greatest extent possible.

m In-clinic dose at Visit 7 to be taken after pre-dose assessments, from newly dispensed Visit 7 open-label study drug kit.

10.5.3.1 Imaging Efficacy Assessments in the Open-Label, Delayed-Start Phase

Brain MRI will be obtained after the additional 26 and 52 weeks of open-label treatment (Visit 9/Week 78 and Visit 10/Week 104) and will be evaluated and quantified as briefly described in Section 10.5.2.1 for the double-blind treatment period. The obtainment and assessment of imaging data are further described in Section 15.3.

For subjects who terminate early, if the subject's last MRI scan was performed <90 days prior to the early termination date, no additional MRI scan is required.

10.5.3.2 Clinical Efficacy Assessments in the Open-Label, Delayed-Start Phase

The ADAS-cog$_{13}$ and ADCS-ADL$_{23}$ will be performed after the additional 26 and 52 weeks of open-label treatment (Visit 9/Week 78 and Visit 10/Week 104) by an approved Efficacy Rater using electronic tablets. Assessments will be recorded and reviewed independently as further described in Section 15.2.

As exploratory clinical efficacy assessments, the MMSE and CDR sum of boxes will be performed after the additional 52 weeks of open-label treatment (Visit 10/Week 104 or upon early termination). These are to be performed by the same rater, where possible, who made the screening assessments and recorded on electronic tablets, as further described in Section 15.2. These are to be the last assessments performed, *i.e.*, after all other efficacy assessments have been completed.

All clinical efficacy assessments will be subject to independent review (all subjects, all applicable time points).

10.5.3.3 Safety Assessments in the Open-Label, Delayed-Start Phase

Safety assessments (described in Section 15.3) will be performed after 26 and 52 weeks (Visit 9/Week 78 and Visit 10/Week 104) of open-label treatment and will include AE review, concomitant medication recording and review, targeted physical and neurological examinations, clinical laboratory testing (*e.g.*, hematology and serum chemistry panels), serum pregnancy testing (women of childbearing potential only), and vital sign measurements. In addition, AEs and changes in concomitant medications will be recorded during a telephone contact after 4 weeks of open-label treatment (Visit 8/Week 56). The targeted physical and neurological examinations along with review of any AEs, changes in concomitant medications, and clinical laboratory results should be performed by a medical assessor (physician/MD/DO).

Subjects with a history of lens implant will undergo an ophthalmologic examination by a licensed optometrist, ophthalmologist, or other suitably qualified medical assessor (physician/MD/DO) at the final visit of the open-label treatment period (Visit 10/Week 104), or upon early termination (after completion of the efficacy assessments), to evaluate whether the lens has become discolored.

10.5.3.4 Other Assessments in Open-Label, Delayed-Start Phase

At Visit 10/Week 104, blood will be collected for determination of MT concentrations. Samples will be collected pre-dose, approximately 1 to 2 hours post-dose, and approximately 4 hours post-dose. To achieve this sample collection schedule, this should be a morning appointment and subjects instructed not to take their morning dose at home. See Section 15.5.1 for further details.

For any early termination visit during the open-label phase, also refer to Section 15.5.1 for blood sampling requirements for determination of MT concentrations.

10.6 Data and Safety Monitoring Board

Safety will be overseen by a Data and Safety Monitoring Board (DSMB) throughout the duration of study conduct. At any time, the DSMB may recommend that dosing be modified or enrollment stopped due to safety concerns. The DSMB may also request to receive additional data unblinded to the subject level in response to identified safety concerns.

Routine meetings are to be scheduled as determined by the DSMB. *Ad hoc* meetings will be convened if needed in response to safety concerns. The DSMB Charter will describe the composition of the DSMB and safety monitoring details, as well as the frequency of meetings needed as the study progresses.

10.7 Definition of End of Study

The end of study will occur when the last subject has completed the final study visit in the open-label, delayed-start phase (whether or not the visit occurs as scheduled). As noted in Section 17.9, an analysis will be performed after the last subject completes the last visit in the double-blind treatment period.

Subjects who complete the study and receive treatment with LMTM up to and including the last open-label visit may be offered an opportunity to subsequently receive treatment with LMTM in a separate EAP.

11 SUBJECT ENROLLMENT AND WITHDRAWAL

11.1 Inclusion Criteria

To be eligible for enrollment in this study, a subject must meet all of the following inclusion criteria:

1. AD, encompassing probable AD and MCI-AD based on 2011 NIA/AA criteria:
   - All cause dementia and probable AD (probable AD)
     In brief, subjects with probable AD dementia must have insidious onset,

worsening impairment in at least two cognitive areas (learning and recall, language, executive function, visuospatial skills), sufficient to significantly interfere with work or usual activities, that is not explained by delirium, drugs, major psychiatric disorder, medical illness, cerebrovascular disease, other forms of dementia, or neurological disorder. The accuracy of the diagnosis will be confirmed independently by the diagnosing physician at site.

OR

- MCI-AD
  In subjects with MCI-AD, there should be evidence of concern about a change in cognition, in comparison with the person's previous level verified by a knowledgeable informant or clinician. Other mild cognitive deficits may also be present, but there must be preservation of independence in functional abilities. Subjects should not meet the criteria for dementia. The cognitive changes must be mild and there must be no evidence of a significant impairment in social or occupational functioning. Impairments must not be explained by delirium, drugs, major psychiatric disorder, medical illness, cerebrovascular disease, other forms of dementia, or neurological disorder.

2. Documented PET scan that is positive for amyloid; if most recent PET scan was performed >3 years prior to Screening and was negative, it may be repeated (a negative amyloid PET scan within the 3 years prior to Screening is exclusionary)
3. MMSE score of 16-27 (inclusive) at Screening, subject to stratification requirements
4. Global CDR score of 0.5 to 2 at Screening (if 0.5, including a score of >0 in one of the functional domains: Community Affairs, Home and Hobbies, or Personal Care)
5. Age <90 years at Screening
6. Females must meet one of the following:
   - Surgically sterile (hysterectomy, bilateral salpingectomy / oophorectomy) for at least 6 months minimum
   - Have undergone bilateral tubal occlusion / ligation at least 6 months prior
   - Post-menopausal for at least 1 year
   - Using adequate contraception (a barrier method [such as condom, diaphragm or cervical/vault cap] with spermicidal foam, gel, film, cream, or suppository; intrauterine device [IUD] or system, or oral or long-acting injected or implanted hormonal contraceptives for at least 90 days prior to Baseline; or vasectomized partner [with the appropriate post-vasectomy documentation of the absence of spermatozoa in the ejaculate]) or true abstinence (when this is in line with the preferred and usual lifestyle of the subject); subjects must be competent to use adequate contraception and to agree to continue to maintain adequate contraception throughout participation in the study (including up to 4 weeks after the last dose of study drug)
7. Subject and/or, in the case of reduced decision-making capacity, legally acceptable representative(s) (LAR(s)), consistent with local and national law, is able to read, understand, and provide written informed consent in the designated language of the study site
8. Has one (or more) identified adult study partner (*i.e.*, a caregiver or informant) who meets the following criteria:
   - Either lives with the subject, or in the investigator's opinion, the extent of contact is sufficient to provide meaningful assessment of changes in subject

behavior and function over time and provide information on safety and tolerability (*e.g.,* sees the subject on average for ≥1 hour/day ≥3 days/week)
- Is willing to provide written informed consent for his/her own participation
- Is able to read, understand, and speak the designated language(s) at the study site
- Agrees to accompany the subject to each study visit
- Is able to verify compliance with study drug

9. The subject must not have been taking either an AChEI, *i.e.*, donepezil, galantamine, or rivastigmine, and/or memantine, for at least 60 days at the time of the baseline assessments
   - Subjects never previously treated with an AChEI and/or memantine may be enrolled if initiation of treatment with these medications is not planned for the time period during which the subject will be participating in this study
10. Able to comply with the study procedures in the view of the investigator

11.2 Exclusion Criteria

The exclusion criteria are:

1. Significant central nervous system (CNS) disorder other than probable AD or MCI-AD, *e.g*., Lewy body dementia, Parkinson’s disease, multiple sclerosis, progressive supranuclear palsy, hydrocephalus, Huntington’s disease, any condition directly or indirectly caused by Transmissible Spongiform Encephalopathy (TSE), Creutzfeldt-Jakob Disease (CJD), variant Creutzfeldt-Jakob Disease (vCJD), or new variant Creutzfeldt-Jakob Disease (nvCJD)
2. Significant intracranial focal or vascular pathology seen on brain MRI scan that would, based on the independent reviewer imaging evaluation, lead to a diagnosis other than probable AD or MCI-AD, including but not limited to:
   - Large confluent white matter hyperintense lesions (*i.e.*, Fazekas score of 3)
   - Other focal brain lesions judged clinically relevant by the investigator
   - Evidence of a prior or current macrohemorrhage
3. Clinical evidence or history of any of the following (within specified period prior to Baseline):
   - Cerebrovascular accident (2 years)
   - Transient ischemic attack (6 months)
   - Significant head injury, for example, associated loss of consciousness, skull fracture or persisting cognitive impairment (2 years)
   - Other unexplained or recurrent loss of consciousness ≥15 minutes (2 years)
4. Diagnosed with epilepsy (a single prior seizure >6 months prior to Screening is considered acceptable)
5. Diagnostic and Statistical Manual of Mental Disorders, Fifth Edition criteria met (for any of the following within specified period):
   - Major depressive disorder (current)
   - Schizophrenia (lifetime)
   - Other psychotic disorders, bipolar disorder (within the past 5 years)
   - Substance (including alcohol) related disorders (within the past 2 years)

6. Metal implants in the head (except dental), pacemaker, cochlear implants, or any other non-removable items that are contraindications to MRI. MRI compatible prosthetics, clips, stents, or any other device proven to be compatible are allowed
7. Resides in hospital or moderate to high dependency continuous care facility (residence in low grade assisted living facility where there is sufficient autonomy to permit valid evaluation of activities of daily living is allowed so long as it is not mandated by an order issued either by the judicial or the administrative authorities)
8. Any physical disability that would prevent completion of study procedures or assessments (*e.g*., blindness or significant uncorrected visual impairment, deafness or significant hearing loss not corrected by hearing aids, non-AD-related speech impairment)
9. History of swallowing difficulties (note: study drug should be swallowed whole and MUST NOT be broken, crushed, chewed, or dissolved in fluids prior to ingestion)
10. Pregnant or breastfeeding
11. G6PD deficiency based on World Health Organization (WHO) classification (<60% of normal, *i.e.,* <6.1 U/g hemoglobin [Hgb])
12. History of significant hematological abnormality or current acute or chronic clinically significant abnormality, including:
    - History of hemoglobinopathy, myelodysplastic syndrome, hemolytic anemia, or splenectomy
    - Screening Hgb value (confirmed upon repeat) below age/sex appropriate lower limit of the central laboratory normal range

    Subjects in whom folate is <4.0 ng/mL may be entered into the study provided folate supplementation (approximately 1 mg/day) is initiated and maintained for the duration of the study; subjects in whom vitamin B_{12} is <150 pg/mL can be allowed if the investigator confirms that it does not affect the cognitive state of the subject and that the subject is supplemented as appropriate prior to the initiation of study drug
13. Abnormal serum chemistry laboratory value at Screening deemed to be clinically significant by the investigator. In addition, subjects with either of the following abnormalities must be excluded:
    - Creatinine clearance <30 mL/min, estimated by the central laboratory according to the Cockcroft and Gault equation
    - TSH above laboratory normal range (subject may be treated [if clinically indicated based on further laboratory testing] and re-screened after 90 days)
14. Clinically significant cardiovascular disease or abnormal assessments (based on the investigator's interpretation of the locally obtained ECG) such as:
    - Hospitalization for acute coronary syndrome (acute myocardial infarction or unstable angina) or symptoms consistent with angina pectoris, within the 12 months preceding Baseline
    - Signs or symptoms of clinical heart failure within the 12 months preceding Baseline
    - Atrial fibrillation on screening ECG or history of atrial fibrillation that is not currently controlled (heart rate ≥85 bpm and/or inappropriate anticoagulation)
    - QTcF (QT corrected for heart rate using Fridericia's formula) at Screening >460 msec in males or >470 msec in females, or low or flat T waves making measurement of QT interval unreliable

- Recent history of poorly controlled hypertension, systolic blood pressure >180 mmHg, or diastolic blood pressure >100 mmHg, after 5 minutes in a seated position at Screening
- Hypotension: systolic blood pressure <100 mmHg after 5 minutes in a seated position at Screening
- Heart rate <48 bpm or >96 bpm by measurement of vital signs (after 5 minutes in a seated position) or by local ECG at Screening

15. Pre-existing or current signs or symptoms of respiratory failure, *e.g.*, caused by chronic obstructive pulmonary disease, bronchial asthma, lung fibrosis, or other disease
    - Subjects with currently diagnosed moderate to severe sleep apnea should be excluded; the definition of moderate to severe includes oxygen supplementation, *e.g.*, nasal prongs or Continuous Positive Airway Pressure (CPAP)
16. Concurrent acute or chronic clinically significant (in the opinion of the investigator) immunologic, hepatobiliary (such as presence of encephalopathy or ascites), or endocrine disease (not adequately treated), and/or other unstable or major disease other than probable AD or MCI-AD; the following are specifically excluded:
    - Active hepatitis or primary biliary cirrhosis
    - Active Human T-Cell Lymphocytic Virus Type III (HTLV-III), Lymphadenopathy Associated Virus (LAV), any mutants or derivatives of HLTV-III or LAV, any condition associated with active Acquired Immunodeficiency Syndrome or similar condition however named
17. Diagnosis of cancer (excluding basal cell carcinoma, squamous cell carcinoma, or prostate carcinoma in situ [Stage 1]) meeting either of the following criteria:
    - Newly diagnosed within past 2 years
    - Previous (>2 years) diagnosis of cancer that has required any form of intervention or treatment within the past 2 years, *e.g.*, chemotherapy, radiotherapy, hormonal therapy, or surgery
18. Prior intolerance or hypersensitivity to MT-containing drug or methemoglobinemia induced by MT-containing drug, similar organic dyes, or any of the excipients
19. Treatment currently or within 90 days before Baseline with any of the following:
    - Souvenaid®
    - Antipsychotics
        - Clozapine (and there is no intent to initiate therapy during the course of the study)
        - Other antipsychotics are allowable provided they have not been initiated within 90 days before Baseline and preferably at a stable dose and regimen
    - Carbamazepine, primidone, valproate
    - Drugs for which there is a warning or precaution in the labeling about methemoglobinemia at approved doses (*e.g.*, dapsone, local anesthetics such as benzocaine used chronically, primaquine, and related antimalarials)
20. Current or prior participation in a clinical trial as follows:
    - Any clinical trial of LMTM
    - Clinical trial of a product for cognition prior to Baseline in which the last dose was received within 90 days prior to Baseline unless confirmed to have been randomized to placebo

- A clinical trial of any other investigational drug, biologic, device, or medical food in which the last dose was received within 28 days prior to Baseline

11.3 Re-screening

Re-screening of a subject is allowed in selected instances, as listed below (to a maximum of two re-screening occasions). Upon re-consent and re-screening, the subject must continue to meet all of the inclusion and exclusion criteria described in Section 11.1 and Section 11.2, respectively. Prior to re-consent, the PI (or SI) should confirm that the subject continues to meet the diagnosis of AD, encompassing probable AD and MCI-AD, of the acceptable severity (MMSE 16-27 and CDR score of 0.5 to 2 [if 0.5, including a score of >0 in one of the functional domains]), and has not used an AChEI and/or memantine for at least 60 days prior to the baseline assessments (inclusive of the baseline ^{18}F-FDG-PET scan in subjects who have a screening CDR of 0.5). Screening may also be paused at a given site due to COVID-19 as described in Section 10.2.1; such subjects do not require re-consent but otherwise, the principles listed below with respect to repeat testing apply.

1. At the initial Screening, MMSE score will be obtained first to determine eligibility. CDR scores will also be obtained for eligibility. A Diagnostic Verification Form will be completed to confirm diagnosis of probable AD or MCI-AD. These assessments will be subject to independent review. If scoring discrepancies are found that cannot be resolved, the subject will be deemed not eligible. The subject may be re-screened (after a wait of 90 days from last MMSE), inclusive of repeat of MMSE and CDR which will again be subject to the independent review to confirm eligibility. For the avoidance of doubt, re-screening is not allowed if a valid MMSE or CDR score falls outside the acceptable criteria.
2. If the initial screening MRI scan is not of sufficient quality as determined by the core imaging laboratory, a repeat scan may be performed. If the repeat scan cannot be accomplished within the time window specified in Table 10-1, the subject must be re-consented and re-screened. For subjects who are re-screened for other reasons and an acceptable MRI scan was already completed during the original Screening window, the scan does not require repetition.
3. Evidence of atrial fibrillation on initial screening ECG or history of atrial fibrillation that is not currently controlled (heart rate ≥85 bpm and/or inappropriate anticoagulation) is not allowed. If better control of the heart rate and/or of anticoagulation can be achieved after adequate treatment, the subject may be entered into the study if still within the 42-day window; otherwise the subject must be re-consented and re-screened. A cardiology consult should be sought for further ECG evaluation if deemed necessary by the investigator.
4. Subjects with vitamin B_{12} levels <150 pg/mL at initial screening which cannot be corrected during the Screening period may be reconsented and re-screened after the deficit has been corrected (see Section 13.8 for further details).
5. Subjects with a TSH above laboratory normal range at the initial screening may be treated (if clinically indicated) and re-consented and re-screened after 90 days.

Any other criterion not listed above would require justification and approval by TauRx.

No other minimum time is required for re-screening unless specifically stated above or elsewhere in the protocol.

11.4 Discontinuations / Withdrawals

11.4.1 Handling of Subjects Who Discontinue Study Drug

For a discussion of reasons for permanent discontinuation of study medication on the basis of safety, see Section 16.11. These include, but are not limited to, clinically evident hemolytic anemia (Section 16.11.1) and decrease in renal function when it raises renal concerns (Section 16.11.3).

Subjects may discontinue study drug at any time for any reason. Furthermore, the investigator also has the right to discontinue study medication if he or she judges that treatment is no longer appropriate, if the subject's clinical condition is worsening, or for an AE.

If study drug is discontinued, the reason and the last date of study drug should be recorded on the eCRF.

For subjects who cease taking study drug but who wish to continue in the study, the planned schedule of assessments should be followed, except for the collection of blood samples for MT concentrations. These subjects will be classified as "the off-treatment-on-study" (TOTOS) group. Subjects who continue in the study off-treatment will not be eligible for a separate EAP. The handling of data for these subjects is discussed in Section 17.4.11.4.

As discussed in Section 13.1, study drug must be discontinued in subjects who initiate treatment with an AChEI and/or memantine. The handling of data for these subjects is discussed in Section 17.4.11.3.

11.4.2 Handling of Study Discontinuation / Withdrawal

Subjects may withdraw (drop out) from the study at any time for any reason. A study partner (who is functioning as an informant) may also withdraw his or her consent from study participation at any time for any reason. If a study partner withdraws his or her consent, the subject must then also be withdrawn if alternative arrangements are not available (*e.g.*, an alternate study partner). (See Section 11.4.3 for replacement of study partners.)

If the subject withdraws from study participation, the reason should be recorded on the eCRF as one of the following:

- Adverse event
- Death
- Lack of efficacy (including progressive disease or worsening of cognitive capacity)
- Lost to follow-up
- Withdrawal by subject or legally acceptable representative (or study partner), including specific reason(s), wherever possible
- Protocol deviation
- Non-compliance with study drug
- Pregnancy
- Study terminated by Sponsor
- Physician decision, including specific reason(s), wherever possible
- COVID-19
- Other (specify)

If the reason for discontinuation of study is an AE, the principal event associated with discontinuation must be specified and recorded. In this case, reasonable effort must be made to clearly document the outcome. If the reason for premature discontinuation is a serious adverse event (SAE), this must be documented and an SAE form completed.

For subjects who withdraw from the study for reasons other than death or subject/LAR or study partner consent withdrawn, a visit should be scheduled as soon as possible after the last dose of study drug and the End-of-Treatment safety evaluations for the current treatment period performed. For subjects or legally acceptable representatives who withdraw consent or when a study partner withdraws consent without available alternate study partner, the investigator should request that the reason be specified and the subject have any clinically indicated safety assessments performed.

11.4.3 Replacements

Subjects who are withdrawn from the study will not be replaced nor can they be re-enrolled or enter the EAP.

In the event a study partner participating as an informant withdraws, one replacement is normally permitted. However, as noted in Section 10.2.1, up to a total of three replacements may be allowed if necessitated by COVID-19, and the replacement study partner has sufficient contact with the subject to permit informed responses regarding their ADL functioning. Multiple study partners participating only as caregivers will be permitted for a given subject, either simultaneously or as replacements for previous study partners, with no specified maximum.

12 STUDY DRUG

12.1 Treatments Administered: Form, Dosage and Administration

Study drug is available as blue, round, film-coated, immediate-release tablets (5-mm diameter, approximately 55-mg core weight) manufactured and packed for TauRx in accordance with Good Manufacturing Practice (GMP), as detailed in the European Union (EU) Guide to GMP. These and visually matching placebo tablets are debossed on one side with the Greek “Tau” symbol.

12.1.1 Active Ingredient

The active ingredient (drug substance) is LMTM (USAN hydromethylthionine mesylate, also known as leuco-methylthioninium bis(hydromethanesulfonate) or the code name, TRx0237). It is included in the tablets as 4 mg LMT (expressed as MT base equivalent).

12.1.2 Inactive Ingredients

Tablets also contain the following inactive compendial excipients: mannitol, crospovidone, microcrystalline cellulose, and magnesium stearate.

The film coat of study drug tablets contains polyvinyl alcohol-part hydrolyzed, talc, titanium dioxide, Macrogol PEG 3350, lecithin (soya), as well as non-compendial FD&C blue #2 (indigo carmine aluminum lake).

12.2 Study Regimens

All subjects will receive four tablets orally per day (two in the morning and two in the evening) in a double-blind fashion for 52 weeks in one of the following three study regimens, in a 4:1:4 ratio for Protocol Version 5.0 and above:

- LMTM 16-mg/day group: Two 4-mg tablets in the morning, and two 4-mg tablets in the evening
- LMTM 8-mg/day group: One placebo tablet and one 4-mg tablet in the morning, and one placebo tablet and one 4-mg tablet in the evening
- Placebo group: Two “dummy” tablets in the morning, and two “dummy” tablets in the evening; one of which may be replaced by a 4-mg MTC tablet to maintain the treatment blind (the remainder being placebo tablets)

After the completion of the 52-week double-blind treatment period, beginning with supplies dispensed at Visit 7, all subjects will receive open-label LMTM 16 mg/day (two 4-mg tablets in the morning and two 4-mg tablets in the evening) for an additional 52 weeks in the open-label, delayed-start phase of the study.

Tablets may be taken with or without meals. Subjects will be instructed to take each dose of study medication with a full glass of water. Study drug should be swallowed whole and MUST NOT be broken, crushed, chewed, or dissolved in fluids prior to ingestion.

The first dose of study drug (Visit 2) and the morning dose at Visit 3 (after 4 weeks of study drug), Visit 7 (after 52 weeks of study drug), and Visit 10 (after 104 weeks of study drug) will be administered in the clinic. These visits should be scheduled to occur in the morning, with subjects instructed not to take their morning dose at home; subjects will be required to remain in the clinic for at least 4 hours post-dose on each occasion. The time of the dose (and at Visits 3, 7 and 10, also the time of the prior dose taken on the preceding day) will be recorded. Note: the Visit 7 dose must be taken from a newly dispensed Visit 7 open-label treatment study drug kit, after pre-dose assessments have been completed (see Table 10-3 for the pre-dose and post-dose assessments to be performed at Visit 7).

All other doses will be taken on an outpatient basis. The subject/study partner will be asked to establish the times of day that the subject will take the study medication (twice daily), with further inquiry as to whether this will be before or after a meal. They will be asked to adhere, to the best of their capability, to the same administration schedule throughout the study.

Alternative arrangements for dosing and dispensing study drug supplies due to COVID-19 may be permitted and are further described in Section 10.2. Requirements for obtaining subject consent consistent with local and national requirements, maintaining the specified study drug storage conditions, accountability, and compliance monitoring will be addressed and documented.

12.2.1 Maximum Anticipated Dosage

The maximum dosage of study drug will be LMTM 16 mg/day.

Note: Dose reduction is not permitted.

12.2.2 Dose Interruption

Interruption of dosing for up to a maximum of 14 consecutive days may be allowed if the investigator determines this is indicated (*e.g.*, due to an AE or any other reported change in the subject's physical condition in the judgment of the investigator) on a maximum of two occasions. The reason for dose interruption should be recorded in the source documentation. If this is exceeded, study drug would need to be discontinued; however, the subject will be encouraged to continue study participation off-treatment. Subjects who continue in the study off-treatment will not be eligible for a separate EAP.

12.3 Randomization

Subjects will be randomized to one of three study regimens described in Section 12.2: LMTM 16 mg/day, LMTM 8 mg/day, or placebo (4:1:4, at the study level). Randomization will be stratified according to severity (three levels: MMSE 16-19, 20-25, or 26-27) at study level for those randomized to Protocol Version 5.0 and above, prior use of standard AD treatment (two levels: AChEI/memantine or none), and region (two levels: North America or Europe).

Enrollment will be such that subjects are assigned to the MMSE severity groups based on screening MMSE score with a target of approximately 2:3:1 (MMSE 16-19, MMSE 20-25, and MMSE 26-27, respectively) for those randomized to Protocol Version 5.0 and above; to achieve this target, enrollment will be monitored and controlled at the site level for high recruiting sites and capped as needed at the study level.

Randomization, stratification, and enrollment cap requirements will be controlled by a web-based RTSM system.

12.4 Packaging, Labeling, and Storage

Study drug will be packaged, labeled, and distributed to study sites by a designated vendor.

Study drug supplied to subjects will be in individual aluminum blister wallets containing 28 tablets. An appropriate number of blister wallets will be contained within cardboard cartons with sufficient supplies until the next scheduled study visit at which dispensing is planned (see Section 12.5, Dispensing). Additional 28-tablet blister wallets will be included in cartons as needed to allow for delays in visit scheduling (or if original medication is lost or damaged).

Study drug package labels will be compliant with applicable regulatory requirements and will include the statement "Keep out of reach of children," the cautionary statement "Caution: New Drug – Limited by Federal (United States) law to investigational use" and/or "For clinical trial use only" as appropriate, as well as any other locally mandated statements. Labels will also be translated into the local language as required.

At a minimum, labels will also include the following information: the name and address of the Sponsor, the study code, a unique identifier, and appropriate contact information. In those jurisdictions where required, an expiry date will be included.

At the study site, study drug must be stored securely (*e.g.*, locked area, pharmacy) and at a temperature not more than 25°C. The temperature at which study drug is stored at the study

site will be recorded daily using a centralized temperature monitoring system if this is available. If not, study drug storage temperature will be recorded each working day using a maximum-minimum thermometer. The packaging protects the study drug from light and moisture.

Subjects and study partners should also be provided with information about required storage conditions. Study drug should be ingested immediately after removal from the blister wallet.

12.5 Dispensing

At the Baseline/Randomization visit (Visit 2), all subjects/study partners will receive a supply of study drug according to their randomization to take home for use until Visit 4 (13 weeks after Baseline). At subsequent drug-dispensing visits, Visits 4, 5, 6, and 7 (13, 26, 39, and 52 weeks after Baseline, respectively) of the double-blind treatment period, as well as Visit 9 (78 weeks after Baseline) of the open-label delayed-start phase, all subjects/study partners will be required to return the complete study drug kit, including all unused study drug and empty packaging, dispensed at the preceding drug-dispensing visit (Visits 2, 4, 5, 6, and 7) before receiving a re-supply of either randomized study drug or open-label treatment, to take home for use until the next scheduled visit. Visit 7 is Baseline/Day 1 of the open-label treatment phase, at which study drug from a newly dispensed Visit 7 open-label study drug kit should be administered after pre-dose assessments (see Table 10-3 for the pre-dose and post-dose assessments to be performed at Visit 7).

Subjects and study partners will be provided with information about storage conditions and taking study drug, including instructions indicating that study drug must be used only as described in this protocol. They will also be informed that tablets should be swallowed whole and MUST NOT be broken, crushed, chewed, or dissolved in fluids prior to ingestion. If there are swallowing difficulties which prevent taking the medication as instructed, subjects should not be entered into the study. Subjects and study partners should be warned that if the product is not swallowed immediately and is allowed to dissolve in the mouth, it may cause discoloration of teeth and oral mucosa.

In the event of a dose interruption, the subjects and study partners will be provided with updated dosing instructions.

See Section 10.2 for a summary of alternate arrangements that may be implemented due to COVID-19.

12.6 Compliance

At Visits 2, 4, 5, 6, and 7 during the double-blind treatment period and at Visit 9 during the open-label, delayed-start phase, the number of tablets dispensed to the subject/study partner will be recorded.

At the Week 4 visit (Visit 3) and at each clinic visit thereafter (Visits 4 to 7 during the double-blind treatment period, and Visits 9 and 10 during the open-label, delayed-start phase), the subject/study partner will bring the complete study drug kit, including unused study drug and empty packaging, to the study site. The number of tablets (all tablets remaining in unopened blister wallets plus any tablets that have been removed from the blister wallets) will be counted and recorded by study site staff. Following the compliance check, the complete study drug kit, including unused study drug and empty packaging, should

be retained by the study site, except at Visit 3, when the drug kit should be returned to the subject/study partner.

Subject compliance with prescribed study drug will also be assessed at each clinic visit (Visits 4 to 7 during the double-blind treatment period, and Visits 9 and 10 during the open-label, delayed-start phase) as well as during the telephone contact (Visit 8) by questioning the subject and study partner. Any apparent discrepancies between the number of tablets taken and the number of tablets which should have been taken since the last visit will be discussed with the subject and study partner.

Any dose interruptions will also be recorded in the source documentation and will be captured in the eCRF.

If during participation in the study, a subject's compliance is determined to be <80% or >120% (taking into consideration any dose interruptions), the subject and study partner should be re-educated about taking study drug properly and the clinical research associate should be informed promptly. If compliance problems are recurrent, the investigator should inform the clinical research associate and contact the Medical Monitor to determine the course of action.

12.7 Study Drug Accountability

The investigator or designee will keep a record of all study drug received, and of all study drug dispensed to and returned by subjects. Drug accountability will be recorded in the RTSM system.

The investigator will ensure that the supplied study drug will be used only for administration to subjects enrolled in this study and for no other purpose.

The study drug accountability record will be checked by a study monitor at monitoring visits.

All unused and returned study drug will be returned to the Sponsor or designee after study completion according to provided instructions.

12.8 Breaking the Blind for Double-Blind Treatment Period

The randomization list for the double-blind treatment period will be maintained within the RTSM system and in secure locations by individuals who are not directly involved in the conduct of the study. This is limited to the unblinded statistician and unblinded programmer at the statistics vendor, the project management personnel at the drug product manufacturer, and project management/system build personnel at the RTSM system vendor.

The blind for an individual subject should not be broken during conduct of the study except in the case of a medical situation for which it is deemed essential to know which treatment the subject has received during the double-blind treatment period to provide appropriate care. If possible, discussion with the Sponsor should be sought before the blind is broken. In an emergency, the investigator may unblind a specific subject and determine the identity of treatment using the RTSM System. Instructions regarding treatment identification using the RTSM System will be available in separate guidance documents. In such circumstances, the Medical Monitor must be contacted and informed of any unblinding as soon as possible. The date, time, and reason for unblinding must be documented. In case of after-hours emergency

unblinding or in circumstances when the investigator is not available, a 24/7 emergency number (provided on patient cards) will be available to determine the identity of the treatment. This will be achieved *via* a "peek blind" function within the RTSM system, whereby an end user with appropriate access can view the unblinded treatment group on screen. Completion of the peek blind transaction will reinstate the blinded status of the subject.

If a subject is unblinded, study drug will be discontinued and the subject will be followed until resolution or stabilization of the event. He/she will then be discontinued from the study.

Information about any subject for whom an unblinding occurs will be provided to the DSMB by the Sponsor or designee within 15 days (within 7 days in the event of a fatal event). For requirements and unblinding procedures for suspected unexpected serious adverse event (SUSAR) reporting, refer to Section 16.8.

After the last subject completes the 52-week, double-blind, treatment period, that portion of the database will be locked and subject populations determined by blinded data review. The database will then be unblinded for purposes of performing primary and secondary efficacy analyses, and a report will be prepared (see Section 17.9); individual subject treatment assignment will not be revealed either to the subjects or personnel involved in the ongoing management and assessment of subjects in the open-label treatment phase.

13 CONCOMITANT MEDICATIONS AND SUBJECT RESTRICTIONS

All concomitant medications and medications administered within the past 90 days from Screening, as well as a lifetime history (as far as possible) of AChEI and/or memantine use will be recorded at the Screening visit (Visit 1). While in the clinic at Baseline (Visit 2; before and after the first dose of study drug is administered), any medications administered will be recorded. "Medication" is used to encompass prescription and over-the-counter drugs or biologics, vitamins used in supra-pharmacologic doses, alternative pharmacotherapies for dementia, medical foods, and for women, forms of contraception. At each subsequent scheduled visit (*i.e.*, Visits 3 to 7 during the double-blind treatment period, and Visits 8 to 10 during the open-label, delayed-start phase) or upon early termination, any changes to existing concomitant medication and any new concomitant medication will be reviewed and recorded. These must be reviewed by a medical assessor (physician/MD/DO).

Concomitant medications identified at Screening generally should be maintained at a constant dose for the duration of the study if clinically indicated. The investigator should evaluate any changes in the doses of existing concomitant medications and/or initiation of new concomitant medications, and the Medical Monitor should be contacted to discuss any concerns as needed. The date of commencement, dose, and date of any change of dose of concomitant medications are to be recorded in the eCRF.

Disallowed treatments and the time windows are listed in Section 28.1.

13.1 AChEI and/or Memantine

Subjects are not to be treated with an AChEI and/or memantine within the 60 days prior to the baseline assessments (inclusive of the baseline ^{18}F-FDG-PET scan in subjects who have a screening CDR of 0.5). If a subject decides to discontinue AD medication in order to enter

the study, a letter will be sent to his/her primary care physician informing him/her of the subject's decision. The withdrawal / discontinuation of AD medication for study inclusion should not occur until the subject has been confirmed eligible for the study.

If a subject begins an AChEI and/or memantine during the study, LMTM will be discontinued and LMTM will not be made available *via* the EAP. Subjects will be encouraged to continue with scheduled assessments if willing to do so without LMTM. Handling of data for such subjects is described in Section 17.4.11.3.

13.2 Drugs with Serotonergic Potential

Methylene blue (MTC) has been shown to be an inhibitor of monoamine oxidase (MAO) and to be associated with serotonin toxicity in conjunction with other serotonergic drugs (discussed in the Investigator's Brochure). Most cases have followed intravenous administration. Two cases have also been reported, one each following enteral and oral administration; however, neither case provides compelling evidence of MT-induced serotonin toxicity via these routes. There is a theoretical potential for serotonin toxicity following administration of oral LMTM alone, as well as a theoretical possibility of clinically significant drug interaction following co-administration of LMTM with a serotonergic medication. There have been four potential cases in the Phase 3 studies of LMTM; it is difficult to determine from the available information whether any represents a true case of serotonin toxicity.

There is no proscription against the use of serotonergic drugs; however, investigators should evaluate subjects for potential serotonin toxicity. For additional details regarding clinical findings from studies, refer to the Investigator's Brochure. A list of drugs with serotonergic potential will be provided separately.

13.3 CYP and P-gp Substrates

Results of a completed drug-drug interaction study using LMTM 450 mg/day indicate that LMTM is generally a weak inhibitor of CYP3A4, CYP2C8, and CYP2C19 enzymes (see the Investigator's Brochure for examples of drugs metabolized by these enzymes). The extent to which this occurs within an individual or with a given drug is not known, especially for those drugs with multiple metabolic pathways. Therefore, subjects on drugs known to be metabolized by one or more of these enzymes (especially those that have a narrow therapeutic index) should be closely monitored for AEs that could suggest an increase in systemic exposure. Dose adjustment of the concomitant medication may be warranted.

LMTM is also a weak inducer of CYP2B6 and P-gp transporter at concentrations relevant to higher doses (see the Investigator's Brochure for examples of substrates); the extent to which this might occur at LMTM doses of up to 16 mg/day is not known. Co-administration of LMTM with digoxin, a P-gp substrate, was shown to result in decreased concentrations of digoxin. Therefore, it is advisable to obtain a baseline digoxin level in subjects on this drug and to monitor digoxin levels while on study. Any such results obtained from the local laboratory should be entered into the eCRF.

13.4 Drugs Used to Manage Behavioral Disturbance

Subjects may be treated with antipsychotics (other than clozapine) provided they have been used in a stable dose and regimen for at least 90 days prior to Baseline. There should be no

intent to initiate such therapy during the course of the study. Should treatment be initiated, the reason(s) should be clearly documented by indicating one or more of the following reasons: delusions, hallucinations, agitation/aggression, depression, anxiety, elation/euphoria, apathy/indifference, disinhibition, irritability/lability, aberrant motor behavior, nighttime behavior, or appetite/eating change.

"As-needed" use of antipsychotics is to be avoided if possible, but such use does not preclude further participation. Similarly, regular or occasional use of benzodiazepines to manage distress, agitation, etc. does not preclude further participation, with the exception of prior to ^{18}F-FDG-PET scans to be obtained in subjects with CDR 0.5 at Screening only (the use of sedatives is not permitted within 24 hours prior to ^{18}F-FDG-PET scans). These must not be used within the 12 hours prior to cognitive testing.

13.5 Other Medications

The medical food and other medications listed in Exclusion Criterion No. 19 (see Section 11.2) are specifically prohibited during participation in this study.

Anxiolytics and/or sedatives/hypnotics may be used as sedation for claustrophobia or agitation or to manage excessive movement during MRI scans; however, use of sedatives is not permitted within 24 hours prior to ^{18}F-FDG-PET scans in subjects with CDR 0.5. Regular or occasional benzodiazepines, chloral hydrate, low dose trazodone (50 mg), or zolpidem may be used as needed at bedtime for sleep.

Unless otherwise prohibited, concomitant medications (preferably at stable doses) considered appropriate by the subject's physician are allowable but should be kept to the minimum possible as clinically indicated. If there are questions about whether or not a medication is permitted in the study, the Medical Monitor should be consulted.

13.6 Dietary Tyramine

Historically, MAO inhibitors as a class have been reported to be associated with hypertensive crises caused by ingestion of foods containing high amounts of tyramine (known as a tyramine or "cheese" reaction). While there is a theoretical potential for a tyramine reaction with MT, there have been no reports to date in subjects taking part in TauRx-sponsored studies, even though there have been no dietary restrictions in these studies. There have also been no effects on blood pressure. Nonetheless, as a precaution, subjects and their study partners (caregivers/informants) should be advised about this potential while taking LMTM (see the Investigator´s Brochure for examples of tyramine-rich foods and beverages, such as air-dried, aged or fermented meats, sausages and salamis; aged cheeses; fava bean pods; non-pasteurized beers; sauerkraut; and most soybean products). They should also be advised to seek medical care immediately in the event of signs or symptoms of hypertensive crisis (sudden onset of severe headache, nausea, stiff neck, tachycardia or palpitations, profuse sweating, and/or confusion) or other sudden or unusual symptoms following ingestion of tyramine-rich foods or beverages.

13.7 Contraceptive Measures

As a precautionary measure, women of childbearing potential (*i.e.*, not documented to be post-menopausal for at least 1 year or not having undergone hysterectomy or bilateral salpingectomy/oophorectomy for at least 6 months minimum), either must have undergone

bilateral tubal ligation or occlusion at least 6 months prior to Baseline or must use adequate contraception. Subjects must be competent to use adequate contraception and must agree to continue to maintain adequate contraceptive measures throughout study participation and for at least 4 weeks after the last dose of study drug. Examples of adequate contraception include:

- Use of a barrier method (condom, diaphragm or cervical/vault cap) with spermicidal foam, gel, film, cream, or suppository
- IUD or system
- Oral or long-acting injected or implanted hormonal contraceptives for at least 90 days prior to Baseline
- Sexual activity restricted to a vasectomized partner (with the appropriate post-vasectomy documentation of the absence of spermatozoa in the ejaculate).
- Abstinence is only acceptable as true abstinence when this is in line with the subject's preferred and usual lifestyle; periodic abstinence (*e.g.*, calendar, ovulation, symptothermal, and post-ovulation methods) and withdrawal are not acceptable methods of birth control.

Serum pregnancy testing will be performed at each scheduled study visit in women of childbearing potential. Such women should be encouraged to return to the clinic in the event of a delayed menstrual period to rule out possible pregnancy, during study conduct and for up to 3 months after the last dose of study drug. Alternative arrangements related to COVID-19 for pregnancy monitoring may be permitted and are described in Section 10.2.

The risk of drug secretion through the ejaculate is not fully studied. To ensure that the fetus is not exposed to MT through vaginal absorption, male subjects (including men who have had vasectomies) whose partners are pregnant should use condoms for the duration of the study and for an additional 10 days after cessation of study treatment. The investigator must provide appropriate counsel to male subjects regarding this issue.

13.8 Folate and Vitamin B_{12}

The manufacturer of the test kits used by the central laboratory for measuring folate and vitamin B_{12} has established the following normal ranges (healthy U.S. males and females aged 18 years and older): 5.9-24.8 ng/mL for folate and 180-914 pg/mL for vitamin B_{12}. However, these values are not applicable for all geographical areas as food in the United States is supplemented with these vitamins. Prior to initiating study drug, subjects with folate levels <4.0 ng/mL or vitamin B_{12} levels <150 pg/mL (*i.e.*, deficient according to WHO Technical Consultation, 2008, 2012), should be supplemented for the duration of the study. Subjects with folate levels <4.0 ng/mL may be entered into the study provided they are supplemented (approximately 1 mg/day folate). Subjects with vitamin B_{12} levels <150 pg/mL should be referred to their primary care physician for evaluation and treatment or the Medical Monitor consulted. There must be a treatment plan in place for any applicable chronic condition. If a condition is diagnosed that the primary care physician believes cannot be reliably or continuously corrected, the subject should be excluded from the study. If review and correction can be achieved within the Screening window, the subject may be entered into the study; otherwise the subject must be re-consented and re-screened after the deficit has been corrected.

14 TERMINATION OF THE STUDY

The Sponsor reserves the right to terminate the study for duly justified reasons in accordance with the national laws. These reasons include in particular:

- Administrative reasons: *e.g.*, financial reasons
- Interest of subject welfare: *e.g.*, new information or events that result in an unfavorable risk-benefit profile

Continued access to investigational study drug may be available as detailed in separate EAP Protocols or by other legally acceptable means.

15 STUDY ASSESSMENTS

15.1 Demographic Data/Medical History

The PI (or SI) must complete the NIA/AA diagnostic interview with the subject and study partner. The NIA/AA criteria should be used as a guide and the information recorded in the subject's medical records. The accuracy of the diagnosis will be confirmed independently by MedAvante-ProPhase (Section 10.5.1.1).

The investigator should collect the following demographic data: age of the subject at informed consent, gender, ethnicity, race, and geographic region.

General baseline characteristics that should be collected are height, weight, creatinine clearance, smoking history, and age at leaving full-time education. Further information indicating years spent in full-time education and its type before the age of 26 years is to be collected (see Section 28.3); also, main occupation during working life is to be provided as indicated.

Disease-specific baseline characteristics that should be collected are time from diagnosis of AD to informed consent (years); amyloid biomarker confirmation; MMSE; CDR; use of an AChEI and/or memantine (previous use or never used and if previous use, drug[s], how long used, when stopped, and reason for stopping); and use of other anti-dementia treatments or medical foods other than AChEIs or memantine. Medical history (including history of lens implantation) will be recorded.

Recently used medications (lifetime use of antidementia medications and within 90 days prior to Screening for other medications) will be recorded at Screening. Concomitantly used medications will be recorded throughout study participation.

15.2 Assessment of Efficacy

15.2.1 Raters

Efficacy instruments (ADAS-cog_{13}, ADCS-ADL_{23}, MMSE, and CDR) will be completed by assessors/raters who are not involved in the assessment of safety parameters that could result in unblinding. These will be administered using Virgil tablets in the local language. Efficacy assessments should be performed at approximately the same time of day throughout the study for a given subject, to the greatest extent possible. For a given subject, raters should remain constant throughout the study. Investigators and other raters will be trained and approved by

MedAvante-ProPhase; only individuals approved to complete each assessment will receive access via the Virgil System. Rater performance and consistency will be subject to surveillance throughout the study. One to two efficacy raters are required at each site (with back-ups as needed).

Information obtained from study partners providing data from assessments of the subject (*i.e.*, informants) will be appropriately identified to distinguish each informant in the event that a given subject has more than one study partner providing data for analysis (see Section 10.2 for a summary of alternate arrangements that may be implemented due to COVID-19).

A summary of the efficacy rater allocation and the order of efficacy assessments by visit is provided in Table 15-1.

Table 15-1: Efficacy Rater Allocation and Order of Assessments by Visit

Section 1. Rater Allocation

Assessments are made by suitably trained efficacy raters who are NOT involved in safety assessment.

It is expected that the SAME rater completes the efficacy assessments for a given subject throughout the study.

Raters should carry out assessments according to a specific allocation:

**Rater Allocation 1** (Preferred)		**Rater Allocation 2**		**Rater Allocation 3**	
Rater 1	MMSE	Rater 1	MMSE	Rater 1	MMSE
	CDR	Rater 2	CDR		CDR
Rater 2	ADAS-cog$_{13}$		ADAS-cog$_{13}$		ADAS-cog$_{13}$
	ADCS-ADL$_{23}$		ADCS-ADL$_{23}$		ADCS-ADL$_{23}$

Section 2. Order of Assessments

Assessments are to be carried out in the order specified. The order of assessment depends on the visit type.

**Visit**	**Order**	**Assessment**
Visit 1 Screening	1.	MMSE (Eligibility)
	2.	CDR (Eligibility)
Visit 2 Baseline	1.	ADAS-cog$_{13}$
	2.	ADCS-ADL$_{23}$
Visit 4, 5, 6	1.	ADAS-cog$_{13}$
	2.	ADCS-ADL$_{23}$
Visit 7 or Double-Blind ET (Also Baseline/Day 1 for Open-Label Phase)	1.	ADAS-cog$_{13}$
	2.	ADCS-ADL$_{23}$
	3.	CDR
	4.	MMSE
Visit 9 Open-Label, Delayed-Start	1.	ADAS-cog$_{13}$
	2.	ADCS-ADL$_{23}$
Visit 10 OL-EOT or OL-ET	1.	ADAS-cog$_{13}$
	2.	ADCS-ADL$_{23}$
	3.	CDR
	4.	MMSE

Abbreviations: ADAS-cog$_{13}$ = Alzheimer's Disease Assessment Scale – cognitive subscale (13-item); ADCS-ADL$_{23}$ = Alzheimer's Disease Cooperative Study – Activities of Daily Living (23-item); CDR = Clinical Dementia Rating; ET = early termination; MMSE = Mini-Mental State Examination; OL-EOT = open label end of treatment; OL-ET = open-label early termination

15.2.2 Instruments

The main efficacy instruments include the following: ADAS-cog$_{13}$ (cognition) and ADCS-ADL$_{23}$ (activities of daily living). From these, Composite Scores will be derived as an exploratory endpoint: at least one that is designed to be sensitive to early stages of the disease as well as one that is less/not impacted by COVID-19 (all Composite Scores will be defined in the SAP prior to sign off and database lock/unblinding). The MMSE and CDR (total and sums of boxes) are rated as secondary instruments. All are described briefly below.

For a given subject, the ADAS-cog$_{13}$ and ADCS-ADL$_{23}$ will be completed by Efficacy Raters, in the local language. The Efficacy Raters must <u>not</u> also elicit, record, or enter data

relating to subject safety. The instruments will be administered *via* an electronic Virgil tablet provided by MedAvante-ProPhase, which will also capture an audio recording of the assessment. Alternative arrangements that may be implemented for conducting these instruments for post-Baseline visits directly impacted by COVID-19 are summarized in Section 10.2.

Each time an efficacy instrument is administered, the person administering it will be documented within the electronic form. Efficacy Raters will be trained and their proficiency in administering applicable efficacy instruments will be confirmed. Raters must be approved by MedAvante-ProPhase prior to administering assessments. A unique identifier and password will be provided to each rater that completes and passes the rater training for applicable assessments. Raters must enter their unique password to commence a subject assessment, and again when they upload the data to verify the identity of the rater.

The MMSE and CDR questionnaires and the ADAS-cog_{13} and ADCS-ADL_{23} will be assessed in the native language by an independent Clinical Reviewer at MedAvante-ProPhase by reference to audio recordings of the assessments. The objective of these independent reviews is to ensure raters adhere to the administration and scoring conventions detailed in rater training. Where necessary, raters will be requested to review their performance and confirm the scoring. For eligibility assessments (screening MMSE and CDR), adjudication will occur for any subject where assessments fall outside of the inclusion criteria. Once efficacy assessments are completed and uploaded, raters should review and discuss with MedAvante-ProPhase and receive approval prior to making any proposed change in scoring. A Diagnostic Verification Form (on the Virgil Tablet) completed by the PI (or SI) at Screening will be reviewed by an independent Clinical Reviewer at MedAvante-ProPhase prior to subjects proceeding to imaging assessments to ensure that subjects meet those elements of the inclusion criteria that relate to clinical diagnosis of probable AD or MCI-AD.

Efficacy assessments should be performed at approximately the same time of day throughout the study for a given subject, to the greatest extent possible. The clinical efficacy assessments should be administered before vital signs measurement and blood sample collection. All attempts should be made to have the same rater collect efficacy outcome data at all visits. The key efficacy scales are described below.

15.2.2.1 ADAS-cog

The ADAS was designed to evaluate the severity of cognitive and non-cognitive or behavioral symptoms of AD (Rosen *et al.*, 1984). The ADAS-cog is the cognitive subscale of the ADAS, originally proposed with 11 items. The ADAS-cog_{11} consists of tasks that measure memory, orientation, language, and praxis resulting in scores that range from 0 to 70, with higher numbers indicating greater impairment. Two additional items have been added in the ADAS-cog_{13} to provide additional sensitivity to change in cognition at earlier stages of the disease (Mohs *et al.*, 1997); these are Delayed Word Recall and Number Cancellation.

As the original ADAS-cog_{11} was used in the earlier Phase 3 studies, that score will be derived from the assessment of the ADAS-cog_{13} for the primary analyses. The Composite Scale described in Section 15.2.2.3 will be based on items selected from the ADAS-cog_{13}.

15.2.2.2 ADCS-ADL$_{23}$

The ADCS-ADL$_{23}$ includes 23 items that were derived from a larger set of items describing performance of activities of daily living (ADL) by AD patients (Galasko *et al*., 1997). Of 45 ADL items originally evaluated during the creation of this instrument, the 23 items showed good test-retest reliability over 1 to 2 months (Galasko *et al*., 2005). Scores of these items (ADCS-ADL$_{23}$) were also shown to correlate with MMSE scores of AD patients and to decline over 12 months in at least 20% of AD patients. The Composite Scale described in Section 15.2.2.3 will also be based on selected items from the ADCS-ADL$_{23.}$

15.2.2.3 Composite Scale(s)

The new Composite Scale based on selected items of the ADAS-cog$_{13}$ and the ADCS-ADL$_{23}$ will be analyzed at 9 months and 12 months during the double-blind treatment period to evaluate its usefulness for future studies. It consists of cognitive subdomains (orientation [8], constructional praxis [5], word recall, third trial [10], assessor rating of subject speech [5], and assessor rating of subject comprehension [5]) from the standard ADAS-cog$_{11}$, and functional items (use of telephone [5], keeping appointments [3], cooking and preparation of meals [4], and cleaning dishes [3]) from the standard ADCS-ADL$_{23}$. The selected cognitive items are based on a non-interventional study (Study TRx-GTD-025) examining performance of a range of psychometric tools which decline over 12 months in discriminating subjects with mild AD from age-matched healthy controls, supplemented with functional items identified as declining over 9 months in add-on treatment arms in Study TRx-237-005, independently confirmed by similar data from TRx-237-015, and supported by ADNI data. The items listed provide a maximum possible score of 48 (higher score indicates less impairment).

Additional Composite Endpoints (if any) such as one that is less/not impacted by COVID-19 will be defined in the SAP and signed off prior to database lock and unblinding.

15.2.2.4 Mini-Mental State Examination (MMSE)

The MMSE (Folstein et al., 1975) was originally developed to differentiate between psychiatric patients with functional and organic conditions, to quantify the level of cognitive impairment, and to monitor changes over time. The MMSE subsequently has become a widely used and extensively validated cognitive test demonstrating satisfactory reliability, validity, and change sensitivity under a wide variety of conditions (Tombaugh and McIntyre, 1992). A modified version of the form, supplied by Psychological Assessment Resources, is used in this study.

The utility of the MMSE as a means of assessing treatment response in AD has been questioned (Bowie *et al*., 1999), but its status as a clinical outcome measure has been supported by the UK National Institute for Clinical Excellence guidance (NICE, 2001). Furthermore, the MMSE has demonstrated an ability to detect change in clinical studies with AChEIs (Birks and Harvey, 2004). Further, in an epidemiological study (Mukaetova-Ladinska *et al*., 2000), pre-mortem MMSE scores have been correlated with post-mortem Braak stage (based on the spread of tau pathology through the brain).

15.2.2.5 Clinical Dementia Rating (CDR)

The CDR rates overall severity of dementia (Hughes *et al*., 1982). The CDR has been shown to have good reliability (Morris *et al*., 1997) and validity (Fillenbaum *et al*., 1996) and to correlate with neuropathological status (Morris *et al*., 1988). The scale is sensitive to change over longer time periods (Berg *et al*., 1988).

The CDR is administered using a worksheet and semi-structured interview of a reliable informant (*e.g*., caregiver) followed by an interview of the subject to assess the following six domains: memory, orientation, judgment and problem solving, community affairs, home and hobbies, and personal care. Each domain is scored on a 5-point scale, where 0 = normal, 0.5 = questionable impairment, 1 = mild impairment, 2 = moderate impairment, and 3 = severe impairment. The CDR total score (also sometimes referred to as the CDR global score) is derived from individual ("box") scores for each of the six domains, in accordance with published scoring rules.

15.3 Imaging Assessments and Procedures

15.3.1 General Considerations

PET imaging using an amyloid ligand and brain MRI play a role in this protocol for eligibility determination of the subjects; brain MRI is also used as a secondary efficacy endpoint as well as to confirm a lack of treatment unblinding. ^{18}F-FDG-PET will be performed in subjects with CDR 0.5 at Screening as a secondary efficacy endpoint at all study sites, and will be assessed by an independent nuclear physician who is trained and not otherwise involved in the clinical conduct of the study. The independent central imaging core laboratories will be responsible for image collection and storing, including, with the subject's (and/or LAR's) consent, collection of amyloid PET scan performed either in the course of the study or previously. The total radiation exposure to subjects from the amyloid PET scan procedure (if not performed previously) is estimated to be 9 mSv for a single scan; the limits for radiation exposure from the ^{18}F-FDG-PET scans are discussed in Section 15.3.3.1.

With respect to ^{18}F-FDG-PET and MRI, the core laboratories will be responsible for checking the quality of imaging data, verifying that site anonymization of the images is maintained, pre-processing of images, presenting data to the reader(s) (for MRI eligibility and volumetric evaluations and ^{18}F-FDG-PET), and analyzing the data. Each core laboratory will provide SAS datasets to the data management and statistics facilities for analysis. All systems and processes used for independent and central reads of this trial will be 21 CFR Part 11 compliant.

Before commencement of central evaluations, vendor study-specific documents such as charters and manuals will be developed that will describe in detail the imaging acquisition protocols, image collection procedures, quality check procedures, site training procedures, reader training, image evaluation procedures (central determination of subjects' eligibility, efficacy, and safety), and communication plans.

All sites will be prospectively trained about imaging requirements including scanner requirements, image acquisition, image transfer to the core laboratories, and timelines that are critical for this trial.

See Section 10.2 for a summary of alternate arrangements that may be implemented due to COVID-19.

15.3.2 Site Selection and Qualification

A site imaging technical evaluation questionnaire will be distributed to potential clinical sites to evaluate their technical and personnel capabilities that will include machine description, availability of phantoms (if applicable), onsite availability of modality-specific technologist or physicist (depending on ^{18}F-FDG-PET or MRI facility), site experience in evaluating brain ^{18}F-FDG-PET and/or MRI, experience in AD and other dementia trials, *etc.* The site's capability of producing quality data that are necessary for this trial will also be evaluated by appropriate phantom imaging and/or review of imaging data of the site's first subject. Continuous monitoring of the quality of imaging data will be performed throughout the trial. Technical details of imaging and quality check procedures will be described in a separate imaging manual.

15.3.3 Imaging Methods for Efficacy

A separate imaging manual will be developed that will outline imaging methods for both ^{18}F-FDG-PET and brain MRI to harmonize image acquisition across the sites. ^{18}F-FDG-PET should be obtained with computerized tomography (CT); brain-dedicated high-resolution PET devices, such as the Siemens High Resolution Research Tomograph system, that use a transmission source for attenuation correction will also be considered from expert sites.

15.3.3.1 ^{18}F-FDG-PET

An ^{18}F-FDG-PET scan will be performed in subjects with CDR 0.5 at Screening using PET/CT or a brain-dedicated high-resolution PET device.

Use of sedatives is <u>not</u> permitted for a period of 24 hours prior to ^{18}F-FDG-PET scans. The subject's fasting (at least 4 to 6 hours) blood glucose level, carried out by the imaging center's standard operating procedures, should be <180 mg/dL (<9.9 mmol/L) in order for the injection to take place. If a screening amyloid scan is acquired under the study, there must be a minimum of 16 hours between the amyloid scan and the ^{18}F-FDG-PET scan due to the half-life of fluorine.

The baseline ^{18}F-FDG-PET scan is to be performed within 42 days before Baseline (Visit 2) only in subjects confirmed to meet all inclusion and exclusion criteria and who have a screening CDR of 0.5. Subjects who were previously treated with an AChEI and/or memantine must have received the last dose at least 60 days prior to this scan. If the initial screening/baseline ^{18}F-FDG-PET is not of sufficient quality to serve as an adequate baseline as determined by the PET Imaging Core Lab, then the scan must be repeated within 42 days before Visit 2 as long as the repeat PET does not cause radiation exposure to the subject that exceeds the limits for the clinical trial (approximately 4.7 mSv for a single scan and 14.1 mSv in total, if a repeat scan is deemed necessary). If the repeat scan cannot be accomplished within the 42-day window before Baseline, the subject must be re-consented and re-screened. For subjects who are re-screened for other reasons and an acceptable ^{18}F-FDG-PET scan was already completed during the original Screening window, the scan does not require repetition.

The Week 52 (end of double-blind treatment) ^{18}F-FDG-PET scan should be performed within ± 14 days of the designated visit. In the event of early termination, if the subject's last ^{18}F-FDG-PET scan was performed ≥30 days prior to that date, the ^{18}F-FDG-PET scan must be done as part of the early termination visit assessments within the time window of the early termination visit (*i.e.*, ± 14 days of the last dose of study drug). The allowable time window for the early termination ^{18}F-FDG-PET scan can be extended to up to 28 days after the last dose of study drug in the double-blind treatment period only with the permission of the Sponsor/Medical Monitor.

Imaging assessments will be made by central readers as follows:

- Brain ^{18}F-FDG-PET will be evaluated for temporal, parietal, and frontal lobe SUVR (normalized with respect to pons and cerebellum) at Baseline and after 52 weeks of double-blind treatment, or upon early termination. Other SUVR ROIs will also be examined including, but not restricted to, inferior temporal gyrus, angular gyrus, anterior and posterior cingulate gyrus, and cerebellum. Change in SUVR parameters will be quantified by the imaging core laboratory.
- SPM preprocessing may be performed, which involves realignment of the dynamic ^{18}F-FDG-PET images, the summation of those images, registration to the MR images in native space, normalization to atlas space, count normalization to a reference region, and smoothing for statistical purposes. After database lock, the voxelwise change in the SUVR maps may be quantified using an SPM approach (models to be characterized in the SAP). Details of analysis conducted will be reported in a separate report.

15.3.3.2 MRI

The screening MRI (to be obtained within the 9-week Screening period [Days -63 to -1] in subjects not receiving an AChEI and/or memantine, or Days -112 to -63 in subjects who begin Screening on an AChEI and/or memantine) will also be used as the baseline for volumetric measurements. The volumetric MRI will be repeated approximately every 13 weeks in the double-blind treatment period after randomization (*i.e.*, after 13, 26, 39, and 52 weeks) as well as after the additional 26 and 52 weeks of open-label treatment. For subjects who terminate early (in either the double-blind treatment or open-label treatment periods), if the subject's last MRI scan was performed <90 days prior to the early termination date, no additional MRI scan is required.

Imaging assessments will be made by central readers as follows:

- Brain MRI will be evaluated for temporal and parietal, whole brain, lateral ventricular, hippocampal, putamen, nucleus accumbens, and nucleus basalis ROI volumes. Change in MRI volumetric parameters will be quantified by the imaging core laboratory.
- T2*-weighted gradient-recalled echo is to be included to enable the detection and quantification of the image hyperintensities.
- SPM preprocessingmay be performed, which involves registration of the MR images to atlas space, segmentation into tissue types (grey, white, cerebral spinal fluid). After database lock, the voxelwise change may be quantified in the segmented MR maps using an SPM approach (models to be characterized in the SAP). Details of analysis conducted will be reported in a separate report.

15.4 Safety Assessments and Procedures

Safety assessments will be performed during Screening to assess subject eligibility for enrollment. All safety assessments will be performed by an independent qualified assessor not involved in efficacy assessments; where specified below, the assessments must be made by a medical assessor (physician/MD/DO). For enrolled subjects, safety assessments will be made at Baseline and at each clinic visit (*i.e.*, after 4, 13, 26, 39, and 52 weeks during the double-blind treatment period as well as after 26 and 52 weeks in the open-label, delayed-start phase); during a telephone contact after 4 weeks in the open-label, delayed-start phase; when needed to follow up on an AE; and upon early termination. See Section 10.2 for a summary of alternate arrangements that may be implemented due to COVID-19. All AEs, vital signs, ECG, clinical laboratory findings, physical and neurological examinations, and ophthalmological examinations (as applicable) will be assessed according to the following:

- AEs will be recorded from the time informed consent is signed and recording will continue throughout the study. The recording and assessment of AEs will be performed by a medical assessor (physician/MD/DO) separate from those administering the efficacy outcome scales (ADAS-cog_{13} and ADCS-ADL_{23} as well as the CDR and MMSE). AEs with an onset after the first dose of study drug or that worsen in intensity or treatment relationship after the first dose will be considered treatment-emergent (and must be reviewed by a medical assessor [physician/MD/DO]). Assessment should continue as needed to follow up an AE to its resolution or acceptable stabilization (including after the last dose of study drug), consistent with the medical judgment of the investigator.
- Blood pressure and pulse will be obtained at Screening, on Day 1 (within 1 hour pre-dose and approximately 2 hours post-dose), and at each clinic visit thereafter or upon early termination. Blood pressure and pulse will be measured after the subject has been in a seated position for at least 5 minutes.
- Height will be measured at Screening only. Body weight will be measured at Screening and at each clinic visit thereafter or upon early termination.
- A 12-lead ECG will be obtained at Screening, with the site judging eligibility based on the local interpretation by a medical assessor (physician/MD/DO).
- TSH, vitamin B_{12}, folate, haptoglobin, and G6PD will be measured at Screening; a thyroid panel may be obtained in response to an elevated TSH. Standard clinical laboratory testing, including hematology and blood chemistry, will be performed at Screening and each subsequent clinic visit (or upon early termination). Testing may also be performed at unscheduled visits as necessary to follow-up an AE (see Section 16.11.1 for testing in response to possible hemolytic anemia). All laboratory results are to be reviewed by a medical assessor (physician/MD/DO). Any abnormal laboratory test result from Screening assessments (prior to treatment) is to be added to the subject's medical history, unless deemed clinically significant by the medical assessor (MD/DO) in which case it will be recorded as an AE.
- A blood sample for a serum pregnancy test will be collected from all women of childbearing potential at Screening and at each subsequent clinic visit (or upon early termination).
- Complete physical and neurological assessments will be performed at Screening (see Section 10.5.1.2). Targeted examinations will be performed pre-dose and approximately 3 hours after administration of the first dose of study drug (Visit 2). Thereafter, targeted examinations are to be performed at each subsequent clinic visit (upon early termination). At a minimum, targeted examinations should include heart and lung auscultation and brief neurological assessment guided by any reported

signs/symptoms/AEs (*e.g.*, evaluating subjects for potential serotonin toxicity). These examinations are to be performed by a medical assessor (physician/MD/DO).

- Slit lamp ophthalmological examination of subjects with history of lens implants will be performed by a licensed optometrist, ophthalmologist, or other suitably qualified medical assessor (physician/MD/DO) prior to the first dose of study drug (during the screening procedures or as part of the baseline assessments), at Visit 7, and at Visit 10 / early termination (after completion of efficacy assessments), to assess whether the lens has been discolored during the trial. The slit lamp examination should also be performed if a subject has cataract surgery / lens implantation at any point during his or her study participation (as soon as possible after the surgery), as well as in response to visual complaints if suggestive of lens discoloration.
- At Screening, medications administered within the last 90 days will be recorded, with the exception of anti-dementia medications, where lifetime use (as far as possible) is to be recorded. Changes in concomitant medications and any new concomitant medications will be recorded at all visits, including the telephone contact during the open-label, delayed-start phase, and reviewed by a medical assessor (physician/MD/DO). For antipsychotics, the reason for use should be documented (see Section 13.4).

Further details regarding alternate arrangements for sample collection and shipment that may be implemented due to COVID-19 are described in Section 10.2.

15.5 Other Assessments

15.5.1 MT Concentration

At Visits 2, 3, 7, and 10, blood will be collected on the same day on three occasions for determination of plasma and whole blood MT concentrations (to the extent possible, parent MT/LMT and *N*-desmethyl MT, and after the sample has had acid and heat treatment, total MT); these visits should be scheduled to occur in the morning. Samples will be collected prior to dosing (in the clinic), approximately 1 to 2 hours post-dose, and approximately 4 hours post-dose. The time of the dose given in the clinic and the time of the blood sample will be recorded. In addition, at Visits 3, 7, and 10, the time of the prior dose taken on the preceding day at home will be recorded.

At any early termination visit, if the subject has not yet discontinued study drug and is willing to take a final in-clinic dose, three blood samples will be collected as described above. If, however, the subject has already discontinued study drug or is unwilling to take a final in-clinic dose, a single blood sample only for determination of MT concentrations will be collected, irrespective of the date or time of the last dose of study drug. The date and time of the last dose must be collected and recorded.

For subjects who continue in the study off-treatment (TOTOS group), blood samples will not continue to be collected for the determination of MT concentrations.

Concentration results will not be made available to the study sites during the conduct of the study. However, results for a given subject (if available) may be provided to the DSMB if requested to aid in interpretation of a significant subject safety issue.

The collection, handling, and shipping of blood samples are described below. These are to be analyzed using validated analytical methods.

See Section 10.2 for a summary of alternate arrangements that may be implemented due to COVID-19.

15.5.1.1 Procedure for Blood Sample Collection

Blood samples (9 mL) will be collected into a suitable vacutainer (9.0 mL), as defined in the laboratory manual. A portion of the whole blood will be centrifuged under refrigeration; the separated plasma will be transferred as defined in the laboratory manual. Both the whole blood aliquot and separated plasma will be stored at *ca* -20°C until shipment to the central laboratory for storage. The complete sample collection and handling procedures to be followed can be found in the laboratory manual.

15.5.1.2 Packaging, Labeling, and Shipping of Blood Samples

The samples must be labeled with unique identification numbers; other labeling information will be detailed in the laboratory manual. Labels must remain intact and indelible throughout processing and frozen storage. Samples will be clearly distinguished from the other bioanalytical samples. Samples are to be transported in insulated containers filled with dry ice. They will be shipped to the central laboratory where they will be stored frozen and shipped in batches to the analytical laboratory.

15.5.2 Genotyping

A single blood sample will be obtained only from subjects who provide legally acceptable informed consent for genotyping evaluation of *ApoE*. The blood sample may be collected any time after eligibility for randomization and continued participation in the study has been confirmed but prior to Visit 7 (end of double-blind treatment period). A volume of approximately 2 mL is to be collected and shipped ambient to the central laboratory (Labcorp) on the day of collection. See Section 10.2 for a summary of alternate arrangements that may be implemented due to COVID-19.

Genotyping results will not be provided to the study sites or to subjects.

16 ADVERSE EVENTS AND SAFETY

16.1 Definition of AEs, Period of Observation, and Recording of AEs

An AE is any unfavorable or unintended sign, symptom, or disease, whether or not considered related to the study treatment. This also includes events resulting from medication error or inappropriate use. AE recording will begin at the time the informed consent form (ICF) is signed. Thereafter, AEs will be ascertained by asking the subject (and study partner) how the subject has been since the last visit. A clinical abnormality, laboratory test value abnormality, or imaging abnormality that the investigator deems to be clinically significant should be recorded as an AE. Any abnormal laboratory test result from Screening assessments (prior to treatment) is to be added to the subject's medical history, unless deemed clinically significant by the medical assessor (MD/DO) in which case it will be reported as an AE.

Every attempt should be made to describe the AE in terms of a diagnosis. Once a clear diagnosis has been made, individual signs and symptoms shall not be recorded unless they represent atypical or extreme manifestations of the diagnosis, in which case they should be reported as separate events. Events leading up to a diagnosis should be retained. If a clear diagnosis cannot be established, each sign and symptom must be recorded individually.

All AEs must be fully recorded in the source documents and in the eCRF, regardless of whether or not the event is considered related to study drug.

New AEs reported by a subject after screen failure will not be recorded for the study. Emergent AEs up to the point of screen failure will be followed up to resolution at the discretion of the PI.

16.2 Eliciting Adverse Event Information

For all AEs, the investigator must pursue and obtain information adequate both to determine the outcome of the AE and to assess whether it meets one or more of the criteria for classification as an SAE or pregnancy requiring immediate notification.

As a part of evaluating subjects for potential AEs, investigators should ask one or more questions directed to evaluating suicidal ideation and behavior. Such questions are to be part of the routine evaluation of AEs at each in-clinic visit, addressed both to subjects and to their study partners.

Follow-up of an AE, even after the final dose of study drug, is required if the AE or its sequelae persist. Follow-up should be continued beyond the scheduled final visit if needed, until the AE or its sequelae resolve or stabilize at a level acceptable to the investigator in his / her medical judgement.

For each AE, information recorded will include the following: the date when the AE started, the date when the AE stopped (or whether it remained ongoing), the intensity of the AE, the relationship of the AE to study drug, action taken with regard to study drug (none, interrupted, or discontinued), other drug therapy (no change, new medication, altered medication, or both of the latter), outcome, and whether or not the AE was considered an SAE.

16.3 Categorizing Intensity

The intensity (severity) of each AE will be assessed by the investigator and graded as mild, moderate, or severe, as follows:

- Mild: An AE that is easily tolerated by the subject, causes minimal discomfort and does not interfere with everyday activities
- Moderate: An AE that is sufficiently discomforting to interfere with normal everyday activities
- Severe: An AE that prevents normal everyday activities
  An AE that is assessed as severe should not be confused with an SAE. Severity is a category utilized for rating the intensity of an event; and both AEs and SAEs can be assessed as severe.

16.4 Investigator's Brochure

The Investigator's Brochure will be used as a guide to assess causality.

16.5 Assessing Causality

The investigator will make a judgement considering whether or not, in his or her opinion, each AE is related to the study drug according to classifications described here. However, even if the investigator feels that there is no relationship to the study drug, the AE should be reported nevertheless. For each AE, the relationship or association (causality) of the AE to study drug will be assessed by the investigator and characterized as not related, unlikely related, possibly related, or related as follows:

- Not related: If there is a confirmed cause of the AE (other medical condition, other therapy) which does not involve the study drug
- Unlikely related: If the temporal association between the AE and the study drug is such that the AE is not likely to be related to the study drug
- Possibly related: If the AE shows a reasonable temporal association to study drug administration but could be due to the subject's clinical state or other therapies administered
- Related: If the AE shows a reasonable temporal association to study drug administration and cannot be explained by the known characteristics of the subject's clinical state

16.6 Categorizing the Outcome

Outcome describes the status of the AE. The investigator will provide information regarding the subject outcome of each AE.

Note: While negligible to slight coloration of urine or feces may occur as a result of study drug, these manifestations should not be recorded as AEs.

Definitions for possible results of an AE outcome are:

- Recovered/resolved: the event has improved or the subject recuperated
- Recovering/resolving: the event is improving
- Not recovered/not resolved: the event has not improved or the subject has not recuperated
- Recovered/resolved with sequelae: the subject recuperated but retained pathological conditions directly resulting from the disease or injury
- Fatal: termination of life as a result of an AE
- Unknown: not known, not observed, not recorded, or refused

16.7 Serious Adverse Event Reporting

An SAE is defined as any event that:

- Results in death (including suicide)
- Is life-threatening
- Results in hospitalization or prolongation of existing inpatient hospitalization
  - Planned admissions for respite care are not to be considered an SAE (the Medical Monitor should be contacted for confirmation regarding whether or not an admission for respite care should be considered planned or unplanned).

Unplanned admissions for respite care will constitute an SAE unless it is as a result of study partner needs that are independent of the subject's condition.

- o An admission or prolongation of existing hospitalization because the subject does not want to be discharged, or because the study partner is unable or unwilling to care for the subject, is not to be considered an SAE.
- o Admissions to a hospital that were planned or anticipated before the start of the study for an unrelated pre-existing medical condition are not to be considered an SAE.

- Results in persistent or significant disability or incapacity
- Is a congenital anomaly or birth defect
- Important medical events that might not be immediately life-threatening or result in death or hospitalization but might jeopardize the subject or might require intervention to prevent one of the other outcomes listed above. Examples of such events are intensive treatment in an emergency room or at home for allergic bronchospasm, blood dyscrasias or convulsions that do not result in hospitalization, or development of dependency or abuse

Medical and scientific judgment should be exercised in deciding whether an event is serious and whether expedited reporting is appropriate in other situations, such as important medical events that may not be immediately life-threatening or result in death or hospitalization but may jeopardize the subject or may require intervention to prevent one of the outcomes listed in the definitions above.

All SAEs must be reported on the eCRF. An assessment should be made by the investigator as to whether the event is study drug-related, *i.e.*, is ‘causally’ related to the study drug.

All SAEs (including any SAEs associated with COVID-19 that occur during study participation) should be reported to the Sponsor designee, Syneos Health, immediately (and not exceeding 24 hours following knowledge of the event). These should be followed by follow-up reports as soon as possible, whether or not the events are deemed study drug-related.

SAEs must be reported by entering the SAE information in the AE/SAE Section of the eCRF system. The information provided in the eCRF system should be as complete as possible, but must contain the following minimum fields:

- Subject number
- Brief description of the SAE (diagnosis or signs/symptoms)
- Serious criteria
- Causality assessment
- Assessment of the intensity of the event

Syneos Health Drug Safety will receive notification of the initial SAE *via* an e-mail alert generated from the eCRF system. In the event of any temporary disruption of the electronic system, an alternative SAE reporting mechanism will be available to site personnel; in this instance, a paper SAE report form will be available. Site personnel will complete the paper SAE report form, scan, and e-mail it within 24 hours to the following e-mail address:

safetyreporting@syneoshealth.com
Fax: +1 877 464 7787
Address:

Syneos Health
Safety/Pharmacovigilance Department
Farnborough Business Park – 1 Pinehurst Road,
Farnborough,
Hampshire, GU14 7BF
United Kingdom

SAEs that are ongoing should be followed until resolved or stabilized to a level acceptable to the investigator.

SAEs reported after screen failure will not be recorded for the study. Emergent SAEs up to the point of screen failure will be followed up to resolution at the discretion of the PI.

The investigator is obliged to provide additional information as requested by the Medical Monitor. In general, this will include a description of the event in sufficient detail to allow for a complete medical assessment of the case and independent determination of possible causality. In the case of a subject death, a summary of available autopsy findings, if performed, must be submitted as soon as possible to Syneos Health. However, any supporting information provided should not reveal a subject's identity beyond the agreed study identifier. The investigator should ensure that information reported is accurate and consistent.

Information not available at the time of the initial report (*e.g.*, an end date for the AE, laboratory values received after the report, or hospital discharge summary) must be documented on a follow-up form. All follow-up information must be reported in the same timelines as initial information.

Any SAEs considered related to the study drug and discovered by the investigator after the study should be communicated in writing to the Sponsor within 24 hours following knowledge of the event.

16.8 Suspected, Unexpected Serious Adverse Reactions and Emerging Safety Information

Suspected, unexpected serious adverse reactions are AEs that are believed to be related to an investigational medicinal product and are both unexpected (*i.e.*, the nature or severity is not expected from the information provided in the Investigator's Brochure) and serious. As stated in the EU 'CT-3' Communication from the Commission (2011/C 172/01) and the US Code of Federal Regulations (21 CFR 312.32), for there to be a reasonable possibility of a causal relationship between the event and study drug there must be facts (evidence) or arguments to suggest a causal relationship. Final assessment of expectedness for purposes of regulatory reporting is the responsibility of the Sponsor.

It is the responsibility of the Sponsor to determine whether a reported SAE fits the classification of a SUSAR and to notify the investigator of the decision as soon as possible.

All SUSAR reporting, whether determined following unblinding during study conduct or apparent only after the study has ended, will adhere to European Directives 2001/20/EC, 21 CFR 312.32 of the U.S. Code of Federal Regulations, Health Canada Food and Drug Regulation C.05.014, and other regions as applicable.

16.9 Malignancies

All cases of malignancies, other than non-melanoma skin cancers, are to be reported to the Sponsor designee, Syneos Health, as outlined above in Section 16.7. Adverse events of malignancies other than non-melanoma skin cancers are to be reported regardless of causality or whether they meet the criteria for serious. These will be reported to Health Canada as Adverse Drug Reactions (ADRs). Expedited reporting in other countries will be consistent with the handling of SUSARs.

16.10 Reporting of Pregnancy

Pregnancy is to be considered an immediately reportable event. This includes pregnancy of a female subject or a female sexual partner of a male subject. An event of a pregnancy will be reported to the Sponsor designee, Syneos Health, immediately (and not exceeding 24 hours following knowledge of the event), and followed by follow-up reports as soon as possible.

Subjects who become pregnant during the clinical study should discontinue study drug immediately and contact the investigator. Subjects should be instructed to notify the investigator of a pregnancy either during the Treatment period of the study or within 90 days after the last dose of study drug. Whenever possible, a pregnancy should be followed to term, any premature terminations reported, and the status of the mother and child reported to the Sponsor after delivery.

Although the pregnancy is not considered to be an AE or SAE, any pregnancy complications should be recorded as AEs or SAEs (if applicable). Any pregnancy should be followed through delivery for observation of any SAE, if possible. Therefore, regardless of whether or not a pregnancy is actually considered an SAE, a pregnancy form should be completed for all pregnancies.

The information provided in the eCRF should be as complete as possible, but must contain the following minimum fields:

- Subject number
- Confirmation that the subject’s contact information is on file at the site
- Date of report of pregnancy
- Expected delivery date

All data related to pregnancy, pregnancy outcome, and SAEs associated with pregnancy should be recorded in a safety database maintained by personnel responsible for pharmacovigilance at Syneos Health. Pregnancies should initially be reported in the Pregnancy Notification Form, Part I and sent by e-mail to the e-mail address provided below. When the outcome of the pregnancy is known, site personnel will complete the Pregnancy Notification Form, Part II and e-mail it to the same e-mail address noted below.

Syneos Health Drug Safety will receive notification of the pregnancy *via* an e-mail alert generated from the eCRF system. In the event of any temporary disruption of the electronic system, site personnel will scan and e-mail a written report within 24 hours to the following e-mail address:

safetyreporting@syneoshealth.com
Fax: +1 877 464 7787
Address:

Syneos Health
Safety/Pharmacovigilance Department
Farnborough Business Park – 1 Pinehurst Road,
Farnborough,
Hampshire, GU14 7BF
United Kingdom

16.11 Guidance for the Handling of Adverse Events of Special Interest and Selected Test Abnormalities

The pre-specified AESIs in this study are hemolytic anemia and lens discoloration. Guidelines for the monitoring and management of the AESIs are given below along with selected test abnormalities. Clinical circumstances requiring additional evaluation of a subject and/or potential interruption or discontinuation of study drug are presented.

16.11.1 Hemolytic Anemia

When there are signs and/or symptoms of clinically evident hemolytic anemia, dosing should be interrupted. A hemolysis panel should be considered (including complete blood count with differential, reticulocyte count [including absolute reticulocyte number], lactate dehydrogenase (LDH), direct and indirect bilirubin, and haptoglobin). See Section 13.8 for guidance on vitamin B_{12}. If confirmed as related to study drug, dosing should be discontinued.

Signs of possible treatment-emergent hemolytic anemia include:

- Decrease by 20% from screening in red blood cell (RBC) count and/or hemoglobin
- Abnormal RBCs in peripheral blood smear
- Elevation of reticulocyte count to above laboratory normal range
- Increase in LDH or indirect bilirubin, or lower haptoglobin than screening

16.11.2 Lens Discoloration

For subjects with a history of lens implants, ophthalmological examinations (slit lamp examinations) will be performed prior to the first dose of study drug (during Screening or as part of Baseline assessments), at Visit 7, and at Visit 10 / early termination as scheduled procedures. Subjects and investigators are informed of the potential for lens discoloration in the Informed Consent Form and Investigator’s Brochure, respectively. If a lens discoloration is identified by slit lamp examination, this is to be recorded as an AESI in the AE eCRF; however, no specific action is required for the study drug. Slit lamp examination should also be performed if a subject has cataract surgery / lens implantation at any point during his or her study participation (as soon as possible after the surgery), as well as in response to visual complaints if suggestive of lens discoloration.

16.11.3 Other Safety Reasons Requiring Discontinuation of Study Drug

If the calculated creatinine clearance is <30 mL/min and renal concerns arise, study drug should be discontinued.

For any other safety concerns that may arise during treatment, the Medical Monitor or 24-hour medical contact number (see Section 4) should be contacted to discuss possible discontinuation of study drug.

16.12 Urgent Safety Measures

The Sponsor and investigator may take appropriate urgent safety measures in order to protect the subjects of the clinical study against any immediate hazard to their health or safety.

The Sponsor and the Medical Monitor or designated deputy will be notified of any urgent safety measures taken by the investigator or qualified designee and advised of their responsibility to notify the licensing authority. The investigator or qualified designee will notify the Independent Ethics Committee/Institutional Review Board (IEC/IRB).

If these measures are taken, the Sponsor or investigator shall immediately give written notice to the pertinent regulatory authorities consistent with the regional/national requirements, IEC/IRB, and DSMB of the measures taken and the circumstances giving rise to those measures. In any event, the written notice shall be no later than 7 days from the date the measures are taken.

17 STATISTICAL ANALYSIS

A SAP will be finalized and signed off prior to database lock and unblinding. The SAP will provide details about the planned analysis. A brief overview of the plans for the primary analysis and selected secondary and exploratory analyses is given in the following sections. Specific ROI and SPM techniques and comparisons to previous Phase 3 data or external databases as well as Population PK analyses will be described in the SAP or a corresponding additional SAP where necessary; the SAP will provide details.

Changes to the planned analysis will be documented in the SAP prior to database lock and unblinding. Deviations from the SAP (if any) will be noted in the clinical study report.

The primary target of estimation is the difference in ADAS-cog$_{11}$ and ADCS-ADL$_{23}$ over 52 weeks in subjects receiving the LMTM dose of 16 mg/day as compared to placebo.

The primary efficacy analyses for FDA will be performed on the Efficacy Modified Intent-to-Treat (E-MITT) which will include all randomized subjects who took at least one dose of study drug and have a baseline and a valid post-baseline efficacy assessment, and for EMA on the Intent-to-Treat (ITT) population with conservative assumptions about disease progression made for subjects who have missing post-baseline assessments. Subjects will be analyzed in the treatment group and according to the stratification variables to which they were randomized.

Changes from baseline to Week 52 on the co-primary efficacy endpoints and other modalities with repeated measures such as MRI will be analyzed using a linear mixed model for repeated measures with unstructured covariance matrix (this assumption about the covariance matrix might be relaxed if the model does not converge; the SAP will provide details). The linear mixed model will contain fixed effects for time, treatment, and a treatment-by-time interaction; additionally, the baseline value of the corresponding endpoint will be included as a covariate and the randomization stratification variables of prior usage of AChEIs and/or

memantine, MMSE split (16-19, 20-25 or 26-27) as determined at Screening, and region will be included as fixed effects in the model. ^{18}F-FDG-PET and other modalities with only one planned post-baseline assessment will be analyzed using an ANCOVA; the covariates for this model will be adjusted accordingly by removing terms with time.

Several of the analyses described below will be performed separately for the comparisons of LMTM 16 mg/day with placebo and 8 mg/day with placebo. As only the LMTM 16 mg/day and placebo comparison is a primary endpoint, the models will use two treatment levels dependent on the comparison of interest. Several analyses provided for the comparisons of LMTM 16 mg/day and placebo will be repeated for the LMTM 8 mg/day *versus* placebo group as secondary or exploratory/sensitivity endpoints. The SAP will detail which analyses are performed for which treatment groups, and in which cases the LMTM 8 mg/day with placebo comparisons will be provided for which sensitivity, subgroup, and exploratory analyses.

Further analysis on selected primary and secondary endpoints will be provided for pooled LMTM 16 mg/day and 8 mg/day arms compared to placebo. The analysis models will be identical to the 16 mg/day *versus* placebo with the exception of pooled treatment arms. The SAP will detail which analyses are repeated for the pooled 16 mg/day and 8 mg/day arms.

More details about the exact contrast statements used for these tests as well as further sensitivity analyses, additional analyses of the primary variables (such as responder analyses and subgroup analyses), and the analyses of the secondary and exploratory endpoints are described in the subsections below as well as in the SAP.

"Late" starters, *i.e.*, subjects originally randomized to placebo, will be compared to "early" starters, *i.e.*, those originally randomized to LMTM 16 mg/day or 8 mg/day (pooled, and individually for both groups as a sensitivity analysis). The last assessment prior to start of open-label treatment (pre-dose at Visit 7) will serve as the baseline assessment for these comparisons. The analyses will be based on a similar linear mixed model for repeated measures with the aim to investigate whether there is a difference in change in primary and MRI imaging endpoints dependent on LMTM treatment history. The comparison will be implemented through a non-inferiority test.

The non-inferiority margin for the primary endpoint of ADAS-cog$_{11}$ will be 2 units, motivated by the fact that these 2 units are small compared to the estimated treatment effect of > 5 units (~40% of this effect size). Margins for other endpoints which are exploratory to support a disease modifying argument will be defined in the SAP. The results will also be presented with the Visit 2 Baseline as baseline and treatment visits Week 26 and Week 52 in the open-label, delayed-start phase as Week 78 and Week 104.

17.1 Efficacy Endpoints

17.1.1 Primary Efficacy Endpoints for Double-Blind Treatment Period

- ADAS-cog$_{11}$ (LMTM 16 mg/day *versus* placebo)
- ADCS-ADL$_{23}$ (LMTM 16 mg/day *versus* placebo)

17.1.2 *Secondary Efficacy Endpoints for Double-Blind Treatment Period*

- Annualized rate of whole brain atrophy on brain MRI using BSI (LMTM 16 mg/day *versus* placebo)
- Difference in temporal lobe ^{18}F-FDG-PET change in SUVR normalized to pons in subjects with CDR 0.5 at Screening (LMTM 16 mg/day *versus* placebo, and LMTM 8 mg/day *versus* placebo), if a sufficient number of subjects have provided data as specified in the SAP
- ADAS-cog_{11} and ADCS-ADL_{23} (LMTM 8 mg/day *versus* placebo)
- Annualized rate of temporal and parietal lobe atrophy on brain MRI using BSI (LMTM 16 mg/day *versus* placebo, and LMTM 8 mg/day *versus* placebo)

17.1.3 *Secondary Endpoint for Open-Label, Delayed-Start Phase*

- Difference in disease progression on the co-primary clinical endpoints and the MRI imaging endpoint for subjects who started treatment in the double-blind treatment phase and those who started treatment in the open-label, delayed-start phase (referred to as "early" and "late" LMTM starters, respectively)
  - Only ADAS-cog_{11} will serve as a secondary endpoint; ADCS-ADL_{23} and other imaging endpoints are exploratory with the aim to be directionally supportive

17.2 Number of Subjects and Sample Size Calculation

The target number of subjects is approximately 450 subjects enrolled in Protocol Version 5.0 and above, randomized in a ratio of 4:1:4 (at the study level) to the LMTM 16 mg/day (200 subjects), LMTM 8 mg/day (50 subjects) and placebo (200 subjects) groups; total enrollment is approximately 500 subjects. Subjects will be assigned to the MMSE severity groups based on screening MMSE score with a target of approximately 2:3:1 (MMSE 16-19, MMSE 20-25, MMSE 26-27 respectively) for those randomized to Protocol Version 5.0 and above. Subjects who drop out after randomization will not be replaced.

Sample size estimations to achieve 90% power (two-sided alpha = 0.05) to detect a difference between LMTM 16 mg/day and placebo (containing intermittent MTC as a urinary discolorant), the primary treatment group comparison in the double-blind treatment period, have been performed for the two co-primary clinical endpoints. These assume a withdrawal rate of 20% to 25% over 52 weeks. The study sample size of approximately 450 subjects (approximately 200 subjects in each treatment group, with a further 50 subjects for secondary analyses of an LMTM 8-mg/day group) is based on the ADCS-ADL_{23} as a larger sample size is required to achieve the target power.

Based on an estimated decline in ADCS-ADL_{23} over 52 weeks in the control arm of 7.7 units with an estimated SD of 8.5 units, the study will have >90% power (two-sided alpha=0.05) to detect a reduction in decline of 3.4 units or more. The 3.4 units are motivated by an estimated treatment effect of 5.0 ± 1.6 (mean ± standard error) units in the pooled studies TRx-237-005/ TRx-237-015.

Based on an estimated decline in ADAS-cog_{11} over 52 weeks based on pooled information from Studies TRx-237-005 / TRx-237-015 in the control arm of 6.5 units with an estimated SD of 5.9 units, 200 subjects per treatment arm provide >90% power (two-sided alpha=0.05) to detect a reduction in decline of 2.6 units or more. The 2.6 units represent a conservative value as the estimated treatment effect based on pooled Studies TRx-237-005 / TRx-237-015 is 5.2 ± 1.3 (mean ± standard error) units.

With 200 subjects randomized to the primary comparison in the double-blind treatment period under Protocol Version 5.0 and above, 160 to 170 subjects per arm will enter the open-label, delayed-start phase assuming the drop-out rates mentioned above. Assuming a further 10% drop out in the delayed-start phase, the key secondary analysis to demonstrate disease modification by comparing early to late starters with a noninferiority margin of -2 ADAS-cog_{11} units has approximately 80% power.

Subjects who withdraw from treatment will be encouraged to stay in the study and continue their assessments off-treatment. Subjects' withdrawal rates will be closely monitored in a blinded fashion, to monitor if sample size calculations remain correct.

17.3 Analysis Populations

The following subject populations will be used for the statistical analyses; all definitions reply on the treatment period in the double-blind phase excluding open-label assessments (population definitions will be used for the delayed-start analyses without any modifications):

- Intent-to-Treat (ITT) population will include all randomized subjects. Three subsets are defined as follows:
  - The Efficacy Modified Intent-to-Treat (E-MITT) population will include all randomized subjects who take at least one dose of the study drug and have a baseline and at least one valid post-baseline efficacy assessment in the treatment period (prior to any potential follow-up assessment).
  - An MRI Imaging Modified Intent-to-Treat (MI-MITT) population will include all randomized subjects who took at least one dose of study drug and have at least one valid baseline and at least one valid post-baseline volumetric MRI.
  - The Imaging Modified Intent-to-Treat population (PI-MITT) for ^{18}F-FDG-PET will include all randomized subjects with screening CDR 0.5 who took at least one dose of study drug and have a baseline and a valid post-baseline SUVR assessment.
- The Per Protocol (PP) population will include all subjects who are in the E-MITT and MI-MITT populations and who do not have any PP exclusionary protocol deviations or intercurrent medical events that could confound the interpretations. PP exclusionary deviations, irrespective of their classification as minor/major, and intercurrent medical events will be determined prior to treatment unblinding.
- The Safety population will include all randomized subjects who take at least one dose of study drug.

Due to the introduction of MTC spiking in the placebo arm with Protocol Version 5.0, the respective MITT populations will be restricted to subjects randomized to Protocol Versions 5.0 and above for the primary and key secondary analyses as per the SAP. The full MITT populations will be analyzed for these as a sensitivity analysis. Details will be provided in the SAP. For the ITT, respective restrictions will be applied as part of the first step in the EMA analysis (see Section 17.4.3).

17.4 Clinical Efficacy and Imaging Analysis

The various analyses planned in the study are outlined below. The SAP will be more specific and clearly state which analysis and which model will be applied to what type of data.

Subjects will be analyzed in the treatment group and stratification to which they were randomized unless stated otherwise in potential sensitivity analyses. For safety analyses, treatment assignment will be based on the treatment actually received. If a subject receives an incorrect treatment transiently, he/she will be assigned to the predominant treatment group (*i.e.,* the treatment group for which he/she received the greatest number of doses).

For E-MITT, MI-MITT, and PI-MITT analyses, only valid data will be included in the primary and secondary analyses. In subjects who have initiated treatment with an AChEI and/or memantine, assessments made after initiation of such treatment are not considered valid as they could confound the interpretation of the results and will be excluded. Such subjects and affected assessments will be identified prior to unblinding. Sensitivity analyses will be provided for the primary and selected secondary analyses including all data.

The primary and key secondary analyses will be further restricted by excluding subjects who were randomized under protocols prior to Protocol Version 5.0; this is due to the spiking with MTC in the placebo arm. Sensitivity analyses not applying this restriction will be provided.

Various imaging and clinical efficacy analyses will be handled differently for the FDA and European Medicines Agency (EMA). Summary statistics will be tabulated by visit and treatment group using observed data. ITT, E-MITT, MI-MITT, and PI-MITT population-weighted least squares means (LSM), unless otherwise stated, treatment differences, and 95% confidence intervals will also be included; the population will be determined by the corresponding model, dependent on whether the endpoint is an imaging endpoint or an efficacy endpoint. Where requested, annualized rates will be estimated based on the population-weighted LSM.

All models will include the randomization stratification variables as covariates unless otherwise stated. If the models do not converge because of too few observations for a given randomization stratification variable, the corresponding variable/variables will be removed from the model.

Various 8 mg/day analyses might be omitted if not promising based on the secondary or other analyses. These and corresponding thresholds will be defined in the SAP. In the specification of various analyses below, they are included for sake of completeness and as they might be performed.

Data listings will include all observed data for the primary and secondary endpoints.

17.4.1 Hypothesis

There are two co-primary efficacy endpoints for this trial: Baseline adjusted decline in ADAS-cog_{11} and Baseline adjusted decline in ADCS-ADL_{23} from Baseline at Week 52. Both

co-primary endpoints must reach significance based on the use of a two-sided test at the alpha=0.05 level of significance for LMTM to be designated as superior to placebo.

The primary analysis will be performed using the E-MITT/ITT populations as defined in Section 17.3, with the additional exclusions mentioned in Sections 17.3 and 17.4.

The global null hypotheses are as follows:

H_{01}: There is no difference between the LMTM 16 mg/day and placebo groups change in ADAS-cog_{11} from baseline to Week 52

and

H_{02}: There is no difference between the LMTM 16 mg/day and placebo groups change in ADCS-ADL_{23} from baseline to Week 52

The global null versus alternative primary efficacy hypotheses is a Union-Intersection Test which requires both co-primary endpoints to meet statistical significance at the 5% two-sided level of significance for the global null hypothesis to be rejected. Additional details will be provided in the SAP.

All secondary analyses have the same underlying null hypothesis that there is no difference in change from baseline in the quantity of interest between the LMTM 16-mg/day group, or the LMTM 8-mg/day group, and the placebo group. For association analyses, the null hypothesis is that there is no association between the variables of interest.

17.4.2 MITT (FDA) Analysis

The FDA analyses will be based on the E-MITT, MI-MITT, or PI-MITT population, with the additional restriction as per Sections 17.3 and 17.4, depending on endpoint, and will include all available valid data for each subject. No data will be imputed (unless specified in selected sensitivity/exploratory analyses). Sensitivity analyses for selected endpoints will be provided where all assessments are included.

Change from baseline in the respective efficacy/imaging parameter will be analyzed using a restricted maximum likelihood-based repeated measures linear mixed model. The model will include fixed effects for treatment group (two levels dependent on the comparison, three levels for sensitivity); nominal time; the treatment group-by-time interaction; and covariates for categorical severity, region, and prior use of AChEIs and/or memantine. The corresponding baseline parameter will be included as a covariate (continuous) as well (certain covariates in the model might be dropped if the model does not converge; see SAP for details). An unstructured covariance model will be used. The Kenward and Roger method of calculating the denominator degrees of freedom will be used for the tests of fixed effects. Treatment comparisons will be based on the modeled change from baseline at Week 52.

If there is only one scheduled post-baseline imaging or efficacy assessment, the model will be replaced by a simple linear model with fixed effects for treatment group and the randomization stratification variables and with the baseline value of the corresponding endpoint as a covariate.

For annualized rate of atrophy or decline, time will be used as a continuous variable supported by corresponding contrast analyses of time as a nominal variable.

17.4.3 ITT (EMA) Analysis

The EMA analyses will be based on the ITT population, regardless of endpoint.

Change from baseline in the respective efficacy/imaging parameter will be analyzed using a restricted maximum likelihood-based repeated measures linear mixed model or, in the case of only one scheduled post-baseline imaging or efficacy assessment, using a simple linear model (the models used are described above in Section 17.4.2). This analysis will be restricted to scheduled, on-treatment visits for subjects randomized under Protocol Version 5.0 and above (a sensitivity will be provided including data from subjects of all protocol versions). An on-treatment visit is a visit where a subject was on investigational product and not on any AChEI and/or memantine.

This provides the on-treatment effect, E, at the final time point. For each subject who withdrew from the study, the decision will be made as part of the blinded data review meeting as to whether the withdrawal was potentially treatment-related. For potentially treatment-related drop-outs, the fraction of subjects randomized to active treatment who withdrew at a given time point will be calculated. If a subject is determined to have withdrawn for non-treatment-related reasons, no further adjustment will be made as the mixed effects model correctly accounts for subjects who are missing at random.

In this intervention effect analysis, subjects who withdraw for potential treatment-related reasons are assumed to retain 100% of the treatment effect they had attained up to the point of withdrawal but do not continue to benefit from treatment afterwards. This corresponds to the estimate I = (E0*w0 + E13*w13 + E26*w26 + E39*w39) + E52*(1-w0-w13-w26-w39), where E13 is the treatment effect at week 13. W13 is defined as the fraction of subjects who withdrew for potentially treatment-related reasons, have a non-missing imaging/efficacy parameter at Week 13, and do not have any on-treatment assessments at any scheduled visit subsequent to Week 13. The other wxx are defined likewise. Wxx is calculated for the active treatment group only. W0 refers to subjects who had no measurement taken after the baseline measurement and thus E0 is zero. This analysis will be implemented as a contrast statement and the estimate I will be reported as the intervention effect.

Annualized rate of atrophy or decline will also be analyzed in a similar fashion.

17.4.4 Estimands

In general, definition of estimands in the analysis of clinical trials become increasingly important. The SAP will specify estimands to provide a more comprehensive assessment of the performance and efficacy of the drug accounting for various factors (importantly, drop-outs).

In line with the ITT analysis of Section 17.4.3, the primary estimand therefore is designed to answer the question on the treatment effect of the LMTM dose of 16 mg/day as monotherapy *versus* placebo (occasional 4 mg/day MTC) in the targeted population of subjects with probable AD and MCI-AD at Week 52, regardless of study drug discontinuation, based on the two co-primary endpoints.

This estimand is constructed in line with ICH E9 (R1) addendum. The five components defining the estimand of interest are listed below:

**A. Treatment:**	52 weeks of LMTM 16 mg/day or matching placebo (4 mg/day MTC) as monotherapy, regardless of adherence.
**B. Population:**	Subjects with probable AD or MCI-AD as described by the inclusion and exclusion criteria, randomized under Protocol Version 5.0 or higher.
**C. Patient-level outcomes / variables:**	Change from Baseline of the ADAS-cog_{11} and ADCS-ADL_{23} at Week 52 as co-primary outcomes.
**D. Population-level summary:**	Population-weighted least squares mean difference.
**E. Intercurrent Events (ICE):**	The following ICE have been identified which could prevent measurement of the primary outcome or change the interpretation of the measured primary outcome:<br>1. Withdrawal from study before completion of 52-week treatment period for non-treatment related reason<br>2. Withdrawal from study before completion of 52-week treatment period for treatment-related reason<br>3. Initiation of AChEI and/or memantine<br>4. Study treatment discontinuation for any reason other than initiation of AChEI and/or memantine (including discontinuation by the investigator if he/she judges that treatment is no longer appropriate, if the subject's clinical condition is worsening or for an AE, or due to study drug dose interruption that is longer / more frequent than specified in ICE #5 below) (see Section 17.4.11.4 regarding analysis of the TOTOS group)<br>5. A dose interruption for more than 14 consecutive days or more than two occasions of dose interruptions up to a maximum of 14 consecutive days<br>6. Intercurrent illnesses, or initiation of medical food or medications not allowed by protocol (which will be identified during the blinded data review meeting)<br>7. Study treatment non-compliance, defined as <80% or >120% taking into consideration any dose interruptions (if this cannot be estimated, it does not result automatically in a non-compliance)<br>8. Death before completion of 52-week treatment period<br>9. COVID-19 infection<br>10. Death due to COVID-19

The handling of ICEs is described as follows:

- **Events 1** will be handled according to a hypothetical strategy as if subjects have stayed on treatment; in a sensitivity analysis, all these subject withdrawals are assumed to be potentially treatment-related, which are then handled using the treatment policy approach from ICE #2.
- **Events 2** will be handled using a treatment policy approach reflecting Copy Incremental from Reference strategy (using data after occurrence of the ICE). Subjects who withdraw for treatment-related reasons are assumed to retain 100% of the treatment effect they had attained up to the point of withdrawal but do not continue to benefit from treatment afterwards, *i.e.*, assuming that the clinical course post ICE for either treatment group follows the placebo group. This corresponds to the estimate I = (E0*w0 + E13*w13 + E26*w26 + E39*w39) + E52*(1-w0-w13-w26-w39), where, for instance, *E13* is the treatment effect at Week 13 and *W13* is defined as the fraction of subjects who withdrew for a potential treatment-related reason, have a non-missing efficacy parameter at Week 13, and do not

have any on-treatment assessments at any scheduled visit subsequent to Week 13. The fractions are calculated within the active treatment group. *W0* refers to subjects who had no measurement taken after the baseline measurement and thus *E0* is zero. The estimate *I* will be reported as the intervention effect and will be calculated using a contrast on the LSM (see Section 17.4.3).

   - Two sensitivity analyses will be run using hypothetical strategies (not using data after occurrence of the ICE), one by imputing missing data for both treatment groups based on data seen in the placebo group, and one by imputing missing data for each treatment group based on data seen in their own group.
   - In another sensitivity analysis, the alternative assumption will be investigated, that the subjects withdrawing from treatment for treatment-related reasons do not retain any treatment effect after Baseline; the intervention effect *I* in this case is I=E*(1-w), where *E* is the treatment effect at Week 52 and *w* is the fraction of subjects within the active treatment group who withdraw for a potential treatment-related reason up to Week 52.

- **Events 3** will be handled according to the original randomized treatment group assuming they stopped treatment and did not start AChEI and/or memantine (analyzed same as ICE #2), not using data after occurrence of the ICE.
- **Events 4** will be handled according to a treatment policy strategy, using data after occurrence of the ICE. In addition, for EMA, the analysis will be carried out using the strategy as described for ICE #2 (not using data after occurrence of the ICE).
- **Events 5, 6, and 7** will be handled according to a treatment policy approach, using all data (also after occurrence of respective ICE).
- **Events 8** will be handled as treatment failure. If the subject dies prior to Week 52, the population average in decline within the placebo arm will be imputed.
- **Events 9 and 10** will be handled with a hypothetical strategy assuming COVID-19 disease would not have happened, not using data after occurrence of the ICE.

17.4.5 Handling of Missing and Incomplete Data

Missing imaging data will not be imputed; it will be assumed that the data are close to missing at random after accounting for the terms in the model for all analyses other than those described for the EMA ITT-based analyses. The EMA ITT-based analyses treat subjects for whom assessments are missing from a certain point onwards as having no treatment benefit thereafter; while the primary analysis assumes that they retained the treatment effect that they had at the point of drop-out, a sensitivity analysis treats them as having lost all treatment effect. While the primary analysis assumes that some of subjects' data might be missing at random for drop-outs, a sensitivity analysis assumes all withdrawals were treatment related. Various other sensitivity analyses, including multiple imputation, time to event, and responder analyses, will be provided to investigate the robustness of the primary and secondary analyses (see respective sections below for more detail; drop-outs are treated as non-responders or having an event of decline in these models).

Efficacy data missing for an entire outcome scale or for the majority of the scale will not be imputed unless stated otherwise. Instead, it will be assumed that the data are close to missing at random after accounting for the terms in the model. However, missing items within a subdomain or scale may be upscaled if some items of the subdomain or scale are present; the SAP will provide details of the handling of partial data for the efficacy endpoints for each scale. As automated data capturing devices are used for all efficacy assessments, the risk of missing data of this type is low. For imaging data, this type of missing data is impossible and consequently they are not subject to upscaling either.

17.4.6 Open-Label, Delayed-Start Analysis

"Late" starters, *i.e.*, subjects originally randomized to placebo, will be compared to "early" starters, *i.e.*, those originally randomized to LMTM 16 mg/day or 8 mg/day (pooled, and individually for both groups as a sensitivity analysis). The last assessment prior to start of open-label treatment (pre-dose at Visit 7) will serve as the baseline assessment for these comparisons. The analyses will be based on a similar linear mixed model for repeated measures as outlined for the FDA/EMA primary analysis (see model specification in Sections 17.4.2 and 17.4.3) with the aim to investigate whether there is a difference in change in primary and MRI imaging endpoints dependent on LMTM treatment history (certain covariates in the model might be dropped if the model does not converge; see SAP for details).

The comparison will be implemented through a non-inferiority test. The non-inferiority margin for the primary endpoint of ADAS-cog_{11} will be 2 units, motivated by the fact that these 2 units are small compared to the estimated treatment effect of > 5 units (~40% of this effect size). Margins for other endpoints which are exploratory to support a disease modifying argument will be defined in the SAP.

The results will also be presented with the Visit 2 Baseline as baseline, and treatment visits Week 26 and Week 52 in the open-label, delayed-start phase as Week 78 and Week 104.

17.4.7 Dose Response Analyses

Various dose response analyses will be performed. The primary endpoints and key secondary endpoints will be analyzed using FDA/EMA primary analyses (see model specification in Sections 17.4.2 and 17.4.3) to show the functional relationship of dose and response on the various endpoints as change from baseline to Week 52. This analysis will also be provided split by MMSE groups as randomized. For CDR 0.5 subjects, the listed secondary analysis of ^{18}F-FDG-PET will be translated into a dose response curve as well.

Several of these dose response analyses are contingent on primary analyses and key secondary analyses showing a significant effect as outlined in the SAP.

17.4.8 Responder Analyses

Responder analyses will be conducted for the primary and selected secondary endpoints by dichotomizing each endpoint.

For the corresponding imaging/efficacy parameter, a responder will be defined as a subject whose change from baseline is less than or equal to a threshold "T" defined as follows (these analyses will be done separately for the LMTM 16-mg/day *versus* placebo and LMTM 8-mg/day *versus* placebo groups):

- Let LSM1 be the LSM of change from baseline for the placebo group from the respective endpoint analysis
- Let LSM2 be the LSM of change from baseline for the LMTM group from the respective endpoint analysis
- Then T = (LSM1 + LSM2)/2

Thresholds will be chosen based on the E-MITT, MI-MITT, or PI-MITT population-weighted LSMs from the respective model. Subjects who do not have a final assessment will be classified as non-responders.

For each separate LMTM group comparison *versus* placebo, the proportions of responders will be compared using the Cochran-Mantel-Haenszel test, adjusting for the randomization strata. Odds ratios and 95% confidence intervals will be presented.

For each of these analyses, if there is a significant effect of treatment on an imaging as well as another imaging or clinical efficacy parameter, the association between responders across these parameters at Week 52 will be assessed by using Pearson's chi-square test to analyze the resulting 2 × 2 table. The number and percent of subjects in each cell of the 2 × 2 table will be tabulated along with the p-value from the Pearson's chi-square test.

Responder analyses will be used to investigate associations between various endpoints. Correlation analyses will complement or replace responder-based analyses of associations in some cases.

17.4.9 Time-to-Event Analyses

Time-to-Event/Time-to-Decline analyses will be performed for a selected subset of endpoints as repeated measurements over time are needed. The distributions of time-to-decline in the key parameters will be summarized using the Kaplan-Meier method, with onset of decline defined as the first of two consecutive measurements that are worse than the baseline score, allowing for a certain margin which will be predefined in the SAP prior to database lock and unblinding; typical margins for the two primary endpoints are 1 unit. If there is no onset of decline prior to Week 52 and if Week 52 has a marked worsening as specified in the SAP for a given subject, an onset of decline will be triggered at Week 52 for that subject. Time-to-decline will be calculated as date of onset of decline – date of randomization + 1.

The time-to-decline analyses are based on visit window. One measurement per visit window is selected, including off-treatment measurements. Subjects without any decline will be censored at the date of last assessment, but if a subject missed the scheduled visit, the subject is censored at the target day of the scheduled visit. The analysis will be run for the ITT population.

Any missing efficacy/imaging value for a scheduled visit will be treated as a worsening from baseline, for that visit. Because of this convention for missing values, it is possible that the first of two consecutive measurements that are worse than the baseline score by the defined amount occurs at a missed visit. In that case, since there is no date associated with the missed visit, the time-to-decline will be calculated based on the scheduled date of the missing visit.

The Cox proportional hazards regression model with effects for treatment group (two or three levels depending on whether primary or sensitivity comparison) and the randomization stratification variables will be used to compare LMTM 8 mg/day *versus* placebo (and LMTM 16 mg/day *versus* placebo, respectively) by way of Hazard Ratio, 95% confidence interval, and p-value (for alpha 0.05). A graph of the Kaplan-Meier estimates will be provided.

There will also be versions of this analysis using actual dates.

17.4.10 Subgroup Analyses

For selected endpoints and treatment groups, subgroup analyses will be provided. These are implemented by restricting a given analysis to the corresponding subgroup or by adding interaction terms in the model with appropriate contrast statements as described in the SAP.

Subgroup analyses will be performed for the primary endpoints and selected secondary endpoints based on the E-MITT/MI-MITT/PI-MITT populations (FDA analysis) by repeating the analysis as described for the primary and secondary endpoints restricted to the subgroup. If the subgroup consists of less than 10% of subjects in observed cases, only summary statistics will be presented. The subject characteristics that will be included in this type of analysis are the following:

- Baseline diagnosis (probable AD and MCI-AD)
- MMSE (16-19, 20-25, and 26-27)
- CDR (0.5 and CDR 1 or 2 pooled)
- AChEI/memantine use (prior use and never used)
- Age group (<75 years and ≥75 years)
- Gender (male and female)
- Race (white and non-white)
- Geographic region (North America and Europe)

17.4.11 Sensitivity Analyses

Sensitivity analyses for key primary as well as secondary parameters will be conducted to assess the impact of missing data and to assess alternative models.

See Section 10.2.3 for a summary of the modifications regarding sensitivity analyses to be implemented due to COVID-19. The SAP will be more specific about the analyses conducted, including those related to COVID-19 impact assessment and will follow available regulatory guidelines.

17.4.11.1 Mis-stratification Analysis

If there are more than 5% of subjects who have been mis-stratified, then the primary analyses will be repeated using the actual status at randomization.

17.4.11.2 All Protocol Version Analysis

As outlined in Sections 17.3 and 17.4, the MITT and on-treatment populations will be restricted further in the primary analysis. A sensitivity will be provided for the respective analysis with all data from subjects or all protocol versions included (unrestricted MITT/on-treatment definitions).

17.4.11.3 Analysis of Subjects Who Started AChEI and/or Memantine

For the FDA analyses, in subjects who initiated treatment with AChEI and/or memantine at any time during the study, data after initiation of such treatment are excluded from the primary analyses as they could confound the results. Such subjects will be identified prior to

unblinding. For EMA analyses, such subjects are treated as subjects who terminated the study at the time of starting AChEI and/or memantine; a conservative post-withdrawal course is assumed for these subjects.

Data for these excluded subjects will be tabulated separately with their observed means, standard deviations, standard errors, and minimum and maximum values of selected changes in imaging and efficacy assessments. Primary reasons for initiating antidementia therapy will be provided. Furthermore, tables will be provided quantifying the number of assessments as well as time points that are impacted by these exclusions.

Sensitivity analyses will be performed including all data for these subjects. Time to event (*i.e.*, time to withdrawal in order to start AChEI and/or memantine) may also be performed if more than 10% meet this criterion; if not, the information will be tabulated.

17.4.11.4 Analysis of the Off-Treatment-On-Study (TOTOS) Subjects

Data for the TOTOS subjects will be handled in a similar fashion as described in Section 17.4.11.3. For the FDA analyses, the TOTOS subjects are included in the analyses (unless they initiated AChEI and/or memantine, in which case data after the initiation of such therapy will be excluded). For the EMA analysis, all TOTOS subjects are treated as subjects who terminated the study; a conservative post-withdrawal course is assumed for these subjects.

Data for these subjects will be tabulated separately with their observed means, standard deviations, standard errors, and minimum and maximum values of selected changes in imaging and efficacy assessments; primary reasons for withdrawal of study drug will be provided. Furthermore, tables will be provided quantifying the number of assessments as well as time points that are impacted by these exclusions.

Sensitivity analyses will be performed including all data for these subjects.

17.4.11.5 Imputation Methods

No data will be imputed in the primary and secondary analysis models unless stated otherwise. As sensitivity analyses, two methods for imputation will be explored, as described below.

17.4.11.5.1 Multiple Imputation

A multiple imputation analysis will be carried out using SAP-specified endpoints and analysis models, but will include the primary and selected secondary endpoints. The missing values will be imputed with multiple imputation methodology using PROC MI in SAS. A multivariate normal imputation model will be used by treatment group with a seed of 237039. The imputation model will have exactly the same covariates as the respective models. A total of 50 imputed datasets will be generated for this analysis. Each of the imputed datasets will be analyzed using the respective model with the same covariates. The MIANALYZE procedure in SAS will be used to combine results.

For endpoints that do not have planned multiple post-baseline assessments, additional variables such as sex, age, and other baseline values will be employed to allow appropriate multiple imputation.

17.4.11.6 Baseline Severity Rate-correction Analysis

The FDA model will include baseline value as an additive covariate. In order to determine whether baseline value influences future assessments, *i.e.*, the rate of progression, a further sensitivity analysis which includes the additional term baseline*visit instead of just baseline will be conducted.

17.4.11.7 Sensitivities for Intervention Effect Analyses

Further sensitivity analyses are planned to determine the effect of withdrawals on estimation of intervention effect (as required by the EMA). These include the alternative assumption that the subjects withdrawing from treatment do not retain any treatment effect; the intervention effect I in this case is I=E*(1-w), where w is the fraction of subjects who withdraw for potential treatment-related reasons. This is implemented as a contrast statement as well. This analysis is only needed if there are more than two scheduled visits with imaging/efficacy assessments, as otherwise this analysis is identical to the EMA analysis model specified in Section 17.4.3.

An additional sensitivity analysis will assume that all subjects withdrew due to a potential treatment-related reason.

17.4.11.8 Polynomial Models

For the key efficacy/imaging assessments with more than one scheduled post-Baseline visit, maximum likelihood (rather than restricted maximum likelihood) based repeated measures models with polynomial time effects with time treated as a continuous variable (number of nominal weeks as well as actual study week defined by study day divided by 7) will be assessed. Tests of the significance of the linear, quadratic, and cubic time effects will be carried out in each treatment group. The estimated annualized change in mean values and standard errors from baseline to Week 52 will be presented.

17.4.11.9 Other Population Analyses

As a sensitivity analysis, the FDA primary analyses and potentially other pre-specified endpoints will be repeated for:

- The PP population
- The set of completers (*i.e.*, all subjects who were on-treatment at Week 52)
- All visits that are not potentially confounded by intercurrent illnesses, COVID-19, and concomitant medications as identified during the blinded data review meeting

17.5 Exploratory Analyses

A number of exploratory analyses will be undertaken, to be specified in the SAP. These could include the following (details will be provided in the SAP):

- To compare the treatment groups on the ADAS-cog_{13}
- To compare the treatment groups on a new composite endpoint/composite endpoints based on selected item of the Alzheimer’s Disease Assessment Scale, 13-item version

(ADAS-cog$_{13}$) and the ADCS-ADL$_{23}$, analyzed at 9 months and 12 months to evaluate its usefulness for future studies (for all subjects and also separately for subjects with CDR 0.5 and CDR 1-2 at screening)
- To compare the treatment groups on the MMSE
- To compare the treatment groups on the CDR sum of boxes
- Comparison of atrophy in MRI parameters (including putamen, nucleus accumbens, and nucleus basalis) according to treatment assignment, as well as the combination of treatment assignment with previous treatment status of AChEIs and/or memantine (prior use or never used) in a subgroup analysis; the influence of the baseline volumes of putamen, nucleus accumbens, and nucleus basalis will also be investigated for selected MRI parameters. Further SPM analyses may be conducted using all available MRI volumetric data to determine regions of significant difference associated with treatment
- Comparison of decline in ^{18}F-FDG-PET SUVR normalized with respect to pons and cerebellum in temporal, parietal, and frontal lobes according to treatment assignment, as well as the combination of treatment assignment with previous treatment status of AChEIs and/or memantine (prior use or never used) in subgroup analyses
- Determination of decline in SUVR in cerebellum normalized with respect to pons according to treatment assignment, as well as the combination of treatment assignment with previous treatment status of AChEIs and/or memantine (prior use or never used) in a subgroup analysis
- Other SUVR regions of interest will also be examined including, but not restricted to, inferior temporal gyrus, angular gyrus, anterior and posterior cingulate gyrus, and cerebellum
- SPM analyses (Friston *et al*., 2007) may be conducted using all available normalized cortical ^{18}F-FDG-PET SUVR data and MRI data to determine regions of significant difference associated with treatment without assuming the location and extent of any differences
- To examine the associations of SPM approaches using all available normalized ^{18}F-FDG-PET SUVR data and volumetric MRI data with the following clinical measures:
    - ADAS-cog$_{13}$
    - ADCS-ADL$_{23}$
    - Composite Scales (selected)
- Comparisons of measures of brain MRI hyperintensities by treatment group; subgroup analyses by previous treatment status of AChEIs and/memantine (prior use or never used) will also be performed, including for the combined LMTM 8-mg/day and placebo groups
- Population pharmacokinetics (to be subject to a section in the SAP or a separate SAP and report)
- To evaluate the influence of *ApoE* genotype (in subjects who provide legally acceptable consent) on the primary and selected secondary endpoints

A number of exploratory comparisons with external data may also be performed, to be specified in the SAP. These include comparisons of LMTM 16 mg/day or LMTM 8 mg/day on the following:
- Comparison of annualized rates of atrophy in a range of brain regions (including temporal lobe and whole brain) with the estimated decline for subjects with MCI-AD and mild AD with MMSE in the range of 20-27 not receiving either of the standard

treatments for AD (AChEIs and/or memantine) from the ADNI; this will be repeated for the entire MMSE range used in this study

- To compare the primary and selected secondary imaging and efficacy endpoints at 52 weeks with pooled mild AD subjects in control groups (subjects receiving LMTM at any dose in combination with an AChEI and/or memantine) from the LMTM Phase 3 trials (Studies TRx-237-005 and TRx-237-015) with baseline MMSE in the range of 20-26; this will be repeated for the entire MMSE range used in this study
- Comparison of decline in temporal lobe ^{18}F-FDG-PET SUVR with the estimated decline for subjects with mild AD (CDR 0.5 at Screening) not receiving either of the standard treatments for AD (AChEIs and/or memantine) from the ADNI
- Accounting for and using historic data more generally such as placebo decline or treatment effects as priors to inform the analyses of this study.

17.6 Demographic and Baseline Characteristics

Demographic variables, baseline characteristics and medical history will be summarized in tables. Medical history will be coded using the Medical Dictionary for Regulatory Activities (MedDRA) Version 20.1. The tabular summaries will present the numbers and percentages of subjects with abnormalities in a given MedDRA System Organ Classification (SOC).

Recently used medications and concomitant medications will be coded using the 01March 2017 version of the WHO drug dictionary. Tabulations will be prepared of all drugs used concomitantly (relative to the first dose of study drug) based on WHO Anatomical Therapeutic Classification (ATC) level 1 term, ATC level 3 term, and Preferred Term (generic name) with frequency and percentage of subjects in each treatment group using each concomitant medication. Separate tabulation will be made of on-treatment changes in antipsychotic medications (to be identified by blinded review), together with the reason(s) for such change.

Further information indicating years spent in full-time education and its level before the age of 26 years is to be collected. In addition, main occupation during working life is to be provided (see Section 28.3); main occupation will be coded using the Standard Occupational Classification, 2010, Volume 2 The Coding Index, UK Office for National Statistics. These have been found to be useful proxies for cognitive reserve (Staff *et al.*, 2004). Exploratory analyses will be described in the SAP.

All data, including study eligibility and screening data (including reason for exclusion), will be listed.

Subjects who are mis-stratified at the time of randomization will be flagged in the listing.

17.7 Safety Analysis

Various safety analyses will be conducted for this study. Details will be provided in the SAP. The planned safety analyses, including tabulations by treatment group, include:

- A quantification of the extent of drug exposure
- Various analyses of AEs (using MedDRA Version 20.1)
    - TEAEs, including by relationship and intensity
    - TEAEs that result in interruption or discontinuation of study drug
    - Selected subsets of TEAEs (referred to as TauRx AE Groupings)

    - Subgroup analyses
    - Time-to-event analyses of TEAEs
    - SAEs and serious adverse reactions
    - Malignancies other than non-melanoma skin cancers
- Clinical laboratory evaluations
- Vital sign analysis
- Physical and neurological examinations

All safety data will be listed.

17.8 Other Data

Total duration of exposure and mean and modal daily dose per subject (including "dose equivalent" for subjects randomized to placebo), will be summarized descriptively by treatment group. Mean, modal, and maximum dose will also be summarized over selected exposure intervals. In addition, tabular summaries of the proportions of subjects with dose interruptions will be prepared for each treatment group. Listings will encompass dosing, drug accountability, and compliance (percentage of tablets taken relative to intended number); any doses that are other than that randomized will be flagged.

The plasma MT and whole blood concentrations (parent MT/LMT, *N*-desmethyl MT, and total MT concentrations, as available) will be listed. The results of population PK and exposure-response analyses will be provided in a separate report.

ApoE genotype will be listed for each subject who consents to this determination. Use of these data in exploratory analyses will be described in the SAP.

17.9 Interim Analysis

No interim futility or efficacy analysis is planned prior to the completion of the double-blind treatment period in which treatment groups will be compared.

Recruitment and discontinuations will, however, be continuously monitored in a blinded fashion to ensure that the sample size calculations remain appropriate. The sample sizes may be increased but not decreased; thresholds will be defined in an interim monitoring SAP. Specifically:

- Should the overall dropout for the duration of the study be projected to exceed 25%, then the number of subjects to be enrolled may be increased in order to power an analysis at 52 weeks.
- The assumed SD for the change from baseline to Week 52 in the primary endpoints may be re-estimated at some point during the study's recruitment period and the sample size required to provide 90% power for the primary endpoints will be re-estimated.
- The balance between the three different MMSE stratification groups is determined to be approximately 2:3:1 (MMSE 16-19, 20-25, and 26-27) for those randomized to Protocol Version 5.0 and above. This value will be closely monitored and controlled at study level. Should the balance be deviating from the desired balance, then the number of subjects to be enrolled may be increased in order to rebalance the design enriching the MMSE groups that are underrepresented.

- As indicated in Section 10.2, COVID-19 can have a significant effect on the number of assessments available for analysis. Quality and subitems of assessments can also be impacted, leaving the primary analysis underpowered. The interim monitoring SAP will provide details regarding how this will be assessed prior to any analysis being conducted and will also outline approaches that are to be followed given pre-determined thresholds.

As noted in Section 10.7, an analysis of the primary efficacy endpoints will be undertaken when the last subject completes the final visit in the double-blind treatment period.. If the null hypotheses presented in Section 17.4.1 are rejected, complete safety and efficacy analysis of the data from the double-blind treatment period will be undertaken and an interim study report prepared. The complete Clinical Study Report will be prepared upon completion of the entire study as defined in Section 10.7.

18 REGULATORY AND ETHICS

Investigators and all other parties involved in the conduct of the study are responsible for ensuring that the study is conducted at their sites in accordance with the approved protocol and with the principles contained in the Declaration of Helsinki, the ICH Guidelines for Good Clinical Practice (GCP)[7] and with applicable country and local regulatory requirements and laws. All deviations identified at or by the site will be reported to the study monitor. See Section 10.2 regarding the Sponsor’s ongoing consideration of updates to guidances and requirements due to COVID-19 to ensure continued protection of subject safety, subject rights, trial integrity, and compliance.

The Sponsor’s designee(s) will be responsible for ensuring that the relevant approval is obtained from the local regulatory authority prior to the start of the study. The relevant documents will be provided to the investigator. The Sponsor’s designee(s) will forward any protocol amendments to the regulatory authority and will ensure that SAEs are reported, and that progress reports and details of any serious protocol violations are provided as required.

Each regulatory authority will be informed should the study be terminated early consistent with local requirements.

18.1 Approval of the Protocol and Amendments

Following authorization by the Sponsor, the final protocol and all related documents (*e.g.*, information sheets and ICFs) will be submitted to the IEC/IRB.

The Sponsor’s designee, Syneos Health, will be responsible for ensuring that regulatory and IEC/IRB approvals are obtained prior to the start of the study. The relevant documents will be provided to the investigator.

Neither the investigator nor the Sponsor will modify this protocol. If modification is necessary, either party must first obtain the concurrence of the other. The party initiating a

[7] International Council for Harmonisation of Technical Requirements for Pharmaceuticals for Human Use (ICH) [homepage on the Internet]. E6(R2): Good Clinical Practice: Consolidated Guideline (or ICH E6(R1) in those jurisdictions where ICH E6(R2) is not yet implemented by the regulatory authorities)

modification will confirm it in writing and the investigator will be responsible for informing the IEC/IRB. In case of a substantial amendment, prior approval of the IEC/IRB is required.

The Sponsor or designee is responsible for the submission of a protocol amendment to the regulatory authority. In the event of a substantial amendment, prior regulatory approval is required for implementation.

18.2 Serious Breaches

It is the responsibility of the Sponsor to notify the licensing authority of any serious breach which is likely to affect, to a significant degree, the safety or mental integrity of the subjects of the study or the scientific value of the study.

All serious breaches will be notified to the pertinent regulatory authorities according to the relevant national regulatory requirement. The reporting will be *via* the Sponsor or delegated party in accordance with TauRx Standard Operating Procedures (SOPs).

18.3 Informed Consent

It is the responsibility of the PI or SI to obtain informed consent from each subject (or his/her LAR who is permitted to provide consent in accordance with local legislation) and study partner(s) participating in this study. At all study sites, the PI or SI who obtains informed consent must be a physician (such as a neurologist, psychiatrist) or other medically qualified person (MD/DO).

Subjects and/or their LAR(s) must give written (signed and personally dated) informed consent prior to study entry and before any study specific procedures are undertaken. The identified study partner(s) for each subject also must provide written consent to his/her own participation as outlined below. Where there is a change of study partner, the new study partner must provide written informed consent.

Potential subjects will be assessed for whether they have capacity to understand the ICF and give consent.

Where possible, fully informed consent will be obtained from the subject. However, subjects entering this study may lack the necessary mental capacity to give fully informed consent. If the potential subject is unable to comprehend the ICF, then one or more LARs will be required to sign the ICF as required by national and/or local law. In this situation, and provided that it is permitted by local legislation, the subject’s agreement to participate in the study will still be obtained to his/her best level of understanding and recruitment will not proceed if the subject refuses or shows signs of significant distress.

Informed consent can be obtained only after it is confirmed that the subject has a likely diagnosis of probable AD or MCI-AD and the aims, methods, anticipated benefits, and known potential hazards of the study have been explained to and discussed with the potential subject and study partner(s) by the investigator. As subjects are not allowed to receive approved AD medication while in the study, they will be informed of the benefits and risks of discontinuing and/or not initiating such therapy, as pertinent. If a subject decides to discontinue AD medication in order to enter the study, a letter will be sent to his/her primary care physician informing him/her of the subject’s decision.

A subject information sheet, providing a written summary of all relevant information, will be given to the potential subject and study partner(s) prior to written informed consent being obtained. The study partner(s) will also be given an information sheet. The information sheets will make clear that access to the subject's medical records will be required. It is the responsibility of the investigator to ensure that the potential subject and study partner(s) are aware of this. The investigator will explain to the potential subject and study partner(s) that they are at liberty to refuse to take part in the study or, should they decide to participate, they may withdraw from the study at any time. Such a decision will not impinge on the future management of the subject. The potential subject and study partner(s) will be allowed as much time as they need to decide whether or not to participate in the study and will be provided with a contact point where further information about the study may be obtained.

The study includes sites in North America and Europe, two geographic regions that maintain descriptions of clinical studies on the internet. As required by the FDA, the ICF must contain the following text: "A description of this clinical trial will be available on http://www.ClinicalTrials.gov, as required by U.S. Law. This Web site will not include information that can identify you. At most, the Web site will include a summary of the results. You can search this Web site at any time." Consistent with European Union law, the ICF also indicates that information will be on https://www.clinicaltrialsregister.eu. Information regarding other national registries will also be included in the ICF, where applicable.

Separate consent may be required by the previous imaging center for transfer of prior amyloid PET scan data to the imaging core laboratory for later confirmation of positive amyloid status.

18.4 Investigator Responsibilities

The primary responsibility of all investigators participating in the study is for the well-being and interests of their subjects, including subjects enrolled in this study. The PI has overall responsibility for the conduct of the trial at his/her study site and may delegate specific duties to appropriately trained members of his/her research team or to other hospital staff, *e.g.*, the pharmacy. Any delegation must be clearly documented in a study site specific delegation list. The PI is responsible for the following:

- Performing the study in accordance with ICH GCP
- Ensuring that adequate time and appropriate resources are available to perform the study as described in this protocol
- Ensuring that all persons assisting with the trial are adequately qualified, trained, and informed about the protocol, trial-related duties, and functions
- Maintaining a list of SIs and other appropriately qualified persons to whom duties have been delegated
- Supervising any individual or party to whom the investigator delegates trial-related duties and functions conducted at the trial site
- Signing an Investigator Agreement to confirm acceptance and willingness to comply with the study protocol
- Maintaining adequate control of study drug and appropriate records of drug disposition
- Maintaining adequate records of each subject's participation

TauRx and the appointed DSMB will constantly evaluate the risk threshold of this particular study by assessing the safety profile as well as assessing the safety profile of other ongoing studies with the same active moiety. Any changes in the risk profile during the course of the study will be communicated to the investigators. In addition, the investigators will review AEs at each visit in accordance with the schedule of assessments, and have the right to interrupt or discontinue study drug for safety reasons as described in Section 11.4.

The burden/distress associated with participation in this study is addressed in the subject information sheet. During the study, the investigators should obtain information from the subjects in order to adequately monitor the degree of burden/distress. Subjects are advised that they have the right to discontinue study drug and withdraw from the study at any time for any reason and should inform the investigators accordingly in order to assist the investigators with monitoring activities.

19 CONFIDENTIALITY AND DATA PROTECTION

All study-related documentation is confidential, whether obtained by the investigator or provided by TauRx or their representative.

The investigator must strictly maintain the confidentiality of subjects in the trial. For all documents and materials submitted to TauRx, its designee, or any electronic system (for example, eCRFs, samples, specimens, and other documents), the investigator must refer to subjects by an identification code. The investigator must keep a separate local log of subject codes, names, and addresses for all subjects, including those that were screened but not enrolled. This local log must never be uploaded to the electronic Trial Master File (eTMF).

Collecting, processing of, and / or transferring data outside the European Economic Area will meet the requirements of EU Directive 2016/679 (General Data Protection Regulation), with appropriate transparency notices included in informed consent documentation. In the United States, data will be protected consistent with Health Insurance Portability and Accountability Act.

Confidentiality of the records identifying each subject shall be maintained. Representatives of the Sponsor, such as monitor(s) or auditor(s), IRB/IEC, and pertinent regulatory authorities will be permitted direct access to these records and other source data/documents as appropriate.

Details of access to the subjects' data will be fully described within the subject information sheet. The consequence of the subject's withdrawal of consent with regards to the use of data will also be described.

For the avoidance of doubt, this protocol does not define or describe any data protection obligations. These will be dealt with in relevant vendor contracts and subject-facing documentation in compliance with relevant local law.

20 STORED SAMPLES AND IMAGING DATA

20.1 Biosamples

TauRx is interested in identifying biomarkers that correlate to a drug's mechanism of action or the disease state under study. Finding appropriate biomarkers can give clinicians a clearer understanding of the most appropriate patients for a particular drug and the most appropriate dose for those patients, offering the potential for improved clinical outcomes.

Storage of samples of plasma and whole blood for possible future research related to determination of potential biomarker predictors of LMTM response or surrogates of treatment response is a mandatory part of this study, unless country-specific laws and regulations prohibit this storage. The samples obtained for genetic testing and the remainder of the samples not used for MT concentration determination as described in Section 15.5 will be stored. Stored samples will retain the subject identifier and, therefore, will not be stored indefinitely. Samples will be stored for a maximum of 8 years after the last subject visit for the study; any sample remaining at that time will be destroyed. Because of the exploratory nature of these analyses and because the results should not change medical management, neither subjects nor investigators will receive the test results.

TauRx's research procedures dictate that a research plan be prepared before conducting any secondary (future) research on stored samples. The research plan would include objectives of the research, scientific rationale for the research, a clear indication that the research is being conducted as secondary research, how alternative authorization to use and disclose the data from secondary research is to be obtained, and how the results are intended to be used. For this study, future research could include, but need not be limited to, measurement of plasma markers of tau and other protein species, genetic and epigenetic markers which might be predictors of LMTM response, or potential surrogate markers of treatment response. Analysis of these data could provide an important, minimally invasive biomarker that would guide future decisions by clinicians and researchers.

20.2 Imaging Data

Additional exploratory analyses are to be undertaken of the blinded ^{18}F-FDG-PET and MRI data generated as part of this study. The analyses will be for scientific research and quality control purposes which are separate from the study objectives. A separate plan will capture standard procedures and a data transfer agreement will detail the requirements for data to be transferred to the imaging center performing the analyses. Data analyses will be conducted in line with required data protection requirements, will not reveal potentially sensitive information about the study or its participants, and will not lead to potential unblinding of study data. The transfer and use of these data will be described in the informed consent form.

21 QUALITY ASSURANCE AND CLINICAL MONITORING

21.1 Standard Procedures

SOPs will be adhered to for all activities relevant to the quality of the study, including protocol compliance, data collection, quality control, and data analyses and reporting.

QA audits will be conducted on critical phases during the clinical and reporting phases of the study. These audits will be carried out by QA personnel, independent of the staff involved in the study, according to relevant SOPs.

Clinical monitoring, both primary and secondary, will be performed by trained clinical research personnel. Clinical monitoring is an integral part of controlling and securing of data integrity and subject safety. The first monitoring visit will be scheduled appropriately after the first subject is screened at a site depending on factors that could impact on data reliability, some of which are mentioned below. The average monitoring frequency will be described in a CMP and will depend on a number of factors, including subject screening and recruitment rates, site performance, and quality adherence. Regulatory recommendations and guidelines will be followed. Detailed expected monitoring activity will be described in the CMP, which will be modified on an ongoing basis to ensure subject safety and data integrity.

The monitor will ensure compliance with the protocol, adherence to regulatory and ICH obligations, accurate reporting of AEs, maintenance of trial records including drug accountability records, and correct administration of study procedures including supply and storage of study materials. ICFs will be reviewed to verify that they are correctly signed and dated by the subject and study partner and the medically qualified PI or SI. At each monitoring visit, subject data will be reviewed and verified against the medical records.

The monitor will require direct access to laboratory test results and other records needed to verify entries on the eCRF.

The investigator (or his/her designated deputy) agrees to cooperate with the monitor and other clinical research personnel to ensure that any problems detected in the course of these monitoring visits are quickly resolved.

Quality review of data and/or trial documentation may be carried out by or on behalf of the Sponsor at any stage. Audits of study sites and/or trial processes may be carried out at any stage.

21.2 COVID-19 Risk Assessment

Due to the impact of COVID-19 on this study, the Sponsor has conducted risk assessments to identify the potential risk levels and impacts of COVID-19, as well as associated mitigating actions for a number of clinical study aspects, including: direct-to-subject study drug shipments, missed study visits and/or clinical assessments, handling of missed post-baseline study data, remote site initiation visits, remote monitoring visits, and remote clinical assessments (*e.g.*, remote ADAS-cog$_{13}$). Vendors have also conducted risk assessments associated with their services to this study. Risk assessment and management are ongoing processes for this study; all risks and mitigations are regularly reviewed and managed, and the Sponsor will continue to ensure that additional risk assessments are carried out and documented, in particular for any potential impact due to COVID-19, as the situation develops and as regulatory guidances are updated.

A summary of alternate arrangements to clinical monitoring that may be implemented due to COVID-19 is provided in Section 10.2.2.

22 DOCUMENTATION

The following documents must be provided before or at site initiation:

- Protocol and amendments (if applicable) signed and dated by applicable Sponsor representatives, as well as by the investigator
- Regulatory approval (or in absence of document, evidence that study may proceed)
- Signed and dated IEC/IRB approval
- Approved subject and study partner information sheets, ICF, and advertisement for recruitment (if any)
- eCRFs
- Confidentiality agreement(s)
- Financial disclosure
- Signed *curricula vitae* for personnel who have signed the authorized delegation log (including PI, all SIs, and designated assistants)
- Authorized signature log/delegation list
- Investigator’s Brochure with signed and dated Investigator’s Brochure receipt
- Signed and dated clinical trial agreement
- Research and development (or institution) approval, if applicable
- Signed and dated indemnity/insurance statement (if applicable)
- Laboratory reference ranges and accreditation for all applicable laboratories (central and local, as applicable)
- Pharmacy agreement (if any)
- Imaging center agreement (if any)
- SAE forms

The protocol, its amendments, and any other required documents must be submitted for appropriate regulatory review and approval.

The investigator at each study site must generate and maintain adequate records (medical records, source documents, and eCRFs) to enable the conduct of this study to be fully documented. The eCRF may serve as the primary collection medium for any data (to be agreed with the investigator and documented in the Source Document Agreement). Each enrolled subject must have an eCRF completed and this must be reviewed and approved by the investigator.

A record must be kept of all subjects consenting for the study and subsequently excluded. The reason for non-participation in the study should be recorded.

The documents specified by ICH GCP (*e.g.*, copies of protocols, CRF pages, original copies of test results, reports, drug dispensing logs, correspondence, records of informed consent, and other documents pertaining to the conduct of the study) must be kept on file by the investigator for a minimum of 25 years after the end of the clinical trial or for the period of time specified by local law for the preservation of hospital subject documents, whichever is the longest. No study documents should be destroyed without prior written agreement between TauRx and the investigator. Should the site wish to assign the study records to another party, or move them to another location, TauRx must be informed.

The study eTMF will be maintained as specified by ICH GCP. The eTMF shall be archived for a minimum of 25 years after the end of the clinical trial in a way that ensures that it is readily available, upon request, to the regulatory authorities.

23 PUBLICATION

Since this is a multicenter study, the community of investigators and delegated individual investigators shall not publish any partial results before the end of the study or before the analysis and publication of the results of the entire study.

The investigator and/or institution shall have the right to publish, display, or otherwise communicate orally, in writing, or electronically (hereafter a "publication") the results of his/her work conducted under this protocol after 12 months from a New Drug Application or equivalent filing, or earlier only with explicit consent of the Sponsor in advance and in writing.

Sites and/or investigators must provide the Sponsor with the opportunity to review the contents of any proposed abstract or publication concerning the work, including any results of the study, in advance of publication, and agree to delay the publication if, in the Sponsor's reasonable view, the publication may prejudice the Sponsor's intellectual property. The Sponsor will make every reasonable effort to consider and release each proposed abstract or publication within 60 days of submission. The investigator and/or site will include, where possible, comments made by the Sponsor. Authorship will be determined by mutual agreement. Access to data will be in accordance with authorship.

24 INDEMNITY, INSURANCE, AND COMPENSATION

A clinical trials insurance and product liability insurance policy will be in place to cover the conduct of this study.

25 ADMINISTRATIVE AND FINANCIAL AGREEMENT

Agreed costs for each participating study site will be met by TauRx. For each study site, an agreement will be prepared and signed off by the relevant authority on behalf of the institution (*e.g.*, National Health Service Trust, University) and by TauRx or its designee before the initiation of the trial. Each PI and SI must also sign a Form FDA 3455 or its equivalent to disclose any financial arrangements or interests.

Subjects will be reimbursed by TauRx, through the investigator, for reasonable travel costs to and from the study site and accommodation in certain circumstances by prior agreement with the Sponsor.

26 STUDY ADMINISTRATION

This trial will be conducted in compliance with ICH GCP and other applicable regulatory requirements.

Vendors and/or independent contract personnel will be contracted to manage and monitor the trial; to provide services for data management and statistical analysis; to provide regulatory advice and services; to handle the reporting of SAEs; to provide services for laboratory, imaging, and PK analysis; to package and distribute the clinical trial supplies; and to provide QA support and services.

Calibration certification for the following equipment maintained by the site and used to generate study data will be confirmed: ECG machines, refrigerated centrifuges, and pharmacy temperature loggers.

27 REFERENCES

Aduhelm FDA Drug Approval Package, Office of Neurology's Summary Review Memorandum. June 2021.

Al-Hilaly YK, Pollack SJ, Vadukul D, *et al.*: Alzheimer's disease-like paired helical filament assembly from truncated tau protein is independent of disulphide cross-linking. J Mol Biol 2017;429:3650-65.

Al-Hilaly YK, Pollack SJ, Rickard JE, *et al.*: Cysteine-independent inhibition of Alzheimer's disease-like paired helical filament assembly by leuco-methylthioninium (LMT). J Mol Biol 2018; https://doi.org/10.1016/j.jmb.2018.08.010.

Albert MS, DeKosky ST, Dickson D, *et al*. The diagnosis of mild cognitive impairment due to Alzheimer's disease: Recommendations from the National Institute on Aging-Alzheimer's Association workgroups on diagnostic guidelines for Alzheimer's disease. Alzheimers Dement 2011; 7(3):270-9.

Baddeley TC, McCaffrey JM, Storey JM, *et al*. Complex disposition of methylthioninium redox forms determines efficacy in tau aggregation inhibitor therapy for Alzheimer's disease. J Pharmacol Exp Ther 2015; 352(1):110-18.

Berg L, Miller JP, Storandt M, *et al.* Mild senile dementia of the Alzheimer type: 2. Longitudinal assessment. Ann Neurol 1988; 23(5):477-84.

Bierer LM, Hof PR, Purohit DP, *et al*. Neocortical neurofibrillary tangles correlate with dementia severity in Alzheimer's disease. Arch Neurol 1995; 52(1):81-8.

Birks JS, Harvey R. Donepezil for dementia due to Alzheimer's disease. Cochrane Database of Systematic Reviews, Issue 3, 2004. Article No. CD001190.

Bowie P, Branton T, Holmes J. Should the Mini Mental State Examination be used to monitor dementia treatments? Lancet 1999; 354(9189):1527-8.

Braak H, Braak E. Neuropathological staging of Alzheimer-related changes. Acta Neuropathol 1991; 82(4): 239-59.

Courtney C, Farrell D, Gray R, et al. AD2000 Collaborative Group. Long-term donepezil treatment in 565 patients with Alzheimer's disease (AD2000): Randomised double-blind trial. *Lancet*. 2004;363:2105-15

Fillenbaum GG, Peterson B, Morris JC. Estimating the validity of the Clinical Dementia Rating Scale: the CERAD experience. Aging (Milano) 1996; 8(6):379-85.

Fitzpatrick AWP, Falcon B, He S, *et al.*: Cryo-EM structures of tau filaments from Alzheimer's disease. Nature 2017; 547:185-90.

Folstein MF, Folstein SE, McHugh PR. "Mini-mental State": A practical method for grading the cognitive state of patients for the clinician. J Psychiatr Res 1975; 12(3):189-98.

Friston K, Ashburner J, Kiebel S, *et al.* (eds) *Statistical Parametric Mapping: The analysis of functional brain images*. 1st ed. Amsterdam: Academic Press; 2007.

Galasko D, Bennett D, Sano M, *et al.* An inventory to assess activities of daily living for clinical trials in Alzheimer's disease. The Alzheimer's Disease Cooperative Study. Alzheimer Dis Assoc Disord 1997; 11(Suppl 2):S33-9.

Galasko D, Schmitt F, Thomas R, *et al.* Detailed assessment of activities of daily living in moderate to severe Alzheimer's disease. J Int Neuropsychol Soc 2005; 11(4):446-53.

Gauthier S, Feldman HH, Schneider LS, *et al*. Efficacy and safety of tau-aggregation inhibitor therapy in patients with mild or moderate Alzheimer's disease: a randomised, controlled, double-blind, parallel-arm, phase 3 trial. Lancet 2016; 388:2873-84.

Hughes CP, Berg L, Danziger WL, *et al.* A new clinical scale for the staging of dementia. Br J Psychiatry 1982; 140:566-72.

Lockhart SN, Baker SL, Okamura N, *et al*. Dynamic PET measures of tau accumulation in cognitively normal older adults and Alzheimer's disease patients measured using ^{18}F THK-5351. PLoS One 2016; 11(6):e0158460.

McKhann GM, Knopman DS, Chertkow H, *et al*. The diagnosis of dementia due to Alzheimer's disease: Recommendations from the National Institute on Aging-Alzheimer's Association workgroups on diagnostic guidelines for Alzheimer's disease. Alzheimers Dement 2011; 7(3):263-9.

Mohs RC, Knopman D, Petersen RC, *et al*. Development of cognitive instruments for use in clinical trials of antidementia drugs: additions to the Alzheimer's Disease Assessment Scale that broaden its scope. The Alzheimer's Disease Cooperative Study. Alzheimer Dis Assoc Disord 1997;11(Suppl 2):S13–21.

Morris JC, McKeel DW Jr, Fulling K, *et al.* Validation of clinical diagnostic criteria for Alzheimer's disease. Ann Neurol 1988; 24(1):17-22.

Morris JC, Ernesto C, Schafer K, *et al.* Clinical Dementia Rating training and reliability in multicenter studies: the Alzheimer's Disease Cooperative Study experience. Neurology 1997; 48(6):1508-10.

Mukaetova-Ladinska EB, Garcia-Siera F, Hurt J, *et al*. Staging of cytoskeletal and β-amyloid changes in human isocortex reveals biphasic synaptic protein response during progression of Alzheimer's disease. Am J Pathol 2000; 157(2):623-36.

National Institute for Clinical Excellence (NICE) Technology Appraisal Guidance No 19. Guidance for the use of donepezil, rivastigmine and galantamine for the treatment of Alzheimer's disease 2001, London: NICE.

Olin J, Schneider L. Galantamine for dementia due to Alzheimer's disease. Cochrane Database of Systematic Reviews, Issue 3, 2002, article No. CD001747.

Pontecorvo MJ, Devous MD, Sr., Navitsky M, *et al.* Relationships between flortaucipir PET tau binding and amyloid burden, clinical diagnosis, age and cognition. Brain 2017; 140:748-63.

Reisberg B, Doody R, Stöffler A, *et al*. Memantine in moderate-to-severe Alzheimer's disease. N Engl J Med 2003; 348(14):1333-41.

Riedel G, Klein J, Niewiadomska G, et al. Mechanisms of anticholinesterase interference with tau aggregation inhibitor activity in tau-transgenic mouse model. Curr Alzheimer Res. 2020;17(3):285-296.

Rosen WG, Mohs RC, Davis KL. A new rating scale for Alzheimer's disease. Am J Psychiatry 1984; 141(11):1356-64.

Schelter BO, Shiells H, Baddeley TC, *et al*. Concentration-dependent activity of hydromethylthionine on cognitive decline and brain atrophy in mild to moderate Alzheimer's disease. J Alzheimers Dis 2019;72(3):931-46

Schneider LS, Dagerman KS, Higgins JP, *et al*. Lack of evidence for the efficacy of memantine in mild Alzheimer disease. Arch Neurol 2011; 68(8):991-8.

Staff RT1, Murray AD, Deary IJ, *et al*. What provides cerebral reserve? Brain 2004; 127(Pt 5):1191-9.

Tombaugh TN, McIntyre NJ. The Mini-Mental State Examination: A comprehensive review. J Am Geriatr Soc 1992; 40(9):922-35.

Wilcock GK, Gauthier S, Frisoni GB, *et al*. Potential of low dose leuco-methylthioninium bis(hydromethanesulphonate) (LMTM) monotherapy for treatment of mild Alzheimer's disease: Cohort analysis as modified primary outcome in a Phase III clinical trial. J Alzheimer's dis 2018; 61:435-57.

Wischik CM, Lai RY, Harrington CR. Modelling prion-like processing of tau protein in Alzheimer's disease for pharmaceutical development. In: Brain Microtubule Associated Proteins: Modifications in Disease. Eds. Avila J, Brandt R, Kosik KS 1997. Amsterdam: Harwood Academic Publishers, 185-241.

Wischik CM, Novak M, Edwards PC, *et al.*: Structural characterization of the core of the paired helical filament of Alzheimer disease. Proc Natl Acad Sci USA 1988a; 85:4884-8.

Wischik CM, Novak M, Thøgersen HC, *et al.*: Isolation of a fragment of tau derived from the core of the paired helical filament of Alzheimer disease. Proc Natl Acad Sci USA 1988b; 85:4506-10.

Wischik CM, Staff RT, Wischik DJ, *et al*. Tau aggregation inhibition therapy: an exploratory Phase 2 study in mild or moderate Alzheimer's disease. J Alzheimer's Dis 2015; 44(2):705-20.

Xia CJ, Makaretz SJ, Caso C, *et al*. Association of in vivo [^{18}F] tau PET imaging results with cortical atrophy and symptoms in typical and atypical Alzheimer disease. JAM Neurol 2017; 74:427-36.

28 APPENDICES

28.1 Disallowed Treatments and Windows

28.1.1 Disallowed Medical Food / Medications Beginning 90 Days Before Baseline and During the Study

Medical food
Souvenaid®

Antipsychotic
Clozapine

Antiepileptic drugs
Carbamazepine
Primidone
Valproate

Anti-malarial drugs
Dapsone
Primaquine and related antimalarials

Anesthetics
Benzocaine or other local anesthetics used chronically

Investigational product for cognition (unless confirmed to have been randomized to placebo)

28.1.2 Disallowed Medications Beginning 60 Days Before Baseline and During the Study

Alzheimer's disease drugs
Donepezil
Galantamine
Rivastigmine
Memantine

28.1.3 Disallowed Treatments Beginning 28 Days Before Baseline and During the Study

Investigational drug (other than for cognition), biologic, device, or medical food

28.2 Study Blood Assessments

Please see schedule of assessments for timing of blood sample collections.

Clinical Chemistry

Sodium
Potassium
Chloride
Bicarbonate
Total protein
Albumin
Calcium
Phosphorus
Glucose
Blood urea nitrogen
Creatinine
Indirect bilirubin
Direct bilirubin
Total bilirubin
Alkaline phosphatase
Alanine aminotransferase
Aspartate aminotransferase
Gamma-glutamyl transferase
Lactate dehydrogenase

Hematology

Red blood cell (RBC) count
Hemoglobin
Hematocrit
MCV, MCH, MCHC
RBC distribution width
Platelet count
White blood cell (WBC) count
WBC Differential (% and absolute)
- Neutrophils
- Eosinophils
- Basophils
- Lymphocytes
- Monocytes

Reticulocytes (absolute and relative)

Other Tests

Serum Pregnancy test
Vitamin B_{12} / Folate
Haptoglobin
Thyroid Stimulating Hormone (TSH)
Glucose-6-Phosphate Dehydrogenase (G6PD)
ApoE (in subjects who consent for this)
Blood sample for MT concentration

28.3 Education / Occupation

28.3.1 Education

Q: How many years did you spend in full time education before the age of 26?

(integer)_ years

Q: How would you best describe your level of education?

1. Elementary
2. Some high school / secondary
3. Completed high school / secondary
4. Some post high school / secondary education
5. College education graduate
6. Post graduate education (including high professional qualification)

28.3.2 Occupation

Q: What was your main occupation during your working life?

(text)

(Responses will be coded using The Standard Operational Classification, 2010, Volume 2 The Coding Index, UK Office for National Statistics)

28.4 Summary of Changes to the Protocol

28.4.1 Protocol Version 2.0

The protocol for study TRx-237-039 (Version 1.0 dated 23 Aug 2017) has been revised (Version 2.0) to clarify the assessments that will be made at various visits as follows:

- The blood will be collected during Visit 2 and Visit 3 (not at Visit 2 *or* Visit 3).
- A spelling error was corrected (dimethyl to desmethyl).
- Clarification was made that the *time of dose* from the previous day will be collected.
- A redundant paragraph was removed.
- Specifications for procedure of blood collection were removed from the protocol as these details will be presented in the laboratory manual.
- The Appendices were renumbered and the table of contents updated.
- Minor edits, such as expansion of abbreviations, were made.

The sections affected by these changes are detailed below.

Summary of Changes	Affected Section(s) in Revised Protocol (Version 2.0)
Targeted examinations will be performed pre-dose and approximately 3 hours after administration of first dose of study drug (Visit 2); these are to be repeated as needed for subjects who remain in the clinic longer than 4 hours. Thereafter, targeted examinations are to be performed at each subsequent visit or upon early termination.	• Synopsis, Safety and tolerability<br>• Table 10-1 Schedule of Assessments, foot note ‘f’<br>• Section 15.2, Bullet #7
Added the following missing bullet to the Synopsis:<br>TSH, vitamin B_{12}, folate, haptoglobin and G6PD will be measured at Screening only. Standard clinical laboratory testing, including hematology, blood chemistry, will be performed at Screening and each visit thereafter including an early termination, if applicable. A blood sample for a serum pregnancy test will be collected for all women of childbearing potential at Screening and at each subsequent visit, or upon early termination.	• Synopsis, Safety and tolerability
Several updates were made to the various statistical analyses planned	• Section 17.8 including subsections
The blood will be collected during Visit 2 and Visit 3 (not at Visit 2 *or* Visit 3)	• Table 10-1 Schedule of Assessments, foot note ‘q’
A redundant bullet was removed	• Synopsis, Safety and Tolerability<br>• Section 15.2
Spelling changed from dimethyl to desmethyl	• Synopsis, safety and tolerability, Bullet #5<br>• Section 15.2 Bullet #5<br>• Section 15.5.2, line 2

Summary of Changes	Affected Section(s) in Revised Protocol (Version 2.0)
The *time of dose* from the previous day will be collected	• Table 10-1 Schedule of Assessments, foot note ‘q’<br>• Section 15.4.1 Paragraph 1
The volume of whole blood, centrifuge speed and the number and type of blood collection tubes were removed	• Section 15.4.1.1
The Appendices were renumbered and the Table of contents updated	• Table of Contents<br>• Appendices 30.3 and 30.3.1

28.4.2 Protocol Version 2.1

The protocol for study TRx-237-039 (Version 2.0 dated 31 Aug 2017) was updated to make administrative changes to provide the latest contact details of the Sponsor and the Sponsor’s personnel, and the details of the Central Laboratory.

Summary of Changes	Affected Section(s) in Revised Protocol (Version 2.1)
The address and phone numbers of the Sponsor and the Sponsor’s personnel was updated.	• Cover page<br>• Section 3 Protocol Approval<br>• Section 4 Responsible Personnel
Version number was changed from 2.0 to 2.1 and date of the protocol was changed from 31 Aug 2017 to 25 Oct 2017.	• Cover page<br>• Headers<br>• Footers<br>• Section 5 Investigator Signature Sheet
The Central Laboratory name, address and phone numbers were added.	• Section 4 Responsible Personnel

28.4.3 Protocol Version 3.0

The protocol for Study TRx-237-039 (Version 2.1 dated 25 October 2017) was updated to modify the study design to a 39-week treatment period (from 26 weeks) in subjects with early AD (*i.e*., by the inclusion of subjects with MCI-AD in addition to mild AD). As a result of the addition of a cognitive/functional Composite Scale (to be finalized prior to database lock and unblinding) and a third treatment arm (LMTM 16 mg/day), the sample size has been increased to approximately 375 subjects (from 180 subjects). Numerous changes have been made to the protocol to clarify and/or specify the timing and sequence of assessments.

A summary of the key changes and primary affected sections is provided below (as the Synopsis is affected by the majority of the changes, it is not listed).

Administrative changes have also been made, such as adding the EudraCT number issued for this study (2017-003558-17); updating references to the 2013 (current) version of the Declaration of Helsinki, the ICH Integrated Addendum to ICH E6(R2) Guidelines for GCP, and GDPR; and updating parties and contact information for responsible parties in Section 4. Additional statistical analyses, including of derived endpoints (such as the Composite Scale) and the higher treatment group, are now described. While a brief summary of planned statistical analyses of primary and secondary endpoints is described and a list of possible exploratory analyses is included, these will be finalized in a Statistical Analysis Plan prior to database lock and unblinding. These are not described below but are enumerated in a separate detailed summary of changes document.

Summary of Changes	Primary Affected Sections in Revised Protocol (Version 3.0)
Background	
The recently adopted United States Abbreviated Names of hydromethylthionine (LMT) and hydromethylthionine mesylate (LMTM, drug substance) have been added	Section 8.1.1 Investigational Product<br>Section 12.1.1 Active Ingredient
Nonclinical and clinical data have been updated to be reflective of most current version of Investigator’s Brochure (v. 20.0); references have been updated as pertinent	Section 8.1.2 Nonclinical Data<br>Section 8.1.3 Clinical Data
Objectives	
A new clinical efficacy scale has been added as a gated primary objective; this Composite Scale is to be derived from cognitive/functional items selected from the ADAS-cog_{11} and ADCS-ADL_{23}	Section 8.2 Rationale<br>Section 9.1 Primary Objectives<br>Section 15.3.3 Composite Scale<br>Section 17 Statistical Analysis<br>Section 17.1.2 Primary Efficacy Endpoint<br>Section 17.2 Number of Subjects and Sample Size Calculation<br>Section 17.4.1 Hypothesis<br>Section 17.4.7 Subgroup Analyses<br>Section 17.4.8.4.1 Multiple Imputation<br>Section 17.5 Exploratory Analyses
The secondary clinical endpoint has been changed from the ADAS-cog_{11} to the ADAS-cog_{13} as potentially more sensitive to change in subjects with milder disease	Section 9.2 Secondary Objectives<br>10.4.2.2 Clinical Efficacy Assessments<br>15.3.1 ADAS-cog<br>17.1.4 Other Secondary Endpoints<br>17.5 Exploratory Analyses

Summary of Changes	Primary Affected Sections in Revised Protocol (Version 3.0)
The sequence of the secondary objectives has been modified and the examination of associations between the ^{18}F-FDG-PET regions of interest and the Composite Scale and the examination of associations between brain MRI endpoints and ADAS-cog$_{13}$, ADCS-ADL$_{23}$, and the Composite Scale have been added. Comparison of the LMTM 16 mg/day and placebo groups has also been added.	Section 9.2 Secondary Objectives<br>Section 17.1.3 Secondary Imaging Endpoints<br>Section 17.1.4 Other Secondary Endpoints
Exploratory analyses and exploratory analyses that are comparisons with external data are now listed only in the Statistical Analysis section.	Section 9.3 (*Section removed*)<br>Section 9.4 (*Section removed*)<br>Section 17.5 Exploratory Analyses
Study Design	
The duration of the study has been increased from 6 to 9 months; the numbers of visits, schedules of safety and efficacy assessments, and statistical analyses have also been updated accordingly.	Section 10.1 General Description<br>Section 10.3 Duration<br>Section 10.4.2 Baseline and Post-randomization Assessments<br>Section 10.4.2.1 Imaging Efficacy Assessments<br>Section 10.4.2.2 Clinical Efficacy Assessments<br>Section 10.4.2.3 Safety Assessments<br>Section 12.2 Study Regimens<br>Section 15.2.3 Imaging Methods for Efficacy<br>Section 15.3 Clinical Assessments and Raters<br>Section 15.4 Safety Assessment and Procedures<br>Section 17 Statistical Analysis
Total study duration for an individual subject has been increased from 36 weeks to up to 54 weeks, including a Screening period of up to 15 weeks (105 days) and a Treatment period of 39 weeks, which is updated throughout the protocol. As a result, the total duration of the overall study has been increased to at least 24 months, depending on recruitment rate.	Section 10.1 General Description<br>Section 10.3 Duration
A third treatment arm, LMTM 16 mg/day, has been added and the total sample size increased from 180 subjects (90 each randomized to placebo and LMTM 8 mg/day) to 375 subjects (150 each randomized to placebo and LMTM 8 mg/day and 75 randomized to LMTM 16 mg/day). As many as 900 subjects may be screened (increased from 360).	Section 10.1 General Description<br>10.2 Study Population<br>17.2 Number of Subjects and Sample Size Calculation
The follow-up telephone contact, previously scheduled to occur 4 weeks after the last dose of study drug, has been deleted; the protocol clarifies that investigators are to follow any unresolved adverse events to resolution or acceptable stabilization consistent with their medical judgment.	Section 10.1 General Description<br>Section 10.4.2 Baseline and Post-randomization Assessments<br>Section 10.4.2.3 Safety Assessments<br>Section 11.4 Discontinuations / Withdrawals<br>Section 15.4 Safety Assessment and Procedures<br>Section 16.2 Eliciting Adverse Event Information<br>Section 16.7 Serious Adverse Event Reporting
Patient Population (Inclusion and Exclusion Criteria)	
The patient population has been expanded to include subjects with mild cognitive impairment due to Alzheimer's disease (MCI-AD), such that the study is now being performed in subjects with early Alzheimer's disease (AD) (inclusive of both mild AD and MCI-AD)	Section 8.2 Rationale<br>Section 9 Objectives<br>Section 10.1 General Description<br>Section 10.2 Study Population<br>Section 10.4.1.1 Diagnostic and Cognitive Eligibility Assessments<br>Section 11.1 Inclusion Criteria

Summary of Changes	Primary Affected Sections in Revised Protocol (Version 3.0)
Subjects must be able to give their own consent; consent by a caregiver on behalf of the subject is no longer allowed	Section 9.2 Secondary Objectives<br>Section 10.4.1 Screening Assessments<br>Section 10.4.2 Baseline and Post-randomization Assessments<br>Section 10.4.3 Other Assessments<br>Section 11.1 Inclusion Criteria<br>Section 15.5.3 Genotyping<br>Section 18.3 Informed Consent
A PET scan positive for amyloid is now required for eligibility (either by prior documentation or undertaken for the study)	Section 10.1 General Description<br>Section 10.2 Study Population<br>Section 10.4.1 Screening Assessments<br>Section 10.4.1.1 Diagnostic and Cognitive Eligibility Assessments<br>Section 11.1 Inclusion Criteria<br>Section 10.4.1 Screening Assessments<br>Section 15.1 Demographic Data/Medical History<br>Section 15.2.1 General Considerations<br>Section 15.2.3.1 ^{18}F-FDG-PET<br>Section 18.3 Informed Consent
The acceptable disease severity based on MMSE has been expanded from 20-25 to 20-27; randomization will now be stratified based on a MMSE of 20-25 and 26-27 (in a 2:1 ratio, monitored and controlled at the site level and capped as needed at the study level)	Section 10.1 General Description<br>Section 10.2 Study Population<br>Section 11.1 Inclusion Criteria<br>Section 11.3 Re-screening<br>Section 12.3 Randomization<br>Section 17.4.7 Subgroup Analyses
Screening Global CDR score of 0.5 now has a further requirement that subjects have a score of >0 in one of the functional domains (Community Affairs, Home and Hobbies, or Personal Care)	Section 10.2 Study Population<br>Section 11.1 Inclusion Criteria<br>Section 11.3 Re-screening
Subjects taking an AChEI and/or memantine at Screening may now be enrolled, provided they are willing to discontinue such medication; the screening period in such subjects has been expanded to 15 weeks to allow for the requisite eligibility assessments prior to the discontinuation of such medication (at least 60 days before Baseline ^{18}F-FDG-PET); if subjects discontinue these medications, a letter is to be sent to the primary care physicians to inform them of this decision	Section 10.1 General Description<br>Section 10.4.1 Screening Assessments<br>Section 10.4.1.1 Diagnostic and Cognitive Eligibility Assessments<br>Section 10.4.2 Baseline and Post-randomization Assessments<br>Section 11.1 Inclusion Criteria<br>Section 13.1 AChEI and/or Memantine
As a result of the increased sample size, at least 100 sites may be required (increased from 60) and sites from geographical regions outside of North America and Europe may also be sought	Section 10.1 General Description<br>Section 10.2 Study Population<br>Section 12.3 Randomization<br>Section 17.4.7 Subgroup Analyses
Additional exclusion criteria:<br>• Any physical disability that would prevent completion of study procedures or assessments (with examples provided)<br>• Use of Souvenaid® within the 90 days prior to Baseline	Section 11.2 Exclusion Criteria

Summary of Changes	Primary Affected Sections in Revised Protocol (Version 3.0)
Modifications of exclusion criteria include:<br>• Moderate to severe sleep apnea, even if adequately controlled (previously, subjects who were controlled were accepted)<br>• Carbamazepine is not allowed (previously, use for treatment of restless legs was permitted)<br>• G6PD deficiency has been defined based on WHO classification (<60% of normal, *i.e.*, <6.1 U/g Hgb)<br>• Use of olanzapine is no longer exclusionary (the only disallowed antipsychotic is clozapine)<br>• Need for a cardiology consult in subjects with left bundle branch block has been removed<br>• The acceptable window for prior use of an investigational product for cognition has been reduced to 90 days (from 120 days) prior to Baseline (rather than Screening)	Section 11.2 Exclusion Criteria<br>Appendix 28.1
Study Drug	
With the addition of a third treatment arm, all subjects will now receive four tablets daily, two in the morning and two in the evening, to maintain the study blind.	12.2 Study Regimens
The study drug wallet now contains 28 tablets (rather than 14 tablets)	12.4 Packaging, Labeling, and Storage
Assessments	
Information regarding education and main occupation will now be collected at Screening	Section 15.1 Demographic Data / Medical History<br>Section 17.6 Demographic and Baseline Characteristics<br>Appendix 28.3 Education / Occupation
In subjects with extended Screening, medical assessments for eligibility may need to be repeated if initial assessments were more than 42 days prior to Baseline	Section 10.4.1 Screening Assessments<br>Section 10.4.1.2 Other Medical Screening Assessments
Brain MRI hyperintensities will now be quantified	Section 10.4.2.1 Imaging Efficacy Assessments<br>Section 15.2.3.2 MRI<br>Section 17.5 Exploratory Analyses
MMSE and CDR sum of boxes (which are obtained at Screening) will now also be assessed after 39 weeks, or upon early completion of the study	Section 10.1 General Description<br>Section 10.4.2 Baseline and Post-randomization Assessments<br>Section 10.4.2.2 Clinical Efficacy Assessments<br>Section 15.3 Clinical Assessments and Raters<br>Section 17.5 Exploratory Analyses
Whole blood will also be assessed for MT, *N*-desmethyl MT, and total MT concentrations (in addition to plasma)	Section 10.1 General Description<br>Section 10.4.2 Baseline and Post-randomization Assessments<br>Section 15.5.1 MT Concentration<br>Section 15.5.1.1 Procedure for Blood Sample Collection<br>Section 17.8 Other Data
A urine sample will be collected at the final visit (following all safety and efficacy assessments) for determination of color by the central laboratory; results will not be reported to the site	Section 10.1 General Description<br>Section 10.4.3 Other Assessments<br>Section 15.5.2 Urine Color<br>Section 17.8 Other Data
Other	
Malignancies (with the exception of non-melanoma skin cancers) are to be to be reported as ADRs to Health Canada	Section 16.9 Malignancies<br>Section 17.7 Safety Analysis

Summary of Changes	Primary Affected Sections in Revised Protocol (Version 3.0)
Stored samples may be kept for up to 8 years for future, as yet unspecified, analyses; consent to such storage is mandatory for participation in the study	Section 20 Stored Samples

28.4.4 Protocol Version 4.0

The protocol for Study TRx-237-039 (Version 3.0 dated 31 May 2018) has been updated to include administrative changes, as the responsible party for study management, monitoring, and pharmacovigilance has changed (previously PAREXEL International, now Syneos Health). Additional modifications and clarifications have been incorporated with respect to the Sponsor contact details, objectives and statistical analyses, background, study population, informed consent, study assessments, documentation, data protection, and study administration.

A summary of the key changes and affected sections is provided below.

Summary of Changes	Affected Sections in Revised Protocol (Version 4.0)
Sponsor Information	
The contact details for the Sponsor have been clarified to indicate that the company headquarters are in Singapore, while the operational location is in the United Kingdom.	Cover page
Compliance Statement	
Compliance statement regarding ICH GCP further clarified to include ICH E6(R1) in those jurisdictions where ICH E6(R2) is not yet implemented by the regulatory authorities. Reference to the Declaration of Helsinki has been clarified to refer to the current applicable version.	Section 2 GCP Compliance Statement<br>Section 5 Investigator Signature Sheet<br>Section 18 Regulatory and Ethics
Responsible Personnel	
Changes have been made to include updated contact information for the Global Project Manager, North America Medical Monitor, Europe Medical Monitor, and Pharmacovigilance, as the responsible party is now Syneos Health (previously PAREXEL International); the procedures for safety and regulatory/ethics reporting have been updated accordingly. Additional telephone numbers have been added to the list of DSSL 24-hour Medical Contacts, as the study will also be conducted in Italy and Spain. The vendor for recruitment services (ThreeWire) has been removed.	Section 4 Responsible Personnel<br>Section 16.7 Serious Adverse Event Reporting<br>Section 16.9 Malignancies<br>Section 16.10 Reporting of Pregnancy<br>Section 18 Regulatory and Ethics<br>Section 18.1 Approval of the Protocol and Amendments
Objectives and Statistical Analyses	
The evaluation of the outcomes described in the Primary and Secondary Objectives for LMTM 16 mg/day and placebo has been clarified in Objective Nos. 6 and 12, and an evaluation of the outcomes of selected endpoints for pooled doses of LMTM 8 mg/day and 16 mg/day compared to placebo has been added as Objective No. 13. The various LMTM 8 mg/day as well as LMTM 16 mg/day and pooled comparisons to placebo are described in the SAP.	Synopsis<br>Section 9.2 Secondary Objectives<br>Section 17 Statistical Analysis
Background	
A new reference has been added regarding cysteine-independent inhibition of Alzheimer's disease-like paired helical filament assembly by leuco-methylthioninium (Al-Hilaly *et al.*, 2018).	Section 8.1.1 Investigational Product<br>Section 27 References
The summary of PK data from studies in subjects with renal and hepatic impairment has been clarified; results have been added from a completed study in healthy older volunteers.	Section 8.1.3.1 Pharmacokinetics

Summary of Changes	Affected Sections in Revised Protocol (Version 4.0)
Study Population	
Up to approximately 1500 subjects may be screened for the study (increased from 900).	Section 10.2 Study Population
Informed Consent	
Clarification that at all study sites, the PI or SI who obtains informed consent must be a physician (such as a neurologist, psychiatrist) or other medically qualified person.	Section 10.4.1 Screening Assessments<br>Section 18.3 Informed Consent<br>Section 21 Quality Assurance and Clinical Monitoring
Study Assessments	
Clarification that the initial ophthalmological examination for subjects with lens implants will be performed prior to the first dose of study drug (during the Screening procedures or as part of the Baseline assessments), rather than being limited to Screening.	Synopsis<br>Section 10.1 General Description<br>Section 10.4.1 Screening Assessments<br>Section 10.4.1.2 Other Medical Screening Assessments<br>Section 10.4.2 Baseline and Post-randomization Assessments<br>Section 10.4.2.3 Safety Assessments<br>Section 15.4 Safety Assessments and Procedures
Baseline characteristics to be collected include smoking history and history of lens implantation.	Section 15.1 Demographic Data/Medical History
Additional instruction for investigators assessing AEs during the study includes asking one or more questions to evaluate suicidal ideation and behavior at each in-clinic visit, as recommended by the U.S. FDA. Questions are to be addressed to both subjects and their study partners.	Section 16.2 Eliciting Adverse Event Information
Clarification provided that lens discoloration reported during ophthalmological examination is to be reported as an SAE.	Section 16.7 Serious Adverse Event Reporting
Medical history and adverse events will be coded using MedDRA Version 20.1.	Section 17.6 Demographic and Baseline Characteristics
Direct bilirubin and alkaline phosphatase have been added to clinical chemistry laboratory assessments.	Section 28.2 Study Blood Assessments
Documentation	
Further details regarding archiving and management of the eTMF are provided. The requirements for document retention and archiving have been clarified to refer to a minimum of 25 years after the end of the clinical trial.	Section 7 Abbreviations<br>Section 22 Documentation
Clarification that all deviations identified at or by the site will be reported to the study monitor, and deviations from the SAP (if any) will be noted in the clinical study report.	Section 17 Statistical Analysis<br>Section 18 Regulatory and Ethics
Data Protection	
Clarification that data protection obligations are not defined in the protocol, but rather are to be described in vendor contracts and subject-facing documentation (*e.g.*, in appropriate transparency notices included in informed consent documentation) in compliance with relevant local laws.	Section 19 Confidentiality and Data Protection
Study Administration	
Reference to pipettes has been removed from calibration certification, as the pipettes provided in the laboratory kits are disposable.	Section 26 Study Administration

28.4.5 Protocol Version 4.1

The protocol for Study TRx-237-039 (Version 4.0 dated 24 August 2018) has been revised in this administrative amendment to include an additional Sponsor signatory for Medical Oversight, and to remove references to specific personnel for Medical Monitoring representation in North America and Europe. The most current Medical Monitoring contact information is now provided by cross-reference to the Site Contact List in the Investigator Site File.

A summary of the changes and affected sections is provided below.

**Summary of Changes**	**Affected Sections in Revised Protocol (Version 4.1)**
Protocol Signatories	
An additional Sponsor signatory has been added for TauRx Medical Oversight.	Section 3 Protocol Approval
Responsible Personnel	
Specific names and contact information have been removed for personnel responsible for medical monitoring (*i.e.*, the North America Medical Monitor and Europe Medical Monitor). The most current contact information is now provided by cross-reference to the Site Contact List in the Investigator Site File.	Section 4 Responsible Personnel

28.4.6 Protocol Version 5.0

The protocol for study TRx-237-039 (Version 4.1 dated 9 November 2018) has been revised primarily to include the following modifications:

- The study design now includes two phases, including the double-blind treatment period (now 12 months, extended from 9 months) with subjects randomized to LMTM 16 mg/day, LMTM 8 mg/day, or placebo, followed by an open-label, delayed-start treatment period with LMTM 16 mg/day for an additional 52 weeks; study visits and schedules of assessments have been modified accordingly

- The study population now includes subjects with early to mild-moderate AD (previously only early AD), and the number of randomized subjects has been increased to approximately 450 subjects (previously 375 subjects)

- The primary objectives for the double-blind treatment period now pertain to comparing LMTM 16 mg/day with placebo for the co-primary endpoints of ADAS-cog_{11} and ADCS-ADL_{23} (difference in temporal lobe ^{18}F-FDG-PET change in SUVR and the Composite Scale are no longer primary objectives), and for assessing safety and tolerability; secondary objectives have been modified to include comparisons of LMTM 16 mg/day with placebo in whole brain atrophy as measured by MRI, to restrict the ^{18}F-FDG-PET endpoints to subjects with CDR 0.5 at Screening, and to compare the LMTM dose of 8 mg/day for selected endpoints

Modifications have also been made to administrative information, criteria for subject enrollment and eligibility for the EAP, study drug supplies for the placebo group (now including a urinary discolorant to maintain blinding during the double-blind treatment period), and study assessments. A summary of the key changes and primary affected sections is provided below (as the Synopsis is affected by the majority of the changes, it is not listed).

Minor revisions have also been incorporated for clarity; these are not described below but are enumerated in a separate detailed summary of changes document.

Summary of Changes	Affected Sections in Revised Protocol (Version 5.0)
Administrative	
The GCP compliance statement has been modified to indicate that the study will be conducted in compliance with the principles contained in the Declaration of Helsinki, rather than include reference to a specific version.	Section 2 GCP Compliance Statement<br>Section 5 Investigator Signature Sheet<br>Section 18 Regulatory and Ethics
The statistician for the study has been changed to Bjoern Schelter, PhD (Data Analytics and Biostatistics Lead for TauRx).	Section 3 Protocol Approval
The TauRx Global Project Leads for this study are now Sotereos Gates and Alison Walker (previously Sean Neville).	Section 4 Responsible Personnel
A Coordinating Investigator, Serge Gauthier, C.M., C.Q., MD, FRCPC, is now identified.	Section 4 Responsible Personnel
The current 24-hour medical contact (DSSL) information is also provided by cross-reference to the Site Contact List in the Investigator Site File.	Section 4 Responsible Personnel

Summary of Changes	Affected Sections in Revised Protocol (Version 5.0)
For image hypersensitivities, Aberdeen Biomedical Imaging Centre has been removed as the data output will be provided by BioClinica, Inc.	Section 4 Responsible Personnel
Study Population	
The protocol now includes subjects with early to mild-moderate AD rather than only those with early AD. MMSE and CDR severity scores at Screening have been modified to reflect this change (*i.e.*, MMSE 16 to 27 [inclusive] and Global CDR 0.5 to 2 [if 0.5, including a score of >0 in one of the functional domains]). An additional stratification level for MMSE severity (*i.e.*, 16-19) has been added, with a target of approximately 2:3:1 for MMSE scores of 16-19, 20-25, and 26-27, respectively.	Cover Page<br>Section 10.1 General Description<br>Section 10.2 Study Population<br>Section 11.1 Inclusion Criteria<br>Section 11.3 Re-screening<br>Section 12.3 Randomization<br>Section 17 Statistical Analysis<br>Section 17.2 Number of Subjects and Sample Size Calculation
The study will be conducted in approximately 150 study sites in North America and Europe (previously planned to be at least 100 study sites in North America, Europe, and Rest of World). Approximately 2000 subjects (previously up to 1500 subjects) may be screened.	Section 10.2 Study Population<br>Section 12.3 Randomization<br>Section 17.4.9 Subgroup Analyses
Clarification provided that, at Screening, an interview and examination by the investigator are required to ensure subjects have a likely diagnosis of AD or MCI-AD prior to being offered consent forms. It is further specified that the accuracy of the diagnosis is to be confirmed independently by the diagnosing physician at the site.	Section 10.4.1 Screening Assessments<br>Section 11.1 Inclusion Criteria
Treatment Groups	
The primary treatment group comparison is now LMTM 16 mg/day *versus* placebo, with secondary comparison of subjects receiving 8 mg/day *versus* placebo. The rationale for revising primary LMTM dosing to 16 mg/day (using plasma concentration thresholds based upon a population PK model) and for adding the open-label, delayed-start treatment phase is described. Safety margins for nonclinical data were updated to reflect the 16 mg/day dose. Supportive data regarding reduction in treatment effects of LMTM by pretreatment with an AChEI or memantine are also described.	Section 8.1.2 Nonclinical Data<br>Section 8.2 Rationale<br>Section 9.1 Primary Objectives<br>Section 9.2.1 Double-blind Treatment Period<br>Section 10.1 General Description<br>Section 12.2 Study Regimens
The number of randomized subjects has been increased to approximately 450 subjects (previously 375 subjects), with a ratio of 4:1:4 (at the study level) to LMTM 16 mg/day (200 subjects), LMTM 8 mg/day (50 subjects), and placebo (200 subjects).	Section 10.1 General Description<br>Section 10.2 Study Population<br>Section 12.2 Study Regimens<br>Section 12.3 Randomization<br>Section 17.2 Number of Subjects and Sample Size Calculation
Study Design	
The study design has been modified to occur in two phases. The randomized, double-blind, placebo-controlled portion is now a 12-month (extended from 9-month) study, including five (rather than four) post-randomization visits (Visits 3 through 7). Following completion of the double-blind treatment period, subjects will continue open-label treatment with LMTM 16 mg/day for an additional 52 weeks, representing a modified delayed start of treatment; the open-label, delayed-start phase will include three treatment visits (Visit 8 [telephone contact], Visit 9, and Visit 10).<br><br>Prior treatment assignment will not be unblinded; however, upon completion of the initial 52-week, double-blind, placebo-controlled treatment period, the database will be	Cover Page<br>Section 8.2 Rationale<br>Section 9 Objectives<br>Section 10.1 General Description<br>Section 10.3 Duration<br>Section 10.4.2 Baseline and Post-randomization Assessments in Double-Blind Treatment Period<br>Section 10.4.3 Assessments in the Open-Label, Delayed-Start Phase<br>Section 12.2 Study Regimens<br>Section 12.5 Dispensing<br>Section 12.6 Compliance<br>Section 12.8 Breaking the Blind for the Double-Blind Treatment Period

Summary of Changes	Affected Sections in Revised Protocol (Version 5.0)
locked and unblinded for analysis. Individual subject treatment assignment will not be divulged to subjects or individuals involved in the conduct of the ongoing open-label, treatment phase (except in cases of medical situations where it is deemed essential in order to provide appropriate care). Access to the randomization list has also been clarified.	Section 13 Concomitant Medications and Subject Restrictions<br>Section 17.1.3 Secondary Endpoint for Open-Label, Delayed-Start Phase<br>Section 17.4.1 Hypothesis<br>Section 17.4.5 Open-Label, Delayed-Start Analysis
Total duration of participation for an individual subject is now up to 120 weeks (rather than 54 weeks), including a Screening period of up to 16 weeks, a double-blind treatment period of 52 weeks, and a further open-label, delayed-start phase of 52 weeks. Overall study duration is now anticipated to be at least 40 months (rather than 24 months), depending on recruitment.	Section 10.1 General Description<br>Section 10.3 Duration
Subject Enrollment and Withdrawal	
The protocol now states that, in the case of subjects with reduced decision-making capacity, legally acceptable representative(s), consistent with national law, may provide (and withdraw) written informed consent.	Section 7 Abbreviations<br>Section 10.4.1 Screening Assessments<br>Section 11.1 Inclusion Criteria<br>Section 11.4.2 Handling of Study Discontinuation / Withdrawal<br>Section 18.3 Informed Consent
Clarification that subjects are not to be treated with an AChEI and/or memantine within the 60 days prior to Baseline assessments, rather than only Baseline ^{18}F-FDG-PET as previously stated.	Section 10.1 General Description<br>Section 10.4.1 Screening Assessments<br>Section 11.1 Inclusion Criteria<br>Section 13.1 AChEI and/or Memantine
Re-screening of a subject is allowed to a newly specified maximum of two re-screening occasions. An additional acceptable instance for re-screening has been added for subjects with Hgb below the lower limit of laboratory normal; such subjects may be re-consented and re-screened after appropriate management at the discretion of the TauRx Medical lead. Any other criterion not included the list would require approval by TauRx.	Section 11.3 Re-Screening
Criteria for significant head injury no longer specifies a specific duration of associated loss of consciousness.	Section 11.2 Exclusion Criterion #3
Criteria for epilepsy now specifies that a single prior seizure >6 months prior to Screening is considered acceptable.	Section 11.2 Exclusion Criterion #4
Determination of whether or not a Screening Hgb value below age/sex appropriate lower limit of normal is exclusionary now includes discussion with the Medical Monitor, with final decision at the discretion of the Medical Oversight Lead.	Section 11.2 Exclusion Criterion #12
Subjects with moderate to severe sleep apnea are to be excluded if the apnea is currently, rather than previously, diagnosed; the definition of moderate to severe apnea is now included (*e.g.*, requiring oxygen supplementation).	Section 11.2 Exclusion Criterion #15
Clarified that active hepatitis or primary biliary cirrhosis; or active HTLV-III, LAV, mutants/derivatives of such, or conditions associated with AIDS or similar, are exclusionary.	Section 11.2 Exclusion Criterion #16
Further definitions have been added for exclusions regarding cancer diagnosis (*e.g.*, new diagnosis within past 2 years, or previous [>2 years] diagnosis requiring any intervention or treatment within past 2 years).	Section 11.2 Exclusion Criterion #17
Subjects who complete the study and receive treatment with LMTM up to and including the last open-label visit may be eligible for the EAP (and once entered into the EAP, are required to remain off AChEIs and/or memantine). Patients	Section 10.2 Study Population<br>Section 10.6 Definition of End of Study<br>Section 11.4.1 Handling of Subjects who Discontinue Study Drug

Summary of Changes	Affected Sections in Revised Protocol (Version 5.0)
who discontinue treatment permanently in this study will not be eligible for the EAP.	Section 12.2.2 Dose Interruption
Study Drug	
The study title now refers to the investigational study drug by its USAN. In addition to hydromethylthionine mesylate being the USAN, clarification is provided that it is also the INN.	Cover Page<br>Section 8 Background and Rationale for the Study<br>Section 8.1.1 Investigational Product<br>Section 12.1.1 Active Ingredient
The drug supplies for the placebo group will include tablets containing a urinary discolorant, MTC, 4 mg. To prevent inadvertent unblinding due to urinary discoloration, subjects randomized to placebo may receive a 4-mg tablet of MTC as one of the four tablets to be taken daily (the remainder being placebo tablets), in order to maintain the treatment blind.	Section 8.1.1 Investigational Product<br>Section 8.2 Rationale<br>Section 12.2 Study Regimens
Additional drug dispensing visits now include Visits 6 and 7 during double-blind treatment, as well as Visit 9 of the open-label, delayed-start phase, at which time the number of tablets dispensed will be recorded and the complete study drug kit will be returned. Subject compliance with study drug now will also occur at Visits 6 and 7 during the double-blind treatment period, at Visits 9 and 10 during the open-label, delayed-start phase, and during the telephone contact (Visit 8).	Section 10.4.2 Baseline and Post-randomization Assessments in Double-Blind Treatment Period<br>Section 10.4.3 Assessments in the Open-Label, Delayed-Start Phase<br>Section 12.2 Study Regimens<br>Section 12.5 Dispensing<br>Section 12.6 Compliance
Imaging Assessments	
^{18}F-FDG-PET imaging is now to be performed only in AD subjects with CDR 0.5; imaging is to occur at Baseline and at end of double-blind treatment, *i.e.*, Visit 7/Week 52 (or early termination). For purposes of establishing the Baseline assessment, ^{18}F-FDG-PET images are to be obtained prior to Visit 2 and at least 60 days after the last dose of AChEI and/or memantine.	Section 9.2.1 Double-blind Treatment Period<br>Section 10.1 General Description<br>Section 10.4.1 Screening Assessments<br>Section 10.4.2 Baseline and Post-randomization Assessments in Double-Blind Treatment Period<br>Section 10.4.2.1 Imaging Efficacy Assessments in Double-Blind Treatment Period<br>Section 13.4 Drugs Used to Manage Behavioral Disturbance<br>Section 13.5 Other Medications<br>Section 15.3.1 General Considerations<br>Section 15.3.3.1 ^{18}F-FDG-PET<br>Section 17.1.2 Secondary Efficacy Endpoints for Double-Blind Treatment Period<br>Section 17.3 Analysis Populations<br>Section 17.4.6 Dose Response Analyses
Additional volumetric brain MRIs and cognitive / functional assessments (ADAS-cog$_{13}$ and ADCS-ADL$_{23}$) are to be conducted at Visit 7/Week 52, as well as after the additional 26 and 52 weeks of open-label treatment (Visit 9/Week 78 and Visit 10/Week 104), or upon early termination in the case of the ADAS-cog$_{13}$ and ADCS-ADL$_{23}$. As pertains to early termination and MRI scans, no additional scan is required if the subject's last scan was performed < 90 days prior to the early termination date for either the double-blind treatment period or open-label treatment period.	Section 10.1 General Description<br>Section 10.4.2 Baseline and Post-randomization Assessments in Double-Blind Treatment Period<br>Section 10.4.2.1 Imaging Efficacy Assessments in Double-Blind Treatment Period<br>Section 10.4.2.2 Clinical Efficacy Assessments in Double-Blind Treatment Period<br>Section 10.4.3 Assessments in the Open-Label, Delayed-Start Phase<br>Section 10.4.3.1 Imaging Efficacy Assessments in the Open-Label, Delayed-Start Phase<br>Section 15.3.3.2 MRI
The estimated total radiation exposure to subjects from the amyloid PET scan procedure (if not performed previously) is now provided.	Section 15.3.1 General Considerations
Clinical Efficacy Assessments	
At Screening, the CDR assessment is only to be performed if the subject meets the MMSE inclusion criteria, and if the	Section 10.1 General Description

Summary of Changes	Affected Sections in Revised Protocol (Version 5.0)
subject does not qualify with either of these scales, no further assessments are to be made and the subject will be considered a screen failure. For enrolled and randomized subjects, the MMSE and CDR sum of boxes (as exploratory clinical efficacy assessments) are to be administered at Visit 7 and at the final open-label, delayed-start visit (Visit 10), or upon early termination.	Section 10.4.1.1 Diagnostic and Cognitive Eligibility Assessments<br>Section 10.4.2 Baseline and Post-randomization Assessments in Double-Blind Treatment Period<br>Section 10.4.2.2 Clinical Efficacy Assessments in Double-Blind Treatment Period<br>Section 10.4.3 Assessments in the Open-Label, Delayed-Start Phase<br>Section 10.4.3.2 Clinical Efficacy Assessments in the Open-Label, Delayed-Start Phase<br>Section 15.2.2 Instruments<br>Section 17.5 Exploratory Analyses
Efficacy rater requirements, allocation, and the order of assessments by visit have been clarified. The CDR and MMSE are now further described in the protocol with additional references included.	Section 15.2 Assessment of Efficacy *(and all subsections)*<br>Section 27 References
Safety Assessments	
Safety assessments are to occur at each clinic visit during double-blind treatment, with additional assessments after 26 and 52 weeks of open-label treatment (Visit 9/Week 78 and Visit 10/Week 104), and as needed to follow up on an AE. A telephone contact has been added to record AEs and changes in concomitant medications after 4 weeks in the open-label, delayed-start phase (Visit 8/Week 56). All safety assessments are to be performed by an independent qualified assessor not involved in efficacy measures, with certain assessments now specified to be conducted and/or reviewed by a medical assessor (physician/MD/DO), including targeted physical and neurological examinations, clinical laboratory and ECG results, and AEs and concomitant medications.	Section 10.1 General Description<br>Section 10.4.1.2 Other Medical Screening Assessments<br>Section 10.4.2 Baseline and Post-randomization Assessments in Double-Blind Treatment Period<br>Section 10.4.2.3 Safety Assessments in Double-Blind Treatment Period<br>Section 10.4.3 Assessments in the Open-Label, Delayed-Start Phase<br>Section 10.4.3.3 Safety Assessments in the Open-Label, Delayed-Start Phase<br>Section 13 Concomitant Medications and Subject Restrictions<br>Section 15.4 Safety Assessments and Procedures
Additional ophthalmological examinations in subjects with lens implants are to occur at Visits 7 and 10 (or early termination) to assess for potential lens discoloration. It is now specified that the examinations are to be conducted by an ophthalmologist, optometrist, or other suitably qualified medical assessor (physician/MD/DO).	Section 10.1 General Description<br>Section 10.4.1 Screening Assessments<br>Section 10.4.1.2 Other Medical Screening Assessments<br>Section 10.4.2 Baseline and Post-randomization Assessments in Double-Blind Treatment Period<br>Section 10.4.2.3 Safety Assessments in Double-Blind Treatment Period<br>Section 10.4.3 Assessments in the Open-Label, Delayed-Start Phase<br>Section 10.4.3.3 Safety Assessments in the Open-Label, Delayed-Start Phase<br>Section 15.4 Safety Assessments and Procedures
Targeted physical examinations, to be performed by a medical assessor, are now specified to include, at a minimum, heart and lung auscultation and brief neurological assessment guided by any reported signs/symptoms/AEs (*e.g.*, evaluating for potential serotonin toxicity).	Section 10.4.2 Baseline and Post-randomization Assessments in Double-Blind Treatment Period<br>Section 10.4.3 Assessments in the Open-Label, Delayed-Start Phase<br>Section 15.4 Safety Assessments and Procedures
For women of childbearing potential, it is now specified that the return visit to the clinic for pregnancy testing is to occur within 3 months of last exposure to study drug in the event of delayed menstruation.	Section 10.1 General Description<br>Section 13.7 Contraceptive Measures<br>Section 16.10 Reporting of Pregnancy
New AEs and SAEs reported by a subject after screen failure will not be recorded for the study. Emergent AEs and SAEs up to the point of screen failure will be followed up to resolution at the discretion of the PI.	Section 16.1 Definition of AEs, Period of Observation, and Recording of AEs<br>Section 16.7 Serious Adverse Event Reporting

Summary of Changes	Affected Sections in Revised Protocol (Version 5.0)
Other Assessments	
In addition to study visits that are to occur in the morning for Visits 2 and 3 to allow for in-clinic dosing and PK blood sampling, the protocol now specifies that in-clinic dosing and PK sampling are also to occur at morning appointments for Visit 7 (after 52 weeks on study drug) and Visit 10 (after 104 weeks of study drug). Subjects are to be instructed not to take their morning dose at home.	Section 10.1 General Description<br>Section 10.4.2 Baseline and Post-randomization Assessments in Double-Blind Treatment Period<br>Section 10.4.2.4 Other Assessments in Double-Blind Treatment Period<br>Section 10.4.3 Assessments in the Open-Label, Delayed-Start Phase<br>Section 10.4.3.4 Other Assessments in Open-Label, Delayed-Start Phase<br>Section 12.2 Study Regimens<br>Section 15.5.1 MT Concentration
A urine sample will no longer be collected for color determination by the central laboratory.	Section 10.1 General Description<br>Section 10.4.2 Baseline and Post-randomization Assessments in Double-Blind Treatment Period<br>Section 10.4.2.4 Other Assessments in Double-Blind Treatment Period<br>Section 15.5.2 Urine Color *(deleted)*
Blood samples for determination of plasma and whole blood MT concentrations will now be 9-mL blood samples collected in 9-mL vacutainers (previously 8-mL).	Section 15.5.1.1 Procedure for Blood Sample Collection
Objectives and Endpoints / Statistical Analyses	
The primary objectives now pertain to the double-blind treatment period only, comparing LMTM 16 mg/day with placebo for the co-primary endpoints of ADAS-cog$_{11}$ and ADCS-ADL$_{23}$ (difference in temporal lobe ^{18}F-FDG-PET change in SUVR and the Composite Scale are no longer primary objectives), and for assessing safety and tolerability. Secondary objectives have been modified for the double-blind treatment phase to include comparisons of LMTM 16 mg/day with placebo in whole brain atrophy as measured by MRI, to restrict the ^{18}F-FDG-PET endpoints to subjects with CDR 0.5 at Screening, and to compare the LMTM dose of 8 mg/day for selected endpoints. Brain MRI remains as a secondary efficacy endpoint, with clarification that it will also be used to confirm a lack of treatment unblinding.	Section 9.1 Primary Objectives<br>Section 9.2 Secondary Objectives<br>Section 9.2.1 Double-blind Treatment Period<br>Section 9.2.2 Open-Label, Delayed-Start Phase<br>Section 15.3.1 General Considerations<br>Section 17 Statistical Analysis<br>Section 17.1.1 Primary Efficacy Endpoints for Double-Blind Treatment Period<br>Section 17.1.2 Secondary Efficacy Endpoints for Double-Blind Treatment Period<br>Section 17.4.1 Hypothesis
The Composite Scale will now be based on items selected from the ADAS-cog$_{13}$, rather than the ADAS-cog$_{11,}$ and on selected items from the ADCS-ADL$_{23,}$ with analysis at 9 months and 12 months during double-blind treatment to evaluate usefulness for future studies and is included in the exploratory analyses.	Section 15.2.2.1 ADAS-cog<br>Section 15.2.2.2 ADCS-ADL$_{23}$<br>Section 15.2.2.3 Composite Scale
Two open-label, delayed-start phase objectives have been added: determination if there is a difference in disease progression on the co-primary clinical endpoints and MRI imaging endpoint for subjects who started treatment in the double-blind phase and those who started in the open-label, delayed-start phase (*i.e.*, “early” and “late” LMTM starters, respectively), and assessment of safety and tolerability of LMTM given for up to 104 weeks. Additional statistical analysis for the open-label phase has been defined.	Section 9.2.2 Open-Label, Delayed-Start Phase<br>Section 17 Statistical Analysis<br>Section 17.1.3 Secondary Endpoint for Open-Label, Delayed-Start Phase
Sample size estimations and study power to determine treatment effect have been revised based upon changes to primary treatment group, study population, co-primary clinical endpoints, and length of the double-blind treatment period of the study. Sample size estimations have also been	Section 17.2 Number of Subjects and Sample Size Calculation

Summary of Changes	Affected Sections in Revised Protocol (Version 5.0)
added for the open-label, delayed-start treatment period of the study.	
Clarification is provided that the primary efficacy analyses for FDA will be based on the E-MITT, MI-MITT, or I-MITT population (depending on endpoint), and for EMA on the ITT population. The co-primary efficacy endpoints and MRI will be analyzed using a linear mixed model for repeated measures with unstructured covariance matrix, and ^{18}F-FDG-PET analyzed using an ANCOVA, with covariates for this model adjusted accordingly.	Section 17 Statistical Analysis<br>Section 17.4 Clinical Efficacy and Imaging Analysis<br>Section 17.4.2 FDA Analysis
Evaluation of the influence of ApoE genotype (in subjects who provide legally acceptable consent) on the primary and selected secondary endpoints is now an exploratory endpoint (previously a secondary endpoint).	Section 9.2 Secondary Objectives<br>Section 17.1.2 Secondary Efficacy Endpoints for Double-Blind Treatment Period<br>Section 17.5 Exploratory Analyses
No interim futility or efficacy analysis is planned in which treatment groups will be compared. Recruitment and discontinuations will, however, be continuously monitored in a blinded fashion and projections on future dropouts calculated. In addition, a blinded interim analysis to re-estimate the assumed SD for the change from Baseline to Week 52 in the primary endpoints may be carried out at some point during the study's recruitment period.	Section 17.9 Interim Analysis

28.4.7 Protocol Version 5.1

The protocol for Study TRx-237-039 (Version 5.0 dated 9 July 2019) has been revised primarily to include modifications to the exclusion criterion regarding hematological abnormalities (reverting to the original criterion), clarifications to background information for the study drug and the efficacy objectives/statistical analyses, as well as updates to responsible personnel. A summary of the key changes and affected sections is provided below.

Additional revisions are editorial and are intended to correct typographical errors or add further clarification.

Summary of Changes	Affected Sections in Revised Protocol (Version 5.1)
Responsible Personnel	
Charles River Laboratories Edinburgh Ltd has been added to responsible personnel for analysis of MT concentrations (cross-validation purposes only).	Section 4 Responsible Personnel
Subject Enrollment and Withdrawal	
The provision for discussion of potentially exclusionary low screening Hgb values with the Medical Monitor has been removed; Hgb values below age/sex appropriate lower limit of the central laboratory normal range are once again exclusionary regardless of etiology.	Synopsis<br>Section 11.2 Exclusion Criterion #12<br>Section 11.3 Re-screening
Study Drug	
Clarification has been added that the increased incidences of pancreatic islet cell adenoma and adenoma or carcinoma (combined) in male rats, reported in a 2-year carcinogenicity study performed by the National Toxicology Program, were attributed to MTC by FDA (as interpreted by the FDA Executive Carcinogenicity Assessment Committee).	Section 8.1.2 Nonclinical Data
Clarification has been added that the magnitude of concentration-dependent treatment effects was reduced when LMTM was given to subjects who were concurrently using (rather than prior use of) AChEIs and/or memantine.	Section 8.1.3.3 Efficacy
Objectives and Endpoints / Statistical Analyses	
To correct a typographical error in the synopsis regarding the first secondary endpoint (the comparison of the LMTM dose of 16 mg/day with the placebo group in annualized rate of whole brain atrophy, rather than temporal and parietal lobe atrophy).	Synopsis
To clarify the exploratory analysis for comparison of decline in temporal lobe ^{18}F-FDG-PET SUVR with the estimated decline for subjects with mild AD (CDR 0.5); reference to the MMSE range has been removed. The analyses will be further described and clarified in the SAP.	Section 17.5 Exploratory Analyses

28.4.8 Protocol Version 6.0

The protocol for Study TRx-237-039 (Version 5.1 dated 05 February 2020) has been revised to incorporate modifications to the study conduct and monitoring, guidance for continued data collection and analysis, and ongoing risk assessment due to the COVID-19 Public Health Emergency. In addition, the total number of subjects to be randomized has increased, and updates have been incorporated for contact information for responsible personnel, as well as modifications and/or clarifications to investigator responsibilities, study assessments, and statistical analyses. Reference to a separate plan has been included for additional analyses to be undertaken of the blinded ^{18}F-FDG-PET and MRI data for scientific research and quality control purposes. A summary of the key changes and affected sections is provided below.

Additional revisions are editorial and are intended to correct typographical errors and editorial inconsistencies as well as to add clarification.

Summary of Changes	Affected Sections in Revised Protocol (Version 6.0)
Changes due to COVID-19 Public Health Emergency	
The post-Baseline visits for safety, PK, and genotyping blood sample collection as well as efficacy assessments may be completed in-clinic at the site, in the home, or at other safe, suitable alternative location if deemed necessary to protect subjects due to COVID-19. Acceptable procedures for safety and efficacy assessments, blood sample collection, shipment, and analysis are described. Study visit windows may be further extended at the discretion of the Sponsor when justification is provided.	Synopsis<br>Section 10.1 General Description<br>Section 10.2.1 Changes to Study Conduct<br>Section 10.5.3 Assessments in the Open-Label, Delayed-Start Phase<br>Section 13.7 Contraceptive Measures<br>Section 15.4 Safety Assessments and Procedures<br>Section 15.5.1 MT Concentration<br>Section 16.7 Serious Adverse Event Reporting
Alternative arrangements for dispensing study drug supplies may be permitted if deemed necessary to protect subjects due to COVID-19. Requirements for obtaining subject consent consistent with local and national requirements, maintaining the specified study drug storage conditions, and handling, accountability, and compliance monitoring will be addressed and documented.	Synopsis<br>Section 10.1 General Description<br>Section 10.2.1 Changes to Study Conduct<br>Section 12.2 Study Regimens<br>Section 12.5 Dispensing
If a subject is undergoing screening assessments and cannot have assessments performed due to COVID-19, the subject should be put on screening pause until such times as COVID-19 impacts have ceased.	Synopsis<br>Section 10.2.1 Changes to Study Conduct<br>Section 10.5.1.1 Diagnostic and Cognitive Eligibility Assessments<br>Section 11.3 Re-screening
The Sponsor may approve subject randomization without a brain ^{18}F-FDG-PET scan if the scan cannot be performed due to COVID-19.	Synopsis<br>Section 10.1 General Description<br>Section 10.2.1 Changes to Study Conduct<br>Section 10.5.1 Screening Assessments<br>Section 10.5.2 Baseline and Post-randomization Assessments in Double-Blind Treatment Period
All MRI scans are to be performed at approved MRI centers using approved scanners; no alternative scanners will be accepted; there is to be no change in scanner for a given subject over the course of the study. Guidance will be provided for scheduled MRI visits at approved imaging centers that may be closed due to COVID-19, including acceptable windows outside of the scheduled visit window during which MRI scans can still be performed and would be accepted for analysis.	Synopsis<br>Section 10.1 General Description<br>Section 10.2.1 Changes to Study Conduct<br>Section 15.3.1 General Considerations<br>Section 15.3.3.2 MRI
All ^{18}F-FDG-PET scans are to be performed at approved PET centers using approved scanners; there is to be no	Synopsis<br>Section 10.1 General Description<br>Section 10.2.1 Changes to Study Conduct

Summary of Changes	Affected Sections in Revised Protocol (Version 6.0)
change in scanner for a given subject over the course of the study.	Section 15.3.1 General Considerations
The ADAS-cog$_{13}$ may also be performed by videoconference and the MMSE, ADCS-ADL$_{23}$, and CDR may be performed by speakerphone. The remote efficacy scale assessments are now described in the protocol.	Synopsis<br>Section 10.1 General Description<br>Section 10.2.1 Changes to Study Conduct<br>Section 15.2.2 Instruments
Clarification is now provided that multiple study partners participating only as caregivers will be permitted for a given subject, either simultaneously or as replacements for previous study partners, with no specified maximum. A maximum total of two study partners participating as informants (*i.e.*, one replacement) would normally be permitted for a given subject; however, up to a total of four study partners (*i.e.*, three replacements) are permitted if circumstances change due to COVID-19. Information obtained from study partners participating as informants will be appropriately identified to distinguish each informant in the event that a given subject has more than one study partner providing data for analysis.	Synopsis<br>Section 10.1 General Description<br>Section 10.2.1 Changes to Study Conduct<br>Section 10.5.1 Screening Assessments<br>Section 10.5.1.1 Diagnostic and Cognitive Eligibility Assessments<br>Section 11.4.2 Handling of Study Discontinuation / Withdrawal<br>Section 11.4.3 Replacements<br>Section 15.2.1 Raters
For PK blood sample collection at Visit 2, Visit 3, Visit 7, and Visit 10, the third sample to be collected approximately 4 hours after the dose is not required if the visit occurs in the home.	Synopsis<br>Section 10.2.1 Changes to Study Conduct<br>Section 10.5.2 Baseline and Post-randomization Assessments in Double-Blind Treatment Period<br>Section 10.5.2.4 Other Assessments in Double-Blind Treatment Period<br>Section 10.5.3 Assessments in the Open-Label, Delayed-Start Phase<br>Section 10.5.3.4 Other Assessments in Open-Label, Delayed-Start Phase<br>Section 15.5.1 MT Concentration
Sections have been added describing the Sponsor's ongoing risk assessment regarding the impact of COVID-19 on this study, including guidance for continued data collection and potential changes in site and remote monitoring procedures, as well as changes to statistical analyses.	Synopsis<br>Section 10.2 Changes Implemented Due to COVID-19<br>Section 10.2.1 Changes to Study Conduct<br>Section 10.2.2 Changes to Study Monitoring<br>Section 10.2.3 Changes to Statistical Analyses<br>Section 17.4.11 Sensitivity Analyses<br>Section 17.9 Interim Analysis<br>Section 18 Regulatory and Ethics<br>Section 21.2 COVID-19 Risk Assessment *(new)*
Responsible Personnel	
A DSSL 24-hour medical contact telephone number has been added for France.	Section 4 Responsible Personnel
Investigator responsibilities include supervising any individual or party to whom the investigator delegates trial-related duties and functions conducted at the trial site.	Section 18.4 Investigator Responsibilities
Study Population	
The number of randomized subjects has been increased: a sufficient number of subjects will be recruited such that approximately 500 subjects are enrolled; beginning with Version 5.0, approximately 450 subjects are to be enrolled and randomized in a 4:1:4 ratio (at the study level) to the LMTM 16-mg/day, LMTM 8-mg/day, and placebo groups. The inclusion criterion for an MMSE score of 16-27 (inclusive) at Screening will be subject to stratification requirements.	Synopsis<br>Section 10.1 General Description<br>Section 10.3 Study Population<br>Section 11.1 Inclusion Criteria<br>Section 12.2 Study Regimens<br>Section 12.3 Randomization<br>Section 17.2 Number of Subjects and Sample Size Calculation

Summary of Changes	Affected Sections in Revised Protocol (Version 6.0)
Imaging	
If subjects have an acceptable MRI scan or PET scan (the latter only in subjects who have a screening CDR of 0.5) already completed during the original Screening window, the scan does not require repetition.	Section 10.2.1 Changes to Study Conduct<br>Section 10.5.1.1 Diagnostic and Cognitive Eligibility Assessments<br>Section 11.3 Re-screening<br>Section 15.3.3.1 ^{18}F-FDG-PET
Reference is included to a separate plan and data transfer agreement that will capture standard procedures for additional exploratory analyses to be undertaken to characterize the blinded ^{18}F-FDG-PET and MRI data for scientific research and quality control purposes, which are separate from the study objectives.	Section 20.2 Imaging Data *(new)*
Added consistent wording regarding baseline ^{18}F-FDG-PET scan in subjects who have a screening CDR of 0.5.	Synopsis<br>Section 10.1 General Description<br>Section 10.5.2.1 Imaging Efficacy Assessments in Double-Blind Treatment Period<br>Section 11.3 Re-screening<br>Section 13.1 AChEI and/or Memantine<br>Section 15.3.3.1 ^{18}F-FDG-PET
Safety	
Permanent discontinuation due to decreased creatinine clearance is only required in subjects if renal concerns arise.	Section 11.4.1 Handling of Subjects Who Discontinue Study Drug<br>Section 16.11.2 Other Safety Reasons Requiring Discontinuation of Study Drug
Any abnormal laboratory test result from Screening assessments (prior to treatment) is to be added to the subject's medical history, unless deemed clinically significant by the medical assessor (MD/DO) in which case it will be recorded as an AE.	Section 15.4 Safety Assessments and Procedures<br>Section 16.1 Definition of AEs, Period of Observation, and Recording of AEs
Other Assessments	
Guidance is now provided regarding collection of blood samples at early termination visits for determination of MT concentrations.	Synopsis<br>Section 10.5.2 Baseline and Post-randomization Assessments in Double-Blind Treatment Period<br>Section 10.5.2.4 Other Assessments in Double-Blind Treatment Period<br>Section 10.5.3 Assessments in the Open-Label, Delayed-Start Phase<br>Section 10.5.3.4 Other Assessments in Open-Label, Delayed-Start Phase<br>Section 15.5.1 MT Concentration
Statistical Analyses	
The secondary efficacy endpoints to compare the difference in temporal lobe ^{18}F-FDG-PET change in SUVR normalized to pons in subjects with CDR 0.5 at Screening (LMTM 16 mg/day *versus* placebo, and LMTM 8 mg/day *versus* placebo) will now only be analyzed if a predefined threshold is reached for a sufficient number of subjects providing data as specified in the SAP.	Synopsis<br>Section 9.2.1 Double-Blind Treatment Period<br>Section 17.1.2 Secondary Efficacy Endpoints for Double-Blind Treatment Period

Summary of Changes	Affected Sections in Revised Protocol (Version 6.0)
Upon completion of the initial 52-week, double-blind, placebo-controlled treatment period, the database will be locked and unblinded for analysis, and an interim report will be prepared describing the complete efficacy and safety analyses of the double-blind phase; individual subject prior treatment assignment will not be divulged to subjects or individuals involved in the operational conduct of the ongoing open-label, treatment phase.	Section 10.1 General Description<br>Section 10.7 Definition of End of Study<br>Section 12.8 Breaking the Blind for Double-Blind Treatment Period<br>Section 17.9 Interim Analysis
Due to the introduction of MTC spiking in the placebo arm with Protocol Version 5.0, the respective MITT populations will be restricted to subjects randomized to Protocol Versions 5.0 and above for the primary and key secondary analyses as per the SAP. The full MITT populations will be analyzed for these as a sensitivity analysis. Details will be provided in the SAP.	Section 17.3 Analysis Populations<br>Section 17.4 Clinical Efficacy and Imaging Analysis
It is now indicated that the SAP will specify estimands to provide a more comprehensive assessment of the performance and efficacy of the drug accounting for various factors (importantly, drop-outs).	Section 17.4.4 Estimands
The last observation carried forward (LOCF) imputation has been removed from the statistical analyses.	Section 17.4.11.6 Last Observation Carried Forward (LOCF) Imputation *(deleted)*

28.4.9 Protocol Version 7.0

The protocol for Study TRx-237-039 (Version 6.0 dated 09 October 2020) has been revised to incorporate changes to responsible personnel; updates to the background information to reflect the most current version of the Investigator's Brochure; clarification of assessments and drug dispensing for Visit 7 of double-blind treatment (also Baseline/Day 1 of open-label phase); addition of lens discoloration as an AESI and further instruction regarding timing of slit lamp examinations; clarification that MT concentration sampling will not continue for the off-treatment on-study (TOTOS) group; and further description of composite scores and endpoints. Additional statistical analysis updates include sensitivity and subgroup analyses, and further detail provided for primary endpoints, all in response to potential impacts of COVID-19; the primary efficacy hypotheses have been clarified; and the primary estimand and five components of interest are now described.

A summary of the key changes and affected sections is provided below.

Additional revisions are editorial and are intended to correct typographical errors and editorial inconsistencies as well as to add clarification.

Summary of Changes	Affected Sections in Revised Protocol (Version 7.0)
Signatories / Responsible Personnel	
Modified address for Sponsor Medical Oversight signatory.	Section 3 Protocol Approval
The Global Project Manager has been changed to Marty Perry, with corresponding telephone numbers and email address added.	Section 4 Responsible Personnel
The 24-hour Medical Contact has been changed from DSSL to Emergency Scientific and Medical Services Global (ESMS Global).	Section 4 Responsible Personnel
The Central Laboratory has been changed from Covance, Inc. to Labcorp Central Laboratory Services (e.g., Labcorp Central Laboratory Services LP in the Americas and Labcorp Central Laboratory Services S.à r.l in Europe).	Section 4 Responsible Personnel<br>Section 10.2.1 Changes to Study Conduct<br>Section 10.5.1 Screening Assessments<br>Section 15.5.2 Genotyping
Certara USA, Inc. is now responsible for Statistical Analysis of Pharmacokinetic Data rather than the Institute for Clinical Pharmacodynamics, with corresponding addresses and telephone number added for Certara's New Jersey and Amsterdam locations.	Section 4 Responsible Personnel
The data management and statistics responsible personnel have now been divided, with Synteract, Inc. continuing to be responsible for data management and Cytel Inc. now responsible for statistics (corresponding address and telephone number added for Cytel Inc.).	Section 4 Responsible Personnel<br>Section 12.8 Breaking the Blind for Double-Blind Treatment Period
Background	
The background information has been updated to be reflective of the most current version of the Investigator's Brochure (Version 23.0), including updates to nonclinical and clinical data as well as the study drug terminology. The discussion of drugs currently available to treat AD has been updated to include the recent FDA accelerated approval of Aduhelm (aducanumab).	Synopsis<br>Section 8 Background and Rationale for the Study<br>Section 8.1.1 Investigational Product<br>Section 8.1.2 Nonclinical Data<br>Section 8.1.3.1 Pharmacokinetics<br>Section 8.1.3.3 Efficacy<br>Section 8.1.3.4 Safety<br>Section 8.2 Rationale<br>Section 12.1.1 Active Ingredient<br>Section 13.2 Drugs with Serotonergic Potential

Summary of Changes	Affected Sections in Revised Protocol (Version 7.0)
	Section 13.3 CYP and P-gp Substrates<br>Section 13.6 Dietary Tyramine<br>Section 13.8 Folate and Vitamin B_{12}<br>Section 15.5.1 MT Concentration<br>Section 17.8 Other Data<br>Section 27 References
Study Visits and Drug Dispensing	
Clarification provided that Visit 7 of the double-blind treatment period is also Baseline/Day 1 of the open-label phase. The in-clinic dose of study drug at Visit 7 is to be taken from a newly dispensed open-label study drug kit, after pre-dose assessments have been completed. The pre-dose and post-dose assessments to be performed at Visit 7 have also been clarified.	Synopsis<br>Section 10.5.2 Baseline and Post-randomization Assessments in Double-Blind Treatment Period<br>Section 10.5.3 Assessments in the Open-Label, Delayed-Start Phase<br>Section 12.2 Study Regimens<br>Section 12.5 Dispensing<br>Section 15.2.1 Raters<br>Section 17 Statistical Analyses<br>Section 17.4.6 Open-Label, Delayed-Start Analysis
Efficacy	
Composite Scores (and additional Composite Endpoints [if any]) derived from the ADAS-cog_{13} and the ADCS-ADL_{23} will be defined in the SAP prior to sign off and database lock/unblinding, with at least one designed to be sensitive to early stages of the disease as well as one that is less/not impacted by COVID-19.	Synopsis<br>Section 15.2.2 Instruments<br>Section 15.2.2.3 Composite Scale(s)<br>Section 17.5 Exploratory Analyses
Safety	
Clarification that slit lamp ophthalmological examinations in subjects with history of lens implants are to be performed prior to the first dose of study drug (Screening/Baseline), at Visit 7, and at Visit 10/early termination to check for lens discoloration. In addition, this examination is now to be performed if subjects have cataract surgery/lens implantation at any point during study participation (as soon as possible after the surgery), as well as in response to visual complaints if suggestive of lens discoloration.	Synopsis<br>Section 15.4 Safety Assessments and Procedures<br>Section 16.11.2 Lens Discoloration
A new section added for lens discoloration, which is now a pre-specified AESI (in addition to hemolytic anemia). If a lens discoloration is identified by slit lamp examination, it is to be recorded as an AESI in the AE eCRF; however, no specific action is required for study drug. Lens discoloration has been removed from the list of examples of SAEs that are not life-threatening, as it should be reported as an AESI unless SAE reporting criteria are later met.	Section 16.7 Serious Adverse Event Reporting<br>Section 16.11 Guidance for the Handling of Adverse Events of Special Interest and Selected Test Abnormalities<br>Section 16.11.2 Lens Discoloration
Clarification that adverse events of malignancies other than non-melanoma skin cancers are to be reported to Syneos Health (regardless of causality or whether they meet the criteria for serious).	Section 16.9 Malignancies<br>Section 17.7 Safety Analysis
Other Assessments	
For MT concentration sampling, if a subject has discontinued study drug but continues to attend study visits off-treatment (the TOTOS group), blood samples will not continue to be collected for measurement of MT concentrations.	Synopsis<br>Section 10.5.2 Baseline and Post-randomization Assessments in Double-Blind Treatment Period<br>Section 10.5.3 Assessments in the Open-Label, Delayed-Start Phase<br>Section 11.4.1 Handling of Subjects Who Discontinue Study Drug

Summary of Changes	Affected Sections in Revised Protocol (Version 7.0)
	Section 15.5.1 MT Concentration
Statistical Analyses	
Further detail provided that if >5% of all assessments (over all subjects and visits) of a given primary endpoint are impacted by COVID-19 (based on the eCRF COVID-19 Impact Assessment), the impacted assessment will be excluded and upscaled when the respective primary endpoint is analyzed.	Section 10.2.3 Changes to Statistical Analyses
Other sensitivity analyses were added and include: subgroup analysis of endpoint ascertainment (in-clinic, remote) with subjects assigned to the subgroup with most visits done, excluding (in-clinic) Baseline and Week 52 visits; analysis of ADAS-cog$_{11}$ to include only subset of items (for all visits) which are administered remotely by telephone or video call; analysis of ADCS-ADL$_{23}$ to exclude items affected by COVID-19 restrictions; and analysis of composite scale designed as COVID-19 impact free joint score, to include items selected from ADAS-cog$_{11}$ and ADCS-ADL$_{23}$ that are not expected to be impacted by COVID-19.	Section 10.2.3 Changes to Statistical Analyses
Clarification provided that the two co-primary endpoints are Baseline adjusted decline in ADAS-cog$_{11}$ and Baseline adjusted decline in ADCS-ADL$_{23}$ from Baseline at Week 52.	Section 17.4.1 Hypothesis
The primary efficacy hypotheses have been clarified, with the global null versus alternative as a Union-Intersection Test requiring both co-primary endpoints to meet statistical significance at the 5% two-sided level of significance for the global null hypothesis to be rejected.	Section 17.4.1 Hypothesis
For the ITT (EMA) analysis, no data will be imputed, unless specified in selected sensitivity/exploratory endpoints.	Section 17.4.3 ITT (EMA) Analysis
The Estimands section has been expanded, with the primary estimand now defined and constructed in line with ICH E9 (R1) addendum. The five components for the estimand of interest are as follows: treatment, population, patient-level outcomes/variables, population-level summary, and intercurrent events (ICE).	Section 17.4.4 Estimands

**Sponsor:**	TauRx Therapeutics Ltd.		
**Protocol:**	TRx-237-039		
**Document Version No.:**	3.0	**Document Date:**	07-JUN-2023

STATISTICAL ANALYSIS PLAN

(Double-Blind and Open-Label Extension Phase)

Protocol TRx-237-039

Randomized, Double-Blind, Placebo-Controlled, Three-Arm, 12-Month, Safety and Efficacy Study of Hydromethylthionine Mesylate (LMTM) Monotherapy in Subjects with Alzheimer's Disease Followed by a 12-Month Open-Label Treatment

**Protocol Number: (Version Date)**	TRx-237-039 (7.0)<br>28-JUL-2021 (UK: 19-AUG-2021)
**Name of Test Drug:**	Hydromethylthionine Mesylate (HMTM) previously referred to as LMTM, TRx0237
**Phase:**	3
**Methodology:**	Randomized, Double-Blind, Placebo-Controlled, Three-Arm, 12-Month, Safety and Efficacy Study of Hydromethylthionine Mesylate (LMTM) Monotherapy in Subjects with Alzheimer's Disease Followed by a 12-Month Open-Label Treatment
**Sponsor:**	TauRx Therapeutics Ltd.<br>395 King Street, Aberdeen AB24 5RP<br>Scotland, UK<br>Tel: [redacted]<br><br>TauRx Therapeutics Ltd.<br>3 Shenton Way, #21-04 Shenton House<br>Singapore 068805, Republic of Singapore
**Sponsor Representative:**	Professor Bjoern Schelter<br>Chief Analytics Officer

TauRx / Cytel Confidential: This is a controlled document and shall not be changed, saved, copied or reproduced by any unauthorized person.

**Sponsor:**	TauRx Therapeutics Ltd.		
**Protocol:**	TRx-237-039		
**Document Version No.:**	3.0	**Document Date:**	07-JUN-2023

Sponsor Representative: Dr Diane Downie
Operations Director

Document Date: 07 June 2023

Document Version: 3.0

TauRx / Cytel Confidential: This is a controlled document and shall not be changed, saved, copied or reproduced by any unauthorized person.

SIGNATURE PAGE

Protocol Title: Randomized, Double-Blind, Placebo-Controlled, Three-Arm, 12-Month, Safety and Efficacy Study of Hydromethylthionine Mesylate (LMTM) Monotherapy in Subjects with Alzheimer's Disease Followed by a 12-Month Open-Label Treatment

Sponsor: TauRx Therapeutics Ltd.
395 King Street, Aberdeen AB24 5RP
Scotland, UK
Tel: [redacted]

TauRx Therapeutics Ltd.
3 Shenton Way, #21/04 Shenton House
Singapore 068805, Republic of Singapore

Protocol Number: TRx-237-039

SAP Document Date/Version: 07 June 2023/v3.0

Cytel, Inc. Author:
Sebastian Pfeiffer

Senior Biostatistician

Signature:_______________________________________

Date:____

Sponsor Approval

By signing this document, I acknowledge that I have read the document and approve of the planned statistical analyses described herein. I agree that the planned statistical analyses are appropriate for this study, are in accordance with the study objectives, and are consistent with the statistical methodology described in the protocol, and all applicable regulatory guidance's and guidelines.

I have discussed any questions I have regarding the contents of this document with the biostatistical author.

I also understand that any subsequent changes to the planned statistical analyses, as described herein, may have a regulatory impact and/or result in timeline adjustments. All changes to the planned analyses will be described in the Clinical Study Report (CSR).

TauRx / Cytel Confidential: This is a controlled document and shall not be changed, saved, copied or reproduced by any unauthorized person.

**Sponsor:**	TauRx Therapeutics Ltd.		
**Protocol:**	TRx-237-039		
**Document Version No.:**	3.0	**Document Date:**	07-JUN-2023

Sponsor Signatory:

[redacted]

Signature:____

Date:____

Sponsor Signatory:

[redacted]

Signature:____

Date:____

TauRx / Cytel Confidential: This is a controlled document and shall not be changed, saved, copied or reproduced by any unauthorized person.

MODIFICATION HISTORY

Unique Identifier for SAP Version	Date of SAP Version	Author	Changes from the Previous Version
			Initial issuance of document
V 1.0	27-AUG-2021	Sebastian Pfeiffer	First signed version
V 2.0	01-MAY-2022	Sebastian Pfeiffer	Implementing protocol v7.0 and v7.0 UK. Incorporating information from SAP review meeting and BDRM. Specifying SPM analysis.
V 3.0	07-JUN-2023	Sebastian Pfeiffer	Update of section Statistical Analyses of Open-Label Treatment Phase (including TLF shells). Adding Biomarker Analysis section (including TLF shells). Differentiation between subjects receiving only true placebo and subjects receiving at least one dose of MTC. Additional (post hoc) analyses.

TauRx / Cytel Confidential: This is a controlled document and shall not be changed, saved, copied or reproduced by any unauthorized person.

**Sponsor:**	TauRx Therapeutics Ltd.		
**Protocol:**	TRx-237-039		
**Document Version No.:**	3.0	**Document Date:**	07-JUN-2023

TABLE OF CONTENTS

MODIFICATION HISTORY 5
1. INTRODUCTION AND OBJECTIVES OF ANALYSIS 16
1.1. INTRODUCTION 16
1.2. OBJECTIVES OF STATISTICAL ANALYSIS 17
1.3. STUDY PROTOCOL VERSIONS 17
1.4. UK VERSIONS OF STUDY PROTOCOL 18
1.5. INTERIM ANALYSIS 19
1.6. DATABASE LOCKS 19
2. STUDY DESIGN 21
2.1. SYNOPSIS OF STUDY DESIGN 21
2.2. SCREENING PERIOD 22
2.3. DOUBLE-BLIND TREATMENT PHASE (WEEK 0 UP TO WEEK 52) 22
2.4. OPEN-LABEL TREATMENT PHASE (WEEK 52 UP TO WEEK 104) 22
2.5. CHANGES OF STUDY CONDUCT DUE TO COVID-19 23
2.6. RANDOMIZATION METHODOLOGY 23
2.7. STOPPING RULES AND UNBLINDING 24
2.8. STUDY PROCEDURES 25
2.9. EFFICACY, PHARMACOKINETIC, SAFETY, AND OTHER VARIABLES 30
2.9.1. EFFICACY VARIABLES 30
2.9.2. PHARMACOKINETIC VARIABLES 32
2.9.3. SAFETY VARIABLES 33
2.9.4. OTHER VARIABLES 34
3. SUBJECT POPULATIONS 35
3.1. POPULATION DEFINITIONS 35
3.1.1. DOUBLE-BLIND POPULATIONS 35

TauRx / Cytel Confidential: This is a controlled document and shall not be changed, saved, copied or reproduced by any unauthorized person.

3.1.2. OPEN-LABEL POPULATIONS 36

3.1.3. HMTM TREATMENT PHASE POPULATION 36

3.2. PROTOCOL DEVIATIONS 36

4. STATISTICAL METHODS 38

4.1. GENERAL METHODS 38

4.2. DATA CONVENTIONS 38

4.3. COMPUTING ENVIRONMENT 40

4.4. WITHDRAWALS, DROPOUTS, LOSS TO FOLLOW-UP 40

4.5. MISSING, UNUSED, AND SPURIOUS DATA 41

4.5.1. UPSCALING 41

4.5.2. MULTIPLE IMPUTATION 42

4.6. CONTROL GROUP LABELING 45

4.7. SUBJECT DATA LISTINGS 45

4.8. SUMMARY STATISTICS 46

5. STATISTICAL ANALYSIS OF DOUBLE-BLIND TREATMENT PHASE 48

5.1. PRIMARY EFFICACY ANALYSIS 48

5.2. MULTIPLE COMPARISONS/MULTIPLICITY 48

5.3. SAMPLE SIZE JUSTIFICATION 49

5.4. PROTOCOL DEVIATIONS 49

5.5. VISIT WINDOWS 49

5.6. SUBJECT DISPOSITION 50

5.7. DEMOGRAPHIC AND BASELINE CHARACTERISTICS 52

5.8. PRIOR AND CONCOMITANT MEDICATIONS 53

5.9. EFFICACY EVALUATION 54

5.9.1. ESTIMANDS 54

5.9.2. PRIMARY EFFICACY ENDPOINT ANALYSIS 61

5.9.3. SECONDARY EFFICACY ENDPOINT ANALYSIS 66

TauRx / Cytel Confidential: This is a controlled document and shall not be changed, saved, copied or reproduced by any unauthorized person.

5.9.4. RESPONDER ANALYSIS (SENSITIVITY) 70

5.9.5. TIME-TO-EVENT/DECLINE ANALYSIS (SENSITIVITY) 71

5.10. EXPLORATORY ANALYSES 72

5.10.1. ADAS-COG_{13} 73

5.10.2. COMPOSITE SCALE 73

5.10.3. MMSE 73

5.10.4. CDR-SOB 73

5.10.5. MRI PARAMETERS 73

5.10.6. ^{18}F-FDG-PET PARAMETERS 73

5.10.7. MRI HYPOINTENSITIES AND HYPERINTENSITIES 74

5.10.8. *APOE* GENOTYPE 74

5.11. STATISTICAL PARAMETRIC MAPPING ANALYSES (SPM) 74

5.11.1. VBM VOLUMETRIC MRI ANALYSIS 75

5.11.2. FDG PET/CT ANALYSIS 75

5.11.3. ASL MR RCBF ANALYSIS 76

5.12. SAFETY ANALYSES 76

5.12.1. EXTENT OF DRUG EXPOSURE AND COMPLIANCE 76

5.12.2. ADVERSE EVENTS 78

5.12.3. LABORATORY DATA 81

5.12.4. VITAL SIGNS 85

5.12.5. PHYSICAL AND NEUROLOGICAL EXAMINATIONS 86

5.12.6. OPHTHALMOLOGICAL EXAMINATION 87

6. STATISTICAL ANALYSIS OF OPEN-LABEL TREATMENT PHASE 88

6.1. INTRODUCTION 88

6.2. OPEN-LABEL TREATMENT PHASE DEFINITIONS 88

6.3. SUMMARY STATISTICS 88

6.4. SAMPLE SIZE JUSTIFICATION 89

TauRx / Cytel Confidential: This is a controlled document and shall not be changed, saved, copied or reproduced by any unauthorized person.

6.5. PROTOCOL DEVIATIONS ... 89

6.6. VISIT WINDOWS ... 90

6.7. SUBJECT DISPOSITION ... 91

6.8. DEMOGRAPHIC AND BASELINE CHARACTERISTICS ... 92

6.9. PRIOR AND CONCOMITANT MEDICATIONS ... 92

6.10. SECONDARY EFFICACY ENDPOINT ANALYSIS ... 93

6.10.1. SENSITIVITY ANALYSIS ... 94

6.11. EXPLORATORY EFFICACY ANALYSIS ... 94

6.11.1. ADCS-ADL_{23} ... 95

6.11.2. MRI ... 95

6.12. POST HOC EFFICACY ANALYSIS ... 95

6.13. SAFETY ANALYSIS ... 97

6.13.1. EXTENT OF DRUG EXPOSURE AND COMPLIANCE ... 97

6.13.2. ADVERSE EVENTS ... 98

6.13.3. LABORATORY DATA ... 100

6.13.4. VITAL SIGNS ... 102

6.13.5. PHYSICAL AND NEUROLOGICAL EXAMINATIONS ... 102

6.13.6. OPHTHALMOLOGICAL EXAMINATION ... 102

7. PLASMA BIOMARKER ANALYSIS ... 103

7.1. BIOMARKER ... 103

7.1.1. BIOMARKER SAMPLES ... 104

7.2. ANALYSIS POPULATION ... 105

7.3. STATISTICAL ANALYSIS OF BIOMARKER OUTCOMES ... 105

7.3.1. SUMMARY STATISTICS ... 105

7.3.2. PRIMARY OUTCOMES ... 105

7.3.3. SECONDARY OUTCOMES ... 107

7.3.4. ADDITIONAL POTENTIAL BIOMARKERS ... 108

TauRx / Cytel Confidential: This is a controlled document and shall not be changed, saved, copied or reproduced by any unauthorized person.

7.3.5. ADDITIONAL ANALYSIS 108

7.4. FUTURE SAMPLE ANALYSIS 109

8. PHARMACOKINETIC EVALUATIONS 110

9. ADNI AND META ANALYSIS 111

10. CHANGES TO PLANNED ANALYSES 112

11. REFERENCES 113

12. APPENDIX 114

12.1. CONSIDERATIONS REGARDING COVID-19 114

12.1.1. FDA: STATISTICAL CONSIDERATIONS FOR CLINICAL TRIALS DURING THE COVID-19 PUBLIC HEALTH EMERGENCY 114

12.1.2. EMA: POINTS TO CONSIDER ON IMPLICATIONS OF CORONAVIRUS DISEASE (COVID-19) ON METHODOLOGICAL ASPECTS OF ONGOING CLINICAL TRIALS 116

12.2. SCALES 117

12.2.1. ADAS-COG SCORING 117

12.2.2. ADCS-ADL_{23} SCORING 118

12.2.3. MMSE 119

12.2.4. CDR-SOB 119

12.3. RULES FOR DETERMINING “WORST” VALUE 120

12.3.1. CLINICAL LABORATORY PARAMETERS 120

12.3.2. SCALES, VITAL SIGNS, AND WEIGHT MEASUREMENT 120

12.4. ATC CODES OR PREFERRED TERMS FOR MEDICATIONS 121

13. STATISTICAL OUTPUTS TO BE GENERATED 122

14. CLINICAL STUDY REPORT APPENDICES 123

TauRx / Cytel Confidential: This is a controlled document and shall not be changed, saved, copied or reproduced by any unauthorized person.

LIST OF IN-TEXT TABLES

Table		Page
Table 1	Protocol evolution	18
Table 2	Schedule of Assessments for double-blind treatment phase	25
Table 3	Schedule of Assessments for continued open-label treatment phase	28
Table 4	MRI and ^{18}F-FDG-PET parameters and their raw data pendants	31
Table 5	Evaluation Intervals for Safety and Efficacy Analyses of the Double-Blind Phase	50
Table 6	Laboratory – Potentially clinically significant values	83
Table 7	Laboratory – Related parameters	85
Table 8	Vital signs – Potentially clinically significant values	86
Table 9	Evaluation Intervals of the Open-Label Treatment Phase	90

TauRx / Cytel Confidential: This is a controlled document and shall not be changed, saved, copied or reproduced by any unauthorized person.

ABBREVIATIONS

Abbreviation	Definition
AChEI	Acetylcholinesterase Inhibitor
AD	Alzheimer's Disease
ADAS-cog_{13}	Alzheimer's Disease Assessment Scale – cognitive subscale (13-item)
ADCS-ADL_{23}	Alzheimer's Disease Cooperative Study – Activities of Daily Living (23-item)
ADNI	Alzheimer's Disease Neuroimaging Initiative
AE	Adverse Event
AESI	Adverse Event of Special Interest
ALT	Alanine Aminotransferase
ANCOVA	Analysis of Covariance
ApoE(4)	Apolipoprotein E(4) genotype
ASL	Arterial Spin Labeling
AST	Aspartate Aminotransferase
BDRM	Blinded Data Review Meeting
BSI	Boundary Shift Integral
CBF	Cerebral Blood Flow
CDR	Clinical Dementia Rating
CDR-SOB	Clinical Dementia Rating – Sum Of Boxes
Cmax,ss	Maximal concentration during steady state
CMH	Cochran-Mantel-Haenszel
COVID-19	Coronavirus Disease 2019 Public Health Emergency
CRF	Case Report Form
CSR	Clinical Study Report
DRM	Data Review Meeting
DSMB	Data and Safety Monitoring Board
ECG	Electrocardiogram
eCRF	Electronic Case Report Form
EDC	Electronic Data Capture

TauRx / Cytel Confidential: This is a controlled document and shall not be changed, saved, copied or reproduced by any unauthorized person.

Abbreviation	Definition
EMA	European Medicines Agency
E-MITT	Efficacy Modified Intention-To-Treat
EOT	End Of Treatment
ET	Early Termination
FCS	Fully Conditional Specification
FDA	Food and Drug Administration
^{18}F-FDG-PET	18F-fluorodeoxyglucose positron emission tomography
FWE	Familywise Error Rate
G6PD	Glucose-6-Phosphate Dehydrogenase
GFAP	Glial Fibrillary Acidic Protein (biomarker)
GGT	Gamma-Glutamyl Transferase
GLM	General Linear Models
HMTM	Hydromethylthionine Mesylate
ICE	Intercurrent Event
ICF	Informed Consent Form
ICH	International Council for Harmonisation of Technical Requirements for Pharmaceuticals for Human Use
ITT	Intention-To-Treat
LOQ	Limit Of Quantification
LSM	Least Squares Mean
MAOI	Monoamine Oxidase Inhibitor
MAR	Missing At Random
MCI-AD	Mild Cognitive Impairment due to AD
MDRD	Modification of Diet in Renal Disease
MedDRA	Medical Dictionary for Regulatory Activities
MHRA	Medicines & Healthcare products Regulatory Agency
MI-MITT	MRI Imaging Modified Intention-To-Treat
ML	Maximum Likelihood
MMRM	Mixed effect Model with Repeated Measurement
MMSE	Mini-Mental State Examination

TauRx / Cytel Confidential: This is a controlled document and shall not be changed, saved, copied or reproduced by any unauthorized person.

**Abbreviation**	**Definition**
MNI	Montreal Neurological Institute
MRI	Magnetic Resonance Imaging
MT	Methylthioninium
MTC	Methylthioninium Chloride
NfL	Neurofilament Light (biomarker)
OL	Open-Label
PCS	Potentially Clinically Significant
PK	Pharmacokinetic
PET	Positron Emission Tomography
PI	Principal Investigator
PI-MITT	PET Imaging Modified Intention-To-Treat
PT	Preferred Term
P-tau181	Tau phosphorylated at residue 181 (biomarker)
P-tau231	Tau phosphorylated at residue 231 (biomarker)
rCBF	Regional Cerebral Blood Flow
REML	Restricted Maximum Likelihood
ROI	Region Of Interest
RTF	Rich Text Format
RTSM	Randomization and Trial Supply Management
SAE	Serious Adverse Event
SAP	Statistical Analysis Plan
SAR	Serious Adverse Reaction
SAS®	Statistical Analysis System
SD	Standard Deviation
SNRI	Serotonin and Norepinephrine Reuptake Inhibitor
SOC	System Organ Class
SPM	Statistical Parametric Maps
SSRI	Selective Serotonin Reuptake Inhibitor
SUVR	Standardized Uptake Value Ratio
TC	Telephone Contact

TauRx / Cytel Confidential: This is a controlled document and shall not be changed, saved, copied or reproduced by any unauthorized person.

Abbreviation	Definition
TCA	Tricyclic Antidepressant
TEAE	Treatment Emergent Adverse Event
TICV	Total Intracranial Volume
TLV	Total Lesion Volume
TOTOS	The Off Treatment On Study (subjects)
TSH	Transmissible Spongiform Encephalopathy
T-tau	Total tau (biomarker)
UK	United Kingdom
VBM	Voxel Based Morphometry
vMRI	Volumetric Magnetic Resonance Imaging
WBC	White Blood Cell

TauRx / Cytel Confidential: This is a controlled document and shall not be changed, saved, copied or reproduced by any unauthorized person.

1. INTRODUCTION AND OBJECTIVES OF ANALYSIS

1.1. Introduction

An unmet need exists to develop new medications for Alzheimer's Disease (AD) that more directly modify the underlying disease pathology and offer longer-term and greater efficacy. HMTM (hydromethylthionine mesylate), the investigational product, is believed to have the potential to confer benefits over existing treatments for AD due to its ability to affect the process of tau aggregation responsible for the underlying neurofibrillary pathology of AD. Available nonclinical and clinical evidence supports the clinical evaluation of HMTM in AD.

Results from two phase 3 studies of HMTM on AD subjects (TRx-237-005, TRx-237-015) suggest that HMTM 8 mg/day given as monotherapy may be effective in delaying progression of mild to moderate AD on co-primary clinical efficacy endpoints, ADAS-cog_{11} and ADCS-ADL_{23}, and also on magnetic resonance imaging (MRI) measures of progression of brain atrophy and ^{18}F-FDG-PET (^{18}F-fluorodeoxyglucose positron emission tomography) measures of impairment in neuronal metabolic function, and that there is no added benefit to using doses substantially higher than 8 mg/day. Recent population pharmacokinetic (PK) studies have confirmed that there are concentration-response relationships for cognitive and neuroimaging outcomes at the 8 mg/day dose. This is seen whether HMTM is taken alone or as add-on to symptomatic treatments. In a within-cohort meta-analysis of both studies, HMTM at a dose of 8 mg/day as monotherapy was found to produce significant deceleration in the annualized rate of whole brain atrophy after 9 months of treatment. The magnitude of the concentration-dependent treatment effects was reduced when HMTM was given to subjects who were concurrently using acetylcholinesterase inhibitors (AChEIs) and/or memantine. The reduction in treatment effects of HMTM by treatment with an AChEI or memantine has been reproduced in a tau transgenic mouse model and appears to reflect a generalized homeostatic downregulation that is induced in multiple brain systems to compensate for the activating effects of symptomatic treatments.

In the continued development of HMTM, 16 mg/day has been chosen as the primary target dose. This was based on a population PK-response model that was applied to data from the earlier Phase 3 studies. The clearance of parent Methylthioninium (MT) is most significantly associated with renal function, suggesting that the most important predictor of whether or not a subject achieves a parent MT Cmax,ss above the threshold is creatinine clearance. At a dose of 8 mg/day, approximately 60% of subjects would be expected to have plasma concentrations above the threshold concentration that was identified with pharmacologic activity whereas at a dose of 16 mg/day, all subjects would. In order to provide some degree of urinary discoloration in what as originally a "true" placebo control group, a small amount of methylthioninium chloride (MTC), 4 mg, was introduced to placebo treatment kits (one tablet on two intermittent occasions twice per week, i.e., 8 mg/week). This amount of MTC had been anticipated to be without activity, consistent with results of a prior Phase 2 trial and estimated plasma

TauRx / Cytel Confidential: This is a controlled document and shall not be changed, saved, copied or reproduced by any unauthorized person.

half-life. For this reason, this treatment arm is referred to herein (and in tables, listings, and figures) as “control” rather than “placebo”.

This document describes the plan for the full statistical analyses and reporting of study TRx-237-039, which in its current version is a randomized, double-blind, controlled, three-arm, 12-month, safety and efficacy study of HMTM monotherapy in subjects with AD followed by a 12-month open-label treatment. Results of the analyses described in this Statistical Analysis Plan (SAP) will be included in the Clinical Study Report (CSR).

The planned analyses identified in this SAP will be included in regulatory submissions and future manuscripts. Any post-hoc or unplanned analyses performed to provide results for inclusion in the CSR but not identified in this prospective SAP, will be clearly identified in the CSR.

This SAP is primarily based on the protocol version 7.0 from 28-JUL-2021 and version 7.0 UK from 19-AUG-2021. Key decisions based on earlier versions are acknowledged where appropriate.

The statistical analysis of pharmacokinetic data will be described in a separate SAP (responsible: Certara USA, Inc.; 100 Overlook Center, Suite 101; Princeton, NJ 08540; United States), but will be included/referenced in the CSR.

1.2. Objectives of Statistical Analysis

The primary objectives of the study TRx-237-039 are to evaluate the efficacy and safety of HMTM (16 mg/day) over up to two years of treatment, depending on the initially randomized treatment group, compared to the control arm.

This SAP is designed to outline the methods to be used in the analysis of study data to answer the study objectives. Populations for analysis, data handling rules, statistical methods, and formats for data presentation, are provided.

1.3. Study Protocol Versions

The following table shows an overview of the differences in duration, population, sample size and randomization ratios, and planned doses groups between the study protocol versions.

TauRx / Cytel Confidential: This is a controlled document and shall not be changed, saved, copied or reproduced by any unauthorized person.

Table 1 Protocol evolution

	Version 2.1	Version 3.0 – 4.1	Version ≥5.0
**Duration of study**	6 months (double-blind)	9 months (double-blind)	12 months (double-blind) + 12 months (open-label)
**Population**	Mild AD<br><br>(MMSE 20-25)	MCI-AD, or mild AD<br><br>(MMSE 20-27)<br>PET positive for amyloid	MCI-AD, mild AD, or moderate AD<br>(MMSE 16-27)<br>PET positive for amyloid
**Target enrollment (randomization ratio according to doses below)**	180<br>(1:1)	375<br>(2:2:1)	500 (≥v5.0: 450)<br>(1:4:4)
**Planned doses (sample size)**	HMTM 8 mg/day (N=90)<br>Placebo (N=90)	HMTM 8 mg/day (N=150)<br>Placebo (N=150)<br>HMTM 16 mg/day (N=75)	HMTM 8 mg/day (N=50)<br>Placebo/MTC (N=200)<br>HMTM 16 mg/day (N=200)

The primary efficacy analysis is performed based on the subjects randomized under protocol version 5.0 or higher, in which some MTC tablets were introduced to placebo treatment kits (on average twice per week spiking) as urinary discolorant to maintain the treatment blinding. In a sensitivity analysis, all randomized subjects are analyzed. Safety assessment will be performed based on all randomized subjects as well.

1.4. UK Versions of Study Protocol

The protocol (version 5.1, 05-FEB-2020) was adapted in response to requests made by the Medicines & Healthcare products Regulatory Agency (MHRA), after the inclusion of moderate AD subjects with protocol version 5.0.

The resulting first UK-specific version is protocol 5.2 UK (02-MAR-2020), which requires that subjects be either treatment naïve or to have already discontinued prior treatment with AChEI and/or memantine due to intolerance, lack of efficacy, or recommendation by the primary care provider prior to giving consent to participate in the study. The other major change is to require that the first visit in the newly

TauRx / Cytel Confidential: This is a controlled document and shall not be changed, saved, copied or reproduced by any unauthorized person.

added open-label delayed-start period (after 2 weeks, V8) be an in-clinic visit rather than a telephone contact given that some subjects will be receiving HMTM treatment for the first time.

For all subsequent UK versions, no additional country specific changes were implemented.

1.5. **Interim Analysis**

An early efficacy analysis is planned prior to the completion of the study.

The initial 52-week, double-blind, controlled treatment phase is completed when the last subject completes the final visit in the double-blind treatment phase (Week 52 visit, V7, pre-dose procedures). Then, the database will be partially locked and unblinded for analysis (see following Section 1.6). Treatment assignment will not be divulged to subjects or individuals involved in the operational conduct on-site of the ongoing open-label treatment phase. For further information regarding unblinding, see Section 2.7.

The complete CSR will be prepared upon completion of the entire study.

COVID-19 related changes to the study are documented as specified in the versions 6.0 and 6.0 UK or later versions of the protocol. During the study, the impact of COVID-19 on the study was closely monitored by the Sponsor as per a separate analysis plan (iSAP, v1.0, 28-MAY-2021) in relation to drop-out rates and site reported (in Medidata RAVE) impact on assessments and endpoints. No adjustments to the study design, sample size or conduct were made as a result of this monitoring.

1.6. **Database Locks**

For future references, all data from data snapshots and database locks, which are described below, will be saved and are not to be changed.

To perform the analysis of the double-blind treatment phase, a data snapshot will be done after all outstanding queries concerning double-blind treatment phase data are resolved. All case report forms (CRFs) will be approved by the Principal Investigator (PI). After that, double-blind treatment phase data must not be edited or added, except for updating data related to safety, which can be found in CRFs including but not limited to Medical History, Concomitant Medications, Procedures, Study Drug Interruption Log and Serious Adverse Events (SAEs). For the analysis of the double-blind treatment phase, the following data will be included:

- All (efficacy) data until the last visit of the double-blind treatment phase,
- Eye exam, if it was performed until the end of the Week 52 protocol-specified visit window,
- Any further (safety) data up until the receipt of the first dose of the open-label treatment phase drug, and

TauRx / Cytel Confidential: This is a controlled document and shall not be changed, saved, copied or reproduced by any unauthorized person.

- Data of imaging parameters (MRI, ^{18}F-FDG-PET) assessed within 14 days after first dose of the open-label treatment phase drug.

After the last subject visit of the open-label treatment phase, the database will be locked in accordance with the Database Lock Plan. Data from previously locked CRFs will be checked to capture any potential changes in data, by comparing SDTM and/or ADaM datasets, and/or re-running the analysis of the double-blind treatment phase based on the final database lock data; discrepancies will be listed. For the analysis of the open-label treatment phase, all data until the end of the study will be included. Full listings will be re-run including data from the double-blind and open-label treatment phases.

TauRx / Cytel Confidential: This is a controlled document and shall not be changed, saved, copied or reproduced by any unauthorized person.

2. STUDY DESIGN

2.1. Synopsis of Study Design

This is a two-phase outpatient study of HMTM administered as monotherapy in approximately 500 subjects (450 under protocol version 5.0 or higher) with early to mild-moderate AD: a randomized, double-blind, controlled, 52-week treatment phase followed by a 52-week open-label treatment phase that represents a modified delayed start of treatment. Subjects[a] for whom legally acceptable informed consent was obtained and who were found eligible on the basis of Screening evaluations, were randomly assigned at Baseline to receive either HMTM 16 mg/day, HMTM 8 mg/day, or control (i.e., placebo/MTC) (4:1:4, at the study level); as noted earlier, the drug supplies for the control group included tablets containing a urinary discolorant (MTC), 4 mg, dosed at an average frequency of two tablets per week. The primary treatment group comparison during the double-blind treatment phase is between HMTM 16 mg/day and control. Following completion of the 52-week treatment phase, all subjects (regardless of randomized treatment assignment or response) will continue open-label treatment with HMTM 16 mg/day for a further 52 weeks.

[a] Not receiving concomitant AChEI and/or memantine (required for patients in the UK) or otherwise receiving concomitant AChEI and/or memantine at the time of signing informed consent and agreeing to discontinue them before randomization (see Section 2.2).

Figure 1 Schematic of study design (Protocol version 5.0+)

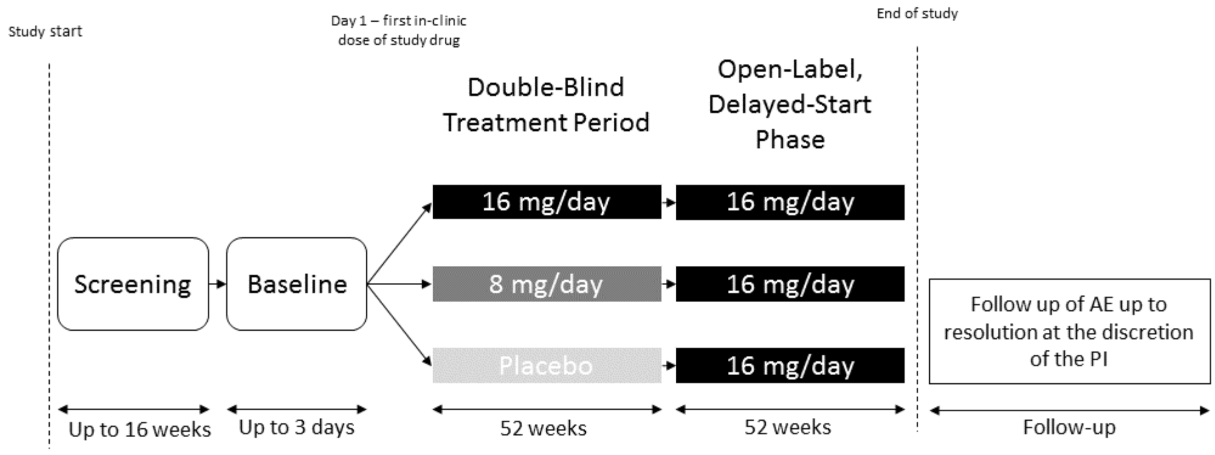

TauRx / Cytel Confidential: This is a controlled document and shall not be changed, saved, copied or reproduced by any unauthorized person.

2.2. Screening Period

The Screening period is to be up to 9 weeks for subjects not receiving an AChEI and/or memantine at the time of signing the consent (Initial Screening Visit, Visit 1). For subjects receiving an AChEI and/or memantine, the Screening period may be extended for up to a further 6 weeks, 15 weeks in total (+7 days at Sponsor discretion), to allow for the performance of the necessary Screening tests prior to the discontinuation of an AChEI and/or memantine and to permit a washout of at least 60 days from the last dose prior to the Baseline assessments (inclusive of the Baseline ^{18}F-FDG-PET scan in subjects who have a Screening Clinical Dementia Rating (CDR) of 0.5). Signing of the initial informed consent form (ICF) is assigned to the screening period irrespective of when it occurred.

The Screening period could be extended for COVID-19 related reasons as specified in the versions 6.0 and 6.0 UK, or later versions of the protocol.

For re-screened subjects, information from the previous screenings will be included and is flagged in the subject data listings. In case of screening data being used for analysis, the latest available screening information, including previous screenings, will be used.

2.3. Double-Blind Treatment Phase (Week 0 up to Week 52)

Five post-Baseline visits are scheduled during the double-blind treatment phase (Visit 3 (Week 4): safety, and Visits 4, 5, 6, and 7 (Week 13, 26, 39, and 52): efficacy, imaging, and safety, see Table 2).

Unscheduled visits may occur as needed for assessment, or upon early termination. In addition, subjects are to be followed as needed for the resolution or stabilization of an AE, including following the last dose, consistent with the Investigator’s medical judgement.

The Baseline value is defined as the last non-missing value prior to first dose of study drug or value obtained on the same day of the first dose of study drug. In case of pre- and post-dose assessments at the Baseline visit, the pre-dose value will be used as the Baseline assessment. If no assignment to pre-/post-dose is possible algorithmically, it will be treated as pre-dose unless specified otherwise during the Blinded Data Review Meeting (BDRM).

2.4. Open-Label Treatment Phase (Week 52 up to Week 104)

During the open-label treatment phase, three visits are scheduled in-clinic unless otherwise noted: Visit 8 at 56 weeks (telephone contact only for all but UK sites), Visit 9 at 78 weeks, and Visit 10 at 104 weeks. The last non-missing value/assessment prior to first dose of the open-label treatment phase serves as the Baseline visit for the open-label treatment phase, but subjects with a missing Week 52 efficacy endpoint are excluded from the respective non-inferiority PPv5-OL population (see Section 3.1.2).

TauRx / Cytel Confidential: This is a controlled document and shall not be changed, saved, copied or reproduced by any unauthorized person.

2.5. Changes of Study Conduct due to COVID-19

The protocol was revised to version 6.0 and 6.0 UK to incorporate modifications to the study conduct and monitoring, guidance for continued data collection and analysis, and ongoing risk assessment due to the Coronavirus Disease 2019 Public Health Emergency (COVID-19). Key assessments deciding the eligibility of subjects as well as Baseline, Week 52, Week 56 for UK sites, and early termination assessments must be done on-site and cannot be assessed remotely. Other visits can be done remotely as per the agreed risk assessments and data collection plans.

Since ^{18}F-FDG-PET was not needed to determine study eligibility and is not the primary efficacy imaging, the Sponsor may have approved subject randomization without a brain ^{18}F-FDG-PET scan if the scan could not be performed due to COVID-19. For imaging (MRI, ^{18}F-FDG-PET), alternative scanners will only be accepted if data comparability is confirmed and ensured prior to assessments being made; every effort was made to avoid any change in scanner for a given site and subject over the course of the study. If a Baseline ^{18}F-FDG-PET scan could not be done due to lack of imaging facility, a follow-up ^{18}F-FDG-PET scan was not required.

If a subject underwent Screening assessments but could not have protocol-required assessments performed due to COVID-19, the subject could be put on screening pause until such times as COVID-19 impacts had ceased (abstention from treatment with an AChEI and/or memantine should have been retained).

The planned in-clinic post-Baseline visits (except Week 52 visit), including blood samples for safety laboratory assessments as well as optional genotyping, may have been completed at home, or at other safe, suitable alternative location if deemed necessary to protect subjects due to COVID-19.

Study visit windows may have been extended beyond the allowed +/- 14 days at the discretion of the Sponsor when justification was provided and unless two planned study visits did not fall into the same visit window as per Sections 5.5 / 6.5.

Efficacy scales may have also been performed remotely, if approved by the Sponsor in advance on a case-by-case basis.

Dose interruption not triggered by the site clinician because of COVID-19 might be allowed for more than 14 days at the discretion of the Sponsor.

2.6. Randomization Methodology

With protocol version 5.0 (see Table 1), subjects who satisfied all eligibility criteria were randomized to one of three study regimens HMTM 16 mg/day, HMTM 8 mg/day, or control (4:1:4, at the study- rather than site-level). Randomization was stratified by (**bold** level will be used as reference category in statistical analyses, if needed and results are not population weighted):

TauRx / Cytel Confidential: This is a controlled document and shall not be changed, saved, copied or reproduced by any unauthorized person.

- Severity (three levels: Mini-Mental State Examination (MMSE) **16-19 (moderate)**, 20-25 (mild), or 26-27 (MCI-AD)),
- Prior use of standard AD treatment (two levels: AChEI/memantine or **none**), and
- Region (two levels: North America or **Europe**).

Global enrollment across the whole study was managed such that subjects were assigned to the Screening MMSE severity groups with a target of approximately 2:3:1 (MMSE 16-19, MMSE 20-25, and MMSE 26-27, respectively) for those randomized under protocol version 5.0 and above. Each stratum was closed when the target enrollment was achieved.

If not indicated otherwise, any analysis or summary that groups subjects by prior use of AChEI and/or memantine, is inclusive of those with a prior history of such use and those who withdrew usage for the purposes of this study (such as lack of efficacy). When data are analyzed descriptively, discontinuation of AChEI and/or memantine before entering the study and discontinuation of AChEI and/or memantine for purposes of study participation (after entering the study) will be differentiated.

If a subject consented to protocol version 2.1 and did not reconsent to a later protocol version, the stratification information provided by the site is considered correct with respect to prior use of AChEI and/or memantine, unless there is specific information in the CRF that contradicts that. In earlier version of the protocol (v2.1), there was no stratum for MMSE as the inclusion criterion was to have an MMSE of 20-25; for all these subjects the stratum will be imputed as MMSE 20-25.

2.7. Stopping Rules and Unblinding

The randomization list for the double-blind treatment phase is maintained within the Randomization and Trial Supply Management system (RTSM, hosted by Clario previously known as BioClinica) and in secure locations by individuals who are not directly involved in the conduct of the study. The blind for an individual subject should not be broken during conduct of the study except in the case of a medical emergency for which it is deemed essential to know which treatment the subject has received during the double-blind treatment phase to provide appropriate care. Then, the Investigator may unblind a specific subject and determine the identity of treatment using the RTSM System. This will be achieved via a “peek blind” function within this system, whereby an end user with appropriate access can view the unblinded treatment group on screen; completion of the peek blind transaction will reinstate the blinded status of the subject. For any such affected subject, study drug will be discontinued, and the subject will be followed until resolution or stabilization of the event and then discontinued from study.

After the initial 52-week, double-blind, controlled treatment phase is completed, a data snapshot will be done (see Section 1.6) and unblinded for analysis. It will be an interim lock, because some electronic case report form (eCRF) pages might need to be reopened to allow for resolution of AEs for instance; a version of all raw data for the analysis of the double-blind treatment phase will be stored; prior to the final database lock of the study, a comparison with the stored data from the interim lock will be

TauRx / Cytel Confidential: This is a controlled document and shall not be changed, saved, copied or reproduced by any unauthorized person.

performed and changes will be identified/followed-up. Treatment assignment will not be divulged to subjects or individuals involved in the operational conduct on-site of the ongoing open-label treatment phase.

The study is monitored for safety by a blinded Data and Safety Monitoring Board (DSMB) throughout its duration. At any time, the DSMB may recommend that the study may continue, with or without modifications, or be terminated due to safety concerns. The DSMB may also request to receive additional data unblinded to the subject level in response to identified safety concerns.

Further information about blinded and unblinded personnel during the conduct of the study is given in the document *Study Blind Maintenance Plan for TRx-237-039* (current version 3.0, 27-OCT-2022) – a Sponsor controlled document.

2.8. **Study Procedures**

The schedule of assessments for double-blind and open-label treatment phase, as outlined in the study protocol, is provided in Table 2 and Table 3, respectively.

Table 2 Schedule of Assessments for double-blind treatment phase

TauRx / Cytel *Confidential: This is a controlled document and shall not be changed, saved, copied or reproduced by any unauthorized person.*

Visit Name	Screening	Baseline		Double-Blind Treatment Phase				
Visit Number	1	2		3	4	5	6	7 (or ET)
Weeks Relative to Baseline	≥ -9 weeks	-		4 weeks	13 weeks	26 weeks	39 weeks	52 weeks
Allowable Time Window in Days		(+3) Pre-Dose	Post-Dose	(±3)	(±14)	(±14)	(±14)	(±14)
Likely diagnosis of probable AD or MCI-AD, and confirmation	X							
Informed consent by Subject (and/or LAR) and Study Partner(s)	X							
Demographics	X							
Medical history and concomitant medication review	X							
12-lead electrocardiogram	X							
Amyloid PET scan	X							
^{18}F-FDG-PET	X							X
Randomization		X						
MRI	X				X	X	X	X
ADAS-cog$_{13}$ and ADCS-ADL$_{23}$		X			X	X	X	X
AE/Concomitant Medication Recording/Review		X	X	X	X	X	X	X
Physical/Neurological Examinations	X							
Targeted Physical/Neurological Examinations		X	X	X	X	X	X	X
Ophthalmological Examination	X	X						X

TauRx / Cytel *Confidential: This is a controlled document and shall not be changed, saved, copied or reproduced by any unauthorized person.*

**Visit Name**	**Screening**	**Baseline**		**Double-Blind Treatment Phase**				
**Visit Number**	**1**	**2**		**3**	**4**	**5**	**6**	**7 (or ET)**
**Weeks Relative to Baseline**	**≥ -9 weeks**	**-**		**4 weeks**	**13 weeks**	**26 weeks**	**39 weeks**	**52 weeks**
**Allowable Time Window in Days**		**(+3)**		**(±3)**	**(±14)**	**(±14)**	**(±14)**	**(±14)**
		**Pre-Dose**	**Post-Dose**					
Clinical Laboratory testing	X	X		X	X	X	X	X
Pregnancy Testing	X	X		X	X	X	X	X
Blood Pressure, Pulse, Body Weight	X	X	X	X	X	X	X	X
Study Drug Dispensing		X			X	X	X	X
Study Drug Compliance Assessment				X	X	X	X	X
Blood Sample for MT Concentration		X	X	X				X
Blood Sample for Genotyping (optional)		X						
MMSE	X							X
CDR	X							X

TauRx / Cytel Confidential: This is a controlled document and shall not be changed, saved, copied or reproduced by any unauthorized person.

Table 3 Schedule of Assessments for continued open-label treatment phase

Visit Name	Baseline/Day 1 for Open-Label Phase		Continued Open-Label Treatment Phase		
Visit Number	7		8 (TC) [a]	9	10 (OL-EOT or OL-ET)
Weeks Relative to Baseline	52 weeks		56 weeks	78 weeks	104 weeks
Allowable Time Window in Days	(±14)		(±3)	(±14)	(±14)
	Pre-Dose	Post-Dose			
^{18}F-FDG-PET	X				
MRI	X			X	X
ADAS-cog$_{13}$ and ADCS-ADL$_{23}$	X			X	X
AE/Concomitant Medication Recording/Review	X	X	X	X	X
Targeted Physical/Neurological Examinations	X		X [a]	X	X
Ophthalmological Examination	X				X
Clinical Laboratory testing	X		X [a]	X	X
Pregnancy Testing	X		X [a]	X	X
Blood Pressure, Pulse, Body Weight	X		X [a]	X	X
Study Drug Dispensing	X			X	
Study Drug Compliance Assessment	X		X	X	X
Blood Sample for MT Concentration	X	X			X
MMSE	X				X
CDR	X				X

TauRx / Cytel Confidential: This is a controlled document and shall not be changed, saved, copied or reproduced by any unauthorized person.

[a] Onsite for UK sites, and telephone contact (TC) for non-UK sites. Scheduled onsite assessment for UK sites only.

TauRx / Cytel Confidential: This is a controlled document and shall not be changed, saved, copied or reproduced by any unauthorized person.

2.9. Efficacy, Pharmacokinetic, Safety, and Other Variables

2.9.1. Efficacy Variables

2.9.1.1. Primary Efficacy Variables

Primary efficacy variables are (assessed at Baseline, 13, 26, 39, 52, 78, and 104 weeks, or early termination):

- ADAS-cog_{11}
- ADCS-ADL_{23}

The **ADAS-cog_{11}** is the cognitive subscale of the ADAS, originally proposed with 11 items (domains: memory, praxis, orientation, and language), resulting in scores that range from 0 to 70, with higher numbers indicating greater impairment. For the ADAS-cog_{13} two additional items have been added (in domain Memory: Delayed Word Recall, and as a new domain Attention: Number Cancellation) to provide additional sensitivity to change in cognition at earlier stages of the disease, resulting in a maximal score of 85 (see Appendix 12.2). As the original ADAS-cog_{11} was used in the earlier Phase 3 studies (TRx-237-005, TRx-237-015), that score will be derived from the assessment of the ADAS-cog_{13} for the primary analyses. ADAS-cog_{13} will be analyzed as an explorative efficacy variable.

The **ADCS-ADL_{23}** includes 23 items describing the performance of activities of daily living (ADL) by AD subjects with scores ranging from 0 to 78 and higher numbers indicating lesser impairment.

2.9.1.2. Secondary Efficacy Variables

Secondary efficacy variables are:

- Brain MRI evaluated for whole brain volume and temporoparietal lobe volume (assessed at Screening, 13, 26, 39, 52, 78, and 104 weeks, or early termination).
- Brain ^{18}F-FDG-PET evaluated for temporal lobe, in Standardized Uptake Value Ratio (SUVR) normalized to pons in subjects with CDR of 0.5 (assessed at Screening and 52 weeks, or early termination).

Change in MRI volumetric parameters and change in ^{18}F-FDG-PET SUVR parameters are quantified by imaging core laboratories (MRI: Clario previously known as BioClinica, ^{18}F-FDG-PET: Invicro).

If documented volumes are split into left and right area, these two values will be averaged to get the total value. For the MRI temporoparietal lobe volume, temporal lobe volume and parietal lobe volume will be averaged also.

For the open-label treatment phase, only ADAS-cog_{11} will serve as a secondary endpoint; ADCS-ADL_{23} and other imaging endpoints are exploratory with the aim to be directionally supportive.

TauRx / Cytel Confidential: This is a controlled document and shall not be changed, saved, copied or reproduced by any unauthorized person.

Table 4 MRI and ^{18}F-FDG-PET parameters and their raw data pendants

	Parameter	Raw data variable(s)
MRI	Whole brain	BILATERAL WHOLE BRAIN
	Temporoparietal lobe	LEFT TEMPORAL LOBE<br>RIGHT TEMPORAL LOBE<br>LEFT PARIETAL LOBE<br>RIGHT PARIETAL LOBE
	Lateral ventricular	VENTRICLES
	Hippocampal	LEFT HIPPOCAMPUS<br>RIGHT HIPPOCAMPUS
	Putamen	LEFT PUTAMEN<br>RIGHT PUTAMEN
	Nucleus accumbens	LEFT ACCUMBENS AREA<br>RIGHT ACCUMBENS AREA
	Nucleus basalis	NUCLEUS BASALIS
	Total lesion volume	WHITEMATTER HYPOINTENSITIES
^{18}F-FDG-PET	Temporal lobe	TEMPORAL_CORTEX_L<br>TEMPORAL_CORTEX_R
	Parietal lobe	PARIETAL_CORTEX_L<br>PARIETAL_CORTEX_R
	Frontal lobe	FRONTAL_CORTEX_L<br>FRONTAL_CORTEX_R
	Cerebellum	MEAN_CEREBELLUM_GRAY
	Anterior cingulate gyrus	ANTERIOR_CINGULUM_L<br>ANTERIOR_CINGULUM_R

TauRx / Cytel Confidential: This is a controlled document and shall not be changed, saved, copied or reproduced by any unauthorized person.

	Parameter	Raw data variable(s)
	Posterior cingulate gyrus	POSTERIOR_CINGULUM_L<br>POSTERIOR_CINGULUM_R

2.9.1.3. Exploratory Efficacy Variables

Exploratory efficacy variables are:

- MMSE (assessed at Screening and 52 weeks, or early termination)
- CDR (levels: 0.5, 1, 2, 3) and CDR – Sum Of Boxes (CDR-SOB) (range: 0 to 18) (assessed at Screening, 52, and 104 weeks, or early termination)
- ADAS-cog$_{13}$ (assessed at Baseline pre-dose, 13, 26, 39, 52, 78, and 104 weeks, or early termination).
- Composite Scale (analyzed at 39 and 52 weeks).
  The Composite Scale is the sum of cognitive subdomains from ADAS-cog$_{11}$ (Word Recall [score: 10], Constructional Praxis [5], Orientation [8], Spoken Language Ability [5], and Comprehension [5]) and functional items from ADCS-ADL$_{23}$ (Use of telephone [5], Keeping appointments [3], Cooking and preparation of meals [4], and Cleaning dishes [3]), resulting in a maximum possible score of 48 (higher score indicates less impairment). Since a higher score for ADAS-cog$_{11}$ indicates more impairment (less impairment for ADCS-ADL$_{23}$), the values of ADAS-cog$_{11}$ will be transformed (maximal score minus actual score) before summarizing both assessments.
- COVID-19 Composite Scale (see Sections 12.2.1, and 12.2.2)
- Brain MRI evaluated for lateral ventricular, hippocampal, putamen, nucleus accumbens, nucleus basalis, and further region of interest (ROI) volumes
- Brain ^{18}F-FDG-PET evaluated for temporal lobe in SUVR normalized to cerebellum, and parietal lobe, and frontal lobe, both in SUVR normalized to pons and normalized to cerebellum in subjects with CDR of 0.5 (assessed at Screening and 52 weeks, or early termination). In addition, further parameters to evaluate are cerebellum in SUVR normalized to pons; and anterior and posterior cingulate gyrus in SUVR normalized to pons and normalized to cerebellum.

2.9.2. Pharmacokinetic Variables

Plasma MT and whole blood concentrations (parent MT, *N*-desmethyl MT, and total MT, to the extent possible) are determined at Baseline, Week 4, Week 52, and Week 104, or early termination. Samples are collected prior to dosing (in clinic), approximately 1 to 2 hours post-dose and approximately 4 hours

TauRx / Cytel Confidential: This is a controlled document and shall not be changed, saved, copied or reproduced by any unauthorized person.

post-dose. At the early termination visit, a single blood sample is collected only for determination of MT concentrations, if the subject had already discontinued study drug or was unwilling to take a final in-clinic dose.

The statistical analysis of PK data performed by Certara USA, Inc., is not within the scope of this SAP and will be described in a separate document.

2.9.3. Safety Variables

Safety assessments performed during the study include the following:

- **Blood pressure** and **pulse** measured at Screening, Baseline pre-dose and approximately 2 hours post-dose, 4, 13, 26, 39, 52, 78, and 104 weeks, or early termination
- **Weight** measured at Screening, 4, 13, 26, 39, 52, 78, and 104 weeks, or early termination
- Standard **clinical laboratory** testing, including **hematology** and **blood (serum) chemistry**, performed at Screening, 4, 13, 26, 39, 52, 78, and 104 weeks, or early termination

  TSH, vitamin B12, folate, haptoglobin, and G6PD measured at Screening; a thyroid panel may be obtained in response to an elevated TSH. Additional testing as needed in response to an AE.
- Blood sample for a **serum pregnancy test** at Screening, 4, 13, 26, 39, 52, 78, and 104 weeks, or early termination, and up to 3 months after last dose of study drug; in women of childbearing potential, such women should be encouraged to return to the clinic or request serum pregnancy testing in the event of a delayed menstrual period to rule out possible pregnancy.
- **Targeted physical and neurological examinations** performed pre-dose and approximately 3 hours after administration of the first dose of study drug (Baseline). Thereafter, performed at 4, 13, 26, 39, 52, 78, and 104 weeks, or early termination. At a minimum, targeted examinations should include heart and lung auscultation and brief neurological assessment guided by any reported signs/symptoms/AEs (e.g., evaluating subjects for potential serotonin toxicity).
- **Ophthalmological examination** of subjects with history of lens implants performed prior to the first dose of study drug and at Week 52 and Week 104, to assess whether the lens has been discolored during the trial.
- Recording of **medications** administered within the last 90 days before Screening. Except for anti-dementia medications, where lifetime use (as far as possible) is to be recorded. Changes in concomitant medications and any new medications will be recorded at all subsequent visits, including the telephone contact.
- **AEs** recorded from the time the informed consent was signed throughout the study and, if pertinent, until resolution of the event. AEs with an onset after the first dose of study drug, or that worsen in intensity or treatment relationship after the first dose will be considered treatment-emergent adverse events (TEAE).

TauRx / Cytel Confidential: This is a controlled document and shall not be changed, saved, copied or reproduced by any unauthorized person.

2.9.4. Other Variables

Other assessments are:

- A single blood sample for Apolipoprotein E gene (*ApoE*) obtained from subjects who provide legally acceptable informed consent, collected at any time after eligibility for randomization and continued participation in the study has been confirmed but prior to Week 52.
- Other biomarkers will be analyzed as well. Their analysis is pre-specified in the separate Research Plan "TRx-237-039_Secondary Research Plan_Plasma Biomarker Analysis_v2.2_16Mar23". The analysis to be performed is described in more detail in Section 7 of this SAP.
- MRI arterial spin labelling parameters will be provided in a subject data listing.

TauRx / Cytel Confidential: This is a controlled document and shall not be changed, saved, copied or reproduced by any unauthorized person.

3. SUBJECT POPULATIONS

3.1. Population Definitions

The following subject populations will be evaluated and used for presentation and analysis of the data.

3.1.1. Double-Blind Populations

Intention-to-Treat (ITT) Population: all randomized subjects. Subjects are analyzed as randomized.

Efficacy Modified Intention-to-Treat (E-MITT) Population: all ITT subjects who received at least one dose of study drug and have a Baseline and at least one *valid* post-Baseline efficacy assessment (either ADAS-cog_{11} or ADCS-ADL_{23}). Subjects are analyzed as randomized.

MRI Imaging Modified Intention-to-Treat (MI-MITT) Population: all ITT subjects who received at least one dose of study drug and have a Baseline and at least one *valid* post-Baseline volumetric MRI (either whole brain or temporoparietal lobe). Subjects are analyzed as randomized.

PET Imaging Modified Intention-to-Treat (PI-MITT) Population: all ITT subjects with screening CDR of 0.5 and who received at least one dose of study drug and have a Baseline and at least one *valid* post-Baseline SUVR assessment (temporal lobe). If data is available from subjects with a CDR > 0.5, these subjects will not be included in this population. Subjects are analyzed as randomized.

Per Protocol (PP) Population: all subjects who are in the E-MITT and MI-MITT population, without any important protocol deviation (see Section 3.2) that would deem the subject exclusionary from the PP Population (as per BDRM decision) or intercurrent medical events that could confound the interpretations. Subjects are analyzed as randomized. **Important here refers to a classification that is decided during BDRM based on the original protocol deviation classification as major/minor and their impact on key (efficacy/safety) outcomes of this study. The original classification as major/minor will be provided as well.**

A valid value means that the assessment was not affected by prior initiation of an AChEI and/or memantine.

Safety Population: all subjects who received at least one dose of study drug. Subjects are analyzed as treated. If a subject received different doses, the predominant treatment group (based on the number of doses) will be assigned.

v5-Populations: If the above-mentioned populations (ITT, E-MITT, MI-MITT, PI-MITT, PP, Safety) are restricted to subjects randomized under study protocol version 5.0 or higher, which are the primary efficacy analysis populations, the population names are: ITTv5, E-MITTv5, MI-MITTv5, PI-MITTv5, PPv5, and Safetyv5.

Pharmacokinetic (PK) Population: all subjects who were administered at least one dose of study drug and have at least one analyzable post-dose PK sample.

TauRx / Cytel Confidential: This is a controlled document and shall not be changed, saved, copied or reproduced by any unauthorized person.

The analysis of estimands is based on the ITT population. For the FDA, the modified endpoint specific ITT populations (MITTs) will be analyzed. The Safety population is the primary population for the analysis of safety endpoints.

Two population trackers will be generated and will be provided to Cytel for the use in the analyses. The first as a result of the double-blind treatment phase BDRM, and the second as a result of the open-label treatment phase Data Review Meeting (DRM). These trackers will specify if subjects are included or need to be excluded from certain populations (e.g., TOTOS, PP, PP-OL). The population tracker from the BDRM will also specify if dropouts are potentially related to treatment.

3.1.2. Open-Label Populations

The following subject populations will be evaluated and used for presentation and analysis of the open-label data:

Intention-to-Treat Open-Label (ITT-OL) Population: all randomized subjects who received at least one dose of study drug from the open-label treatment phase or continued off-treatment during the open-label treatment phase (e.g., TOTOS subjects from the double-blind treatment phase). Subjects are analyzed as randomized.

Per Protocol Open-Label (PP-OL) Population: all randomized subjects who received at least one dose of study drug from the open-label treatment phase and without any important protocol deviation during the open-label treatment phase. Subjects are analyzed as randomized.

Safety Open-Label (Safety-OL) Population: all subjects who received at least one dose of study drug from the open-label treatment phase. Subjects are analyzed as treated.

Pharmacokinetic Open-Label (PK-OL) Population: all subjects who were administered at least one dose of study drug from the open-label treatment phase and have a subsequent analyzable open-label treatment phase PK sample.

v5-Populations: If the above-mentioned populations (e.g., ITT, and PP) are restricted to subjects randomized under study protocol version 5.0 or higher, which are the primary efficacy analysis populations, the population names are: ITTv5-OL, and PPv5-OL.

3.1.3. HMTM Treatment Phase Population

Safety HMTM (Safety-HMTM) Population: all subjects with at least one dose of HMTM study drug. This means that control subjects (who received placebo and/or MTC 8 mg/week) not participating in the open-label treatment phase will be excluded. Subjects are analyzed as treated.

3.2. Protocol Deviations

The Sponsor, or designee, will be responsible for producing the final protocol deviations file and for determining what kind of deviations will be considered important and which of these require the

TauRx / Cytel Confidential: This is a controlled document and shall not be changed, saved, copied or reproduced by any unauthorized person.

subject to be excluded from the PP population. This file will include a description of the protocol deviation, will provide the information regarding previous classification (minor/major), will provide the classification into important/non-important done by the Sponsor, and will be finalized prior to unblinding of the double-blind treatment phase. The level of detail and information provided will allow full traceability.

All protocol deviations will be presented in a subject data listing (by Synteract, the vendor of the RAVE EDC clinical database; as well as via a Sponsor protocol deviation Log, which includes the manual generated protocol deviations that cannot be assigned to a specific subject data point). This listing will be presented pooled (COVID-19 and non-COVID-19 related protocol deviations) and separate for COVID-19- and non-COVID-19-related protocol deviations.

Summaries by categorization (important/non-important) and type (or code) will be provided by treatment phase. This will be done pooled and separate for COVID-19- and non-COVID-19-related protocol deviations. Also, one version will count all protocol deviations and one version will count only the overall unique deviations (when one deviation is resulting in subsequent ones).

Some examples for protocol deviations are:

- Protocol deviations related to Inclusion/Exclusion criteria (ineligible subject randomized to the study)
- Randomization/drug dispensation errors (incorrect kit dispensed to a subject)
- Use of “prohibited” concomitant treatments (e.g., AChEI or memantine)
- Non-compliance regarding study drug intake or endpoints assessment
- Baseline or Week 52 visit are not done in-clinic, but remotely
- Out of window assessments

TauRx / Cytel Confidential: This is a controlled document and shall not be changed, saved, copied or reproduced by any unauthorized person.

4. STATISTICAL METHODS

4.1. General Methods

All outputs will be incorporated into Rich Text Format (RTF) files, sorted, and labeled according to the International Council for Harmonisation of Technical Requirements for Pharmaceuticals for Human Use (ICH) recommendations, and formatted to the appropriate page size(s).

Tabulations will be produced for appropriate demographic, Screening, Baseline, efficacy, and safety parameters. For categorical variables, summary tabulations of the number and percentage within each category (with a category for missing data) of the parameter will be presented. For continuous variables, the mean, median, lower quartile (Q1), upper quartile (Q3), standard deviation, minimum and maximum values will be presented. Time-to-event data will be summarized using Kaplan-Meier Methodology using 25th, 50th (median), and 75th percentiles (if available) with associated two-sided 95% confidence intervals (Hall-Wellner Bands), as well as percent of censored observations.

Formal statistical hypothesis testing will be performed on the primary and secondary efficacy endpoints with all tests conducted at the two-sided, 0.05 level of significance.

4.2. Data Conventions

Mean, median, standard deviation, standard error, Q1 and Q3 will be presented with one more decimal place compared to the raw data, and minimum and maximum will be presented with the same number of decimal places as the raw data. Percentages will be presented with one decimal place.

Wherever a calendar date is presented in a listing, the corresponding Study Day will be included, with Study Day defined as:

- date – first dose date + 1, where date >= first dose date
- date – first dose date, where date < first dose date

For listings including day 1 data with assessments collected prior to and after first study drug, a flag will be included for pre-dose assessments.

The following conversion factors will be used to convert days to months or years where applicable:

- 1 month = 30.4375 days
- 1 year = 365.25 days
- 1 week = 7 days

Additional data handling rules are as follows:

TauRx / Cytel Confidential: This is a controlled document and shall not be changed, saved, copied or reproduced by any unauthorized person.

- Age (years) = year of informed consent – year of birth
- Weight values recorded in pounds will be converted to kilograms using the following formula: kilograms = pounds/2.2046
- Height values recorded in inches will be converted to centimeters using the following formula: centimeters = inches*2.54
- Temperature recorded in Fahrenheit to Celsius: 1 °C = (°F – 32)/1.8
- Duration on study (weeks) = (Last visit date – randomization date + 1) / 7
- Duration on treatment (weeks) = (Last dose date – first dose date + 1) / 7
- (Absolute) Change from Baseline = Value at the time point – Baseline value
- Relative Change from Baseline = (Value at the time point – Baseline value) / Baseline value * 100

To calculate descriptive / inferential statistics for laboratory values containing values below/above the limit of quantification, the following general rule of thumb will be applied: each laboratory value below/above the limit of quantification will be imputed numerically to the nearest value below/above the limit, respecting the same number of decimal places than numerical values (e.g., if the number of decimal places=0, subtract/add 1 to the limit of quantification (LOQ); if the number of decimal places=1, subtract/add 0.1 to the LOQ, etc.). Is the LOQ already the lowest value larger than zero, the LOQ will be imputed numerically to the nearest value below the limit with one more decimal place (e.g., if the LOQ is 1, subtract 0.1 from the LOQ: 0.9). The subject data listings will not show the imputed value, but that the value is below or above the limit of quantification.

Partial dates will be reviewed and imputed (where possible) after the BDRM and before unblinding, potentially resulting in an Excel file, which will be provided to Cytel for the use in the analysis. Remaining cases (if existing), which are required for calculation, or cases for which no additional information is provided after the BDRM or if no file has been generated will be handled as follows:

<u>Start dates</u>

- For missing start day only: Day will be imputed as the first day of the month (i.e., 1) with the following exception: if the partial date falls in the same month and year as the first date of dosing, then the partial date will be imputed to equal the first date of dosing (unless the known end date lies before the first date of dosing, when imputing the incomplete start date).
- For missing start day and month: Day and month will be imputed as the first day of the year (i.e., 1 January) with the following exception: if the partial date falls in the same year as the first date of dosing, then the partial date will be imputed to equal the first date of dosing (unless the known end date lies before the first date of dosing, when imputing the incomplete start date).

<u>Stop dates</u>

<u>TauRx / Cytel</u> Confidential: This is a controlled document and shall not be changed, saved, copied or reproduced by any unauthorized person.

- For missing stop day only: Day will be imputed as the last day of the month (i.e., 28, 29, 30, or 31) or the last day of study contact if earlier.
- For missing stop day and month: Day and month will be imputed as the last day of the year (i.e., 31 December) or the last day of study contact if earlier.

4.3. Computing Environment

Statistical analyses will be performed using SAS® statistical software (Version 9.4 or higher), unless otherwise noted. Medical History and AEs will be coded using Medical Dictionary for Regulatory Activities (MedDRA) version 20.1. Recently used medications and concomitant medications will be coded using the World Health Organization (WHO) Drug version from 01MAR2017.

In addition, main occupation during working life is to be provided, which will be coded using the Standard Occupational Classification, 2010, Volume 2 The Coding Index, UK Office for National Statistics.

More coding details are described in the Data Management Plan.

4.4. Withdrawals, Dropouts, Loss to Follow-up

Subjects who withdraw from the study were not to be replaced.

For subjects who cease taking study drug but who wish to continue in the study, the planned schedule of assessments should be followed, except for the collection of blood samples for MT concentrations (with protocol version 7.0 and 7.0 UK). These subjects will be classified as “the off-treatment-on-study” (TOTOS) group. A subgroup of TOTOS are the subjects with antidementia therapy initiation (AchEI and/or memantine) after informed consent.

Interruption of dosing for up to a maximum of 14 consecutive days may be allowed if the Investigator determines this is indicated (e.g., due to an AE or any other reported change in the subject’s physical condition in the judgment of the Investigator) on a maximum of two occasions. If this is exceeded, study drug would need to be discontinued; however, the subject will be encouraged to continue study participation off-treatment (TOTOS). If subjects stopped the dosing without the Investigator’s approval, they could get back on treatment.

For each subject who withdraws from the study, the decision will be made as part of the BDRM if the withdrawal is potentially treatment-related. For potentially treatment-related dropouts, the fraction of subjects who withdrew at a given time point will be calculated for each treatment group. In addition, a table and figure of the Kaplan-Meier estimator of time to potential treatment-related withdrawal will be given.

<u>TauRx / Cytel</u> Confidential: This is a controlled document and shall not be changed, saved, copied or reproduced by any unauthorized person.

A separate listing of the subjects with antidementia therapy initiation will be provided, including the reason if available. Furthermore, listings will be provided for the efficacy endpoints showing all assessments and assessments which are impacted by antidementia therapy initiation being flagged.

4.5. Missing, Unused, and Spurious Data

In the estimand approach multiple imputation will be applied in hypothetical strategies (see Section 5.9.1). In the MITT analysis multiple imputed data are included as a sensitivity analysis.

4.5.1. Upscaling

Upscaling will be applied in the presence of missing and/or non-valid data, as long as there are sufficient data available.

According to Section 2.5 efficacy scales may have been performed remotely. Based on the document *TRx-237-039: Impact of Remote Administration of Efficacy Scales* the following items are not possible to assess remotely:

- ADAS-cog$_{13}$: All but Orientation. All others, except Ideational Praxis, Number Cancellation, and Commands (sub-subdomains 3 and 4), are possible, when assessment is done by video call. For ADAS-cog$_{11}$ without Ideational Praxis and Commands the maximal achievable total score is 60.
- MMSE: Naming, Comprehension, Reading, Writing, Drawing (may possible, when assessment is done by video call).

For ADSC-ADL$_{23}$ the following items are affected by COVID-19 restrictions:

- Travel, Shopping, Keeping appointments, Left alone, Talk about current events, and Pastime (hobby or game). The maximal achievable total score of the remaining items is 58.

When these items are excluded in the COVID-19 sensitivity analysis, the remaining items will be upscaled as described.

For the end of treatment value (see Section 5.9.1.3), the last available upscaled on-treatment value will be used for analysis.

4.5.1.1. ADAS-cog$_{11}$ and ADAS-cog$_{13}$

The ADAS-cog$_{11}$ / ADAS-cog$_{13}$ total score is the sum of four / five domain scores (see Appendix 12.2.1).

The Word Recall subscore will be calculated as the mean of the non-missing scores from the three trials, rounded to 2 decimal places. The other subscores are obtained directly from the CRF page.

Within a given domain, there may be some missing subscores. If the sum of the maximum possible score of the non-missing items m is greater or equal to one half (50%) of the maximum possible score

TauRx / Cytel Confidential: This is a controlled document and shall not be changed, saved, copied or reproduced by any unauthorized person.

for the domain *t*, the domain score will be scaled up using the formula: *t*s/m*, with *s* being the score of the non-missing subscores.

Otherwise, the domain score will be missing. If any domain score is missing, the ADAS-cog_{11} / ADAS-cog_{13} total score will be missing.

4.5.1.2. ADCS-ADL_{23}

For ADCS-ADL_{23}, it is possible to answer items with "Don't Know". If not more than four items are answered that way, the items are not missing and will count as 0. Upscaling of the other missing values is then done as follows:

Let *m* be the maximal obtainable score of the non-missing items (including "Don't Know"), and *s* the score of the non-missing items (including "Don't Know"). If m/78 is < 2/3, then the total score is set to missing, otherwise upscaling will be performed using the formula: *78*s/m*.

In case of more than four items answered with "Don't Know", the Total score is not provided by the data vendor. Therefore, a more complex upscaling algorithm will be applied to calculate the Total score. Since it cannot be decided, which four "Don't Know" items will count as 0 and which not, the mean of the maximal obtainable scores of the items answered with "Don't Know" is used in the following upscaling rule:

Let *x* be the mean of the maximal obtainable scores of the items answered with "Don't Know", m_2 the maximal obtainable score of the non-missing items (excluding "Don't Know") plus *4*x*, and *s* the score of the non-missing items (excluding "Don't Know") (plus *4*0*).

If $m_2/78$ is < 2/3, then the total score is set to missing, otherwise upscaling will be performed using the formula: $78*s/m_2$.

4.5.1.3. MMSE, CDR-SOB

The following rule is true for MMSE, and CDR-SOB.

Let *t* be the maximal score (MMSE: 30, CDR-SOB: 18), *m* the maximal obtainable score of the non-missing items, and *s* the score of the non-missing items. If m/t is < 2/3, then the total score is set to missing, otherwise upscaling will be performed using the formula: *t*s/m*.

4.5.1.4. Composite Scale and COVID-19 Composite Scale

For the two composite scales, upscaling will be performed analogously to the algorithm as described above (see Sections 4.5.1.1 and 4.5.1.2), restricted to the respective composite scale subscores/ items. If one of the two scales is missing, the respective composite scale missing.

4.5.2. Multiple Imputation

Upscaling will be performed prior to the use of multiple imputation.

TauRx / Cytel Confidential: This is a controlled document and shall not be changed, saved, copied or reproduced by any unauthorized person.

Multiple imputation will be performed only for the double-blind treatment phase data of the two co-primary endpoints and the secondary endpoint MRI whole brain volume.

Missing values will be imputed multiple times to account for the uncertainty about the true values to impute. A multivariate imputation by fully conditional specification (FCS) methods will be used, which handles arbitrary missing patterns. This will be applied using the FCS statement in the MI procedure in SAS®.

The imputation model uses the same fixed factors and covariates as in the respective analysis models, unless specified otherwise.

The seed of **237039** was pre-specified in the study protocol and must be used in the final run. A total of 50 imputed datasets will be generated.

For the MITT populations data after multiple imputation are analyzed in a sensitivity analysis. The imputation model will not include the treatment indicator.

Sample SAS code (will be fully validated at the analysis stage):

```
PROC MI DATA=dataset SEED=237039 NIMPUTE=50 OUT=datami;
CLASS visit severity region prioruse;
FCS REG(adascog11 = visit severity region prioruse adascog11bl);
FCS REG(adcsadl23 = visit severity region prioruse adcsadl23bl);
FCS REG(mriwbv = visit severity region prioruse mriwbvbl);
VAR visit severity region prioruse adascog11bl adcsadl23bl mriwbvbl adascog11 adcsadl23
mriwbv;
RUN;
```

Imputed values will not be edited to fit their respective definition area, since they are not equivalent to observed data and serve only to help estimating covariances between variables.

In sensitivity analyses of the primary estimand (see Section 5.9.1.1) the following two strategies will be followed, as summarized in Section 4.5.2.1 and 4.5.2.2:

4.5.2.1. Imputing missing data for both treatment groups based on data seen in control group

Outcomes are imputed for all treatment groups as if they would have continued on the control, which assumes the statistical behavior of control- and HMTM-treated subjects is the statistical behavior of control-treated subjects.

TauRx / Cytel Confidential: This is a controlled document and shall not be changed, saved, copied or reproduced by any unauthorized person.

This will be implemented by the MNAR statement, which imputes missing values by using the pattern-mixture model approach:

Sample SAS code (will be fully validated at the analysis stage):

```
PROC MI DATA=dataset SEED=237039 NIMPUTE=50 OUT=datami;
CLASS visit severity region prioruse;
FCS REG(adascog11 = visit severity region prioruse adascog11bl);
FCS REG(adcsadl23 = visit severity region prioruse adcsadl23bl);
FCS REG(mriwbv = visit severity region prioruse mriwbvbl);
MNAR MODEL (adascog11 / MODELOBS=(treatment='control'));
MNAR MODEL (adcsadl23 / MODELOBS=(treatment='control'));
MNAR MODEL (mriwbv / MODELOBS=(treatment='control'));
VAR visit severity region prioruse adascog11bl adcsadl23bl mriwbvbl adascog11 adcsadl23 mriwbv;
RUN;
```

4.5.2.2. Imputing missing data for each treatment group based on data seen in their own group

Outcomes are imputed assuming that subjects would follow their initial treatment (rather than switching to control after discontinuation), conditional on baseline and pre-withdrawal data included in the analysis. Therefore, the treatment arm will be included in the imputation model.

Sample SAS code (will be fully validated at the analysis stage):

```
PROC MI DATA=dataset SEED=237039 NIMPUTE=50 OUT=datami;
CLASS visit severity region prioruse treatment;
FCS REG(adascog11 = visit severity region prioruse adascog11bl treatment);
FCS REG(adcsadl23 = visit severity region prioruse adcsadl23bl treatment);
FCS REG(mriwbv = visit severity region prioruse mriwbvbl treatment);
VAR visit severity region prioruse treatment adascog11bl adcsadl23bl mriwbvbl adascog11 adcsadl23 mriwbv;
RUN;
```


TauRx / Cytel Confidential: This is a controlled document and shall not be changed, saved, copied or reproduced by any unauthorized person.

4.5.2.3. Analysis of multiple imputed data

Each of the 50 imputed datasets will be analyzed as done for the non-multiple imputed data, by adding the BY _IMPUTATION_ statement. Afterwards, these 50 results will be combined using standard Rubin's combination rules. Since the analyses results are assumed to be normally distributed no additional transformation will be necessary.

```
PROC MIANALYZE PARMS (CLASSVAR=FULL) = mxparms;
CLASS treatment visit severity region prioruse;
MODELEFFECTS treatment|visit severity region prioruse scorebl;
BY parameter;
ODS OUTPUT PARAMETERESTIMATES=mimxparms;
RUN;
```

4.6. Control Group Labeling

With protocol version 5 on, drug supplies for the control group included tablets containing a urinary discolorant (MTC), 4 mg, dosed at an average frequency of two tablets per week (see Sections 1.3 and 2.1). This means, that most of the subjects randomized to placebo/control received at least one dose of MTC. The sponsor will provide an Excel file to Cytel, which will list the subjects who received placebo only and subjects who transitioned from placebo to MTC 8 mg/week. All other control group subjects will have received MTC 8 mg/week.

In the analysis, a few tables will present subjects who received placebo only separately from subjects who received at least one dose of MTC, which is then highlighted in the respective section of this SAP. In this case, the following treatment group labels will be used: “Placebo Only” and “MTC 8 mg/week”.

For other analyses in which subjects randomized under a protocol version before version 5 are included (e.g., ITT population, see Section 3.1.1), the label for the control group will be “Placebo and/or MTC 8 mg/week”, and for the analyses in which subjects randomized under a protocol version before version 5 are not included (e.g., ITTv5 population), the label for the control group will be “MTC 8 mg/week”.

In the subject data listings control group subjects will be labeled as “Placebo Only” and “MTC 8 mg/week”, respectively.

4.7. Subject Data Listings

Subject data listings of all documented data of interest will be provided (see efficacy, safety, and exploratory analysis sections).

TauRx / Cytel Confidential: This is a controlled document and shall not be changed, saved, copied or reproduced by any unauthorized person.

For the efficacy endpoints the following listings will be prepared: one showing the total score of the scales (ADAS-cog_{11}, ADAS-cog_{13}, ADCS-ADL_{23}, Composite Scale, and COVID-19 Composite Scale), one showing the MRI parameters and another one showing the ^{18}F-FDG-PET parameters. These listings will be accompanied by:

- the information under which protocol version the subject has been randomized,
- a flag for subjects who are excluded from the respective MITT population,
- a flag for assessments that are not potentially confounded by intercurrent illnesses, COVID-19, and concomitant medications (which includes assessments after initiation of an AchEI and/or memantine) as identified during the BDRM,
- a flag for "off treatment" assessments that are after 14 days after the withdrawal of study treatment, or after 14 days after the beginning of a dose interruption and before the restart of study drug intake,
- a flag if the assessment was done remotely,
- the upscaled value,
- a flag indicating a response, and
- a flag indicating a decline.

In addition, for the scales, the individual items and any computed subdomains/ scores etc. will be listed. If feasible, ADAS-cog_{11} and ADAS-cog_{13} can be listed together. For MMSE and CDR separate listings will be provided, too.

Further subject data listings are described in their respective sections (e.g., subject disposition).

4.8. Summary Statistics

To complete the efficacy analysis, which is described in the following sections, summary statistics will be tabulated by visit and treatment group using observed data (after upscaling is applied); for the absolute values, and the change from Baseline, based on the respective analysis populations. This will be repeated for subjects with treatment initiation of AchEI and/or memantine (post-hoc, if sample size is sufficiently high), and for withdrawals from study treatment but remaining in the study (TOTOS, see Section 4.4), separately, for selected primary and secondary endpoints (ADAS-cog_{11}, ADCS-ADL_{23}, MRI whole brain volume, ^{18}F-FDG-PET temporal lobe). If the number of subjects with treatment initiation of AchEI and/or memantine is less than five, a separate subject data listing is enough. Summary statistics for sensitivity analysis will be done post-hoc, if of interest.

If applicable, the absolute values, change from Baseline, and treatment difference (with its 95% confidence intervals), between the HMTM active group(s) and control, based on the least squares

TauRx / Cytel Confidential: This is a controlled document and shall not be changed, saved, copied or reproduced by any unauthorized person.

means (LSM) from the corresponding MMRM or ANCOVA model, will be presented by visit (e.g., see Section 5.9.2.1).

The number and percentage of affected (missing or likely impacted) assessments by COVID-19 will be summarized by endpoint and visit, and further by treatment group and overall, using the information from the respective eCRF *Covid-19 Impact Assessment*. From this eCRF the reason(s) why the study visit was affected, e.g., *Subject unable to attend clinic visit*, will be analyzed in the same way. The denominator is the number of subjects who were still on study for the respective visit.

TauRx / Cytel Confidential: This is a controlled document and shall not be changed, saved, copied or reproduced by any unauthorized person.

5. STATISTICAL ANALYSIS OF DOUBLE-BLIND TREATMENT PHASE

In the following, *decline* is defined as a change into the direction of greater impairment. An *on-treatment visit*, and their corresponding assessments, is defined as a visit where a subject was on investigational product (within the last 14 days) and not on any AchEI and/or memantine (except short-term use). This means, in case of a longer dose interruption a visit can be off-treatment if the subject was not on-treatment within the last 14 days of the visit.

5.1. Primary Efficacy Analysis

There are two co-primary efficacy endpoints for this trial: Baseline adjusted decline in ADAS-cog_{11} and Baseline adjusted decline in ADCS-ADL_{23} from Baseline at Week 52. Repeated measurements on these endpoints scheduled at Weeks 13, 26, 39, and 52 will be treated using a mixed effects model (see Section 5.9). Suppose $\mu_{ADAS\text{-}cog11,T}$ and $\mu_{ADCS\text{-}ADL23,T}$ are the means of decline at Week 52 for the treated arm and $\mu_{ADAS\text{-}cog11,C}$ and $\mu_{ADCS\text{-}ADL23,C}$ are the corresponding means for the control arm, then the global null and the alternative primary efficacy hypotheses can be written as:

H_0: $\mu_{ADAS\text{-}cog11,T} = \mu_{ADAS\text{-}cog11,C}$ OR $\mu_{ADCS\text{-}ADL23,T} = \mu_{ADCS\text{-}ADL23,C}$; versus

H_1: $\mu_{ADAS\text{-}cog11,T} \neq \mu_{ADAS\text{-}cog11,C}$ AND $\mu_{ADCS\text{-}ADL23,T} \neq \mu_{ADCS\text{-}ADL23,C}$.

Thus, the global null versus alternative is a Union-Intersection Test (UIT) which requires both co-primary endpoints to meet statistical significance at the 5% two-sided level of significance for the global null hypothesis to be rejected.

5.2. Multiple Comparisons/Multiplicity

In the double-blind treatment phase, the primary comparison is between HMTM 16 mg/day and control, based on subjects randomized under study protocol version 5.0 or higher.

Both co-primary endpoints must reach significance based on the use of a two-sided test at the alpha=0.05 level of significance for HMTM 16 mg/day to be designated as superior to control. This is because the global null hypothesis is only rejected if and only if the null hypothesis for each of the two co-primary endpoints are rejected. Furthermore, since the efficacy endpoints are primarily analyzed based on two different analysis populations (ITTv5, and the respective MITTv5), the pre-defined sequence is MITTv5 before ITTv5. ITT and MITT populations will be analyzed in the framework of sensitivity analyses.

Therefore, no multiplicity adjustment is necessary.

TauRx / Cytel Confidential: This is a controlled document and shall not be changed, saved, copied or reproduced by any unauthorized person.

5.3. Sample Size Justification

Sample size estimations to achieve 90% power (two-sided alpha = 0.05) to detect a difference between HMTM 16 mg/day and true placebo have been performed for the two co-primary clinical endpoints. These assume a withdrawal rate of 20% to 25% over 52 weeks. The study sample size – here restricted to subjects randomized under protocol version 5.0 or higher as this is the primary analysis population – of approximately 450 subjects (approximately 200 subjects in each treatment group, with a further 50 subjects for secondary analyses of an HMTM 8 mg/day group) is based on the ADCS-ADL_{23} as a larger sample size is required to achieve the target power.

Based on an estimated decline in ADCS-ADL_{23} over 52 weeks in the control arm of 7.7 units with an estimated SD of 8.5 units, the study will have approximately 93.2% power (two-sided alpha=0.05) to detect a reduction in decline of 3.4 units or more. The 3.4 units are motivated by an estimated treatment effect of 5.0 ± 1.6 (mean ± standard error) units in the pooled studies TRx-237-005/ TRx-237-015.

Based on an estimated decline in ADAS-cog_{11} over 52 weeks based on pooled information from Studies TRx-237-005 / TRx-237-015 in the control arm of 6.5 units with an estimated SD of 5.9 units, 200 subjects per treatment arm provide approximately 96.7% power (two-sided alpha=0.05) to detect a reduction in decline of 2.6 units or more. The 2.6 units represent a conservative value as the estimated treatment effect based on pooled Studies TRx-237-005 / TRx-237-015 is 5.2 ± 1.3 (mean ± standard error) units.

5.4. Protocol Deviations

Protocol deviations from the double-blind treatment phase will be analyzed as described in Section 3.2.

5.5. Visit Windows

Since data might be documented under a wrong label (as a wrong visit) within the EDC system, all data will be (re-)assigned to a visit according to the column *Intervals for analysis* in Table 5.

Furthermore, if a subject has multiple values for a parameter within a visit window, the “worst” value will be used for that visit window summary (see Appendix 12.3), for efficacy endpoints the closest to the scheduled visit will be used (if two have the same distance from the scheduled visit the later one will be used, and if two have the same day, the Sponsor will provide a file, which identifies the value to be used in the analysis). For the double-blind treatment phase, priority at Week 52 is given to assessments that took place before initiation of the open-label treatment in case there are multiple values for them within the visit window.

TauRx / Cytel Confidential: This is a controlled document and shall not be changed, saved, copied or reproduced by any unauthorized person.

As an exception, for the analysis of the double-blind treatment phase, a delayed Week 52 ^{18}F-FDG-PET or MRI assessment will be included in the double-blind treatment phase even if it has been done within 14 days after first dose of the open-label treatment phase drug. In addition, the eye exam is eligible for the double-blind treatment phase, if it was performed until the end of the Week 52 protocol-specified visit window. For all other efficacy endpoints and safety information only assessments before the first dose of the open-label treatment phase or if assessments were done on the same day of the first open-label phase treatment are eligible for analysis of double-blind treatment phase data.

Table 5 Evaluation Intervals for Safety and Efficacy Analyses of the Double-Blind Phase

Evaluation (scheduled day)	Protocol-Specified Interval	Intervals for Analysis		
		Safety assessments	ADAS-cog$_{13}$, ADCS-ADL$_{23}$, MRI	^{18}F-FDG-PET, MMSE, CDR
Baseline (1) [a]	–1 to 1	≤1	≤1	1
Week 4 (29)	26 to 32 [±3]	2 to 67	-	-
Week 13 (92)	78 to 106 [±14]	68 to 137	2 to 137	-
Week 26 (183)	169 to 197 [±14]	138 to 228	138 to 228	-
Week 39 (274)	260 to 288 [±14]	229 to 319	229 to 319	-
Week 52 (365)	351 to 379 [±14]	320 to 379	320 to 456	>1

[a] Day 1 – first in-clinic dose of study drug

5.6. Subject Disposition

Based on the screened set, a subject data listing will give an overview of the following screening and randomization information (one row for each subject):

- Protocol version under which the subject has signed the initial informed consent
- Date of initial informed consent
- Duration of the screening period
- If screening failure. If so, reasons for screening failure
- Date of randomization, strata used for randomization, a flag if the subject was miss-stratified, treatment assignment, and initial screening number (if re-screened)

A separate listing will just include the subjects with an extended screening period due to the COVID-19 pandemic allowed by the Investigator. The identified subjects will be provided to Cytel after the BDRM.

TauRx / Cytel Confidential: This is a controlled document and shall not be changed, saved, copied or reproduced by any unauthorized person.

Subjects who are mis-stratified during the randomization are flagged in the aforementioned listing, but will be also listed separately with the following information: randomized treatment group, strata used for randomization, and correct strata values.

Based on the ITT population, a subject data listing will give an overview of the following disposition information (one row for each subject):

- Protocol version under which the subject has been randomized
- First and last dose dates of study drug intake
- Date of study exit
- A flag for the completion of the study, and a flag for the TOTOS (see Section 4.4)
- All reasons why the subject did not complete the study, and a flag for the primary reason
- For the TOTOS, the reason why the subject discontinued the study drug

Based on the ITT population, a subject by-visit listing, including all planned and unplanned visits, will be created with the following information: randomized treatment group, visit name, and visit date range (including study day). Visit windowing will not be performed for this listing, but visits with a clear wrong label, with a visit date range outside of the associated visit window, will be flagged.

Based on the ITT population, a subject data listing will provide an overview of the double-blind treatment phase populations (see Section 3.1.1) and will list the following events (sorted by subject, and date; one row for each event):

- Affiliation to the study populations (one column per study population)
- Date of initial informed consent
- Date of randomization
- Protocol re-consents, with the protocol version and signing dates (one row for each re-consent)
- Date and primary reason of study discontinuation
- Date of study drug discontinuation, if study drug discontinuation is permanent and if the date is different from the date of study discontinuation
- Discontinuation date of AChEI and/or memantine for purposes of the study (if available)
- Date(s) of COVID-19 infection
- Date(s) of COVID-19 vaccination
- Date of Week 52 visit

TauRx / Cytel Confidential: This is a controlled document and shall not be changed, saved, copied or reproduced by any unauthorized person.

The number of screening failure reasons will be tabulated (by region, and total). For large study sites, separate tables might be provided.

Information on the number of subjects randomized, the number of subjects in each analysis population, the number of subjects completing the study as well as the double-blind treatment phase (subsets on and off treatment), the number of subjects discontinuing the study drug but continuing the study (TOTOS), the primary reason for discontinuation of study drug and study, and all reasons for discontinuation of study drug and study will be tabulated. This will be done by treatment group and overall, for the ITT, E-MITT, E-MITTv5, ITTv5, and PPv5 populations, separately. Furthermore, based on the ITT population, this will be tabulated by region.

If the reason for discontinuation of study drug is missing the primary reason for discontinuation of study will be used instead.

5.7. Demographic and Baseline Characteristics

Data will be provided in subject data listings, based on the ITT population.

Demographic and Baseline characteristics will be summarized for the ITT, E-MITT, E-MITTv5, ITTv5, and PPv5 populations. This will also be done for subgroups (based on the ITTv5 population) by use of AChEI and/or memantine prior to Baseline, for subjects characterized as mild AD or MCI-AD (MMSE: 20-27), for subjects characterized as moderate AD (MMSE: 16-19), by region, and for highly recruiting sites (more than 10% of randomized subjects).

General baseline information to be summarized include age at informed consent, sex, ethnicity (Not Hispanic or Latino, Hispanic or Latino) including only the US sites, race, and geographic region; height, weight, creatinine clearance, smoking history, childbearing status/contraception, age at leaving full-time education, and Fazekas score (periventricular white matter score, deep white matter score, and overall score = maximum of periventricular white matter score and deep white matter score). Regarding the race: If race was answered in the eCRF with “not reported” or with a similar comment in “other”, such as “NA”, “NA in France”, “not allowed by law”, “not applicable”, “not authorized”, or “not permitted by law”, race is set to unknown.

Disease specific Baseline characteristics include the verified AD diagnosis (probable AD dementia, MCI-AD [mild cognitive impairment due to AD], based on the Diagnostic Verification form), time from diagnosis of AD to informed consent (years) and time from presumptive AD diagnosis to informed consent (years), *ApoE* genotype (in the subset who provide consent), MMSE, CDR, and previous use of an AChEI and/or memantine. Additionally, a summary of the amyloid PET will be provided, whether it was previously done or within this study. The Diagnostic Verification form was not available for subjects who have been randomized under protocol version 2.1, since only subjects with mild AD were included (see Section 1.3), therefore these subjects will be allocated to the probable AD dementia subgroup. The AD diagnosis is defined as medical history of cognitive impairment and is identified by the following

TauRx / Cytel Confidential: This is a controlled document and shall not be changed, saved, copied or reproduced by any unauthorized person.

medical history Preferred Terms (PT): Dementia Alzheimer's type, Cognitive disorder, Memory impairment, Transient global amnesia, Amnesia, Amnestic disorder, Dementia, Mixed dementia. The presumptive AD diagnosis is defined as the earliest of the following dates:

- Use of AD medication: ATC code N06DA (e.g., Donepezil, Galantamine, Rivastigmine), and ATC code N06DX (Memantine, and 'other anti-dementia drugs')
- Positive amyloid PET scan
- Verified AD diagnosis

Medical history (coding see Section 4.3) will be summarized in a table presenting the numbers and percentages of subjects with medical history terms in a given MedDRA System Organ Classification (SOC) for the ITT and ITTv5 population.

Analysis of recently used medications and concomitant medications is described in the following Section 5.8.

No formal statistical comparisons of treatment groups for any Baseline characteristics will be performed.

5.8. Prior and Concomitant Medications

Prior used medications (discontinued before the start of study drug) and concomitant medications (on medication at the first dose of study drug or after) will be coded using the 01 March 2017 version of the WHO drug dictionary and the Anatomical Therapeutic Classification (ATC) level 1 term, ATC level 3 term, and PT. If the end-date and/or start-date are missing, the medication will be allocated to the concomitant medication.

If a medication date or time is missing or partially missing and it cannot be determined whether it was taken prior to or concomitantly with treatment, it will be considered a concomitant medication.

The use of prior medications, and concomitant medications will be included in subject data listings for the ITT population. For subjects who previously used an AChEI and/or memantine, the start and stop date of medication, whether the medication was stopped prior to screening or between screening and randomization, and reason for stopping usage will be listed separately; this listing will include a flag for subjects randomized under the UK study protocol version 5.0 or higher, since for these subjects, AChEI and/or memantine should end before the start of screening. A subset listing will also be provided for subjects who were using an antipsychotic treatment (see Section 10.4), which includes the reasons for use as well.

The following summaries/tabulations will be prepared for the ITT and ITTv5 populations.

Tabulations with frequency and percentage, by treatment group and medication, will be prepared separately for all prior, for all concomitantly used drugs (used at the time of first study drug or after),

TauRx / Cytel Confidential: This is a controlled document and shall not be changed, saved, copied or reproduced by any unauthorized person.

and for all concomitantly used drugs that the patient is on at the time of first study drug, where subjects reporting more than one medication are counted only once within each level summation.

Summary tables will be provided, showing the numbers of subjects with initiated treatments of: SSRI/SNRI (see Section 12.4), drugs of serotonergic potential (see latest version of the Serotonergic Drugs List – a Sponsor controlled document, which was distributed to Cytel prior to the database lock of the double-blind treatment phase), and any antipsychotic medications. For antipsychotics initiated on-treatment, the reasons for use will be also summarized.

For subjects who previously used an AChEI and/or memantine, the reasons for stopping such medications, duration of use, and duration of time since stopping prior to randomization will be tabulated.

5.9. Efficacy Evaluation

The primary objective of the final amended version of the study protocol (see Section 1.1) is to evaluate the efficacy of HMTM 16 mg/day compared with control over 52 weeks in patients with AD using the MMRM (including the nominal visits Week 13, Week 26, Week 39 and Week 52), including the randomization stratification variables (see Section 2.6) as fixed factors, the respective efficacy Baseline value as covariate, and the treatment indicator (plus its interaction term with the visit variable). The co-primary endpoints are the difference between HMTM 16 mg/day and control in ADAS-cog_{11} and ADCS-ADL_{23} at Week 52.

The next section will give the definitions of the estimands, which are then used in the following efficacy endpoint analysis sections. As a reminder to Section 5.2 the pre-defined analysis sequence is MITTv5 analysis followed by the estimands analysis based on ITTv5. Despite the definition of this sequence, the MITT based analysis is primary for the FDA while the ITT based analysis is considered primary for the EMA.

Regarding the MITT analyses, in subjects who have initiated treatment with an AChEI and/or memantine, assessments made after initiation of such treatment are not considered valid as they could confound the interpretation of the results and will be excluded. Such subjects and affected assessments will be identified prior to unblinding. Sensitivity analyses will be provided for the primary and selected secondary analyses including all data (MRI whole brain volume, and ^{18}F-FDG-PET temporal lobe).

5.9.1. Estimands

Whenever analyzed, the estimands for the comparison of 8 mg/day versus control are defined analogously.

5.9.1.1. Primary Estimand (ITTv5)

The Primary Estimand is designed to answer the question on the treatment effect of HMTM dose of 16 mg/day as monotherapy versus control (containing a small amount of MTC, spiked) in the targeted

TauRx / Cytel Confidential: This is a controlled document and shall not be changed, saved, copied or reproduced by any unauthorized person.

population of subjects with probable AD and MCI-AD at Week 52, regardless of trial product discontinuation, based on the two co-primary endpoints.

This estimand is constructed in line with ICH E9 (R1) addendum. The five components defining the estimand of interest are:

**A. Treatment:**	52 weeks HMTM dose of 16 mg/day or matching control (containing a small amount of MTC) as monotherapy, regardless of adherence.
**B. Population:**	Subjects with probable AD and MCI-AD as described by the inclusion and exclusion criteria, randomized under protocol version 5.0 or higher.
**C. Patient-level outcomes / variables:**	The co-primary outcomes are the change from Baseline of the ADAS-cog_{11} and ADCS-ADL_{23} at Week 52.
**D. Population-level summary:**	Population weighted least squares means difference.
**E. Intercurrent Events:**	The following ICE have been identified which could prevent measurement of the primary outcome or change the interpretation of the measured primary outcome:<br>1. Subjects withdraw from study before the completion of 52 weeks treatment phase for non-treatment related reason<br>2. Subjects withdraw from study before the completion of 52 weeks treatment phase for treatment related reason<br>3. Initiation of AChEI and/or memantine<br>4. Study treatment discontinuation for any reason other than initiation of AChEI and/or memantine (including discontinuation by the Investigator if he/she judges that treatment is no longer appropriate, if the subject's clinical condition is worsening, or for AE, or due to study drug dose interruption longer / more frequent than specified in ICE #5 below) (TOTOs, see Section 4.4)<br>5. A dose interruption for more than 14 consecutive days or more than two occasions of dose interruptions up to a maximum of 14 consecutive days due to safety concerns and initiated by the PI (see Section 4.4)<br>6. Intercurrent illnesses, or initiation of medical food or medications not allowed by protocol (which will be identified during the BDRM)

TauRx / Cytel Confidential: This is a controlled document and shall not be changed, saved, copied or reproduced by any unauthorized person.

7. Study treatment non-compliance, defined as <80% or >120% taking into consideration any dose interruptions (if this cannot be estimated, it does not result automatically in a non-compliance)

8. Deaths before the completion of 52 weeks treatment phase

9. COVID-19 infection

10. Death due to COVID-19

Events 1 will be handled according to a hypothetical strategy as if patients have stayed on treatment.

In a sensitivity analysis all these subject withdrawals are assumed to be potentially treatment-related, which are then handled using the treatment policy approach from ICE #2.

Events 2 will be handled using treatment policy approach reflecting Copy Incremental from Reference strategy (using data after occurrence of the ICE for the estimation, if available). Subjects who withdraw for treatment-related reasons are assumed to retain 100% of the treatment effect they had attained up to the point of withdrawal but do not continue to benefit from treatment afterwards, in other words, assuming that the clinical course post ICE for either treatment group follows control treatment group. This corresponds to the estimate I = (E0*w0 + E13*w13 + E26*w26 + E39*w39) + E52*(1-w0-w13-w26-w39), where for instance *E13* is the treatment effect at Week 13 and *W13* is defined as the fraction of subjects who withdrew for a potential treatment-related reason, have a non-missing efficacy parameter at Week 13, and do not have any on-treatment assessments at any scheduled visit subsequent to Week 13. The fractions are calculated within the HMTM treatment group. *W0* refers to subjects who had no measurement taken after the baseline measurement and thus *E0* is zero. The estimate *I* will be reported as the intervention effect and will be calculated using a contrast on the LSM (see Section 5.9.2.1).

Two sensitivity analyses will be run using hypothetical strategies (not using data after occurrence of the ICE), one by imputing missing data for both treatment groups based on data seen in control group, and one by imputing missing data for each treatment group based on data seen in their own group (see Section 4.5.2).

In another sensitivity analysis the alternative assumption will be investigated, that the subjects withdrawing from treatment for treatment-related reasons do not retain any treatment effect after Baseline; the intervention effect *I* in this case is I=E*(1-w), where *E* is the treatment effect at Week 52 and *w* is the fraction of

TauRx / Cytel Confidential: This is a controlled document and shall not be changed, saved, copied or reproduced by any unauthorized person.

subjects within the HMTM treatment group who withdraw for a potential treatment-related reason up to Week 52.

Events 3 will be handled according to the original randomized treatment group assuming they did not start AChEI and/or memantine and will be analyzed the same as ICE #2.

Events 4 will be handled according to a treatment policy strategy, using data after occurrence of the ICE. A sensitivity analysis will be carried out using the strategy as described for the ICE #2.

Events 5, 6, and 7 will be handled according to a treatment policy approach, using all data (also after occurrence of respective ICE).

Events 8 will be handled as treatment failure. If the patient dies prior to Week 52, the missing assessments will be imputed by the population average in decline, calculated by visit, within the control arm.

Events 9, and 10 will be handled with a hypothetical strategy assuming COVID-19 disease would not have happen, not using data after occurrence of the ICE.

5.9.1.2. Primary Estimand (ITT)

The Primary Estimand (ITT) is designed to answer the question on the treatment effect of HMTM dose of 16 mg/day as monotherapy versus control (with and/or without MTC) in the targeted population of subjects with probable AD and MCI-AD at Week 52, regardless of trial product discontinuation and enrollment before or after protocol version 5.0.

This estimand is constructed in line with ICH E9 (R1) addendum. The five components defining the estimand of interest are:

**A. Treatment:**	Up to 52 weeks HMTM dose of 16 mg/day as monotherapy or matching control (with and/or without MTC).
**B. Population:**	Subjects with probable AD and MCI-AD as described by the inclusion and exclusion criteria.
**C. Patient-level outcomes / variables:**	The co-primary outcomes are the change from Baseline of the ADAS-cog_{11} and ADCS-ADL_{23} at Week 52.
**D. Population-level summary:**	Population weighted least squares means difference.

TauRx / Cytel Confidential: This is a controlled document and shall not be changed, saved, copied or reproduced by any unauthorized person.

E. Intercurrent Events: Identification and handling of ICE as described for the primary estimand (see Section 5.9.1.1), excluding the sensitivity analyses.

5.9.1.3. Secondary Estimand – While on Treatment Strategy (ITTv5)

The Secondary Estimand is designed to answer the question on the treatment effect of HMTM dose of 16 mg/day as monotherapy versus control (containing a small amount of MTC, spiked) in the targeted population of subjects with probable AD and MCI-AD up to 52 weeks while on treatment, based on the two co-primary endpoints.

This estimand is constructed in line with ICH E9 (R1) addendum. The five components defining the estimand of interest are:

A. Treatment: Up to 52 weeks of HMTM dose of 16 mg/day or matching control (containing a small amount of MTC) as monotherapy.

B. Population: Subjects with probable AD and MCI-AD as described by the inclusion and exclusion criteria, randomized under protocol version 5.0 or higher.

C. Patient-level outcomes / variables: The co-primary outcomes are the change from Baseline of the ADAS-cog_{11} and $ADCS\text{-}ADL_{23}$ at the end of treatment of up to 52 weeks (see Section 4.5.1)

D. Population-level summary: Population weighted least squares means difference.

E. Intercurrent Events: The following ICE have been identified which could prevent measurement of the primary outcome or change the interpretation of the measured primary outcome:

1. A dose interruption for more than 14 consecutive days or more than two occasions of dose interruptions up to a maximum of 14 consecutive days due to safety concerns and initiated by the PI (see Section 4.4)

2. Intercurrent illnesses and initiation of medical food or medications not allowed by protocol, identified during the BDRM

3. Study treatment non-compliance, defined as <80% or >120% taking into consideration any dose interruptions (if this cannot be estimated, it does not automatically result in a non-compliance)

4. Deaths before the completion of 52 weeks treatment phase

TauRx / Cytel Confidential: This is a controlled document and shall not be changed, saved, copied or reproduced by any unauthorized person.

Events 1, 2, and 3 that occur while on treatment will be handled according to a treatment policy strategy, using data after occurrence of the ICE.

Events 4 will be handled as treatment failure. If the patient dies prior to Week 52, the missing assessment at the end of treatment will be imputed by the population average in decline at the end of treatment within the control arm.

5.9.1.4. MRI Estimand (ITTv5)

The MRI Estimand is designed to answer the question on the treatment effect of HMTM dose of 16 mg/day as monotherapy versus control (containing a small amount of MTC) in the targeted population of subjects with probable AD and MCI-AD at Week 52, regardless of trial product discontinuation, based on the MRI whole brain volume.

This estimand is constructed in line with ICH E9 (R1) addendum. The five components defining the estimand of interest are:

**A. Treatment:**	52 weeks HMTM dose of 16 mg/day or matching control (containing a small amount of MTC) as monotherapy, regardless of adherence.
**B. Population:**	Subjects with probable AD and MCI-AD as described by the inclusion and exclusion criteria, randomized under protocol version 5.0 or higher.
**C. Patient-level outcome / variable:**	Annualized rate, defined as the absolute change from Baseline, of whole brain atrophy as measured by MRI and quantified using the Boundary Shift Integral (BSI) at Week 52.
**D. Population-level summary:**	Population weighted least squares means difference.
**E. Intercurrent Events:**	Identification and handling of ICE as described for the primary estimand (see Section 5.9.1.1), including the sensitivity analyses.

The MRI Estimand (ITT) is defined as the MRI Estimand, extending the population to the ITT population as done for the Primary estimand (ITT) (see Section 5.9.1.2).

5.9.1.5. PET Estimand (ITTv5)

The PET Estimand is designed to answer the question on the treatment effect of HMTM dose of 16 mg/day as monotherapy versus control (containing a small amount of MTC) in the targeted population of subjects with *very mild* AD (CDR of 0.5) at Week 52, regardless of trial product discontinuation, based

on the ^{18}F-FDG-PET temporal lobe in SUVR. If data is available from subjects with other than a CDR of 0.5, these will not be included in the analysis.

This estimand is constructed in line with ICH E9 (R1) addendum. The five components defining the estimand of interest are:

**A. Treatment:**	52 weeks HMTM dose of 16 mg/day or matching control (containing a small amount of MTC) as monotherapy, regardless of adherence.
**B. Population:**	Subjects with very mild AD (CDR of 0.5) and as further described by the inclusion and exclusion criteria, randomized under protocol version 5.0 or higher.
**C. Patient-level outcome / variable:**	Change from Baseline of the ^{18}F-FDG-PET temporal lobe in SUVR at Week 52.
**D. Population-level summary:**	Population weighted least squares means difference.
**E. Intercurrent Events:**	Identification of ICE is as described for the primary estimand (see Section 5.9.1.1).<br><br>**Events 1** will be handled according to a hypothetical strategy as if patients have stayed on treatment.<br><br>In a sensitivity analysis all these subject withdrawals are assumed to be potentially treatment-related, which are then handled using the treatment policy approach from ICE #2.<br><br>**Events 2** will be handled using a treatment policy approach assuming that the subjects withdrawing from treatment for treatment-related reasons do not retain any treatment effect after Baseline; the intervention effect *I* in this case is I=E*(1-w), where *E* is the treatment effect at Week 52 and *w* is the fraction of subjects within the HMTM treatment group who withdraw for potentially treatment-related reasons up to Week 52.<br><br>**Events 3** will be handled according to the original randomized treatment group assuming they did not start AChEI and/or memantine and will be analyzed the same as ICE #2.<br><br>**Events 4** will be handled according to a treatment policy strategy, using data after occurrence of the ICE. A sensitivity analysis will be carried out using the strategy as described for the ICE #2.

Events 5, 6, and 7 will be handled according to a treatment policy approach, using all data (also after occurrence of respective ICE).

Events 8 will be handled as treatment failure. If the patient dies prior to Week 52, the missing assessment at Week 52 will be imputed by the population average in decline within the control arm.

Events 9, and 10 will be handled with a hypothetical strategy assuming COVID-19 disease would not have happen, not using data after occurrence of the ICE.

The PET Estimand (ITT) is defined as the PET Estimand, extending the population to the ITT population as done for the Primary estimand (ITT) (see Section 5.9.1.2).

5.9.2. **Primary Efficacy Endpoint Analysis**

5.9.2.1. MITT Population Analysis

Data will be analyzed based on the E-MITTv5 population (see Section 3.1.1) using a restricted maximum likelihood-based (REML) MMRM, including a nominal visit variable, the treatment indicator and the interaction term between treatment and visit, the randomization stratification variables (see Section 2.6) as fixed factors, and the respective efficacy Baseline value as covariate; with an unstructured covariance matrix and the Kenward and Roger method of calculating the denominator degrees of freedom for the tests of fixed effects.

Sample SAS code (will be fully validated at the analysis stage):

```
PROC MIXED DATA=dataset METHOD=REML COVTEST;
CLASS subject treatment(REF='Control') visit severity(REF='moderate') region(REF='Europe') prioruse(REF='None');
MODEL scorediff = treatment|visit severity region prioruse scorebl / SOLUTION CL DDFM=KENWARDROGER;
REPEATED visit / SUB=subject TYPE=UN;
LSMEANS treatment*visit visit / CL;
*BY _IMPUTATION_;
LSMESTIMATE treatment*visit "intervention effect at Week 52" 0 0 0 1 0 0 0 -1 / CL;
*Summary statistics for treatment difference based on LSM
LSMESTIMATE treatment*visit "intervention effect at Week 13" 1 0 0 0 -1 0 0 0 / CL;
LSMESTIMATE treatment*visit "intervention effect at Week 26" 0 1 0 0 0 -1 0 0 / CL;
```


TauRx / Cytel Confidential: This is a controlled document and shall not be changed, saved, copied or reproduced by any unauthorized person.

*LSMESTIMATE treatment*visit "intervention effect at Week 39" 0 0 1 0 0 0 -1 0 / CL;*

RUN;

If the mixed model does not converge, the unstructured covariance matrix is replaced by firstly, an auto-regressive covariance structure then by a compound symmetry covariance structure. If the model still does not converge, the randomization stratification variables will be removed, in the following order: region, prior AChEI and/or memantine use, MMSE.

Sensitivity analysis to the statistical model

Model-based sensitivity analysis are:

- Including the interaction term Baseline*visit (*scorebl*visit*) in model statement
- Maximum likelihood (ML) (rather than REML) based repeated measures model (*method=ml*) with polynomial (linear, quadratic, and cubic) time effects and time treated as a continuous variable (number of nominal weeks as well as actual study week defined by study day divided by 7). The estimated annualized change in mean values and standard errors from Baseline to Week 52 will be presented for each treatment group. For sample SAS code see Section 5.9.3.1.

Supplementary and other sensitivity analyses

Supplementary analyses are (starting population: E-MITTv5):

- Including subjects randomized before study protocol version 5.0 (E-MITT population)
- Restricted to the PP population
- Restricted to the completers (subjects who did not stop the study or the study medication early, during the double-blind treatment phase)
- Include assessments after initiation of an AChEI and/or memantine, if more than 5% of the randomized subjects initiated AChEI and/or memantine during the double-blind treatment phase
- Restricted to all visits not potentially confounded by intercurrent illnesses, COVID-19, and concomitant medications (which includes assessments after initiation of an AChEI and/or memantine) as identified during the BDRM
- Stratified by subgroups. If the subgroup consists of less than 10% of the randomized subjects, only summary statistics will be presented.
    - AChEI/memantine use (prior use, never used) [binary]
    - Verified AD diagnosis (probable AD, MCI-AD) [binary]
    - MMSE (16-19, 20-25, and 26-27), in case of mis-stratification the actual value is used

TauRx / Cytel Confidential: This is a controlled document and shall not be changed, saved, copied or reproduced by any unauthorized person.

- MMSE (16-21, 22-27)
- CDR (0.5, 1 or 2) [binary], in case of mis-stratification the actual value is used
- Age group (<75 years, ≥75 years) [binary]
- Sex (male, female) [binary]
- Race (White, non-White) [binary], Race = White (if checked as only race), otherwise Race = non-White (even if multiple races, including White, were checked)
- Geographic region (North America, Europe) [binary]

- Including the *ApoE* genotype as fixed factor (Presence of the ε4 Allele, Absence of the ε4 Allele) [binary]
- Both HMTM treatment groups pooled versus control

Sensitivity analyses are:

- If more than 5% of the randomized subjects have been mis-stratified, then the primary analyses will be repeated using the actual status at randomization
- Multiple imputation analysis (see Section 4.5.2)

For the two co-primary endpoints and their change from Baseline to Week 52, the empirical distribution functions for HMTM 16 mg/day and control will be compared graphically and using a Kolmogorov-Smirnov test. If the Week 52 visit value is not available, the last available on-treatment value will be used.

Sample SAS code (will be fully validated at the analysis stage):

```
PROC NPAR1WAY DATA=dataset EDF PLOTS=EDFPLOT;
CLASS treatment;
VAR scorediff;
EXACT KS;
RUN;
```

For the following efficacy endpoint combinations, the correlation of their changes from Baseline to Week 52 will be analyzed (pooling the data from all three treatment groups):

- ADAS-cog_{11} vs. MRI whole brain volume
- ADCS-ADL_{23} vs. MRI whole brain volume

If for the respective pairwise comparison, the two Week 52 visit values are not available, the last available on-treatment visit with both respective values being available will be used. A scatter plot will compare the values for the two endpoints and will also present the p-value based on the Pearson correlation.

Sample SAS code (will be fully validated at the analysis stage):

```
PROC CORR DATA=dataset;
VAR endpoint1 endpoint2;
RUN;
```

Additional sensitivity / supplementary analyses due to COVID-19 for the two co-primary endpoints

Considerations made by the European Medicines Agency (EMA) and the Food and Drug Administration (FDA) (see Appendix, 8.1) guide possible changes to the statistical analysis due to COVID-19.

The following sensitivity and supplementary analysis will be performed for the two co-primary endpoints and based on the E-MITTv5 population (see Section 3.1.1) to investigate the impact of COVID-19:

- Subgroup analysis by the way of endpoint ascertainment (in-clinic, remote). A subject is assigned to the subgroup with the most visits done with respect to in-clinic or by remote (excluding Baseline and Week 52, which must be performed in-clinic). If a visit was done in-clinic or by remote is documented in the eCRF *Covid-19 Impact Assessment*.
- ADAS-Cog$_{11}$: Include only the subset of items (for all visits), which can be administered by remote by telephone or video call (see Section 4.5.1).
- ADCS-ADL$_{23}$: Exclude items which are affected by COVID-19 restrictions (see Section 4.5.1).
- A composite scale designed as a COVID-19 impact free joint score ("COVID-19 Composite Scale") will be analyzed. The included items, selected from ADAS-Cog$_{11}$ and ADCS-ADL$_{23}$, are indicated in section 12.2 and result in a score ranging from 0 to 113. Since a higher score for ADAS-cog$_{13}$ indicates more impairment (less impairment for ADCS-ADL$_{23}$), the values of ADAS-cog$_{13}$ will be transformed (maximal score minus actual score) before summarizing over both assessments.
- Subgroup of subjects with / without COVID-19 infection.
- Subgroup of subjects with / without COVID-19 vaccination.

If a subgroup consists of less than 10% of the randomized subjects, only summary statistics will be presented.

TauRx / Cytel Confidential: This is a controlled document and shall not be changed, saved, copied or reproduced by any unauthorized person.

5.9.2.2. Estimand Analysis

The primary and secondary estimands (see Section 5.9.1.1, and 5.9.1.3) will be analyzed with the ITTv5 population (see Section 3.1.1) using the MMRM as described in the previous section for the MITT population.

With the estimand described in Section 5.9.1.2 the analysis of the primary estimand will be repeated for the ITT population, but without its sensitivity analyses.

Sample SAS code (will be fully validated at the analysis stage):

PROC MIXED DATA=dataset METHOD=REML COVTEST;

CLASS subject treatment(REF='Control') visit severity(REF='moderate') region(REF='Europe') prioruse(REF='None');

MODEL scorediff = treatment|visit severity region prioruse scorebl / SOLUTION CL DDFM= KENWARDROGER;

REPEATED visit / SUB=subject TYPE=UN;

*LSMEANS treatment*visit visit / CL;*

**BY _IMPUTATION_;*

*LSMESTIMATE treatment*visit "intervention effect" nw13 nw26 nw39 nw52 -nw13 -nw26 -nw39 -nw52 / DIVISOR=Nhmtm CL;*

*LSMESTIMATE treatment*visit "intervention effect (sensitivity)" 0 0 0 nw52 0 0 0 -nw52 / DIVISOR=Nhmtm CL;*

RUN;

nw0, nw13, *nw26*, and *nw39* are the number of subjects within the respective HMTM treatment group who withdrew for potentially treatment-related reasons (ICE #2 in Section 5.9.1.1), have a non-missing efficacy parameter at Baseline, Week 13 (26, 39), and do not have any on-treatment assessments at any scheduled visit subsequent to Baseline, Week 13 (26, 39). With *Nhmtm* the sample size in the HMTM treatment group, *nw52* is calculated as *nw52 = Nhmtm – nw0 – nw13 – nw26 – nw39*. The numbers will need to be calculated before applying the analyses model (for example saved in macro variables). With respect to sensitivity analyses, *nwx* and *nw* will need to be re-calculated, when handling further ICEs as ICE #2.

The secondary estimand is analyzed using an ANCOVA, including the randomization stratification variables (see Section 2.6) as fixed factors, the respective efficacy Baseline value and the total duration of exposure (see Section 5.12.1) as covariates, and the treatment indicator; with the Kenward and Roger method used for calculating the denominator degrees of freedom for the tests of fixed effects.

TauRx / Cytel Confidential: This is a controlled document and shall not be changed, saved, copied or reproduced by any unauthorized person.

Sample SAS code (will be fully validated at the analysis stage):

PROC MIXED DATA=dataset METHOD=REML COVTEST;

CLASS subject treatment(REF='Control') severity(REF='moderate') region(REF='Europe') prioruse(REF='None');

MODEL scorediff = treatment severity region prioruse scorebl (duration) / SOLUTION CL DDFM= KENWARDROGER;

LSMEANS treatment / CL;

LSMESTIMATE treatment "intervention effect at the end of treatment" 1 -1 / CL;

RUN;

5.9.3. Secondary Efficacy Endpoint Analysis

All secondary analyses have the underlying null hypothesis that there is no difference in change from Baseline in the quantity of interest between the HMTM 16 mg/day group, or the HMTM 8 mg/day group, and the control group.

The sequence of the secondary efficacy analysis is as follows:

- MRI whole brain: HMTM 16 mg/day versus control
- ^{18}F-FDG-PET temporal lobe (normalized to pons): HMTM 16 mg/day versus control
- ^{18}F-FDG-PET temporal lobe (normalized to pons): HMTM 8 mg/day versus control
- ADAS-cog$_{11}$ and ADCS-ADL$_{23}$: HMTM 8 mg/day versus control
- MRI temporoparietal lobe: HMTM 16 mg/day versus control, and HMTM 8 mg/day versus control

5.9.3.1. MRI: Whole Brain Atrophy

The atrophy, defined as the absolute change from Baseline, is already calculated by the responsible imaging core laboratory (see Section 2.9.1.1). The atrophy will be analyzed using the corresponding MMRM. The estimated annualized rates and the treatment group difference are obtained from the LSM.

MITT population analysis

Data will be analyzed based on the MI-MITTv5 population (see Section 3.1.1) according to Section 5.9.2.1 (including the supplementary and sensitivity analysis).

In a further exploratory analysis, the Baseline values of the MRI parameters putamen, nucleus accumbens, and nucleus basalis will be included as covariates.

TauRx / Cytel Confidential: This is a controlled document and shall not be changed, saved, copied or reproduced by any unauthorized person.

Sample SAS code with time being continuous (will be fully validated at the analysis stage):

PROC MIXED DATA=dataset METHOD=REML COVTEST;

CLASS subject treatment(REF='Control') severity(REF='moderate') region(REF='Europe') prioruse(REF='None');

MODEL atrophy = treatment|time severity region prioruse scorebl / SOLUTION CL DDFM=KENWARDROGER;

REPEATED / SUB=subject TYPE=UN;

LSMEANS treatment / CL;

*ESTIMATE "HMTM at Week 52" intercept 1 treatment 1 0 time 52 treatment*time 52 0 / CL;*

*ESTIMATE "Control at Week 52" intercept 1 treatment 0 1 time 52 treatment*time 0 52 / CL;*

*ESTIMATE "intervention effect at Week 52" treatment 1 -1 treatment*time 52 -52 / CL;*

**Polynomial time effects;*

**Quadratic;*

*ESTIMATE "HMTM at Week 52 (2)" intercept 1 treatment 1 0 time 52 treatment*time 52 0 time*time 2704 treatment*time*time 2704 0 / CL;*

*ESTIMATE "Control at Week 52 (2)" intercept 1 treatment 0 1 time 52 treatment*time 0 52 time*time 2704 treatment*time*time 0 2704 / CL;*

*ESTIMATE "intervention effect at Week 52 (2)" treatment 1 -1 treatment*time 52 -52 treatment*time*time 2704 -2704 / CL;*

**Cubic;*

*ESTIMATE "HMTM at Week 52 (3)" intercept 1 treatment 1 0 time 52 treatment*time 52 0 time*time 2704 treatment*time*time 2704 0 treatment*time*time*time 140608 0 / CL;*

*ESTIMATE "Control at Week 52 (3)" intercept 1 treatment 0 1 time 52 treatment*time 0 52 time*time 2704 treatment*time*time 0 2704 treatment*time*time*time 0 140608 / CL;*

*ESTIMATE "intervention effect at Week 52 (3)" treatment 1 -1 treatment*time 52 -52 treatment*time*time 2704 -2704 treatment*time*time*time 140608 -140608 / CL;*

RUN;

Estimand analysis

The MRI estimand (see Section 5.9.1.4) will be estimated analogously to the primary estimand (see Sections 5.9.1.1 and 5.9.2.2).

5.9.3.2. ^{18}F-FDG-PET: Temporal Lobe

If data is available from subjects with other than a CDR of 0.5, these will not be included in the analysis.

MITT population analysis

Data will be analyzed based on the PI-MITTv5 population (see Section 3.1.1), using an ANCOVA, including the randomization stratification variables (see Section 2.6) as fixed factors, the respective efficacy Baseline value as covariate, and the treatment indicator; with the Kenward and Roger method used for calculating the denominator degrees of freedom for the tests of fixed effects.

Sample SAS code (will be fully validated at the analysis stage):

PROC MIXED DATA=dataset METHOD=REML COVTEST;

CLASS subject treatment(REF='Control') severity(REF='moderate') region(REF='Europe') prioruse(REF='None');

MODEL scorediff = treatment severity region prioruse scorebl / SOLUTION CL DDFM=KENWARDROGER;

LSMEANS treatment / CL;

LSMESTIMATE treatment "intervention effect" 1 -1 / CL;

RUN;

Supplementary and sensitivity analysis will be performed as described in Section 5.9.2.1, except the model-based sensitivity analysis involving the time.

Estimand analysis

The PET estimand (see Section 5.9.1.5) will be estimated using the ANCOVA as described previously for the MITT population.

Sample SAS code (will be fully validated at the analysis stage):

PROC MIXED DATA=dataset METHOD=REML COVTEST;

CLASS subject treatment(REF='Control') severity(REF='moderate') region(REF='Europe') prioruse(REF='None');

MODEL scorediff = treatment severity region prioruse scorebl / SOLUTION CL DDFM=KENWARDROGER;

LSMEANS treatment / CL;

LSMESTIMATE treatment "intervention effect" nw52 -nw52 / DIVISOR=Nhmtm CL;

RUN;

TauRx / Cytel Confidential: This is a controlled document and shall not be changed, saved, copied or reproduced by any unauthorized person.

With *nw* the total number of subjects who withdrew for potentially treatment-related reasons during the study until Week 52 in the respective HMTM group and *Nhmtm* the total sample size in the HMTM group, then *nw52* = *Nhmtm* - *nw*. The numbers will need to be calculated before applying the analyses model (for example saved in macro variables).

5.9.3.3. MRI: Temporoparietal Lobe Atrophy

For this secondary variable no further sensitivity / supplementary analyses are pre-specified. If necessary, post-hoc analysis will be performed to get a more in-depth understanding of the data.

MITT population analysis

Data will be analyzed based on the MI-MITTv5 population, using the MMRM as described in the sections 5.9.2.1 and 5.9.3.1.

In an exploratory analysis the Baseline values of the MRI parameters putamen, nucleus accumbens, and nucleus basalis will be included as covariates.

Estimand analysis

Data will be analyzed according to Section 5.9.2.1 using the estimand as described in Section 5.9.1.4 adapted to the temporoparietal lobe, based on the ITTv5 population.

5.9.3.4. HMTM 8 mg/day versus control

As a general remark, for the 8 mg/day versus control comparison, the order of analysis is ^{18}F-FDG-PET temporal lobe, MRI temporoparietal lobe, ADAS-cog_{11}, ADCS-ADL_{23}.

MITT population analysis

The analysis of ADAS-cog_{11} and ADCS-ADL_{23} will be performed as outlined in Section 5.9.2, restricted to the primary analysis models and analysis populations ITTv5 and E-MITTv5, respectively. Further post-hoc analysis may follow.

In a sensitivity analysis for E-MITTv5, all three treatment groups are included in the PROC Mixed call using appropriate contrast statements for pairwise treatment comparisons against control.

The analysis of the ^{18}F-FDG-PET temporal Lobe will be done as described in Section 5.9.3.2 without the sensitivity / supplementary analysis.

The analysis of the MRI temporoparietal lobe atrophy will be done as described in Section 5.9.3.3 without the MITT explorative analysis.

Estimand analysis

The analysis of ADAS-cog_{11} and ADCS-ADL_{23} will be performed as outlined in Section 5.9.2, restricted to the primary estimand, without further sensitivity analysis. Further post-hoc analysis may follow.

TauRx / Cytel Confidential: This is a controlled document and shall not be changed, saved, copied or reproduced by any unauthorized person.

The analysis of the ^{18}F-FDG-PET temporal lobe will be done as described in Section 5.9.3.2 without the sensitivity / supplementary analysis.

The analysis of the MRI temporoparietal lobe atrophy will be done as described in Section 5.9.3.3 without the MITT explorative analysis.

5.9.4. Responder Analysis (Sensitivity)

This analysis is based on the ITTv5 population and is done separately for each of the five primary / secondary efficacy variables (ADAS-cog_{11}, ADCS-ADL_{23}, MRI whole brain, MRI temporoparietal lobe, and ^{18}F-FDG-PET temporal lobe) and the exploratory efficacy variable Composite Scale, and furthermore separately for each HMTM group comparison versus control.

A responder will be defined as a subject whose change from Baseline to Week 52 is less or equal to a threshold *T* (if a larger value means greater impairment, otherwise: *more* or equal), which will be calculated based on the least squares means from the primary analysis model (in Section 5.9.2.1, including all three treatment groups together) and the respective MITTv5 population (E-MITTv5, MI-MITTv5, PI-MITTv5).

T is defined as follows, with LSM_C and LSM_T the LSM change from Baseline at Week 52 in the control arm and (HMTM) treated arm, respectively: $T = (LSM_C + LSM_T)/2$.

Subjects who do not have a final assessment at Week 52 will be classified as non-responders. Subjects with no Baseline assessment will not be included in the analysis and counted as missing.

According to the study protocol, the proportion of responders will be analyzed using the Cochran-Mantel-Haenszel (CMH) test, adjusting for the randomization strata variables separately (severity, prior use of standard AD treatment, and region). In addition, the CMH test will be performed adjusting for the prior use of standard AD treatment stopped before Screening. Odds ratios and 95% confidence intervals will be presented.

Sample SAS code (will be fully validated at the analysis stage):

```
PROC LOGISTIC DATA=dataset;
CLASS treatment(REF='Control');
MODEL response=treatment;
STRATA (rand)stratum;
EXACT treatment;
RUN;
```

As a sensitivity analysis a logistic regression will be applied to include the randomization strata variables simultaneously (without prior use of standard AD treatment stopped before Screening):

```
PROC LOGISTIC DATA=dataset;
CLASS treatment(REF='Control') severity(REF='moderate') region(REF='Europe') prioruse(REF='None');
MODEL response=treatment severity region prioruse;
RUN;
```

For statistically significant treatment effects (p-value < 0.05) on the aforementioned five efficacy variables, the association between responders across these variables at Week 52 will be assessed by using Pearson's chi-square test to analyze the resulting 2 × 2 table. The number and percent of subjects in each cell of the 2 × 2 table will be tabulated along with the p-value from the Pearson's chi-square test.

Sample SAS code (will be fully validated at the analysis stage):

```
PROC FREQ DATA=dataset;
TABLES response1*response2 / CHISQ;
RUN;
```

5.9.5. Time-to-event/Decline Analysis (Sensitivity)

This analysis, based on the ITTv5 population, will include off-treatment measurements (TOTOS) and will be done separately for the primary and secondary efficacy variables ADAS-cog_{11}, ADCS-ADL_{23}, and MRI whole brain volume. It will only compare subjects on HMTM 16 mg/day and control.

Time-to-event/decline will be calculated as date of onset of decline – date of first dose + 1 and will use only data from the double-blind treatment phase and upscaled values for the two co-primary endpoints.

Time of onset of decline is the first of two consecutive measurements with worsening, whereas worsening is defined as an impairment, with respect to the Baseline value of at least a change in units as outlined here:

- ADAS-cog_{11}, ADCS-ADL_{23}: 1
- MRI whole brain volume: 6,500 (mm^3 = µL)

If the threshold of the MRI whole brain volume does not appear to be reasonable once the unblinded randomization codes are used, these can be modified slightly (similarly if a model may not converge).

If the available Week 52 value (or the value of the last assessment in general, in case of early termination) is worsened without a prior worsening, an onset of decline will be triggered at Week 52 (or the respective last visit) for that subject. Subjects without any decline will be censored at the date

of last assessment, but if a subject missed the scheduled visit, the subject is censored at the target day of the scheduled visit.

Any missing efficacy/imaging value for a scheduled visit will be treated as a worsening from baseline, for that visit, but a single missing value at the last planned visit without a missing value or without a worsening in the previous visit will not count as a decline. If the time of onset is missing due to a missed scheduled visit the scheduled date of the missing visit will be used.

The Cox proportional hazards model with effects for treatment group and the randomization stratification variables will be used to compare HMTM 16 mg/day versus control by way of Hazard Ratio, 95% confidence interval, and p-value. A graph and a table of the Kaplan-Meier estimates will be provided.

Sample SAS code (will be fully validated at the analysis stage):

```
PROC PHREG DATA=dataset PLOTS(OVERLAY)=SURVIVAL;
CLASS subject treatment(REF='Control') severity(REF='moderate') region(REF='Europe') prioruse(REF='None');
MODEL time*status(0) = treatment severity region prioruse;
ID subject;
RUN;
```

One version will be based on the visit windows (nominal data), and another will use actual dates (continuous data).

5.10. Exploratory Analyses

If not indicated otherwise the analyses will be performed based on the respective MITTv5 population comparing HMTM 16 mg/day with control as described in Section 5.9.2.1, without any further sensitivity and supplementary analysis.

Descriptive analysis calculated by treatment group and visit based on the observed data will be given.

These analyses are broadly classified into two groups, analyses of clinical endpoints and imaging endpoints. Of particular interest are to test the hypotheses that HMTM 16mg/day leads to a reduction in MRI total lesion volume over time, that HMTM 16 mg/day increases the ^{18}F-FDG-PET SUVR in cerebellum measured with respect to pons, and that HMTM 16 mg/day shows a significant reduction in decline on the composite endpoint in the mild (CDR 0.5) population.

TauRx / Cytel Confidential: This is a controlled document and shall not be changed, saved, copied or reproduced by any unauthorized person.

5.10.1. ADAS-cog$_{13}$

The ADAS-cog$_{13}$ is assessed at Baseline pre-dose, 13, 26, 39 and 52 weeks (or early termination) during the double-blind treatment phase and will be analyzed using the MMRM as described in Section 5.9.2.1.

This analysis will be repeated for the subgroup on subjects with MCI-AD, and mild AD (MMSE 20-27).

5.10.2. Composite Scale

The Composite Scale is assessed at Baseline pre-dose, 13, 26, 39 and 52 weeks (or early termination) during the double-blind treatment phase and will be analyzed using the MMRM as described in Section 5.9.2.1, but also investigating the treatment effect at Week 39.

Additionally, this analysis will be repeated in subjects with CDR of 0.5 at Screening, and CDR of 1-2 at Screening.

5.10.3. MMSE

The MMSE is assessed at Screening and Week 52 (or early termination) during the double-blind treatment phase and will be analyzed using the ANCOVA as described in Section 5.9.3.2 for the ITTv5 population.

5.10.4. CDR-SOB

The CDR-SOB is assessed at Screening and Week 52 (or early termination) during the double-blind treatment phase and will be analyzed using the ANCOVA as described in Section 5.9.3.2 for the ITTv5 population.

5.10.5. MRI Parameters

The atrophy of further brain MRI parameters (putamen, nucleus accumbens, and nucleus basalis) will be analyzed using the MMRM as described in Section 5.9.3.1.

Furthermore, the Baseline values of putamen, nucleus accumbens, and nucleus basalis will be included as covariates in the analysis of secondary endpoints whole brain, and temporoparietal lobe (see Sections 5.9.3.1 and 5.9.3.3).

In a sensitivity analysis of the two co-primary endpoints ADAS-cog$_{11}$ and ADCS-ADL$_{23}$, the Baseline values of MRI whole brain volume and MRI T1 imaging TLV and their interactions with treatment (the additive terms are included as well) will be added to the analysis model (see Section 5.9.2.1).

5.10.6. ^{18}F-FDG-PET Parameters

The ^{18}F-FDG-PET SUVRs in subjects with mild AD (CDR of 0.5 at Screening):

- temporal lobe normalized to cerebellum,
- cerebellum normalized to pons,

TauRx / Cytel Confidential: This is a controlled document and shall not be changed, saved, copied or reproduced by any unauthorized person.

- parietal lobe normalized to pons and normalized to cerebellum, and
- frontal lobe normalized to pons and normalized to cerebellum

will be analyzed using the ANCOVA as described in Section 5.9.3.2.

Other SUVR regions anterior, and posterior cingulate gyrus will be examined descriptively by summarizing the actual value and change from Baseline.

5.10.7. MRI Hypointensities and Hyperintensities

The TLV (hypointensities) based on MRI T1 imaging and the MRI hyperintensities total volume based on MRI FLAIR will be analyzed using the MMRM as described in Section 5.9.3.1.These parameters and the other MRI FLAIR sub-parameters deep volume, infratentorial volume, and periventricular volume will be listed together with the other MRI parameters.

5.10.8. *ApoE* Genotype

A subject data listing will be provided for *ApoE* genotype data, including only the subjects who consented to this determination. In this listing, the genotype classification (presence/absence of the ε4 allele) will be given, but not the detailed genotype.

The influence of the genotype (presence/absence of ε4 allele) will be evaluated for the two co-primary endpoints and selected secondary endpoints (MRI whole brain volume, and ^{18}F-FDG-PET temporal lobe) by adding the genotype classification as a fixed factor into the MMRM and ANCOVA model (see Section 5.9.2.1).

5.11. Statistical Parametric Mapping Analyses (SPM)

This section describes the planned SPM analyses, performed by an external vendor. Changes to these analyses, will be discussed and presented in the CSR or SPM analysis report. Further analyses might be performed dependent on the outcome of these exploratory analyses.

SPM12 is used to process volumetric MRI (vMRI), Arterial Spin Labeling (ASL) MRI and ^{18}F-FDG-PET images for the pre-specified voxel-wise analysis, and to define and estimate General Linear Models (GLM) yielding Statistical Parametric Maps (SPM) of each main effect. Pre-Processing includes format conversion, realignment, summing and co-registration to native MR space, tissue class segmentation, DARTEL registration and population template creation, normalization to standard template space and, smoothing of images.

Processed vMRI, ASL regional cerebral blood flow (rCBF) and ^{18}F-FDG-PET images will be used to fit a GLM for statistical inference at every voxel in the brain. A design matrix for each main effect analysis specifies a statistical model to explain variance in the image space. Statistical models are implemented as two-sample t-tests for group analysis, and linear regression for continuous variables, with control

TauRx / Cytel Confidential: This is a controlled document and shall not be changed, saved, copied or reproduced by any unauthorized person.

and nuisance variables in each case. For each main effect analysis, a design matrix is created to estimate beta weights explaining how each model vector independently influences variance in the processed images, plus some error. A contrast vector specifies the relative contribution of these model vectors, using standard least squares, and is associated with a t-statistic reflecting the combined weights divided by the standard error at each voxel. Multiple comparison's correction employing the familywise error rate (FWE) is used to control for the large number of t-tests being performed. The FWE threshold is set to p<.05.

Specifically, a whole-brain GLM analysis will be performed to derive voxel-wise statistical maps for each of the following main effects:

1. Delta Drug vs Delta control, vMRI
2. Delta Drug vs Delta control, 18F-FDG-PET
3. Delta Drug vs Delta control, ASL MRI

5.11.1. **VBM volumetric MRI analysis**

The voxel based morphometry (VBM) analysis will be performed using the approach described by John Ashburner (www.fil.ion.ucl.ac.uk/~john/misc/VBMclass15.pdf), briefly described here:

Preprocessing

SPM→Spatial→Segment: To generate the roughly (via a rigid-body) aligned grey and white matter images of the subjects.

SPM→Tools→Dartel Tools→Run Dartel: (create Template): Determine the nonlinear deformations for warping all the grey and white matter images so that they match each other.

SPM→Tools→Dartel Tools→Normalise to MNI Space: Actually, generate the smoothed "modulated" warped grey and white matter images.

Statistical comparison

Within the SPM package we will construct a GLM for each voxel to compare the change in grey matter volume in the in the treated group with the change in grey matter volume in the control group. The comparison will be done correcting for the covariate total intracranial volume (TICV) and the randomization stratification variables. The effect of age and sex will also be tested.

Within the SPM package we will construct a GLM for each voxel to compare the change in white matter volume in the in the treated group with the change in white matter volume in the control group. The comparison will be done correcting for the same covariates mentioned above.

5.11.2. **FDG PET/CT analysis**

Preprocessing

TauRx / Cytel Confidential: This is a controlled document and shall not be changed, saved, copied or reproduced by any unauthorized person.

As per the acquisition protocol the 6 by 5 minute reconstructed, attenuation corrected PET images will be realigned and summed to produce a single FDG image for each time point.

The single summed image will then be normalized into MNI space using the SPM provided template.

The normalized summed image will then be scaled using a ponds region of interest so that like for like comparisons can be made.

This step will be repeated using the cerebellum as the normalizing region of interest.

Statistical comparison

Within the SPM package we will construct a GLM for each voxel to compare the change in normalized pons scaled FDG uptake treated group with the change in FDG uptake in the control group. The comparison will be done correcting for randomization stratification variables as covariates, the effect of age and sex will be tested as well.

Within the SPM package we will construct a GLM for each voxel to compare the change in normalized cerebellum scaled FDG uptake treated group with the change in FDG uptake in the control group. The comparison will be done correcting for same covariates as mentioned above.

5.11.3. **ASL MR rCBF analysis**

Preprocessing

The ASL rCBF images will be normalized into Montreal Neurological Institute (MNI) space using the SPM provided template.

Statistical comparison

Within the SPM package we will construct a GLM for each voxel to compare the change in normalized ALS rCBF images treated group with the change ASL rCBF in the control group. The comparison will be done correcting for same covariates as for the PET analysis.

5.12. **Safety Analyses**

Safety analyses will be conducted using the Safety population. Key analyses will be repeated based on the Safetyv5 population. The analyses of the double-blind treatment phase are limited to the events up to the first dose of the open-label treatment phase. The eye exam is assigned to the double-blind treatment phase, if it was performed until the end of the Week 52 protocol-specified visit window.

5.12.1. **Extent of Drug Exposure and Compliance**

For subjects randomized under protocol version 5.0 or above, it was planned that a subject receives four tablets (2 tablets twice) orally per day for 52 weeks:

- 16 mg/day: 4 mg four times per day

TauRx / Cytel Confidential: This is a controlled document and shall not be changed, saved, copied or reproduced by any unauthorized person.

- 8 mg/day: 4 mg twice and control twice per day
- Control: Control four times per day (with one MTC 4 mg tablet interspersed on two occasions within each week on a deterministic but variable schedule allowing one to five days between two doses)

For subjects randomized in version 2.1, it was planned that a subject receives two tablets orally per day for 6 months. If such a subject reconsented to version 3.0 or higher, they subsequently received four tablets orally per day.

First dose dates, dose interruptions, and last dose dates are documented in the eCRFs.

Drug accountability: the record of all study drug dispensed to and returned by subjects (including kit ID and a comment field) is recorded in the RTSM system. The drug accountability information is shared as an SAS file. Things to note are:

- Total Dispensed is auto populated by the system and is dependent on the kit type. Protocol versions v1-v3 have 210 tablets and protocol versions v4+ have 420 tablets.
- Total Used Qty, Total Unused Qty, Total Lost Qty & Total Damaged Qty along with the Accountability Comments are entered by the site. Any queries with the data in these columns should be directed to the person in column Accounted By or Verified By.
- There will be at least two rows per kit when accountability has been verified. Column Action Taken will be populated with an “I” or “U” where “I” is the initial entry and “U” is an updated/verification entry.
- The report only lists kits that have had drug accountability performed in Trident. If the site has not performed drug accountability in Trident, then the kit will not appear in the report.
- Baseline, Week 13, Week 26, and Week 39 visits are performed during double-blind treatment phase where one kit is dispensed at each visit. Week 52 (OL) and Week 78 (OL) are performed during the open-label treatment phase where two kits are dispensed.

The drug accountability sheet therefore has several rows for one dispensed visit/kit. It monitors the initial creation and every update done to it. To analyze the data, the most recent information will be used, which is the last entry, by using the sorting of the raw data file, of each kit.

The following information will be summarized descriptively by treatment group and overall, whereas subjects randomized to placebo/control will be analyzed separately by subjects receiving only true placebo and subjects receiving at least one dose of MTC:

- Total duration of exposure (in weeks): (last dose date – first dose date + 1)/7
- Total duration of treatment (in weeks), which excludes days in which dose was interrupted
- Mean daily dose: Total of administered dose during the duration of exposure, relative to the total duration of exposure

TauRx / Cytel Confidential: This is a controlled document and shall not be changed, saved, copied or reproduced by any unauthorized person.

- Total subject-years of exposure: sum of total exposure in years

$$\frac{1}{365.25}\sum_{subjects}\frac{\#tablets\ taken\ during\ db\ phase}{\#tablets\ planned\ during\ db\ phase} * total\ duration\ of\ exposure\ (days)$$

- Compliance: # Tablets taken / # tablets planned until Week 52 (or until early study drug termination). Including frequency and percentage of subjects with <80% and >120% compliance. A compliance flag will be re-calculated together with the actual compliance figure.
- Frequency and percentage of subjects with dose interruptions
- Frequency and percentage of subjects in the duration of exposure categories (with categories based on the scheduled day for each planned visit, see Table 5: 1 day, 2 to 29 days, 30 to 92 days, etc.), based on total duration of exposure, including interruption days

This summarization will be done for the Safety population, but also repeated restricted to the subjects randomized before protocol version 5.0, and restricted to the subjects randomized under or after protocol version 5.0 (Safetyv5).

Subject data listings will encompass dosing, drug accountability, and compliance. Any doses that are other than that randomized will be flagged and the protocol version under which the subject had been randomized will be given.

5.12.2. **Adverse Events**

Adverse events (AEs) will be coded using the MedDRA version 20.1 and are displayed in tables and listings using SOC and PT.

Treatment emergent AEs (TEAE) are defined as AEs with:

- Onset after first dose of study drug or
- Worsen in intensity after first dose of study drug or
- Worsen in treatment relationship after first dose of study drug

Based on the Safety population, a time-to-event analysis (event: any TEAE) will be performed using the model as described in Section 5.9.5). The time-to-first TEAE will be calculated as date of onset – date of first dose + 1, and subjects with no TEAE will be censored at the time of study discontinuation.

AEs are summarized by subject incidence rates, therefore, in any tabulation, a subject contributes only once to the count for a given AE (SOC or PT, most related occurrence, or most intense occurrence) regardless the number of episodes.

The total number of subjects and the numbers stratified by MedDRA SOC and PT, along with the corresponding percentage, with the following AE will be derived and summarized by treatment group and overall:

TauRx / Cytel Confidential: This is a controlled document and shall not be changed, saved, copied or reproduced by any unauthorized person.

- Any TEAE (separate columns for true placebo and MTC)
- Any TEAE with an onset of Day 1 (separate columns for true placebo and MTC), complemented by a subject data listing
- Any TEAE (categorized by severity, and separate columns for true placebo and MTC), repeated for the Safetyv5 population.
- Any TEAE (categorized by severity) which occurred within two weeks after any COVID-19 vaccination (separate columns for true placebo and MTC)
- Any TEAEs assessed by the Investigator as related to treatment (i.e., related or possibly related) (separate columns for true placebo and MTC)
- Any TEAEs severe in intensity and assessed by the Investigator as related to treatment (as defined above) (separate columns for true placebo and MTC)
- Any TEAE that resulted in interruption or discontinuation of study drug (presented separately and combined) (separate columns for true placebo and MTC)
- Any SAE (separate columns for true placebo and MTC)
- Any serious adverse reaction (SAR), which are the SAEs judged to be possibly related or related to the study drug by the Investigator (separate columns for true placebo and MTC)
- Malignancies other than non-melanoma skin cancers (separate columns for true placebo and MTC)
- COVID-19 infection, a Pearson's chi-square test is used to compare the frequencies pairwise versus the control group
- *TauRx AE Groupings*: Subsets of TEAEs (defined by PT, the latest version of this allocation list, which is to be used in the analysis, was provided to Cytel prior to the database lock of the double-blind treatment phase), summarized per grouping and sub-grouping (separate columns for true placebo and MTC), repeated for the Safetyv5 population
  - Group: Targeted Gastrointestinal Events
    - Sub-group: Diarrhea
    - Sub-group: Gastrointestinal Irritation
    - Sub-group: Nausea/Vomiting
  - Group: Renal and Urinary Disorders (Including Infections)
    - Sub-group: Urinary Tract Infection
    - Sub-group: Urinary Frequency/Urgency

TauRx / Cytel Confidential: This is a controlled document and shall not be changed, saved, copied or reproduced by any unauthorized person.

- Group: Anemia and Related Terms
    - Subgroup: Anemia (excluding vitamin deficiencies)
- Group: Falls and Related Terms
    - Sub-group: Falls
- Group: Hypersensitivity
    - Sub-group: Rash
- Group: Renal Function Impairment
- Group: Hepatic Function Impairment
- Group: Behavioral and Psychological Symptoms of Dementia
    - Sub-group: Affective/Anxiety Symptoms
    - Sub-group: Behavioral Symptoms
    - Sub-group Psychotic Symptoms
    - Sub-group: Sleep Disorders
- Group: Cardiac Ischemia

- TauRx AE Groupings will be repeated for the following subgroups, showing only the main *TauRx AE Groupings*:
    - AChEI/memantine use (prior use, never used) [binary], repeated for
        - Discontinuation of AChEI and/or memantine before entering the study
        - Discontinuation of AChEI and/or memantine for purposes of study participation
    - Age group (<75 years, ≥75 years) [binary]
    - Sex (male, female) [binary]
    - Race (White, non-White) [binary], Race = White (if only race checked), otherwise Race = non-White (even if multiple races, including White, were checked)
    - Renal Function at Baseline: Creatinine Clearance (≤ 50 mL/min, > 50 mL/min) [binary]
    - Use or no use of concomitant medications with serotonergic potential (see latest version of the Serotonergic Drugs List, Section 5.8) at any time during the study [binary]
    - Use or no use of SSRI/SNRI [binary]
    - Use or no use of MAOI [binary]

TauRx / Cytel Confidential: This is a controlled document and shall not be changed, saved, copied or reproduced by any unauthorized person.

- The protocol-specified AEs of special interest (AESI), which are hemolytic anemia and lens discoloration (separate columns for true placebo and MTC). A list of all identified events will be provided to the Statistical Programming team.
- On-study TEAEs of subjects who withdrew from AChEI/memantine for purposes of the study (separate columns for true placebo and MTC)

No formal hypothesis-testing analysis of AEs incidence rates will be performed.

All AEs occurring on study will be listed in a subject data listing. Pre-treatment AEs (onset after informed consent and prior to the first dose of study drug) and post-treatment TEAEs (subset of TEAEs with an onset or worsen in intensity or treatment attribution more than 14 days after last dose of study drug) will be flagged. In addition, separate summaries of AEs by MedDRA SOC and PT will be provided for:

- Pre-treatment AEs
- Pre-treatment AEs of subjects who withdrew from AChEI/memantine for purposes of the study
- Post-treatment TEAEs (separate columns for true placebo and MTC)

Furthermore, subject data listings will be provided for the following: AEs leading to dose interruptions, SAEs (separate for fatal and non-fatal), SARs, AEs leading to withdrawal, and malignancies other than non-melanoma skin cancers.

A subject data listing will be provided, which presents additional SAE information.

5.12.3. **Laboratory Data**

Central laboratory data are transferred electronically by Labcorp (previously known as Covance). The Data Transfer Specification document provides a detailed description of the content and format of the laboratory datasets.

Clinical laboratory values will be reported in separate tables and listings for conventional and SI units, justified by the involvement of FDA and EMA.

The Baseline value is defined as the last non-missing value prior to first dose of study drug. Laboratory tests obtained on the date of the first dose will be assigned to pre-treatment; the relative times of blood sampling and dosing will be checked programmatically to confirm this assumption, if this is not true, the respective Screening value will be used.

The Baseline value, the actual value and change from Baseline to each on study evaluation and to the last available on-treatment value will be summarized for each clinical laboratory parameter, including hematology, and blood chemistry; restricted to subjects with at least one post-Baseline value. For continuous laboratory parameters n, Mean, Median, SD and (Min, Max) will be presented; and counts and percentages for categorical parameters. In the event of repeated values, the “worst” value per

TauRx / Cytel Confidential: This is a controlled document and shall not be changed, saved, copied or reproduced by any unauthorized person.

study day will be used. For the hematology parameters hemoglobin and hematocrit, this descriptive analysis will be prepared separately for males and females.

Visit windows are used when results are presented by target visit (see Table 5). For each parameter, if a subject has multiple values within a visit window, the "worst" value will be used for that visit window summary (see Appendix 12.3).

Shift tables will be provided showing the change from Baseline relative to the reference range. Missing categories will be included in the shift tables.

Box-and-whisker plots and line graphs (showing the mean and standard error) of the observed data will be presented for selected parameters including hemoglobin (separately for males and females), reticulocytes, neutrophils, platelet counts, and liver function tests (ALT, AST, GGT, total bilirubin). Conventional and SI units will both be presented within each figure (as two y-axes), along with reference lines for the normal ranges. Other parameters may be identified during data review.

The treatmentwise correlation (HMTM 16 mg/day, HMTM 8 mg/day, and control) between the G6PD value at Screening and the change in hemoglobin from Baseline up to Week 52 visit will be analyzed. Separate scatter plots will present the values of G6PD and hemoglobin, along with the treatmentwise p-values based on the parametric Pearson correlation. If the Week 52 visit value is not available, the last available on-treatment value will be used. In addition, the change in hemoglobin from Baseline up to Week 52 visit will be analyzed using an ANCOVA, including the randomization stratification variables (see Section 2.6) as fixed factors, the hemoglobin Baseline value and the total duration of exposure (see Section 5.12.1) as covariates, and the treatment indicator; with the Kenward and Roger method used for calculating the denominator degrees of freedom for the tests of fixed effects. This will be performed in two G6PD subgroups: one subgroup includes all subjects with a G6PD Screening value between 60 and 80 percent relative to the lower bound of the laboratory's reference range, and one subgroup includes all subjects with a G6PD Screening value higher than 80 percent relative to the lower bound of the laboratory's reference range.

All laboratory data (hematology, and blood chemistry) will be provided in subject data listings, including flags for values outside the reference range, as well as for values being potentially clinically significant (PCS, see Table 6), and for value obtained at a local laboratory. When there are thresholds provided for low and high values, they will be handled separately. A listing will also be provided that details the normal ranges from the central laboratory for all parameters in this study.

TauRx / Cytel Confidential: This is a controlled document and shall not be changed, saved, copied or reproduced by any unauthorized person.

Table 6 Laboratory – Potentially clinically significant values

Parameter	Criteria – SI Units	Criteria – Conventional Units
Hemoglobin	Female: ≤ 95 g/L<br>Male: ≤ 115 g/L<br>Decrease of ≥ 20%	Female: ≤ 9.5 g/dL<br>Male: ≤ 11.5 g/dL<br>Decrease of ≥ 20%
Hematocrit	Female: ≤ 0.32; Male: ≤ 0.37	Female: ≤ 32%; Male: ≤ 37%
WBC count	≤ 2.8 × 10^9/L<br>≥ 16 × 10^9/L	≤ 2800/μL<br>≥ 16000/μL
Neutrophils	≤ 1.0 × 10^9/L	≤ 1000/μL
Eosinophils	≥ 0.7 × 10^9/L	≥ 700/μL
Platelet count	≤ 75 × 10^9/L<br>≥ 700 × 10^9/L	≤ 75 × 10^3/μL<br>≥ 700 × 10^3/μL
Sodium	< 130 mmol/L<br>> 150 mmol/L	< 130 mEq/L<br>> 150 mEq/L
Potassium	< 3.0 mmol/L<br>> 5.5 mmol/L	< 3.0 mEq/L<br>> 5.5 mEq/L
Calcium (EDTA)	< 1.75 mmol/L<br>> 3.00 mmol/L	< 7.00 mg/dL<br>> 12.00 mg/dL
Glucose [a]	< 2.775 mmol/L<br>> 13.878 mmol/L	< 50 mg/dL<br>> 250 mg/dL
Albumin-BCG	< 25 g/L	< 2.5 g/dL
Total Bilirubin	≥ 34.2 μmol/L	≥ 2 mg/dL
ALT	≥ 3 × ULN	≥ 3 × ULN
AST	≥ 3 × ULN	≥ 3 × ULN
Alkaline Phosphatase	≥ 3 × ULN	≥ 3 × ULN
Urea Nitrogen	> 17.85 mmol/L	> 50 mg/dL

TauRx / Cytel Confidential: This is a controlled document and shall not be changed, saved, copied or reproduced by any unauthorized person.

Parameter	Criteria – SI Units	Criteria – Conventional Units
Creatinine	≥ 177 µmol/L	≥ 2 mg/dL
GGT	≥ 3 × ULN	≥ 3 × ULN
LDH	≥ 3 × ULN	≥ 3 × ULN
Phosphorus	< 0.646 mmol/L<br>> 1.777 mmol/L	< 2.0 mg/dL<br>> 5.5 mg/dL

[a] Independent from fasting status.

Tabular summaries will include two categories defined as follows:

- Subjects with any post-Baseline PCS – the unique number of people who meet the criterion (regardless of whether or not they met it at Baseline)
- Subjects with any post-Baseline PCS Worsening
    - Subjects who do not meet PCS criteria at Baseline but do post-Baseline or
    - Subjects who already met the PCS criterion at Baseline and in whom the post-Baseline value is worse than it was at Baseline.

Separate subject data listings for each hematology and blood chemistry parameter will include only subjects with treatment-emergent PCS values. For these subjects, all results for a parameter meeting the PCS criterion will be provided. For a set of selected parameters (see Table 7), related parameters will be added to the listing. The blood chemistry parameter eGFR is not documented in the CRF and will be calculated as follows using the MDRD equation: 175 x Creatinine$^{-1.154}$ x Age$^{-0.203}$ x (0.742 if female) x (1.212 if Black Or African American). The resulting unit is mL/min/1.73m^2.

TauRx / Cytel Confidential: This is a controlled document and shall not be changed, saved, copied or reproduced by any unauthorized person.

Table 7 Laboratory – Related parameters

Laboratory	Selected treatment-emergent PCS parameter	Related parameters
Hematology	Hemoglobin or Hematocrit	RBC count<br>Reticulocytes<br>Hemoglobin<br>Hematocrit<br>MCH<br>MCV
Hematology	WBC count or Neutrophils	WBC count<br>Neutrophils<br>Lymphocytes<br>Monocytes
Blood Chemistry	Urea Nitrogen or Creatinine	Urea Nitrogen<br>Creatinine<br>Creatinine Clearance<br>eGFR
Blood Chemistry	ALT or AST	ALT<br>AST<br>GGT<br>Total Bilirubin<br>Direct Bilirubin<br>Indirect Bilirubin<br>Alkaline Phosphatase<br>LDH

5.12.4. Vital Signs

The Baseline value, the actual value and change from Baseline to each on study evaluation and to the last available on-treatment value will be summarized, with the Baseline value being defined as the last non-missing value prior to first dose of study drug.

A similar summary will be generated for blood pressure values obtained on Day 1 (pre-dose and post-dose) in the subset of subjects identified as concomitantly taking the following pharmacological subgroup of serotonergic drugs at Baseline: SSRIs, SNRIs, or MAOIs (see latest version of the Serotonergic Drugs List, Section 5.8, and Section 12.4).

TauRx / Cytel Confidential: This is a controlled document and shall not be changed, saved, copied or reproduced by any unauthorized person.

PCS vital sign changes are defined in Table 8 and will need to be calculated and identified prior to further analysis. The number and percentage of subjects meeting these criteria (any post-Baseline PCS) will be summarized according to the definition described in Section 5.12.3. Increasing and decreasing changes will be categorized separately.

Vital sign measurements will be presented in a subject data listing.

Table 8 Vital signs – Potentially clinically significant values

Parameter	Criteria
Systolic Blood Pressure	Increase of ≥ 20 mmHg from Baseline and ≥ 180 mmHg<br>Decrease of ≥ 20 mmHg from Baseline and ≤ 90 mmHg
Diastolic Blood Pressure	Increase of ≥ 15 mmHg from Baseline and ≥ 105 mmHg<br>Decrease of ≥ 15 mmHg from Baseline and ≤ 50 mmHg
Pulse	Increase of ≥ 15 beats/min from Baseline and ≥ 120 beats/min<br>Decrease of ≥ 15 beats/min from Baseline and ≤ 50 beats/min
Weight	Decrease of ≥ 7% from Baseline<br>Decrease of ≥ 10% from Baseline
	Increase of ≥ 7% from Baseline<br>Increase of ≥ 10% from Baseline

5.12.5. **Physical and Neurological Examinations**

At Screening a complete physical (evaluation of the skin, head, eyes, ears/nose/throat, neck, thyroid, lungs, heart, lymph nodes, abdomen, and extremities) and neurological examination (evaluation of appearance and behavior, including observation for tremor and abnormal movements, and an evaluation of speech, cranial nerves (2-12), motor (muscle strength), muscle tone, sensory abnormalities, coordination, gait, and tendon reflexes) was performed. These results will be summarized by body system (by treatment group and overall).

Results from the targeted examinations performed at subsequent visits (including examinations after the first dose of study drug up to 14 days after the last dose date) will be summarized by presenting the number and percentage of subjects within each category by visit and by body system/parameter evaluated. For the physical examination, each body system is assessed as Normal or Abnormal. For the neurological examination, each body system is assessed as Normal, Abnormal or Absent.

All physical and neurological examination findings will be presented in subject data listings.

TauRx / Cytel Confidential: This is a controlled document and shall not be changed, saved, copied or reproduced by any unauthorized person.

5.12.6. Ophthalmological Examination

Slit lamp ophthalmological examination of subjects with lens implants will be performed at Screening and at the Week 52 visit (or upon early termination), to assess whether the lens has been discolored during the trial. In addition, the slit lamp examination could also be performed if a subject had cataract surgery / lens implantation at any point during his or her study participation (as soon as possible after the surgery), as well as in response to visual complaints if suggestive of lens discoloration.

The following information will be used to identify subjects with lens implant:

- eCRF Medical History: *Does the subject have intraocular lens implants?* = Yes
- eCRF Medical History: Coded PT term = Cataract or Cataract operation
- eCRF Procedures / Therapies: *Was this procedure an intraocular lens implant?* = Yes

Results from the examination will be summarized by treatment phase (pre-treatment, double-blind treatment phase, and open-label treatment phase), presenting the number of subjects with lens implant, the number of subjects with lens implant and at least one examination during each treatment phase, and the number and percentage of subjects within lens discoloration by treatment phase, and treatment group.

A subject data listing will display the date of the ophthalmological examination, whether the lens was discolored, and any additional details.

TauRx / Cytel Confidential: This is a controlled document and shall not be changed, saved, copied or reproduced by any unauthorized person.

6. STATISTICAL ANALYSIS OF OPEN-LABEL TREATMENT PHASE

6.1. Introduction

The rationale of a delayed-start design is that, when the active drug has a purely symptomatic effect and has no effect on neuropathologic process, a delay in administration should have no lasting effect on subjects (null hypothesis). An effect that slows the progression of disease by modifying the underlying biological pathology, rather than only attenuating symptoms, would be evident if late starters fail to "catch up" to early starters. Demonstrating a disease-modifying effect would imply a sustained benefit of starting such drugs early. Throughout (that is, both the controlled and delayed-start phases), all subjects and study personnel on-site are blinded to each subject's randomization to the early-start or late-start treatment group (HMTM treatment history).

Only subjects completing the 52-week double-blind treatment phase on- or off-treatment are eligible to enter the open-label treatment phase. This includes subjects, that have been randomized under a protocol version before version 5 and includes subjects who re-consented on a protocol version 5+ after the randomization and who completed the 52-week double-blind treatment phase. Subjects who have been off-treatment at Week 52 (TOTOS) stay off-treatment and do not receive any open-label treatment phase study drug.

6.2. Open-Label Treatment Phase Definitions

Based on the HMTM treatment history during the double-blind treatment phase "late" starters are defined as subjects originally randomized to control, and "early" starters are defined as subjects originally randomized to HMTM 16 mg/day or HMTM 8 mg/day. The last available assessment – for the scales: the last available (upscaled) value – prior to or on the same day of the start of the open-label treatment (Visit 7, Week 52) will serve as the Baseline assessment of the open-label treatment phase (Baseline-OL). A delayed performed Week 52 MRI assessment will be eligible for the Baseline-OL assessment if it has been done within 14 days after starting the open-label treatment phase.

The treatment group of subjects originally randomized to control will be labeled as "MTC 8 mg/week".

6.3. Summary Statistics

Summary tables and most of the efficacy tables will be presented by the original randomized treatment group from the double-blind treatment phase and will have the following labels:

- "MTC 8 mg/week -> HMTM 16 mg",
- "HMTM 8 mg -> HMTM 16 mg",

TauRx / Cytel Confidential: This is a controlled document and shall not be changed, saved, copied or reproduced by any unauthorized person.

- "HMTM 16 mg -> HMTM 16 mg", and
- "HMTM Pooled -> HMTM 16 mg".

The "MTC 8 mg/week -> HMTM 16 mg" group presents the "late starters" and the "HMTM Pooled -> HMTM 16 mg" group presents the "early starters" (i.e., HMTM 8 mg and HMTM 16 mg). An explanatory footnote will be added to explain this. A total column, combining the early and late starters, will be presented as needed.

Summary statistics are described in more details in the post hoc efficacy Section 6.12.

Subject data listings conducted during the double-blind treatment phase will be re-run including the open-label treatment phase data, if open-label treatment phase data is available. If appropriate, a flag for open-label treatment phase data will be added and the open-label treatment phase study day will be presented as well (for subjects who have entered the open-label treatment phase). The treatment group labels for subjects participating in the open-label treatment phase as well will be changed to: "MTC 8 mg/week -> HMTM 16 mg", "HMTM 8 mg -> HMTM 16 mg", and "HMTM 16 mg -> HMTM 16 mg"; whereas the treatment group labels for subjects not participating in the open-label treatment phase stay unchanged: "Placebo Only", "MTC 8 mg/week", "HMTM 8 mg", and "HMTM 16 mg".

The summaries of assessments affected by COVID-19 (see Section 4.8), which are presented by study visit for the ITT population, will be extended by the open-label treatment phase study visits.

6.4. Sample Size Justification

With about 400 subjects randomized to control or HMTM 16 mg/day under protocol version 5.0 and above, which is the primary treatment group comparison in the double-blind treatment phase, an estimated 160 to 170 subjects per arm will enter the open-label, delayed-start phase assuming the dropout rates from Section 5.3. Assuming a further 10% drop out in the delayed-start phase, the key secondary analysis to demonstrate disease modification by comparing early to late starters with a noninferiority margin of -2 ADAS-cog_{11} units has approximately 80% power.

6.5. Protocol Deviations

The protocol deviations file, which was finalized for the double-blind treatment phase analysis prior to the unblinding of the double-blind treatment phase will be updated prior to the database lock of the open-label treatment phase.

This file will include a description of the protocol deviation, will provide the information regarding previous classification (minor/major), and will provide the classification done by the Sponsor into important/non-important.

The level of detail and information will provide full traceability.

TauRx / Cytel Confidential: This is a controlled document and shall not be changed, saved, copied or reproduced by any unauthorized person.

Based on the ITTv5-OL and ITT-OL populations, summaries by categorization (important/non-important) and type (or code) will be provided. This will be done pooled and separately for COVID-19- and non-COVID-19-related protocol deviations. Also, one version will count all protocol deviations and one version will count only the overall unique deviations (when one deviation is resulting in subsequent ones).

6.6. Visit Windows

Since data might be documented under a wrong label (as a wrong visit) within the EDC system, all data will be (re-)assigned to a visit according to the column *Intervals for analysis* in Table 9.

Furthermore, if a subject has multiple values for a parameter within a visit window, the "worst" value will be used for that visit window summary (see Appendix 12.3), for efficacy endpoints the closest to the scheduled visit will be used (if two have the same distance from the scheduled visit the later one will be used).

For the analysis of the open-label treatment phase, only data after the first dose of the open-label treatment phase until the end of the study will be included, except Baseline-OL values, which are based on data assessed prior to the first dose of the open-label treatment phase. A delayed performed Week 52 MRI assessment will be eligible as the Baseline-OL assessment if it has been done within 14 days after starting the open-label treatment phase. In addition, eye exams will be assigned to the double-blind treatment phase, if it was performed until the end of the Week 52 protocol-specified visit window.

Table 9 Evaluation Intervals of the Open-Label Treatment Phase

Evaluation (scheduled day)	Protocol-Specified Interval	Intervals for Analysis		
		Safety assessments	ADAS-cog_{13}, ADCS-ADL_{23}, MRI	MMSE, CDR
Week 52 (365) [a]	351 to 379 [±14]	320 to 379	320 to 456	320 to 548
Open-Label Week 56 (394)	380 to 408 [±14]	380 to 470	-	-
Open-Label Week 78 (548)	534 to 562 [±14]	471 to 638	457 to 638	-
Open-Label Week 104 (730)	716 to 744 [±14]	≥639	≥639	>548

[a] Baseline visit for open-label treatment phase

TauRx / Cytel Confidential: This is a controlled document and shall not be changed, saved, copied or reproduced by any unauthorized person.

6.7. Subject Disposition

The subject by-visit listing will be updated with the open-label treatment phase visits.

Based on the ITT population, the subject data listing from the double-blind treatment phase will be updated and extended with the disposition information from the open-label treatment phase:

- Protocol version under which the subject has been randomized
- First and last dose dates of study drug intake (by treatment phase)
- Last date (by treatment phase)
- A flag for the completion, and a flag for the TOTOS (see Section 4.4) (by treatment phase)
- All reasons why the subject did not complete the study/treatment phase, and a flag for the primary reason
- For the TOTOS, the reason why the subject discontinued the study drug

Based on the ITT-OL population, a subject data listing will provide an overview of the open-label treatment phase populations (see Section 3.1.2) and will list the following events (sorted by subject, and date; one row for each event):

- Affiliation to the study open-label treatment phase populations (one column per study population)
- Date of Week 52 visit
- Protocol re-consents during the open-label treatment phase, with the protocol version and signing dates (one row for each re-consent)
- Date and primary reason of study discontinuation during the open-label treatment phase
- Date of study drug discontinuation during the open-label treatment phase, if study drug discontinuation is permanent and if the date is different from the date of study discontinuation
- Any date of COVID-19 infection (during double-blind or open-label treatment phase)
- Any date of COVID-19 vaccination (during double-blind or open-label treatment phase)
- Date of Week 104 visit

The corresponding listing from the double-blind treatment phase will also be updated with the following information:

- Protocol re-consents during the open-label treatment phase, with the protocol version and signing dates (one row for each re-consent)
- Date and primary reason of study discontinuation during the open-label treatment phase

TauRx / Cytel Confidential: This is a controlled document and shall not be changed, saved, copied or reproduced by any unauthorized person.

- Date of study drug discontinuation during the open-label treatment phase, if study drug discontinuation is permanent and if the date is different from the date of study discontinuation
- Any date of COVID-19 infection (during double-blind or open-label treatment phase)
- Any date of COVID-19 vaccination (during double-blind or open-label treatment phase)
- Date of Week 104 visit

Information on the number of subjects in each analysis population of the open-label treatment phase, the number of subjects completing the open-label treatment phase / the study (subsets on and off treatment), the number of subjects discontinuing the study drug but continuing the study, the primary reason for discontinuation of study drug and study, and all reasons for discontinuation of study drug and study will be tabulated. This will be done by HMTM treatment history and overall, for the ITTv5-OL, ITT-OL, and PPv5-OL populations. Furthermore, based on the ITTv5-OL population, this will be tabulated by region.

6.8. Demographic and Baseline Characteristics

The Demographic and Baseline table of the double-blind treatment phase will be repeated for the ITTv5-OL, ITT-OL, and PPv5-OL populations and will be extended by the Baseline-OL values of weight, creatinine clearance, MMSE, and CDR.

The subject data listing of the double-blind treatment phase will be extended by the Baseline-OL values.

No formal statistical comparisons of treatment groups for any Baseline characteristics will be performed.

6.9. Prior and Concomitant Medications

Any medication that has been discontinued before the intake of the first dose of the open-label treatment phase study drug will be defined as prior medication. Any medication that was taken at the day of the first dose of the open-label treatment phase study drug or after will be defined as concomitant medication. Prior used medications and concomitant medications will be coded using the 01 March 2017 version of the WHO drug dictionary and the ATC level 1 term, ATC level 3 term, and PT will be presented in the analysis.

Data conventions are as described in Section 5.8.

The subject data listing of prior medications, and concomitant medications of the double-blind treatment phase will be updated, and the records of the open-label treatment phase medications will be added. An additional flag will be added to indicate the concomitant medications of the open-label treatment phase. A subset of this subject data listing will also be provided for subjects who were using

TauRx / Cytel Confidential: This is a controlled document and shall not be changed, saved, copied or reproduced by any unauthorized person.

an antipsychotic treatment (during the double-blind or open-label treatment phase, see Section 12.4), including the reasons for use.

The following summaries/tabulations will be prepared for the ITTv5-OL and ITT-OL populations.

Tabulations with frequency and percentage, by treatment history and for the pooled treatment histories, will be prepared separately for all prior, for all concomitantly used drugs, and for all concomitantly used drugs that the patient is on at the time of the first dose of the open-label treatment phase study drug. Subjects reporting more than one medication are counted only once within each level summation.

Summary tables will be provided, showing the numbers of subjects with initiated treatments of SSRI/SNRI (see Section 12.4), drugs of serotonergic potential (see latest version of the Serotonergic Drugs List, Section 5.8), and any antipsychotic medications. For antipsychotics initiated on-treatment, the reasons for use will be also summarized.

6.10. Secondary Efficacy Endpoint Analysis

The goal of the open-label phase is to demonstrate disease modification by comparing the ADAS-cog_{11} change from Baseline-OL at Week 104 between early and later starters using a non-inferiority margin. According to the study protocol the non-inferiority margin will be set to 2 units, based on the estimated treatment effect of 5.2 ± 1.3 (mean ± standard error) units from a pooled analysis of the studies TRx-237-005 / TRx-237-015.

This non-inferiority margin, and the thresholds mentioned in Section 6.11, have been selected based on the results of the two previous phase 3 studies TRx-237-005, and TRx-237-015. It was planned to change the non-inferiority margin to 50% of the observed 52 treatment effect, should the decline and treatment effect for this study, seen after topline and full analysis of the double-blind treatment phase, deviate strongly from the expectations based on the TRx-237-005/TRx-237-015 results. With the knowledge of the double-blind treatment phase results, the non-inferiority margin will be as selected, based on the two previous phase 3 studies TRx-237-005, and TRx-237-015.

Efficacy analyses will be conducted using the PPv5-OL population (see Section 3.1.2) with no imputation of missing values. Upscaling for the scales will be performed. The ITTv5-OL population will be analyzed as a supplementary analysis and will include only those subjects with at least one post-Baseline-OL value.

The key secondary endpoint is the Baseline-OL adjusted decline in ADAS-cog_{11} from Baseline-OL at Week 104. Repeated measures on this endpoint scheduled at Week 78, and Week 104 will be treated using a mixed effects model. Suppose μ_{early} and μ_{late} are the corresponding means of decline at Week 104 for the early and late starters, then the null and alternative hypothesis can be written as:

H_0: $\mu_{early} - \mu_{late} \geq 2$; versus

H_1: $\mu_{early} - \mu_{late} < 2$.

The analyses will be performed using the restricted maximum likelihood-based MMRM, including the randomization stratification variables (see Section 2.6) as fixed factors, the ADAS-cog$_{11}$ Baseline-OL value as covariate, and the interaction term between early/ late starters (HMTM treatment history) indicator and visit; with an unstructured covariance matrix and the Kenward and Roger method of calculating the denominator degrees of freedom for the tests of fixed effects. According to the hypotheses, this is a lower-tailed test which will be performed at the alpha=0.025 level of significance.

Sample SAS code (will be fully validated at the analysis stage):

```
PROC MIXED DATA=dataset METHOD=REML COVTEST;
CLASS subject treatmenthist(REF=’Late’) visit severity region prioruse;
MODEL scorediff = treatmenthist|visit severity region prioruse scorew52 / SOLUTION CL
DDFM=kenwardroger;
REPEATED visit / SUB=subject TYPE=UN;
LSMEANS treatmenthist*visit visit / CL;
LSMESTIMATE treatmenthist*visit “intervention effect at Week 104” 0 1 0 -1 / CL testvalue=2
LOWER ALPHA=.025;
*Summary statistics for treatment difference based on LSM
LSMESTIMATE treatment*visit “intervention effect at Week 78” 1 0 -1 0 / CL testvalue=2 LOWER
ALPHA=.025;
RUN;
```

6.10.1. Sensitivity Analysis

The subgroups of subjects originally randomized to HMTM 16 mg/day and 8 mg/day will be analyzed separately, as described in Section 6.10.

6.11. Exploratory Efficacy Analysis

ADCS-ADL$_{23}$, and the MRI parameters (whole brain and temporoparietal lobe) will be analyzed analogously to the ADAS-cog$_{11}$, see Section 6.10, to directionally support a disease modifying argument. Subgroup analyses might be done in a post-hoc analysis.

TauRx / Cytel Confidential: This is a controlled document and shall not be changed, saved, copied or reproduced by any unauthorized person.

6.11.1. ADCS-ADL$_{23}$

Compared to ADAS-cog$_{11}$, with a similar range of values and estimated treatment effect of 5.0 ± 1.6 (mean ± standard error) units from a pooled analysis of the studies TRx-237-005 / TRx-237-015, the non-inferiority margin will be set to -2.

The null and alternative hypothesis can be written as:

H_0: $\mu_{early} - \mu_{late} \leq -2$; versus

H_1: $\mu_{early} - \mu_{late} > -2$.

Regarding the SAS code, the *UPPER* option will be used in the *LSMESTIMATE* statement.

6.11.2. MRI

For the MRI parameters the non-inferiority margin will be set to -13,000 (mm^3 = µL) for whole brain volume and -800 (mm^3 = µL) for the temporoparietal lobe volume.

6.12. Post Hoc Efficacy Analysis

The following efficacy analysis have been added after partial unblinding of study team members. The partial unblinding was done to be able to perform the analysis of the double-blind treatment phase.

To complete the efficacy analysis (see Sections 6.10 and 6.11), based on the ITTv5-OL population (see Section 3.1.2), summary statistics of the absolute values and their change from Baseline-OL of the scales (ADAS-cog$_{11}$, ADAS-cog$_{13}$, ADCS-ADL$_{23}$, Composite Scale, and COVID-19 Composite Scale), MRI parameters (whole brain, temporoparietal lobe, putamen, nucleus accumbens, nucleus basalis, hyperintensities total volume, and hypointensities total lesion volume) will be tabulated by visit (including Baseline-OL, Week 78, and Week 104) and HMTM treatment group as described above. MMSE and CDR-SOB will be tabulated by visit (including Baseline-OL and Week 104) and HMTM treatment group. Upscaling rules will be applied (see Section 4.5.1) and analyses are performed on these upscaled values. The summary statistics of ADAS-cog$_{11}$, ADCS-ADL$_{23}$, and the MRI parameters whole brain volume and temporoparietal lobe volume will be presented based on the PPv5-OL population as well.

Also, summary statistics and treatment differences (with 95% confidence intervals) based on the least squares means (LSM) from the corresponding MMRM models (see Sections 6.10 and 6.11) will be presented for the study visits Baseline-OL, Week 78, and Week 104.

Based on the ITTv5-OL population, for ADAS-cog$_{13}$, ADAS-cog$_{11}$, ADCS-ADL$_{23}$, and the two MRI parameters whole brain volume and temporoparietal lobe volume, line graphs (showing the LSM and standard error) will be generated, presenting the MTC 8 mg/week group and the HMTM 16 mg group (along with their corresponding tables, presenting the LSM and treatment contrast for each visit). The LSM are based on a restricted maximum likelihood-based MMRM including a nominal visit variable, the

TauRx / Cytel Confidential: This is a controlled document and shall not be changed, saved, copied or reproduced by any unauthorized person.

treatment indicator and the interaction term between treatment and visit, the randomization stratification variables (see Section 2.6) as fixed factors, and the respective efficacy Baseline/Baseline-OL value as covariate; with an unstructured covariance matrix and the Kenward and Roger method of calculating the denominator degrees of freedom for the tests of fixed effects. One version of the line graph will include/present the visits from Baseline to Week 104, including Baseline as the covariate in the model, and a second version will include/present the visits from Baseline-OL to Week 104, including Baseline-OL as the covariate in the model. This will be repeated for the verified AD diagnosis subgroups MCI-AD and probable AD.

Based on the ITTv5-OL population, the following pairwise correlations will be analyzed by means of scatter plots with symbolized treatment groups, which include both the parametric Pearson correlation coefficient and the non-parametric Spearman's rank correlation coefficient and their corresponding p-values for each treatment group, along with the regression lines:

- (Changes from Baseline): MRI whole brain volume at Week 52 vs. ADAS-cog_{11} at Week 104
- (Changes from Baseline): MRI whole brain volume at Week 52 vs. ADCS-ADL_{23} at Week 104
- (Changes from Baseline): MRI whole brain volume at Week 52 vs. CDR-SOB at Week 104
- (Changes from Baseline): MRI whole brain volume at Week 52 vs. MMSE at Week 104

Based on the ITTv5 population, for ADAS-cog_{13}, ADAS-cog_{11}, ADCS-ADL_{23}, and the two MRI parameters whole brain volume and temporoparietal lobe volume, the observed change from Baseline at Weeks 26, 52, 78, and 104 will be analyzed with a paired t-test, separately for the subjects originally randomized to HMTM 16 mg/day and subjects originally randomized to MTC 8 mg/week. Two-sample t-test comparisons between the treatment groups for the respective endpoints and each of the Weeks 26, 52, 78, and 104 will be presented as well. This will be repeated for the verified AD diagnosis subgroups MCI-AD and probable AD.

Based on the ITTv5 population, for the ADAS-cog_{11}, the change from Baseline at Week 52 will be analyzed with the paired t-test, separately for the subjects originally randomized to HMTM 16 mg/day and subjects originally randomized to MTC 8 mg/week. The change from Baseline-OL to Week 104 based on the ITTv5-OL population will be analyzed the same way. This will be repeated for the verified AD diagnosis subgroups MCI-AD and probable AD.

Sample SAS code (will be fully validated at the analysis stage):

```
PROC TTEST DATA=dataset ALPHA=.05;
BY treatment;
VAR scorediff;
RUN;
```


TauRx / Cytel Confidential: This is a controlled document and shall not be changed, saved, copied or reproduced by any unauthorized person.

6.13. Safety Analysis

Safety analyses will be conducted using the Safety-OL population (see Section 3.1.2), presenting the data from the open-label treatment phase, whereas the last non-missing value prior to the first dose of the open-label treatment phase study drug will serve as the Baseline-OL value.

Entries of the open-label treatment phase will be added to the subject data listings, which have been prepared for the double-blind treatment phase. An additional flag will be added to indicate the entries of the open-label treatment phase. Updated data and additional entries of the double-blind treatment phase, other than new information on AEs, will need to be investigated.

To assess the safety and tolerability of the HMTM treatment given for up to 104 weeks, exposure, compliance, AEs, and laboratory data will be analyzed including data from the double-blind and open-label treatment phases. For subjects originally randomized to control, data gathered during the double-blind treatment phase will be excluded from this analysis, unless it is needed for the determination of a Baseline. These analyses will be performed based on the Safety-HMTM population (see Section 3.1.3).

6.13.1. Extent of Drug Exposure and Compliance

The planned number of tablets taken by the subject each day for 52 weeks is four (2 tablets twice per day), with each tablet containing 4 mg of HMTM.

Based on the Safety-OL population, the following information will be summarized descriptively by treatment history and pooled treatment histories for the open-label treatment phase:

- Total duration of exposure (in weeks): (last dose date – first dose date + 1)/7
- Total duration of treatment (in weeks), which excludes days in which dose was interrupted
- Mean daily dose: Total of administered dose during the duration of exposure, relative to the total duration of exposure
- Total subject-years of exposure: sum of total exposure in years

$$\frac{1}{365.25}\sum_{subjects}\frac{\#tablets\ taken}{\#tablets\ planned} * total\ duration\ of\ exposure\ (days)$$

- Compliance: # Tablets taken / # tablets planned during the open-label treatment phase (or until early study drug termination), respectively. Including frequency and percentage of subjects with <80% and >120% compliance. The compliance values will be re-calculated and are not taken from the eCRF.
- Frequency and percentage of subjects with dose interruptions

TauRx / Cytel Confidential: This is a controlled document and shall not be changed, saved, copied or reproduced by any unauthorized person.

- Frequency and percentage of subjects in the duration of exposure categories, based on total duration of exposure, including interruption days. The exposure categories can be taken from Section 5.12.1 (as done for the double-blind treatment phase analysis).

This summary will be also prepared including the combined treatment information from the double-blind and open-label treatment phases based on the Safety population, and for the HMTM treatment information from the double-blind and open-label treatment phases based on the Safety-HMTM population (see Section 3.1.3), which means that control treatment information during the double-blind treatment phase will be excluded from the analysis of the HMTM treatment information. The following exposure categories (in days) will be added for this analysis: 366-457, 458-548, 549-639, 640-730, and >730. For the summary of the combined treatment information, the treatment groups will distinguish between subjects who received no open-label treatment and subjects who did receive at least one dose of open-label treatment (e.g., MTC 8 mg/week, MTC 8 mg/week -> HMTM 16 mg).

For additional more general information regarding exposure and compliance, please see Section 5.12.1.

6.13.2. **Adverse Events**

Adverse events (AEs) will be coded using the MedDRA version 20.1 and are displayed in tables and listings using SOC and Preferred Term (PT).

TEAEs during the open-label treatment phase are defined as AEs with:

- Onset after first dose of study drug of the open-label treatment phase or
- Worsen in intensity after first dose of study drug of the open-label treatment phase or
- Worsen in treatment relationship after first dose of study drug of the open-label treatment phase.

The total number of subjects and the numbers stratified by MedDRA SOC and PT, along with the corresponding percentage, will be derived and summarized by the original randomized treatment group from the double-blind treatment phase (MTC 8 mg/week, HMTM 8 mg/day, HMTM 16 mg/day), the pooled HMTM group (HMTM 8 mg/day + HMTM 16 mg/day), and overall, for the following events:

- Any TEAE
- Any TEAE with an onset on the first day of the open-label treatment phase after the first dose of the open-label treatment phase, complemented by a subject data listing
- Any TEAE (categorized by severity)
- Any TEAEs assessed by the Investigator as related to treatment (i.e., related or possibly related)
- Any TEAEs severe in intensity and assessed by the Investigator as related to treatment (as defined above)

TauRx / Cytel Confidential: This is a controlled document and shall not be changed, saved, copied or reproduced by any unauthorized person.

- Any TEAE that resulted in interruption or discontinuation of study drug (presented separately and combined)
- Any serious adverse event (SAE)
- Any serious adverse reaction (SAR), which are the SAEs judged to be possibly related or related to the study drug by the Investigator
- Malignancies other than non-melanoma skin cancers
- *TauRx AE Groupings*: Subsets of TEAEs (defined by PT), summarized per grouping and sub-grouping (see Section 5.12.2).
- The protocol-specified AEs of special interest (AESI), which are hemolytic anemia and lens discoloration. A list of all identified events will be provided to the Statistical Programming team.

In addition, separate summaries of AEs by MedDRA SOC and PT will be provided for:

- Post-treatment TEAEs (after the open-label treatment phase)

For TOTOS subjects from the double-blind treatment phase, which continued off-treatment during the open-label treatment phase, a sub-population of the ITT-OL population – which are not included in the Safety-OL population, the following table and listing will be prepared:

- Any TEAE
- Subject data listing, which presents additional SAE information

HMTM Treatment Phase

For the Safety-HMTM population (see Section 3.1.3), AEs of/ during the HMTM treatment will be analyzed. This means, that for subjects originally randomized to control, events of/ during the double-blind treatment phase (before the first dose of HMTM treatment during the open-label treatment phase) are excluded from this analysis.

The total number of subjects and the numbers stratified by MedDRA SOC and PT, along with the corresponding percentage, will be derived and summarized by the original randomized treatment group from the double-blind treatment phase (MTC 8 mg/week, HMTM 8 mg/day, HMTM 16 mg/day), the pooled HMTM group (HMTM 8 mg/day + HMTM 16 mg/day), and overall, for the following events:

- Any TEAE
- Any TEAE (categorized by severity)
- Any TEAEs assessed by the Investigator as related to treatment (i.e., related or possibly related)

TauRx / Cytel Confidential: This is a controlled document and shall not be changed, saved, copied or reproduced by any unauthorized person.

- Any TEAEs severe in intensity and assessed by the Investigator as related to treatment (as defined above)
- Any TEAE that resulted in interruption or discontinuation of study drug (presented separately and combined)
- Any serious adverse event (SAE)
- Any serious adverse reaction (SAR), which are the SAEs judged to be possibly related or related to the study drug by the Investigator
- Malignancies other than non-melanoma skin cancers

For additional more general information regarding AEs, please see Section 5.12.2.

6.13.3. **Laboratory Data**

Based on the (double-blind treatment phase) Safety population, the Baseline value, the actual value and change from Baseline for the Week 13, Week 26, Week 39, Week 52, Week 78, and Week 104 visits will be summarized by treatment group for the clinical laboratory parameters hemoglobin (separately for males and females), neutrophils, ALT, and AST; restricted to subjects with at least one post-Baseline value. Line graphs (showing the mean and standard error) of the observed data from Baseline to Week 104 will be presented for those parameters as well. The treatment groups will distinguish between subjects who received no open-label treatment and subjects who did receive at least one dose of open-label treatment (e.g., MTC 8 mg/week, MTC 8 mg/week -> HMTM 16 mg).

The Baseline-OL value is defined as the last non-missing value prior to the first dose of the open-label treatment phase study drug. Laboratory tests obtained on the date of the first dose will be assigned to pre-treatment; the relative times of blood sampling and dosing will be checked programmatically to confirm this assumption; if this is not true, a prior value will be used.

The Baseline-OL value, the actual value and change from Baseline-OL for the Week 56, Week 78, Week 104 visits, and for the last available on-treatment value will be summarized by treatment history for each clinical laboratory parameter, including hematology, and blood chemistry; restricted to subjects with at least one post-Baseline-OL value. For the hematology parameters hemoglobin and hematocrit, this descriptive analysis will be prepared separately for males and females.

Visit windows are used when results are presented by target visit (see Table 5 and Table 9). For each parameter, if a subject has multiple values within a visit window, the “worst” value will be used for that visit window summary (see Appendix 12.3).

Shift tables will be provided showing the change from Baseline-OL relative to the reference range. Missing categories will be included in the shift tables.

Box-and-whisker plots and line graphs (showing the mean and standard error) of the observed data from Baseline-OL to Week 104 (excluding visit Week 56) will be presented for the selected parameters

TauRx / Cytel Confidential: This is a controlled document and shall not be changed, saved, copied or reproduced by any unauthorized person.

as described in Section 5.12.3. In addition to the original randomized treatment groups from the double-blind treatment phase, the figures will present the HMTM Pooled group results as well. The treatment group labels will be as described in Section 6.3.

Analogously to the double-blind treatment phase analysis, a summary will present two categories of PCS values:

- Subjects with any post-Baseline-OL PCS – the unique number of people who meet the criterion (regardless of whether or not they met it at Baseline-OL)
- Subjects with any post-Baseline-OL PCS Worsening
    - o Subjects who do not meet PCS criteria at Baseline-OL but do post-Baseline-OL or
    - o Subjects who already met the PCS criterion at Baseline-OL and in whom the post-Baseline-OL value is worse than it was at Baseline-OL.

When the PCS values are summarized, assessments after the first dose of the open-label treatment phase, that have been assigned to Week 52 by using the visit windowing (see Section 6.6), will be analyzed as Week 56 values.

The separate subject data listings with treatment-emergent PCS values will be created analogously to the double-blind treatment phase analysis, presenting the Baseline-OL and post-Baseline-OL PCS values.

HMTM Treatment Phase

Since the scheduled study visits in the open-label treatment phase are planned every 26 weeks and not every 13 weeks as in the double-blind treatment phase, the analysis of the HMTM treatment phase will be divided into 26-week intervals. Visit windowing will be performed for the laboratory data based on the (double-blind treatment phase) study day for the early starters and based on the open-label treatment phase study day for the late starters. The following visit windows will be used: ≤1 (pre-dose) (Baseline-HMTM), 1 (post-dose) to 273 (Week 26, [day 183]), 274 to 456 (Week 52, [365]), 457 to 638 (Week 78, [548]), and ≥639 (Week 104, [730]), with the Baseline-HMTM value being defined as the last non-missing value prior to the first dose of HMTM treatment. If a subject has multiple values for a parameter within a visit window the rule from Section 5.5 will be used. For the subjects originally randomized to control, the values of the visits at Week 78 and 104 will be analyzed as Week 26 and Week 52 due to the delayed start of the HMTM treatment.

The following analysis will be performed based on the Safety-HMTM population (see Section 3.1.3).

The actual value and change from Baseline-HMTM for the Week 26, Week 52, Week 78, and Week 104 visits, and the last available on-treatment value, will be summarized by treatment history and overall, for each clinical laboratory parameter, including hematology, and blood chemistry; restricted to

TauRx / Cytel Confidential: This is a controlled document and shall not be changed, saved, copied or reproduced by any unauthorized person.

subjects with at least one post-Baseline-HMTM value. For the hematology parameters hemoglobin and hematocrit, this descriptive analysis will be prepared separately for males and females.

Line graphs (showing the mean and standard error) of the observed data from Baseline-HMTM to Week 104 will be presented for selected parameters as described in Section 5.12.3. The treatment groups will be presented as described earlier in this section for the separate open-label treatment phase.

For additional more general information regarding laboratory analysis, please see Section 5.12.3.

6.13.4. **Vital Signs**

The Baseline-OL value, the actual value and change from Baseline-OL for the Week 56, Week 78, Week 104 visits, and for the last available on-treatment value will be summarized by treatment history, with the Baseline-OL value being defined as the last non-missing value prior to first dose of the open-label treatment phase study drug.

A tabular summary of subjects with PCS vital sign changes from Baseline-OL will be prepared analogously to the double-blind treatment phase table (see Section 5.12.4). When the PCS values are summarized, assessments after the first dose of the open-label treatment phase, that have been assigned to Week 52 by using the visit windowing (see Section 6.6), will be analyzed as Week 56 values.

6.13.5. **Physical and Neurological Examinations**

The summaries from the double-blind treatment phase (see Section 5.12.5) will be repeated for the Safety-OL population and extended by the visits Week 56, Week 78, and Week 104.

6.13.6. **Ophthalmological Examination**

The summary from the double-blind treatment phase (see Section 5.12.6) will be repeated for the Safety-OL population.

The subject data listing prepared for the double-blind treatment phase (see Section 5.12.6) will be extended by the open-label treatment phase visit(s). An additional flag will be added to indicate the entries of the open-label treatment phase.

TauRx / Cytel Confidential: This is a controlled document and shall not be changed, saved, copied or reproduced by any unauthorized person.

7. PLASMA BIOMARKER ANALYSIS

The analysis described in this section is based on version 2.2 from 16-MAR-2023 of the TRx-237-039 Secondary Research Plan "Plasma Biomarker Analysis", in which a detailed background, scientific rationale, and sample size justification is given.

The following analysis are all exploratory of nature. The analyses stand on their own and corresponding nominal p-values will be reported and no correction for multiple comparisons will be conducted.

A subject data listing of the biomarkers will be provided.

The tau fragment analysis, which was introduced with the version 2.1 of the Secondary Research Plan, is based on a subset of pre-dose Baseline back-up PK plasma samples is not part of this SAP. This analysis will be performed by the University of Aberdeen (Scottish Biologics Facility).

7.1. Biomarker

The initial analysis focuses on the following primary exploratory biomarkers, which will be analyzed in the framework of primary and secondary biomarker endpoints:

- Tau phosphorylated at residue 181 (P-tau181)
- Neurofilament light (NfL)

The null hypotheses for each of these biomarkers are, that there is no difference from Baseline to Week 52 visit within the HMTM group and that HMTM does not have an impact on change of the respective endpoint from Week 52 to Baseline compared to control. For correlations between biomarkers and between other clinical study endpoints (see Section 7.3.3), the null hypothesis is that there is no correlation between those.

The following potential biomarkers are 'other' exploratory biomarker research and will be analyzed analogously to the primary exploratory biomarkers upon request, with T-tau being planned to be analyzed with version 2.2 of the Secondary Research Plan:

- Tau phosphorylated at residue 231 (P-tau231)
- Core-proline tau
- Total tau (T-tau)
- Glial fibrillary acidic protein (GFAP)

There is no prior evidence to show that these biomarkers will show a pharmacodynamic response to treatment with HMTM nor any other approved or investigational drugs for AD. Given this, no hypotheses are provided, but the above null-hypothesis apply here as well.

TauRx / Cytel Confidential: This is a controlled document and shall not be changed, saved, copied or reproduced by any unauthorized person.

For the avoidance of doubt, even in the cases where a clear hypothesis exists which direction the change should take, all tests will be conducted at the two-sided, 0.05 level of significance.

7.1.1. Biomarker Samples

The investigated (potential) biomarkers are pre-analyzed in different companies and institutions: P-tau 181 and P-tau231 (Medpace Inc.), NfL (Drug Development Solutions Ltd.), Core-proline tau (University of Aberdeen Good Laboratory Practice Test Facility), T-tau (University College London Consultants Ltd.), and GFAP (*to be decided*). Due to different processing schedules, the pre-processed data will be provided to Cytel in batches and therefor the statistical analysis will be performed in batches.

Plasma samples have been taken at Baseline, Week 4, Week 52, and Week 104 visits, on three occasions: pre-dose, approximately 1 to 2 hours post-dose, and approximately 4 hours post-dose.

The biomarker data analysis is based on back-up PK plasma samples taken pre-dose during the Baseline and Week 52 visits.

On a subject level: If the pre-dose sample is unavailable, the earliest available post-dose sample (first: 1-2 hours post-dose, second: 4 hours post-dose) will be used; and furthermore, if the back-up plasma sample is unavailable the residual primary PK plasma will be utilized instead, if available. If no proper sample is available, the value is set to missing. This means, the order of samples is as follows:

- Back-up sample: 1. Pre-dose, 2. 1-2 hours pose-dose, 3. 4 hours post-dose
- Residual primary sample: 4. Pre-dose, 5. 1-2 hours pose-dose, 6. 4 hours post-dose
- 7. “Missing”

The first sample which is available will be used for the analysis. This means, for the avoidance of doubt, the analysis will be performed based on all available samples, irrespective of the actual sample used. Thus, pre-dose versus post-dose samples as well as primary versus back-up samples will be used alike. The only relevant characteristic of the sample is, which is used in the analysis, if it is Baseline or Week 52 visit.

Possible reasons for unavailability of a plasma sample are:

- More than one previous freeze-thaw cycle.
- An approximate hemoglobin concentration of 250 mg/dL or more.
- Incorrect collection of a sample.

For completeness, a summary table will be provided, that shows the number of samples used for each biomarker presented by treatment group and total, that were Baseline pre-dose, Baseline 1-2 hours post-dose, Baseline 4 hours post-dose, Week 52 pre-dose, Week 52 1-2 hours post-dose, and Week 52 4 hours post-dose. If needed, this will be further separated by back-up sample and primary sample.

TauRx / Cytel Confidential: This is a controlled document and shall not be changed, saved, copied or reproduced by any unauthorized person.

7.2. Analysis Population

Biomarker Population: subjects randomized under protocol version 5.0 or higher, which completed the double-blind treatment phase, excluding the TOTOS (see Section 4.4). Subjects randomized under a protocol version before version 5 with available biomarker data at Week 52 and completed double-blind treatment phase will be included as well.

7.3. Statistical Analysis of Biomarker Outcomes

The following analysis will be performed based on the Biomarker Population.

Week 52 samples taken after the first day of the open-label treatment phase will not be used in the analysis.

Since from protocol version 5 on, the control group had occasional spiking with 4mg MTC with an average frequency of twice per week, it is therefore of interest to test not only the doses of HMTM compared to control, but also to a subpopulation of control that showed lowest predicted concentrations of parent MT at Week 52. A threshold of <=0.1 ng/mL for predicted Week 52 steady-state (Cmax,ss) parent MT concentration will be used to define the lowest parent MT group. In the analysis output the control group will be presented as “MTC 8 mg/week”.

The actual received treatment and the actual (verified) values of the randomization stratification variables will be used in the analysis.

7.3.1. Summary Statistics

The Baseline and Week 52 visit value of each biomarker to be analyzed, of the scales (ADAS-cog_{13}, ADAS-cog_{11}, and ADCS-ADL_{23}) and MRI and ^{18}F-FDG-PET parameter as mentioned in Section 7.3.3, will be analyzed descriptively (n, mean, median, lower quartile (Q1), upper quartile (Q3), standard deviation, standard error, minimum and maximum values) and will be presented by treatment group, and for the lowest parent MT group as a separate group, and by Baseline MMSE score subgroups.

Baseline MMSE score subgroups are defined as 16-19, and 20-27. An additional split of the scores into 16-21, and 22-27 will be investigated. Also, a split into the verified AD diagnosis subgroups MCI-AD and probable AD will be provided as per the information provided in the Diagnostic Verification form.

The descriptive analysis of the scales and parameters will be repeated, since the analysis population is different to the double-blind treatment phase populations.

7.3.2. Primary Outcomes

For each of the primary exploratory biomarkers P-tau181 and NfL, an ANCOVA will be used to analyze the change from Baseline at Week 52 visit within each treatment group (HMTM 16 mg/day, HMTM 8 mg/day, and control). In this ANCOVA, the change from Baseline at Week 52 will be compared pairwise

for these three treatment groups. The ANCOVA model will include the randomization stratification variables (see Section 2.6) as fixed factors, the respective efficacy Baseline value as covariate, and the treatment indicator; with the Kenward and Roger method used for calculating the denominator degrees of freedom for the tests of fixed effects. One table will present the LSM for the change from Baseline and the pairwise treatment contrasts at Week 52, and one table will present the p-values of the type 3 tests of the fixed effects along with the estimates of the factors and covariates which are included in the model.

In a sensitivity analysis, subject's age and sex, as well as *ApoE*4 carrier status and plasma sample age will be added as further covariates in the primary model. The plasma sample age (of the Week 52 sample) is the difference between the day when the sample was analyzed and the day when the sample was taken + 1. For NfL: a mid-point sample analysis date will be applied, to calculate the plasma sample age. In a second and third sensitivity analysis, Baseline values of MRI whole brain volume and ^{18}F-FDG-PET temporal lobe glucose uptake normalized to pons will be included separately as covariates as well. Factors with a p-value of 0.2 or smaller will be investigated further upon request.

In addition, this primary model will be repeated with the control group being restricted to the subpopulation with lowest parent MT.

This primary model will be repeated (not including the sensitivity analyses and not including the analysis with respect to the lowest parent MT control group) for the subgroups of the stratification variables region, prior use of AChEI and/or memantine, and Baseline MMSE severity (16-19, and 20-27). For the Baseline MMSE severity, the additional split into the subgroups of scores 16-21, and 22-27 will be done as well. This analysis will also be repeated for the verified AD diagnosis subgroups MCI-AD and probable AD.

It is of interest to analyze the correlation between change in NfL and ADAS-cog$_{11}$ accounting for baseline values of NfL and potentially treatment allocation; to address this: the ADAS-cog$_{11}$ change from Baseline at Week 52 will be added as a covariate to a simplified ANCOVA model, additionally only accounting for baseline NfL. In an additional model the ADAS-cog$_{11}$ change from Baseline at Week 104 will be added as a covariate instead. These analyses will be repeated for the verified AD diagnosis subgroups MCI-AD and probable AD, and further repeated replacing ADAS-cog$_{11}$ by ADCS-ADL$_{23}$ and MRI whole brain volume (separately). More advanced versions of this simple model relying on interaction terms or additional covariates might be explored. Analyses targeting a more direct evaluation of the correlation might be explored still accounting for potential covariates. This might be repeated for other potential biomarkers.

It is also of interest to analyze the correlation between baseline values of NfL and ADAS-cog$_{11}$ accounting for the randomization stratification variables (see Section 2.6) in an ANCOVA model with NfL Baseline being the independent variable and ADAS-cog$_{11}$ Baseline the dependent variable. This analysis will be repeated for the verified AD diagnosis subgroups MCI-AD and probable AD, and further repeated replacing ADAS-cog$_{11}$ by ADCS-ADL$_{23}$ and MRI whole brain volume (separately).

TauRx / Cytel Confidential: This is a controlled document and shall not be changed, saved, copied or reproduced by any unauthorized person.

Sample SAS code (will be fully validated at the analysis stage):

PROC MIXED DATA=dataset METHOD=REML COVTEST;

*CLASS subject treatment(REF='Control') severity(REF='16-19') region(REF='Europe') prioruse(REF='None'); /*additional MMSE split: severity(REF='16-21'); by diagnostic criterion: diagnostic(REF='probable AD')*/*

*MODEL diff = treatment severity region prioruse scorebl / SOLUTION CL DDFM= KENWARDROGER; /*diff: change from Baseline*/*

LSMEANS treatment / CL;

*LSMESTIMATE treatment "HMTM 16 vs. Control" 1 0 -1 / CL; /*Pairwise treatment comparison*/*

LSMESTIMATE treatment "HMTM 8 vs. Control" 0 1 -1 / CL;

LSMESTIMATE treatment "HMTM 16 vs. HMTM 8" 1 -1 0 / CL;

ODS OUTPUT LSMeans SolutionF Tests3;

RUN;

7.3.3. Secondary Outcomes

In the following, the changes from Baseline at Week 52 visit will be analyzed for the primary exploratory biomarkers P-tau181 and NfL through pairwise correlations.

These will be analyzed and presented through scatter plots with symbolized treatment group. For each treatment group, both the parametric Pearson correlation coefficient and the non-parametric Spearman's rank correlation coefficient and their corresponding p-values will be presented in this plot, along with the regression lines.

This analysis will be repeated for the Baseline MMSE score subgroups (16-19, 20-27, 16-21, 22-27), and the verified AD diagnosis subgroups MCI-AD and probable AD.

Furthermore, for each primary exploratory biomarker P-tau181 and NfL the change from Baseline at Week 52 visit will be compared pairwise with the change from Baseline at Week 52 for the following scales and parameters:

- ADAS-cog$_{11}$ (after upscaling was applied)
- ADAS-cog$_{13}$ (after upscaling was applied)
- ADCS-ADL$_{23}$ (after upscaling was applied)
- MRI whole brain volume

- MRI lateral ventricular volume
- MRI temporoparietal lobe volume
- ^{18}F-FDG-PET temporal lobe glucose uptake (includes only subjects with a Baseline CDR of 0.5), normalized to pons and normalized to cerebellum

For each primary exploratory biomarker, the correlation between the change from Baseline at Week 52 visit with the predicted steady state plasma level of parent MT at Week 52 will be analyzed as well for the two HMTM treatment groups.

Sample SAS code (will be fully validated at the analysis stage):

```
PROC CORR DATA=dataset pearson spearman;
VAR endpoint1 endpoint2;
RUN;
```

7.3.4. Additional Potential Biomarkers

Additional potential biomarkers are P-tau231, Core-proline tau, T-tau, and GFAP. For each of those biomarkers, if a decision will be made to investigate the respective potential biomarker further, the analyses described in the three previous sections (7.3.1 to 7.3.3) will be repeated. In addition to the pairwise comparison between P-tau181 and NfL, all other pairwise biomarker comparisons will need to be added.

7.3.5. Additional Analysis

The pairwise correlation of Baseline values between the primary exploratory biomarker (P-tau181 and NfL), and the above-mentioned scales and parameters (see Section 7.3.3) will be analyzed irrespective of the treatment group (not per treatment group).

For each of the primary exploratory biomarker (P-tau181 and NfL), an ANCOVA will be used to analyze the natural logarithm of percentage change from Baseline at Week 52. The ANCOVA model will be the same as for the primary outcome (see Section 7.3.2) and the two sensitivity analyses will be performed as well, one including subject's age and sex, as well as *ApoE*4 carrier status and plasma sample age, and the second including MRI whole brain volume and ^{18}F-FDG-PET temporal lobe glucose uptake. Additional analysis will be performed upon request.

TauRx / Cytel Confidential: This is a controlled document and shall not be changed, saved, copied or reproduced by any unauthorized person.

7.4. Future Sample Analysis

If evidence of a beneficial treatment effect of HMTM is apparent for any of the biomarkers, further analysis at all other timepoints (Week 4 and Week 104) for that biomarker will be performed. In such an event, this analysis will be confirmed by amendment to the biomarker research plan, to the test-site-specific plans, and to this SAP, as appropriate.

If beneficial treatment effect of HMTM is apparent for any of the biomarkers, further sample analysis would be required to show pre-dose and post-dose samples are equivalent for that biomarker.

TauRx / Cytel Confidential: This is a controlled document and shall not be changed, saved, copied or reproduced by any unauthorized person.

8. PHARMACOKINETIC EVALUATIONS

A subject data listing with the plasma concentrations (including available modelled parent MT Cmax,ss values) and whole blood concentration data and associated dosing data will be prepared. Calculated values and summary tables will be listed elsewhere.

Statistical Analysis of Pharmacokinetic data will be performed by Certara USA, Inc., and thus its analysis is not within the scope of this SAP but is described elsewhere.

TauRx / Cytel Confidential: This is a controlled document and shall not be changed, saved, copied or reproduced by any unauthorized person.

9. ADNI AND META ANALYSIS

Study data will be compared with ADNI (Alzheimer's Disease Neuroimaging Initiative) data and meta-analysis. This analysis is not within the scope of this SAP but is described elsewhere.

TauRx / Cytel Confidential: This is a controlled document and shall not be changed, saved, copied or reproduced by any unauthorized person.

10. CHANGES TO PLANNED ANALYSES

The exploratory efficacy ^{18}F-FDG-PET parameters inferior temporal gyrus and angular gyrus will not be analyzed, since these are included in the lateral temporal cortex and parietal cortex region of interests, respectively.

The exploratory Bayesian analysis, accounting for and using historic data more generally such as placebo decline or treatment effects as priors to inform the analyses of this study, will not be performed.

After the data snapshot (28-APR-2022), the SAP v2.0 signature and the partial study team members unblinding to perform the analysis of the double-blind treatment phase, the version 2.0 of the SAP has been updated to a version 3.0 with added analyses.

TauRx / Cytel Confidential: This is a controlled document and shall not be changed, saved, copied or reproduced by any unauthorized person.

11. REFERENCES

ICH E9 (R1) addendum on estimands and Sensitivity Analysis in Clinical Trials to the guideline on statistical principles for clinical trials EMA/CHMP/ICH/436221/2017.

Liu, Y., & De, A. (2015). Multiple Imputation by Fully Conditional Specification for Dealing with Missing Data in a Large Epidemiologic Study. International journal of statistics in medical research, 4(3), 287–295. https://doi.org/10.6000/1929-6029.2015.04.03.7

Points to consider on implications of Coronavirus disease (COVID-19) on methodological aspects of ongoing clinical trials, 26 June 2020, EMA/158330/2020 Rev. 1

Ratitch, B., & O'Kelly, M. Implementation of Pattern-Mixture Models Using Standard SAS/STAT Procedures. PharmaSUG 2011 - Paper SP04

Statistical Considerations for Clinical Trials During the COVID-19 Public Health Emergency Guidance for Industry, June 2020, FDA-2020-D-1136

12. APPENDIX

12.1. Considerations regarding COVID-19

This section gives a brief overview of recommendations mentioned therein and their impact to this study regarding its implementation and their applicability. The wording from the guidelines was mostly copied and re-phrased, without changing its essential meaning.

12.1.1. FDA: Statistical Considerations for Clinical Trials During the COVID-19 Public Health Emergency

In this guideline a change of study conduct, requiring a protocol amendment, is proposed to meet the trial objectives, while prioritizing the safety of (all) study participants: This was addressed with the protocol version 6.0 with changes as described in Section 2.5, allowing a less stringent conduct (e.g., remote assessment, use of local laboratories).

Modifications should not be proposed based on data that may introduce bias into the interpretation of trial findings, such as knowledge about magnitude of the treatment effect or information presented by treatment arm: The changes in study conduct suggest no bias, since they should affect all treatment groups likewise.

Information at the participant level should be documented, describing the context and/or reasons for post-Baseline events as they relate to COVID-19, such as discontinuation of treatment, withdrawal of the trial, use of alternative or rescue treatments, missed endpoint ascertainment, and the use of alternative endpoint ascertainment methods: This was addressed with eCRF *Covid-19 Impact Assessment* and the less stringent conduct (remote assessments, local laboratory). Also, corresponding sensitivity analyses were added (see Section 5.9.2.1).

Considerations with respect to the loss of statistical power due to smaller number of randomized subjects are not needed, since the enrollment of patients or study conduct were not stopped early. To overcome the potential loss of information (more generally) from the impact of COVID-19, the number of randomized subjects was increased with study protocol version 6.0.

In an event driven trial the follow-up could be extended: Not applicable.

Sensitivity analyses should be performed examining differences in Baseline characteristics and post-Baseline events (including endpoints and AEs) between the originally enrolled participants and the additional participants to understand the impact of the change in recruitment, including changes to recruitment locations and time of recruitment: Considering that most of the subjects under protocol version 5.0 or higher were randomized during COVID-19 and the quite long follow-up time of 12 months, this is not necessary. Furthermore, enrolment was not extended due to COVID-19 per se.

TauRx / Cytel Confidential: This is a controlled document and shall not be changed, saved, copied or reproduced by any unauthorized person.

Closure of a site for a certain period of time could lead to missing endpoint ascertainments, which may not necessarily be related to the treatment assignment or participant characteristics and outcomes. In this case, removing all participants from closed sites who were scheduled for an endpoint ascertainment from the analysis should not bias the findings. It is important to remove all the participants from the closed sites who were scheduled for the ascertainment, regardless of whether they had previously withdrawn. For this approach, the exclusion of participants should not use post-Baseline participant information but instead use only information at randomization (e.g., site location and randomization date). If a significant number of participants are affected, this strategy may result in a significant loss of information: Remote assessment was enabled to minimize missing endpoint ascertainments.

Similarly, closure of a site for a certain period of time could greatly impact trial-specified treatment for subjects such that it is unlikely that any treatment effect can be observed, then it may be reasonable to exclude participants who were impacted during that period of time. The decision to exclude subjects should not use post-Baseline subject information (e.g., on-treatment time), but instead use Baseline information (e.g., site location and randomization date).

Using alternative ascertainment methods, such as replacing in-person endpoint ascertainment based on performance outcomes or interview-based clinician-reported outcomes with remote ascertainment: Remote assessment and use of local laboratories were established.

Extending the protocol-defined window of time for performing the endpoint ascertainment or using an earlier or later planned ascertainment: The study visit windows were extended.

For a composite endpoint, including additional and clinically relevant components or removing components that cannot be ascertained: For subitems which could not be assessed by remote, those are reported missing and upscaling will be used. Furthermore, only the subset of items for ADAS-Cog and MMSE, which could be administered remotely (see Section 4.5), will be analyzed in a sensitivity analysis.

For a binary endpoint that is based on a continuous or ordinal measurement, using the continuous or ordinal measurement as the endpoint: Not applicable.

Evaluation of the impact of any change in endpoint definition or ascertainment, either through a change in methods or a change in timing, should be carefully evaluated in sensitivity analyses. In particular, any differences in the ascertainment between trial arms or among participants with different Baseline characteristics should be explored: The latter is not applicable, since there are no different approaches between trial arms or based on subjects with different Baseline characteristics. Stratified analysis by way of endpoint ascertainment (in-clinic or remote) will be performed in a sensitivity analysis.

TauRx / Cytel Confidential: This is a controlled document and shall not be changed, saved, copied or reproduced by any unauthorized person.

12.1.2. EMA: Points to consider on implications of Coronavirus disease (COVID-19) on methodological aspects of ongoing clinical trials

In this document, similar to the FDA guideline, the impact analysis should have been performed using unblinded data or being supervised by an independent DMC. Also, the need of a protocol amendment to address changes due to COVID-19. Additional guidance is given regarding the conduct of the study.

Capture systematic deviations resulting from the measures and individual decisions related to the COVID-19 pandemic. Such information will prove valuable in the assessment of the potential impact of these decisions on the trial outcome and should help distinguish between data 'affected' and 'unaffected' by the COVID-19 pandemic. In order to assist efficiently with the identification of deviations related to the COVID-19 pandemic that are of major importance for interpretation of trial results, Sponsors should ensure that their existing systems are able to record pandemic-related protocol deviations and capture related reasons: This was addressed with eCRF *Covid-19 Impact Assessment*.

Data collection should preferably not stop and should continue as long as possible. However, potential risks for study participants when undergoing study-specific procedures take priority in decisions taken. Measures taken in relation to the COVID-19 pandemic may interfere with study treatments, study assessment schedule and individual participants' observation time. It can be expected that study participants within a certain trial will be unequally affected by such general (i.e. external to the trial) COVID-19 pandemic measures: some study participants may already have completed all study relevant activities and recorded measurements before pandemic-related issues started impacting the trial; for other participants, the main individual study phase might fall during a time when it can be affected by the COVID-19 pandemic. Where preparation for the pandemic situation is still possible, investigators should consider which information is essential for the interpretation of the trial and whether an alternative method of data collection might be warranted: The study visit window was extended, remote assessment and use of local laboratories were made possible.

In a pandemic situation, capability and willingness to follow the trial protocol is expected to vary between and within trial participants. All aforementioned issues are assumed to be of particular relevance in multi-center and multi-regional clinical trials. Any attempt to address those issues at the time of study reporting will require information external to the trial concerning COVID-19 pandemic measures per region and per study site. Such information pertains for example to dates and duration of (partial) lockdowns and travel restrictions, as well as any further measures which would affect recruiting study sites. On the individual participant level, any available information concerning COVID-19 testing or infection status should be recorded in trial documentation whenever possible: COVID-19 infections are documented as AE. Subgroup analysis of region is already planned.

Proposals to deal with any identified potential sources of bias comprising identification of newly emerging intercurrent events or missing values, or other unforeseeable required changes to trial elements: Several sensitivity analyses are implemented to deal will missing value and impacted measurements.

TauRx / Cytel Confidential: This is a controlled document and shall not be changed, saved, copied or reproduced by any unauthorized person.

The need/possibility to adjust the trial sample size: See subsection above.

Additional measures when completing the trial after the pandemic, e.g., validation of outcomes that were measured differently: Stratified analysis by way of endpoint ascertainment (in-clinic, remote).

12.2. Scales

12.2.1. ADAS-cog Scoring

Domain	#	Subitem	Maximum Subscore
Memory	1	Word Recall [a, c]	10
	2	Word Recognition [c]	12
	3	Remembering Test Instructions [c]	5
	4	Delayed Word Recall [b, c]	10
Attention	5	Number Cancellation [b]	5
Praxis	6	Constructional Praxis [a, c]	5
	7	Ideational Praxis	5
Orientation	8	Orientation [a, c]	8
Language	9	Naming Objects and Fingers [c]	5
	10	(Following) Commands	5
	11	Spoken Language Ability [a]	5
	12	Word-Finding Difficulty	5
	13	Comprehension [a]	5

[a] Items included in the Composite Scale

[b] Items included in ADAS-cog_{13}, but not in ADAS-cog_{11}

[c] Items included in COVID-19 Composite Scale. The sum of these subitems is 55.

TauRx / Cytel Confidential: This is a controlled document and shall not be changed, saved, copied or reproduced by any unauthorized person.

12.2.2. ADCS-ADL$_{23}$ Scoring

#	Item	Maximum score	#	Item	Maximum score
1	Eating	3	12	Beverage	3
2	Walking	3	13	Cooking and preparation of meals [a]	4
3	Bowel and bladder function at the toilet	3	14	Dispose of garbage or litter	3
4	Bathing	3	15	Travel [b]	4
5	Grooming	3	16	Shopping [b]	4
6a	Dressing Performance	3	17	Keeping appointments [a, b]	3
6b	Physically Dressing Performance	4	18	Left alone [b]	3
7	Use of telephone [a]	5	19	Talk about current events [b]	3
8	Television	3	20	Reading	2
9	Conversation	3	21	Writing	3
10	Cleaning dishes [a]	3	22	Pastime, hobby or game [b]	3
11	Finds personal belongings	3	23	Household appliance	4

[a] Items included in the Composite Scale

[b] Items affected by COVID-19. All other items, whose sum is 58, are included in the COVID-19 Composite Scale.

TauRx / Cytel Confidential: This is a controlled document and shall not be changed, saved, copied or reproduced by any unauthorized person.

12.2.3. **MMSE**

#	Item (alternative item naming)	Maximum Score
1	Temporal orientation (Orientation – Time)	5
2	Spatial orientation (Orientation – Place)	5
3	Immediate memory (Memory – Registration)	3
4	Attention and concentration	5
5	Delayed recall (Memory – Recall)	3
6	Naming (Language – Naming)	2
7	Verbal repetition (Language – Repetition)	1
8	Verbal comprehension (Praxis ideational)	3
9	Writing (Language – Writing Spontaneous)	1
10	Reading a sentence (Language reading Comprehension)	1
11	Constructional praxis (Praxis – Drawing)	1

12.2.4. **CDR-SOB**

#	Item	Maximum Score
1	Memory	3
2	Orientation	3
3	Judgment and Problem Solving	3
4	Community Affairs	3
5	Home and Hobbies	3
6	Personal Care	3

TauRx / Cytel Confidential: This is a controlled document and shall not be changed, saved, copied or reproduced by any unauthorized person.

12.3. Rules for Determining "Worst" Value

12.3.1. Clinical Laboratory Parameters

Rule	Parameters
Highest value	Hematology: eosinophils, basophils, monocytes, reticulocytes<br><br>Blood chemistry: ALT, AST, ALK-P, bicarbonate, creatinine, direct/indirect/total bilirubin, BUN, LDH, TSH, GGT, urea nitrogen
Lowest value	Hematology: neutrophils, RBC count, HCT, hemoglobin, platelets<br><br>Blood chemistry: albumin, creatinine clearance, total protein, Triiodothyronine, Thyroxine, folate, and B12
Farthest from normal range midpoint	Hematology: WBC count, lymphocytes, MCV, MCH, MCHC<br><br>Blood chemistry: glucose (random), sodium, potassium, phosphorus, calcium, chloride

12.3.2. Scales, Vital Signs, and Weight Measurement

Parameter	Rule
ADAS-cog$_{13}$	Highest score
ADCS-ADL$_{23}$	Lowest score
Systolic blood pressure	Value farthest from 125 mmHg
Diastolic blood pressure	Value farthest from 75 mmHg
Pulse	Value farthest from 75 beats per minute
Weight	Greatest weight loss from Baseline

TauRx / Cytel Confidential: This is a controlled document and shall not be changed, saved, copied or reproduced by any unauthorized person.

12.4. ATC Codes or Preferred Terms for Medications

Category of Medications	ATC Codes or Preferred Terms
Antipsychotics	Flagged in the eCRF
MAOIs	N06AF, N06AG (ATC level 4 codes)
SNRIs	Preferred Terms that contain any of the following terms: desvenlafaxine, duloxetine, levomilnacipran, milnacipran, reboxetine, venlafaxine, and vilazodone.
SSRIs	N06AB (ATC level 4 codes)

TauRx / Cytel Confidential: This is a controlled document and shall not be changed, saved, copied or reproduced by any unauthorized person.

13. STATISTICAL OUTPUTS TO BE GENERATED

Will be provided in a separate document.

TauRx / Cytel Confidential: This is a controlled document and shall not be changed, saved, copied or reproduced by any unauthorized person.

14. CLINICAL STUDY REPORT APPENDICES

Will be provided in a separate document.

TauRx / Cytel Confidential: This is a controlled document and shall not be changed, saved, copied or reproduced by any unauthorized person.

28.4.10 Protocol Version 7.1

**Summary of Changes**	**Affected Sections in Revised Protocol (Version 7.1)**
Signatories / Responsible Personnel	
The Global Project Manager has been changed to Marta Medina, with corresponding telephone numbers and email address added.	Section 4 Responsible Personnel
Bjoern Schelter job title has been updated from Data analytics and Biostatistic lead to Chief Analytics Officer	Section 3 Protocol Approval
Vendor Bioclinica removed for Magnetic Resonance Imaging (MRI); suitably qualified vendor added	Section 4 Responsible Personnel
Study Design	
Removed sentence- an interim report will be prepared describing the complete efficacy and safety analyses of the double blind phase.	Section 10.1 General Description
Study Assessments	
Aberdeen Biomedical Imaging centre removed as vendor. Removed sentence any further processing by central reader will be documented in SAP and replaced with Details of analysis conducted will be reported in a separate report.	Section 15.3.3.1 ^{18}F-FDG-PET<br>Section 15.3.3.2 MRI
