# Supplementary material for: Clinical, imaging and blood biomarker outcomes in a Phase 3 clinical trial of tau aggregation inhibitor hydromethylthionine mesylate in mild cognitive impairment and mild to moderate dementia due to Alzheimer’s disease
Source: J Prev Alzheimers Dis. 2026 Jan 21;13(3):100480. doi: 10.1016/j.tjpad.2026.100480 (PMC12861207; doi:10.1016/j.tjpad.2026.100480)
Supplement: Supplementary file 2 [file mmc2.docx]

**Supplementary Appendix:**

TITLE: Clinical, imaging and blood biomarker outcomes in a Phase 3 clinical trial of tau aggregation inhibitor hydromethylthionine mesylate in mild cognitive impairment and mild to moderate dementia due to Alzheimer’s disease

Authors: Claude M Wischik^1,2,*^, Richard Stefanacci^2,3^, Peter Bentham^2,4^, Serge Gauthier^5^, Henrik Zetterberg^6,7,8,9,10,11^, Gordon K Wilcock^12^, Lutz Froelich^13^, Alistair Burns^14^, Emer MacSweeney^15^, Clive Ballard^16^, Jin-Tai Yu^17^, Tay Siew Choon^2^, Vahe Asvatourian^18^, Natalia Muehlemann^18^, Jan Priel^18^, Karin Kook^19^, Tenecia Sullivan^19^, Diane Downie^2^, Sonya Miller^2^, Carol Pringle^2^, John M.D Storey^2, 20^, Tom Baddeley^2,20^, Charles R Harrington^1,2^, Lewis K Penny^1,2,21^, Mohammad Arastoo^1,21^, Roger Staff^22^, Anca-Larisa Sandu^23^, Helen Shiells^2^, Serena Lo^2,3^, Nafeesa Nazlee^2^, Emily Evans^2^, Claire Hull^2^ and Bjoern O Schelter^2,24^

**Supplementary Methods**

**Plasma biomarker analyses**

Plasma was analyzed for NfL using a method validated by Drug Development Solutions by Resolian (Fordham, UK) on the Quanterix Simoa HD-X analyzer, and for p-tau181 by Medpace Reference Laboratories, Cincinnati, OH, USA). For these, measurements were only analysed to 52 weeks since an intervening kit change precluded valid analysis to 104 weeks. P-tau217 was analysed by Meso Scale Diagnostics LLC (Gaithersburg, MD, USA) using the S-PLEX electrochemiluminescence assay. Analysis of samples was blinded to treatment allocation. Statistical analyses were conducted by Cytel (Rotterdam, Netherlands). Analysis of covariance (ANCOVA) was used with fixed effects for treatment group and stratification factors as covariates including baseline MMSE, prior use of AChEI and/or memantine, geographic region, and the respective baseline biomarker value as covariates, and change in the biomarkers from baseline to 52 and/or 104 weeks as the dependent variables. Pairwise correlations were conducted to examine the relationship between changes in the biomarkers at 52 weeks and their correlations with change in clinical and WBV at 52 and 104 weeks.

**Statistical parametric mapping.** T1 whole brain MRI images were processed according to a standard volumetric brain morphology pipeline using the Computational Anatomy Toolbox (CAT12) longitudinal integrated pipeline for voxel brain morphometry (VBM). The number of participants in each group who provided suitable images was 126 for HMTM 16 mg/day, 31 for 8 mg/day, and 163 for MTC 4 mg twice weekly. Data were compared using the longitudinal flexible factorial design with three dose levels and three time points (baseline, 52 and 104 weeks). Locations were assessed within the SPM interface where grey matter change in the control group over 52 or 104 weeks was greater than in those participants receiving HMTM (8 or 16 mg/day groups pooled). Following the standard statistical parametric mapping VBM statistical thresholding approach, an initial threshold of p<0.005 and multiple comparison corrections (false detection rate p<0.05) were applied with a significant cluster level significance of p<0.05). Locations were identified where grey matter change over 52 and 104 weeks in participants randomised to receive MTC was significantly greater than those randomised to HMTM.

**Meta-analysis study selection**

A systematic literature review (SLR) has been conducted and eligible publications identified from the SLR provided for analysis.

PubMed and the Cochrane Library were searched for relevant publications with search terms provided below. Records in clinicaltrials.gov were also hand-searched in case of omission. The inclusion criteria for the meta-analyses were as follows: 1) full-text publication written in English; 2) double-blind, placebo-controlled, randomized clinical trial; 3) patients with probable AD of mild, mild to moderate severity or MCI due to AD; 4) includes treatment duration for at least 52 weeks; and 5) a measure for ADAS-cog, ADL or brain atrophy (whole brain). Studies were excluded for the following reasons: 1) not randomized controlled trials, such as case reports, reviews, or meta-analysis; 2) publication without sufficient information or unable to extract valid outcome data.

The following publication information and patient characteristics were extracted from all studies included: author's name, publication year, sample size, baseline MMSE and diagnostic criteria for AD (amyloid positivity). The following outcome data were extracted where available: change from baseline in whole brain volume, ADAS-cog11, ADAS-cog13, and Alzheimer’s Disease Cooperative Study – Activities of Daily Living (23-item) (ADCS-ADL23).

**Statistical analysis**

Data from the HMTM 16 mg/day and MTC 8 mg/week arms of TRx-237-039 (E-MITT population) were compared against a meta-analysis-pooled placebo data from published studies, in terms of change from baseline of ADAS-cog11, ADAS-cog13, ADCS-ADL23 and whole brain volume at 6 months, 12 months and 18 months. Comparison was performed using a two-sided independent samples t-test with a significance level of 5%.

**Search strategy for PubMed and Cochrane database**

| Database | Search strategy |
| --- | --- |
| PubMed | ((((((((((((((((((Alzheimer Diseases)) OR (Alzheimers Diseases)) OR (Alzheimer Disease)) OR (Alzheimer's Disease)) OR (mild dementia)) OR (Alzheimer Diseases)) OR (Dementia Alzheimer Type)) OR (Alzheimer Type Dementia)) OR (Alzheimer Dementia)) OR (Mild Cognitive Impairment)) OR (mild cognitive impairment)) OR (mild Alzheimer's Disease)) OR (Alzheimer Disease)) OR (Alzheimer Dementias)) OR (Dementia Alzheimer)) OR (Early Alzheimer's Disease)) OR (MCI)) AND ((((((clinical trial) OR (randomized controlled trial)) OR (randomised controlled trial)) OR (RCT)) OR (double-blind)) OR (double blind)) AND (((placebo controlled) OR (placebo)) OR (placebo-controlled )) AND (((((((((((((((((ADAS-Cog) OR (ADAS-cog11)) OR (ADAS-cog12)) OR (ADAS-cog14)) OR (ADAS-cog13)) OR (Alzheimer's Disease Assessment Scale)) OR (ADAS)) OR (ADCS-ADL)) OR (ADCS-MCI-ADL)) OR (Whole brain volume)) OR (Daily Living Scale)) OR (WBV)) OR (Activities of Daily Living Scale)) OR (MRI)) OR (volumetric MRI)) OR (magnetic resonance imaging)) OR (ADL)) OR (Alzheimer's Disease Cooperative Study-Activities of Daily Living Scale)) |
| Cochrane Library | Search term: ‘Alzheimer’s Disease’ or ‘MCI’ or ‘ADAS’  Filter applied: ‘clinical trials’ |

**Supplementary Figure 1: Correlation between change from baseline in plasma NfL and CDR-SB at 52-weeks in MCI**

**Supplementary Figure 2: Comparison of change in NfL concentration and progression on the CDR scale over 12 months in MCI-AD participants with baseline CDR 0.5**

**Supplementary Table 1: Demographic and baseline characteristics in MCI subpopulation**

|  | |  | **Control**  **(MTC 4 mg twice weekly)** | **HMTM 8mg/day** | **HMTM 16mg/day** | **Overall** |
| --- | --- | --- | --- | --- | --- | --- |
|  | |  | n=101 | n=29 | n=98 | n=228 |
| Age (years) | |  |  |  |  |  |
|  | Mean (SD) |  | 70.8 (8·2) | 71·2 (9·1) | 69·9 (8·0) | 70·5 (8·2) |
|  | Median (range) |  | 70·0 (50-87) | 73·0 (48-84) | 71·0 (54-85) | 71·0 (48-87) |
| Sex, n (%) | |  |  |  |  |  |
|  | Male |  | 44 (43·6) | 13 (44·8) | 31 (31·6) | 88 (38·6) |
|  | Female |  | 57 (56·4) | 16 (55.2) | 67 (68.4) | 140 (61.4) |
| MMSE | |  |  |  |  |  |
|  | Mean (SD) |  | 23.3 (2·9) | 23.0 (3·1) | 23.6 (2·9) | 23.4 (3·0 ) |
| Years since AD diagnosis, mean (SD) | | | 2.7 (3·4) | 2.7 (2·1) | 2.2 (2·2) | 2.5 (2·8) |
| History of using ChEI and/or memantine, n (%) | | | 29 (28·7) | 9 (31·0) | 25 (25·5) | 63 (27·6) |
| Race, n (%) | | |  |  |  |  |
|  | Asian |  | 0 | 1 (3·4) | 0 | 1 (0·4) |
|  | Black or African American | | 5 (5.0) | 1 (3.4) | 2 (2·0) | 8 (3·5) |
|  | Native Hawaiian or Other Pacific Islander | | 0 | 0 | 1 (1·0) | 1 (0·4) |
|  | White |  | 94 (93·1) | 27 (93·1) | 84 (85·7) | 205 (89·9) |
|  | Unknown |  | 2 (2·0) | 0 | 9 (9·2) | 11 (4·8) |
|  | Other |  | 0 | 0 | 1 (1·0) | 1 (0·4) |
|  | Multiple Races Checked |  | 0 | 0 | 1 (1·0) | 1 (0·4) |
| Ethnicity, n (%) | |  |  |  |  |  |
|  | Hispanic or Latino |  | 41 (67·2) | 12 (66·7) | 38 (69·1) | 91 (67·9) |
| Geographic Region, n (%) | |  |  |  |  |  |
|  | Europe |  | 39 (38·6) | 11 (37·9) | 41 (41·8) | 91 (39·9) |
|  | North America |  | 62 (61·4) | 18 (62·1) | 57 (58·2) | 137 (60·1) |
| Presence of *APOE* ε4 allele, n (%) | | | |  |  |  |
|  | Positive |  | 47 (52·2) | 12 (44·4) | 38 (42·2) | 97 (46·9) |
|  | Negative |  | 43 (47·8) | 15 (55·6) | 52 (57·8) | 110 (53·1) |

**Supplementary Table 2: Change from baseline for MCI population in ADAS-cog_13_, ADCS-ADL_23_ and WBV over 52-weeks in HMTM 16mg/day and MTC 4mg twice weekly versus meta-analytic controls**

| **Outcome** | **Time point**  **(months)** | **Number of participants (Number Trials) for TauRx / Meta** | **TRx-237-039**  **MTC 4mg twice weekly**  **Mean ± SE** | **Meta-analytic control**  **Mean ± SE** | **p-value**  **(2-sided)** |
| --- | --- | --- | --- | --- | --- |
| ADAS-cog_13_ | 6 | 93 (1) / 2197 (5) | -1.903 ± 0.556 | 1.097 ± 0.124 | 1.36x10^-7^ |
|  | 12 | 85 (1) / 2617 (8) | -0.686 ± 0.740 | 2.896 ± 1.101 | 0.0069 |
| ADCS-ADL_23_ | 6 | 92 (1) / 2077 (5) | 2.638 ± 0.727 | -1.574 ± 0.399 | 3.788x10^-7^ |
|  | 12 | 86 (1) / 2031 (6) | 1.704 ± 0.825 | -3.229 ± 0.525 | 4.5x10^-7^ |
| WBV (cm^3^) | 6 | 89 (1) / 2226 (8) | -5.297 ± 0.760 | -8.175 ± 1.161 | 0.0381 |
|  | 12 | 83 (1) / 2659 (10) | -8.968 ± 1.125 | -13.369 ± 1.207 | 0.0076 |

**Supplementary Table 3: Spearman’s correlation coefficients between baseline plasma pTau217 and NfL concentrations and cognitive and neuroimaging endpoints in the whole population**

| **Outcome Measure** | **pTau217** | | **NfL** | |
| --- | --- | --- | --- | --- |
|  | **Spearman’s rho** | **p-value** | **Spearman’s rho** | **p-value** |
| ADAS-cog_11_ | 0.429 | <0.0001 | 0.284 | <0.0001 |
| ADAS-cog_13_ | 0.477 | <0.0001 | 0.304 | <0.0001 |
| ADCS-ADL_23_ | 0.099 | 0.0588 | -0.017 | 0.7415 |
| MRI whole brain volume | -0.129 | 0.0139 | -0.188 | 0.0003 |
| MRI lateral ventricular volume | 0.338 | <0.0001 | 0.428 | <0.0001 |
| MRI temporoparietal | -0.284 | <0.0001 | -0.259 | <0.0001 |
| PET temporal lobe glucose uptake (pons) | -0.625 | <0.0001 | -0.452 | <0.0001 |

**Principal Investigators from Participating Clinical Sites:**

The table below has been provided to acknowledge the clinical site Principal Investigators (PIs) who significantly contributed to the global Phase 3 Study TRx-237-039 by way of screening and recruiting subjects and study partners.

**Principal Investigators from Participating Clinical Sites by Country**

| **Principal Investigator**  **(Last name, first name)** | **Institution Name** | **City** | **State/Province/**  **Region** |
| --- | --- | --- | --- |
| **Canada** |  |  |  |
| Bergeron, Richard | Recherches Neuro-Hippocampe Inc.- Clinique Mémoire de l’Outaouais  La Clinique de la Mémoire | Gatineau  Ottawa | Québec  Ontario |
| Okorie, Eugene | Okanagan Clinical Trials, Ltd | Kelowna | British Columbia |
| Patry, Claude | Alpha Recherche Clinique | Québec | Québec |
| **France** |  |  |  |
| Auriacombe, Sophie | Hôpital Pellegrin - CHU de Bordeaux | Bordeaux | Nouvelle-Aquitaine |
| Bennys, Karim | Hôpital Guide de Chauliac | Montpellier | Occitania |
| Boutoleau-Bretonniere, Claire | Hôpital Laënnec - CHU de Nantes | Nantes | Pays de la Loire |
| Ceccaldi, Mathiu-Pierre | Hôpital de la Timone | Marseille | Provence-Alpes-Côte d'Azur |
| Hanta, Cezara-Roxana | Hôpital Pontchaillou - CHU de Rennes | Rennes | Brittany |
| Jing, Xie | Hôpital des Charpennes | Villeurbanne | Auvergne-Rhône-Alpes |
| Mollion, Hélène | Hôpital Neurologique Pierre Wertheimer | Bron | Auvergne-Rhône-Alpes |
| Ousset, Pierre-Jean | Hôpital La Grave – Cité de la santé | Toulouse | Occitania |
| Tchalla, Achille | Hôpital Dupuytren - CHU de Limoges | Limoges | Nouvelle-Aquitaine |
| **Italy** |  |  |  |
| Cassetta, Emanuele | Ospedale San Giovanni Calibita Fatebenefratelli | Rome | Lazio |
| Ferrarese, Carlo | Azienda Ospedaliera San Gerardo - Clinica Neurologica | Monza | Lombardy |
| Galluzzi, Samantha | IRCCS Istituto Centro San Giovanni di Dio Fatebenefratelli | Brescia | Lombardy |
| Gigli, Gian Luigi | Clinica Neurologica Santa Maria della Misericordia | Udine | Friuli-Venezia Giulia |
| Giubilei, Franco | Azienda Ospedaliera – Universitaria Sant'Andrea | Rome | Lazio |
| Grimaldi, Luigi | Foundation Institute G.Giglio | Cefalù | Sicily |
| Mecocci, Patrizia | Ospedale Santa Maria della Misericordia, University of Perugia | Perugia | Umbria |
| Pellicano, Clelia | IRCCS Fondazione Santa Lucia | Rome | Lazio |
| Sinforiani, Elena | Istituto Neurologico Casimiro Mondino- I.R.C.C.S | Pavia | Lombardy |
| **Poland** |  |  |  |
| Czarnecki, Maciej | Centrum Medyczne NeuroProtect | Warsaw | Mazowieckie |
| Dobryniewski, Jacek | Podlaskie Centrum Psychogeriatrii | Bialystok | Podlaskie |
| Ilkowski, Jan | NZOZ Neuro-Kard | Poznan | Wielkopolskie |
| Lisewski, Pawel | Centrum Medyczne Neuromed | Bydgoszcz | Kujawsko-Pomorskie |
| Potemkowski, Andrzej | Euromedis Sp. z o.o. | Szczecin | Zachodniopomorskie |
| Szczechowski, Lech | NZOZ Wielospecjalistyczna Poradnia Lekarska Synapsis | Śląskie | Silensia |
| Szczepańska-Szerej, Anna | Indywidualna Praktyka Lekarska  Dr Hab. Med. Anna Szczepańska-Szerej | Lublin | Lubelskie |
| **Spain** |  |  |  |
| Balaguer Martinez, Ernest | Hospital General de Catalunya | Barcelona | Catalonia |
| Arroyo Gonzalez, Rafael | Hospital Universitario  QuironSalud Madrid | Madrid | Madrid Community |
| Krupinski Bielecki, Jerzy | Hospital Universitario Mutua Terrassa | Barcelona | Catalonia |
| Landete, Lamberto | Hospital Universitario Doctor Peset | Valencia | Valencia Community |
| Montejo, Angel | Centro de Salud de San Juan Unidad de Investigación Neurociencias | Salamanca | Castile-León |
| Olazaran, Javier | Hospitales de Madrid | Madrid | Madrid Community |
| Villarejo, Alberto | Hospital Universitario 12 de Octubre | Madrid | Madrid Community |
| Viñuela Fernández, Félix | Hospital Universitario Virgen de la Macarena | Seville | Andalusia |
| **United States of America** | |  |  |
| Acosta, Ana | Advance Medical Research Center | Miami | Florida |
| Alvarez, Alluska | Biomed Research Institute, Inc. | Miami | Florida |
| Bear, David | Emerald Coast Center for Neurological Disorders | Pensacola | Florida |
| Block, Allan | Imagining Endpoints Research | Scottsdale | Arizona |
| Bolouri, Mohammad | AMC Research, LLC | Matthews | North Carolina |
| Cardona, Jose (and Dr Perez) | Indago Research & Health Center, Inc. | Hialeah | Florida |
| Celmins, Dzintra | Albany Medical College | Albany | New York |
| Cevallos-Yepez, Jose | Finlay Medical Research | Greenacres | Florida |
| Drake, Ryan | Neuroscience Research Center, LLC | Canton | Ohio |
| Duffy, John | Syrentis Clinical Research | Santa Ana | California |
| Escobar-Lopez, Jose (and Elizabeth Mones) | Health Care Family Rehab and Research | Miami | Florida |
| Faradji, Victor | Visionary Investigators Network | Miami | Florida |
| Feldman, Robert | Senior Clinical Trials, Inc. | Laguna Hills | California |
| Flitman, Stephen | Xenoscience | Phoenix | Arizona |
| Garcia, Ernesto | L&C Professional Medical Research Institute | Miami | Florida |
| George, Kristi | Josephson Wallack Munshower Neurology P.C. | Indianapolis | Indiana |
| Gonzalez Rojas, Yaneicy | Optimus Clinical Research | Miami | Florida |
| Graff, Jason | The Lindner Research Center | Cincinnati | Ohio |
| Greeley, David | Kingfisher Cooperative, LLC | Spokane | Washington |
| Holdeman, Troy | Heartland Research Associates | Newton | Kansas |
| Karakoc, Tayfun | Texas Center for Drug Development Inc. | Houston | Texas |
| Karathanos, Michael | Tulsa Clinical Research LLC | Tulsa | Oklahoma |
| Keegan, Andrew | The Roskamp Institute, Inc. | Sarasota | Florida |
| Kirk, Gregory | Merrit Island Medical Research | Merrit Island | Florida |
| Leon, Ramon (and Dr Lopez-Brignoni) | IMIC Inc. | Palmetto Bay | Florida |
| Lesch, David | Georgia Neurology and Sleep Medicine Associates | Suwanee | Georgia |
| Mahdad, Mehrdad | HB Clinical Trials Inc. | Fountain Valley | California |
| Malhotra, Shishuka | Neuro-Behavioral Clinical Research, Inc. | North Canton | Ohio |
| Mazzeo, Paul | Coastal Neurology | Port Royal | South Carolina |
| Mega, Michael | Neural Net Research | Portland | Oregon |
| Nash, Marshall | NeuroStudies.net, LLC | Decatur | Georgia |
| Padala, Prasad | Atria Clinical Research | Little Rock | Arizona |
| Patel, Meenakshi | Valley Medical Research | Centerville | Ohio |
| Pelayo, Enrique | Advanced Medical Research Institute | Miami | Florida |
| Pfeffer, Michael | Allied Biomedical Research Institute | Miami | Florida |
| Plopper, Michael | Sharp Mesa Vista Hospital | San Diego | California |
| Ponce De Lion, Mercedes | Vitae Research Center, LLC | Miami | Florida |
| Powell, Richard | Meridien Research | Spring Hill | Florida |
| Puente, Orlando | Miami Dade Medical Research Institute, LLC | Miami | Florida |
| Ricart, Francisco | CCM Clinical Research Group | Miami | Florida |
| Rodriguez-Ables, Lilia | Finlay Medical Research | Miami | Florida |
| Safirstein, Beth | MD Clinical | Hallandale Beach | Florida |
| Schwartzbard, Julie | Visionary Investigators Network | Aventura | Florida |
| Shankle, William | Hoag Memorial Hospital Presbyterian | Newport Beach | California |
| Sharma, Sanjiv | Advanced Memory Research of NJ PC | Toms River | New Jersey |
| Shiovitz, Thomas | California Neuroscience Medical Group | Sherman Oaks | California |
| Soefje, Sherry | Excell Research Inc. | Oceanside | California |
| Stedman, Mary | Stedman Clinical Trials | Tampa | Florida |
| Szigeti, Kinga | UBMD Neurology | Buffalo | New York |
| Taylor, James (and Goodman, Ira) | Bioclinica Research | Orlando | Florida |
| Taylor, Kelly | Sensible Healthcare | Oocee | Florida |
| Thurman, Louise | IPS Research Company | Oklahoma City | Oklahoma |
| Trueba, Pilar | Future Care Solution, LLC | Miami | Florida |
| Turner, Mark | Advanced Clinical Research | Boise | Idaho |
| Turner, Scott (and Dr Lamotte, Dr Bicksel) | Re-Cognition Health | Fairfax | Virginia |
| Valor, Elena | Florida International Research Center (FIRC) | Miami | Florida |
| Vandersluis, Joel | Neurology Diagnostics Inc. | Dayton | Ohio |
| Watson, David | Alzheimer’s Research and Treatment Center | Wellington | Florida |
| White, Alexander | Progressive Medical Research | Port Orange | Florida |
| **United Kingdom** |  |  |  |
| Lynch, Jennifer | Glasgow Memory Clinic Ltd | Glasgow | Lanarkshire, Scotland |
| MacSweeney, Emer | Re-Cognition Health | Birmingham  Guildford  London | West Midlands,  Surrey  Greater London,  England |
| Pearson, Stephen | Re-Cognition Health | Plymouth | Devon, England |
